# A QSP Model for Predicting Clinical Responses to Monotherapy, Combination and Sequential Therapy Following CTLA-4, PD-1, and PD-L1 Checkpoint Blockade

**DOI:** 10.1038/s41598-019-47802-4

**Published:** 2019-08-02

**Authors:** Oleg Milberg, Chang Gong, Mohammad Jafarnejad, Imke H. Bartelink, Bing Wang, Paolo Vicini, Rajesh Narwal, Lorin Roskos, Aleksander S. Popel

**Affiliations:** 1 Department of Biomedical Engineering, Johns Hopkins University School of Medicine, Baltimore, Maryland USA; 2 Clinical Pharmacology, Pharmacometrics and DMPK (CPD), MedImmune, South San Francisco, California USA; 3 Clinical Pharmacology, Pharmacometrics and DMPK, MedImmune, Cambridge, United Kingdom; 4 MedImmune, Gaithersburg, Maryland USA; 5 Department of Clinical Pharmacology and Pharmacy, Amsterdam UMC, Vrije Universiteit Amsterdam, Amsterdam, The Netherlands; 6 The Sidney Kimmel Comprehensive Cancer Center, Johns Hopkins University School of Medicine, Baltimore, Maryland USA

**Keywords:** Melanoma, Cancer models, Computational models, Dynamical systems

## Abstract

Over the past decade, several immunotherapies have been approved for the treatment of melanoma. The most prominent of these are the immune checkpoint inhibitors, which are antibodies that block the inhibitory effects on the immune system by checkpoint receptors, such as CTLA-4, PD-1 and PD-L1. Preclinically, blocking these receptors has led to increased activation and proliferation of effector cells following stimulation and antigen recognition, and subsequently, more effective elimination of cancer cells. Translation from preclinical to clinical outcomes in solid tumors has shown the existence of a wide diversity of individual patient responses, linked to several patient-specific parameters. We developed a quantitative systems pharmacology (QSP) model that looks at the mentioned checkpoint blockade therapies administered as mono-, combo- and sequential therapies, to show how different combinations of specific patient parameters defined within physiological ranges distinguish different types of virtual patient responders to these therapies for melanoma. Further validation by fitting and subsequent simulations of virtual clinical trials mimicking actual patient trials demonstrated that the model can capture a wide variety of tumor dynamics that are observed in the clinic and can predict median clinical responses. Our aim here is to present a QSP model for combination immunotherapy specific to melanoma.

## Introduction

While researchers and clinicians first hypothesized and found evidence for the ability of immune cells to target and eliminate cancer in the late 19^th^ and early 20^th^ centuries, which was later termed immunosurveillance, it was not widely accepted and clinically proven until the 21^st^ century^1,2^. Immunosurveillance has become part of a broader concept called immunoediting, which defines the stages of cancer growth: elimination, equilibrium and escape^3^. The preemptive immune cells (primarily the effector T cells) that eliminate cancer cells can eliminate the tumor in its entirety, come to a point where elimination is in dynamic equilibrium with tumor growth, or the tumor can overcome the immune response against it by dampening the immune response and outgrowing it. Such outgrowth can occur when the tumor size and doubling time together far exceed the abundance and effectiveness of the immune response. Immune responses are dampened due to several factors, including the resistance put forth by cancer cells through cytokine or immune checkpoint expression, in a similar manner by tumor-associated cells (e.g., T-regulatory cells, Tregs, and myeloid-derived suppressor cells, MDSCs) and through variations in tumor immunogenicity that lower the formation of an effective tumor-specific immune response; considering that immune cell access to the tumor is equally possible^4^. Some types of tumors are naturally more immunogenic than others, with melanoma being the most immunogenic^5,6^.

### Immunogenicity, the Mounting of an Anti-tumor Response and its Resistance by the Tumor

The immunogenicity of a tumor, which is the ability of an immune response to be mounted against it, is a function of its tumor burden (i.e., the abundance of tumor antigens recognized by the immune cells) and its antigen mutational landscape (i.e., the variety and strength of those antigens)^7^. Tumors with antigens more abundant, stronger and varied in sequence, are more immunogenic and may activate more T cells against the tumor^8–10^.

Tumor-associated or tumor-specific antigens (TAA or TSA, respectively) are usually proteins, peptides or other factors that are products of the cancer cells^11^. In larger tumors (with higher tumor burden), more TAA and TSA are released through natural death and decay of cancer cells during growth, and during killing of cancer cells by lymphocytes. TAA (used from now on) can be picked up by antigen presenting cells (APCs), which mature (to mAPCs) and present the TAA to naïve T cells during priming to induce their differentiation into activated tumor-targeting effector T cells, which then can eliminate the tumor through cytotoxic activity.

During the process of antigen presentation, each naïve T cell can recognize a specific TAA by its unique T cell receptor (TCR). A T cell with a TCR specific to a TAA is called a clone. There are usually several copies of each clone specific to each TAA available for priming and activation. The more unique TAA a tumor releases, the more T cell clones can be primed and activated to target the tumor. Additionally, the strengths of the different antigens have been classified from weak to strong; the exact reason for why one antigen is stronger or weaker than another is not entirely clear and has been linked to several factors, including the affinity (K_d_) and binding ability between the antigen presented on the major histocompatibility complex (MHC) and its cognate TCR during antigen presentation. Upon the successful elimination (death) of cancer cells by the effector T cells, TAA associated with those cancer cells are released into the environment and thus can be used to prime more effector cells to mount a subsequent attack against the tumor; some non-specific cytotoxic activity by the T cells is also thought to occur by infiltrating effector T cells^12^.

As described, an effective immune response is dictated by the number of T cell clones, tumor burden (or antigen abundance, which is assumed proportional to tumor size) and antigen strength. Considering that an effective immune response can be generated, to eliminate the tumor it must overcome its growth rate and resistance mechanisms. The focus of this study is on the resistance to cancer elimination by immune checkpoints Cytotoxic T-lymphocyte-associated protein 4 (CTLA-4), Programmed cell death protein 1 (PD-1), and Programmed death-ligand 1 (PD-L1) and how their blockade by immune checkpoint inhibitors can lead to a range of patient responses through variations in specific physiological parameters, such as those described above (i.e., numbers of T cell clones, tumor size and antigen strength), encoded in a mechanistic framework. Other key parameters mechanistically considered are the expression levels of the immune checkpoints (particularly PD-L1 on cancer cells, which is a known biomarker of anti-PD-1 efficacy), and inhibitory immune cells, such as T-regulatory cells present in the lymph nodes and the tumor microenvironment. While immune checkpoints in recent years have been found to be prime targets for cancer immunotherapy in melanoma, non-small cell lung cancer (NSCLC), bladder cancer and other cancer types, we particularly examine them here for melanoma^13,14^.

### Immune checkpoint blockade therapies

Both PD-1 and PD-L1 are expressed on effector T cells, cancer cells, as well as the mentioned regulatory immune cells in the tumor microenvironment (TME)^15–17^. When PD-1 interacts with PD-L1, the effector T cells can be prevented from cytotoxic activity and may become anergic; in this way the tumor hijacks the signaling pathways for its own survival. Furthermore, CTLA-4 checkpoints have been found on T cells and play an important role during priming in the activation of effector T cells; their blockade can help boost effector T cell numbers^18,19^. Other checkpoints have also been identified: those found that are most relevant to this study include the ones mentioned above, as well as the co-stimulatory B7 receptors (CD80 and CD86) and CD28, and PD-L2, which also interacts with PD-1^20–23^.

### Mathematical models for immunotherapy and their utility

Several mathematical models of the immune system have been developed thus far; only a small number of them focus on immuno-oncology and immunotherapies, as described here^24–26^. Previous models of immunotherapies have focused on single chemotherapies, combinations of chemotherapies, radiation therapy, a cancer vaccine and IL-2 immunotherapy, or focused on a specific molecule or pathway^27–31^. Quantitative Systems Pharmacology (QSP) approaches to cancer immunotherapy are currently being pursued in academia and industry^32^. It is our belief that computational models, such as the one presented here, will play a substantial role in reducing costs and expediting the drug development and approval processes to bring therapies to market quicker, especially for combination therapies^33^.

Much is still unknown about how and why only some patients benefit from particular therapeutic regimens^34,35^. In order to make personalized cancer immunotherapy a reality, it is necessary to gain a better understanding of the physiological system dynamics at play on both the cellular and molecular levels for each cancer patient, which may ultimately help determine the optimal personalized dosing, regimens and combination strategies. Here, we aim to link how the mentioned physiological parameters that we described biologically (i.e., numbers of T cells clones, tumor size, average antigen strength, expression of PD-L1 on cancer cells, as well as the levels of inhibitory cells in the tumor and lymph node) when put into a well-mixed mechanistic framework and their values varied across defined ranges, can allow us to simulate a variety of anti-tumor responses when the dose level and regimen of each therapy is kept constant. We further show that the median values of our model parameters can be used to predict median responses to various combinations of CTLA-4, PD-1 and PD-L1 blockade therapies that have been reported in the literature specific to the doses and regimens. Lastly, we show the significances of the parameters we have varied in terms of how they may correlate to different types of patient responders, in addition to how variations in these parameters can lead to different types of tumor dynamics. We use this model to make predictions on outcomes of combination therapies and believe it can be used to predict various dosing regimens for optimization purposes within the given framework.

### Choice of virtual patient population

Different methods have been utilized in the literature to create a virtual patient (VP) population dataset; yet it is still an open question of how to define the most representative VP population for each clinical trial. One method defines “plausible virtual patients” within a virtual population (Vpop) using a sampling/acceptance-rejection algorithm with applied prevalence weights to each VP to make sure the VP cohort spans the entire range of clinically observed responses; additionally, stochastic noise can add more variability to this dataset; another method relies on simply fitting parameters within a constrained range to the anti-tumor response data a certain number of times to define the VP set^36,37^. We have employed a technique similar to the latter, where we specifically defined the mentioned parameter ranges quantitatively and qualitatively from literature sources and varied them using a particular distribution method within those ranges to create a multidimensional array of patient parameters for the number of patients we wanted to simulate responses for. Primarily, we assumed that if our model is a representative abstraction of the true mechanism underlying these therapies, we should see a range of responses similar to clinical data. We further primarily focus on the biological parameters discussed, while the remaining parameters in the model were set to their defined averages and not varied. Table S11 lists the parameter ranges and the median values used to define the VP populations for each simulation. We recognize that the question of how to build a proper VP cohort is open and further studies are required to better define it.

## Results

### Model baseline optimization to anti-PD-1 clinical trial outcomes

**Figure 1:**
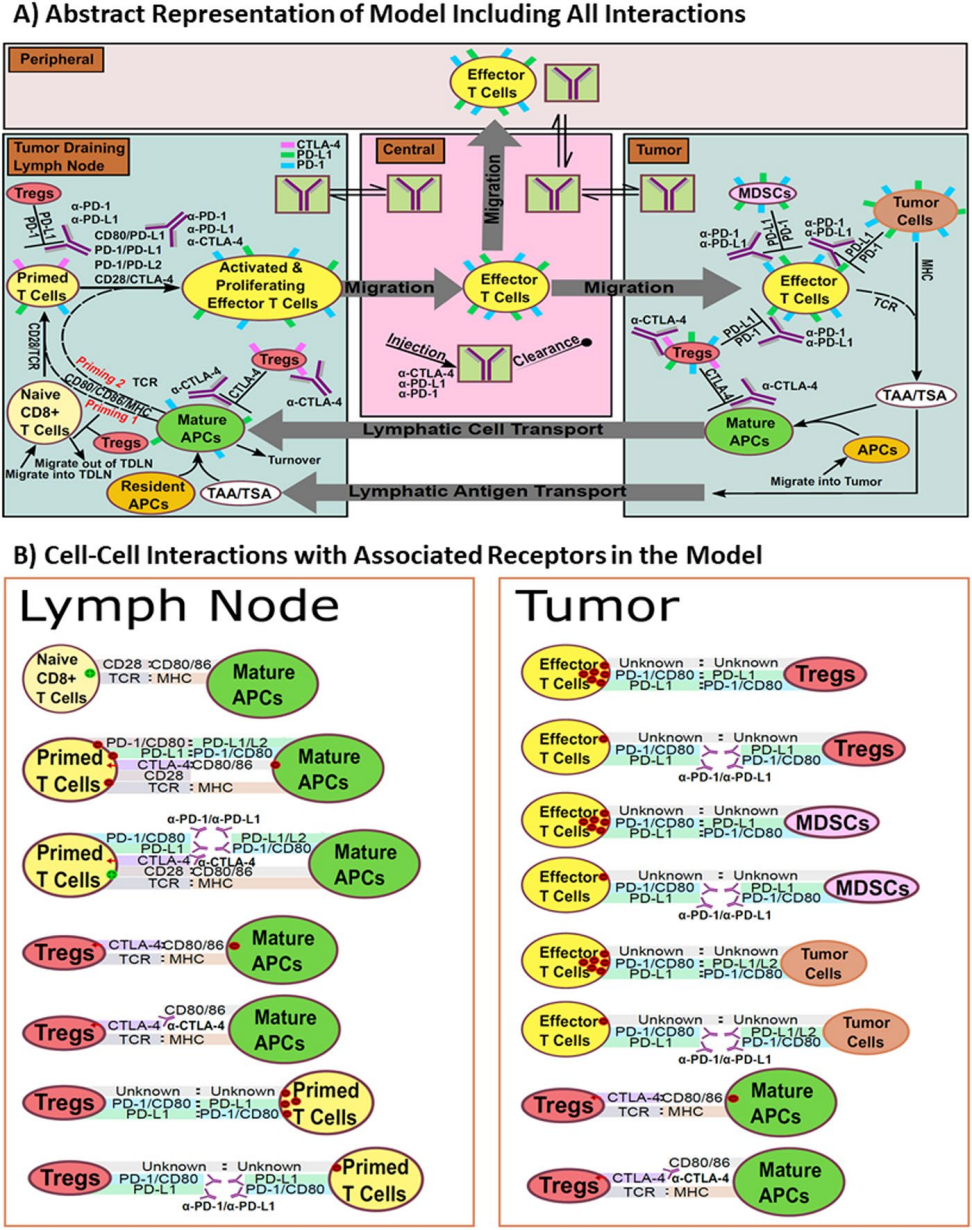
Diagram of model. (**A**) Compartments, and their associated cellular-molecular interactions and distributions are shown. The entire model represents the system of a single VP for each model run given a set of parameters. (**B**) All checkpoint and associated antibody interactions linked to each cell type in the model.

The developed QSP model is depicted in graphical form in Figs 1, S7–S9. These represent the main compartments and the cellular and molecular interactions occurring within them. Physiological trafficking of effector T cells was mathematically represented, as described previously by Zhu *et al*.^38^. The pharmacokinetics (PK) was represented by a minimal physiologically-based pharmacokinetic (mPBPK) model^39^. The model was first optimized to an anti-PD-1 therapy clinical trial dataset and more qualitatively to match the level of tumor responses to anti-CTLA-4 therapy. Certain fit parameters were then used to make predictions for other therapeutic regimens with simulations and compared to clinical responses; the only difference between all the simulated trials were the regimens and dose levels of the specific therapies, as follows.

**Figure 2:**
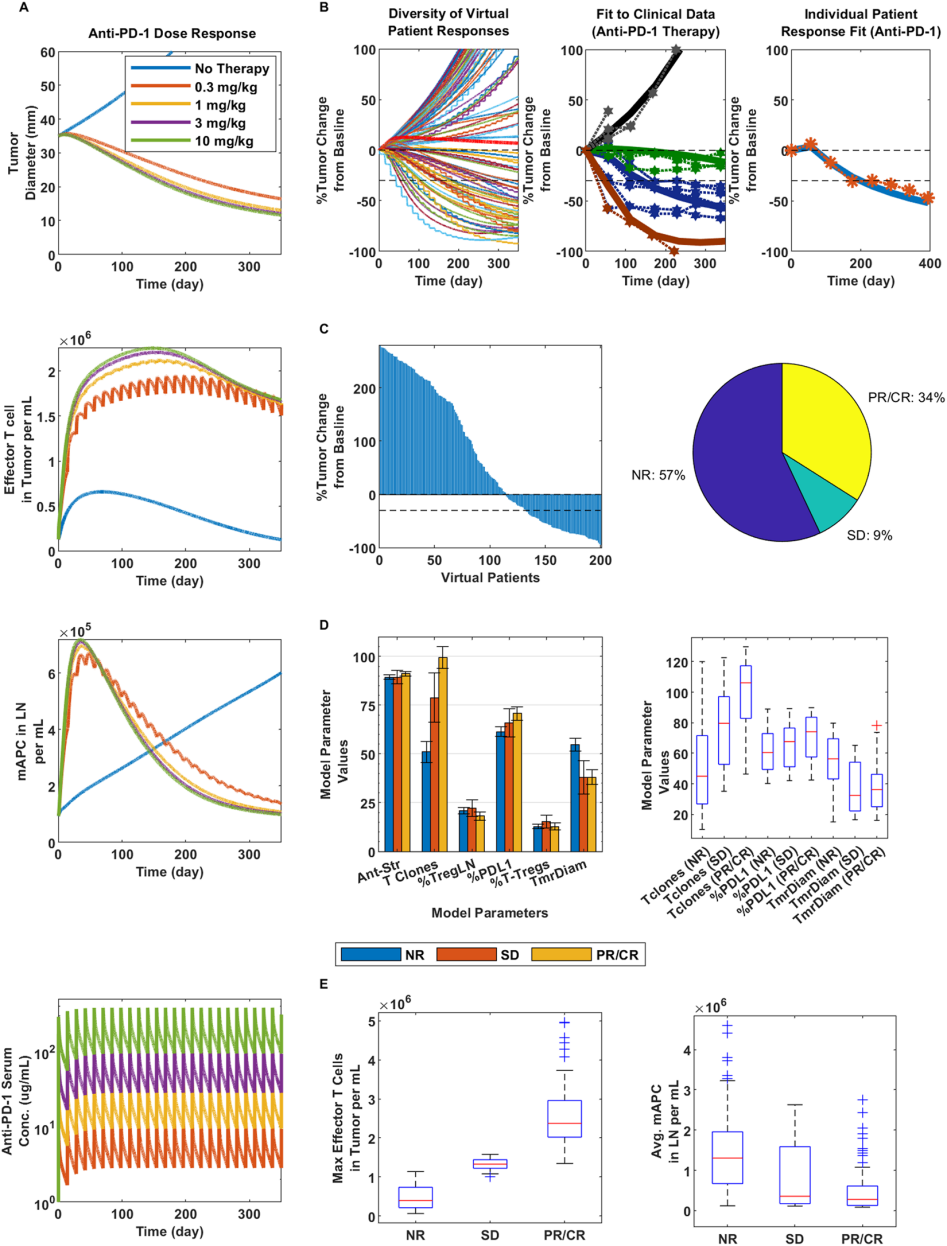
Dose response and clinical validation of anti-PD-1 monotherapy. (**A**) From top to bottom: Tumor response to anti-PD-1 therapy at doses of 0.3, 1, 3 and 10 mg/kg as represented by the colors in the bottom figure in ascending order; the blue line indicates no therapy (top figure). Then, effector T cell density in the tumor (second from the top), mAPC density in the lymph nodes (third from the top) and finally, the PK of anti-PD-1 at the given doses. For all following figures, 3 mg/kg anti-PD-1 was used, following the same regimen. (**B**) Diversity of tumor response (left), fitting to pooled means patient response data (center) and individual patient fit (right); all at 3 mg/kg anti-PD-1. (**C**) Waterfall plot of VPs (left) and pie chart (right) with percent of virtual non-responders (NR), stable disease (SD) and partial or complete responders (PR/CR). (**D**) Bar graph comparison of parameters varied in model for each responder type (left) and box plots of significant differentiators (right). (**E**) Max effector T cell density in the tumor (left) and average mAPC density in the lymph nodes (right) for each responder category.

To initially parameterize the model and demonstrate that it can emulate clinical results, we fit the model to an anti-PD-1 clinical trial conducted by Topalian *et al*. (ClinicalTrials.gov number, NCT00730639)^40^. Figure 2 shows anti-PD-1 simulated dose responses, effector and mAPC dynamics and the PK (Fig. 2A), indicating a largely flat dose response after 1 mg/kg (yellow), as known to occur clinically^41^. Figure 2B, middle, shows the pooled fit to clinical data for progressive disease (black line), stable disease (green line), partial response (blue line) and complete response (red line). For the fitting, the mentioned parameters listed in Table S9 were constrained within their given ranges, while supporting parameters, such as chemokine factor and antigen abundance per cell (that were not varied in subsequent simulations), were also fit to be fixed at a single value for each. The goal was to show that given a fixed set of parameters in the model space, VP sets can then be simulated and compared with clinical data through the variability of the mentioned parameters in Table S11. Figure 2B, right, shows the fit to a single patient responder (by constraining the parameters in Table S11 to their ranges, while keeping all other parameters constant in the model), indicating that that model can capture the delay in response (over 50 days from start of therapy). Altogether, this demonstrates that the model captures several different types of responders within the defined parameter ranges that make up our VP dataset.

While we were able to reasonably fit and capture human clinical tumor response using the set of parameters and 3 mg/kg anti-PD-1 Q2W (dosing every two weeks), we realized that there were several combinations of values for the given parameters within their defined ranges that could lead to the same or similar responses. As a result, we simulated a 200-patient virtual clinical trial using the same regimen by varying the mentioned parameters defined in Table S11 simultaneously using Latin Hypercube Sampling (LHS) method; we show the diversity of responses in Fig. 2B, left. Figure 2C shows a waterfall plot of the simulated responders (left) and that responders represent 43% of total VPs (right) and 34% that were partial/complete responders (PR/CR), approximating the upper end of a known objective response rate (ORR) for PD-1 therapy for melanoma of 43.7%^42^. Figure 2D further shows that of the varied parameters, the number of T cell clones, %PD-L1 expression and starting tumor diameter at therapy were the significant parameters differentiating responders from non-responders. The reported number of T cell clones falls in line with number of average clones for responders (100) reported by Tumeh *et al*.^43^, although it slightly overpredicts the number of clones on average reported for non-responders (10) for melanoma in the clinic, still within the range. Higher PD-L1 expression correlated with increased response, as shown clinically, and smaller tumors showed better response as well. Lastly, Fig. 2E indicates that PR/CR and stable disease (SD) responders had significantly (about 3–6 times) higher maximum median effector T cell density in the tumor (left), although the density of mAPCs on average over time were lower in responders (right), which corresponded to lower overall tumor burden as the tumor shrank.

### Simulations and predictions of anti-CTLA-4 monotherapy

Prior to utilizing the model to make predictions, we analyzed the simulated response for anti-CTLA-4 therapy (Fig. S1) and compared it to our understanding of response on a qualitative level. Response was similar to that seen in^44^ with a common regimen represented by Ku *et al*., (ClinicalTrials.gov number, NCT00324155)^45^; the boost in peripheral effector T cells was the cause of the response in the model, as also shown clinically^46^. We show a clear and wide dose response between 0.3 and 10 mg/kg (red to green) in Fig. S1A, and for the 3 mg/kg case, simulations show that approximately 20% of patients would be responders (Fig. S1C, left). This would be slightly below or around 20–28.5% ORR seen with CTLA-4 therapy reported for advanced melanoma^47^. From this point on, we utilized the model to make predictions via simulations and compared them to clinical outcomes of published data.

### Simulations and predictions of anti-CTLA-4/anti-PD-1 combination therapy

**Figure 3:**
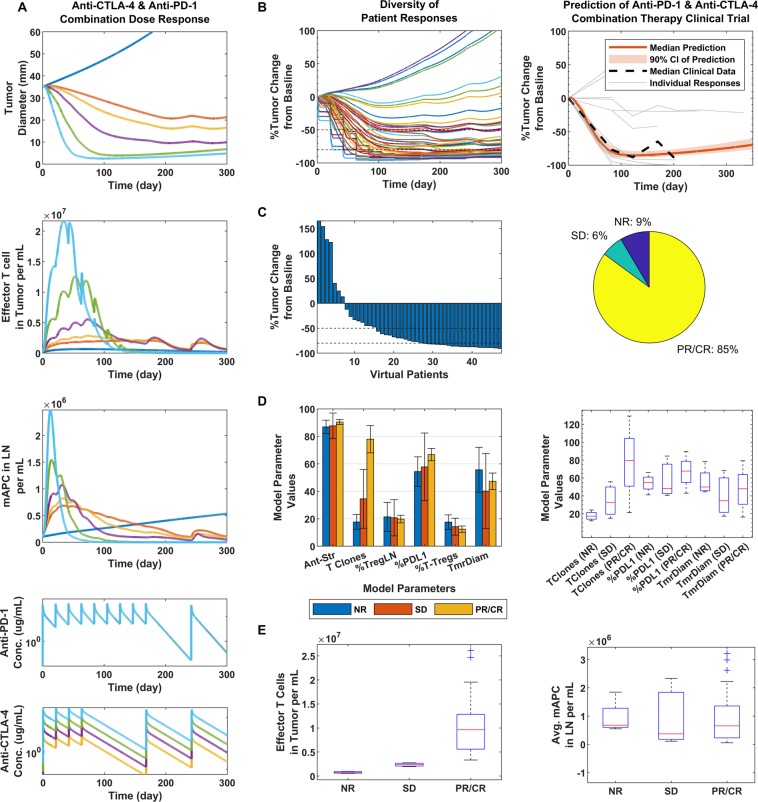
Dose response and clinical validation of anti-PD-1/anti-CTLA-4 combination-therapy. (**A**) From top to bottom: Tumor response to combination therapy at doses of 0.3, 1, 3 and 10 mg/kg of anti-CTLA-4, as represented by the colors in the bottom figure in ascending order and 3 mg/kg for anti-PD-1 was used for all simulations; the blue line indicates no therapy (top figure), and orange indicates only anti-PD-1. Then, effector T cell density in the tumor (second from the top), mAPC density in the lymph nodes (third from the top) and finally, the PK of anti-PD-1 and lastly, anti-CTLA-4 at the given doses. For all following figures, 1 mg/kg anti-PD-1 and 3 mg/kg anti-CTLA-4 were used, following the same regimen. (**B**) Diversity of tumor response (left), prediction of median clinical response data (right). (**C**) Waterfall plot of VPs (left) and pie chart (right) with percent of virtual non-responders (NR), stable disease (SD) and partial or complete responders (PR/CR). (**D**) Bar graph comparison parameters varied in model for each responder type (left) and box plots of significant differentiators (right). (**E**) Max effector T cell density in the tumor (left) and average mAPC density in the lymph nodes (right) for each responder category.

In Fig. 3, we show our predictions for anti-CTLA-4/anti-PD-1 combination therapy, based on the trial regimen as carried out in Wolchok *et al*. (ClinicalTrials.gov number, NCT01024231)^48^, and represented by the PK in Fig. 3A. The trial was simulated using 47 VPs, as done in the clinical trial with 1 mg/kg anti-PD-1 and 3 mg/kg anti-CTLA-4. Figure 3B shows that a higher proportion of VPs can be expected to be PR/CR responders, although some could have progressive disease or SD (left). In Fig. 3B, right, we show that the median of the clinical trial data and the median from our simulated virtual clinical trial overlap to a large extent within the narrow confines of the 90% confidence interval. We further see that the clinical trial also had patients who had stable and progressive disease, although to a much lower extent than those who showed partial or complete responses. Of our VP population, 85% were PR/CR responders (Fig. 3b, right), compared to clinical results showing 79% having robust responses, and with further analysis we indicate that the number of T cell clones and an increase in maximum effector T cell density in the tumor to be the main significant driving factors for response in our virtual population; similar to anti-PD-1 monotherapy case, except starting tumor diameter and %PD-L1 at therapy were less significant and the density of effector T cells in the tumor showed a wider range for responders.

### Simulations and predictions of anti-PD-L1 monotherapy

**Figure 4:**
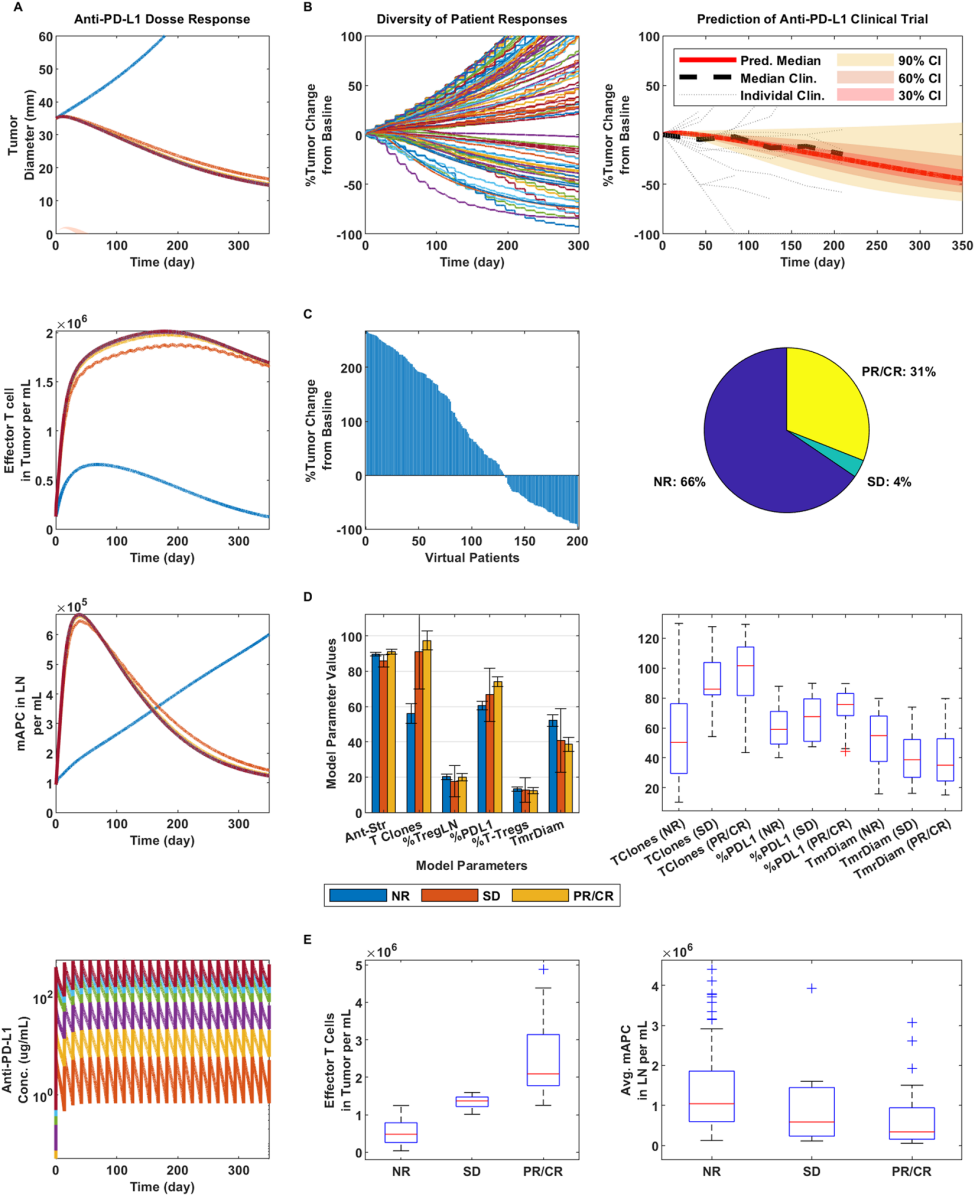
Dose response and clinical validation of anti-PD-L1 monotherapy. (**A**) From top to bottom: Tumor response to anti-PD-L1 therapy at doses of 0.3, 1, 3, 10, 15 and 20 mg/kg as represented by the colors in the bottom figure in ascending order; the blue line indicates no therapy (top figure). Then, effector T cell density in the tumor (second from the top), mAPC density in the lymph nodes (third from the top) and finally, the PK of anti-PD-L1 at the given doses. For all following figures, 20 mg/kg anti-PD-L1 was used, following the same regimen. (**B**) Diversity of tumor response (left), prediction of median clinical response data (right). (**C**) Waterfall plot of VPs (left) and pie chart (right) with percent of virtual non-responders (NR), stable disease (SD) and partial or complete responders (PR/CR). (**D**) Bar graph comparison parameters varied in model for each responder type (left) and box plots of significant differentiators (right). (**E**) Max effector T cell density in the tumor (left) and average mAPC density in the lymph nodes (right) for each responder category.

We further employed the same kind of analysis to anti-PD-L1 monotherapy in comparison to a trial run by Brahmer *et al*., (ClinicalTrials.gov number, NCT00729664)^49^, where 20 mg/kg anti-PD-L1 was administered Q2W. Figure 4A shows that much like for anti-PD-1 therapy, the dose response is largely flat. As shown previously, we demonstrate that a diversity of responses across a VP population of 200 is possible (Fig. 4B, left) and that the simulated median within a 30% confidence interval largely overlaps with the clinically determined median (Fig. 4B, right). We further predict that overall clinical response would be lower for anti-PD-L1 than that shown for anti-PD-1, where 35% (Fig. 4C) showed overall response compared with 43% in our VP population, respectively, which is in line with what was seen qualitatively in the mentioned trials. Interestingly, we determine PD-L1 therapy as lower in efficacy compared to PD-1 therapy for melanoma, which may be a consequence of the associated checkpoints (i.e., CD80, PD-1 and PD-L2, expressed at literature reported averages for melanoma). In fact, PD-L2 expression can help overcome PD-L1 blockade, since the latter cannot prevent the former’s interaction with PD-1; while anti-PD-1 blocks both interactions of PD-1 with PD-L1 and PD-L2 and CD80 is minimally expressed in comparison. The effects of varying CD80, PD-1, PD-L1 and PD-L2 expression levels on response during each therapy are examined in a sensitivity analysis, described later. In a similar manner, as for anti-PD-1 therapy, we find that number of T cell clones, %PD-L1 expression and tumor diameter at therapy are significantly different between non-responders and those showing partial or complete responses (Fig. 4D). We further show the same type of trend for maximum effector T cell density in the tumor and average mAPC density in the lymph nodes as for anti-PD-1 therapy.

### Sequential therapy of anti-CTLA-4 first then anti-PD-1 vs. the reverse for induction

**Figure 5:**
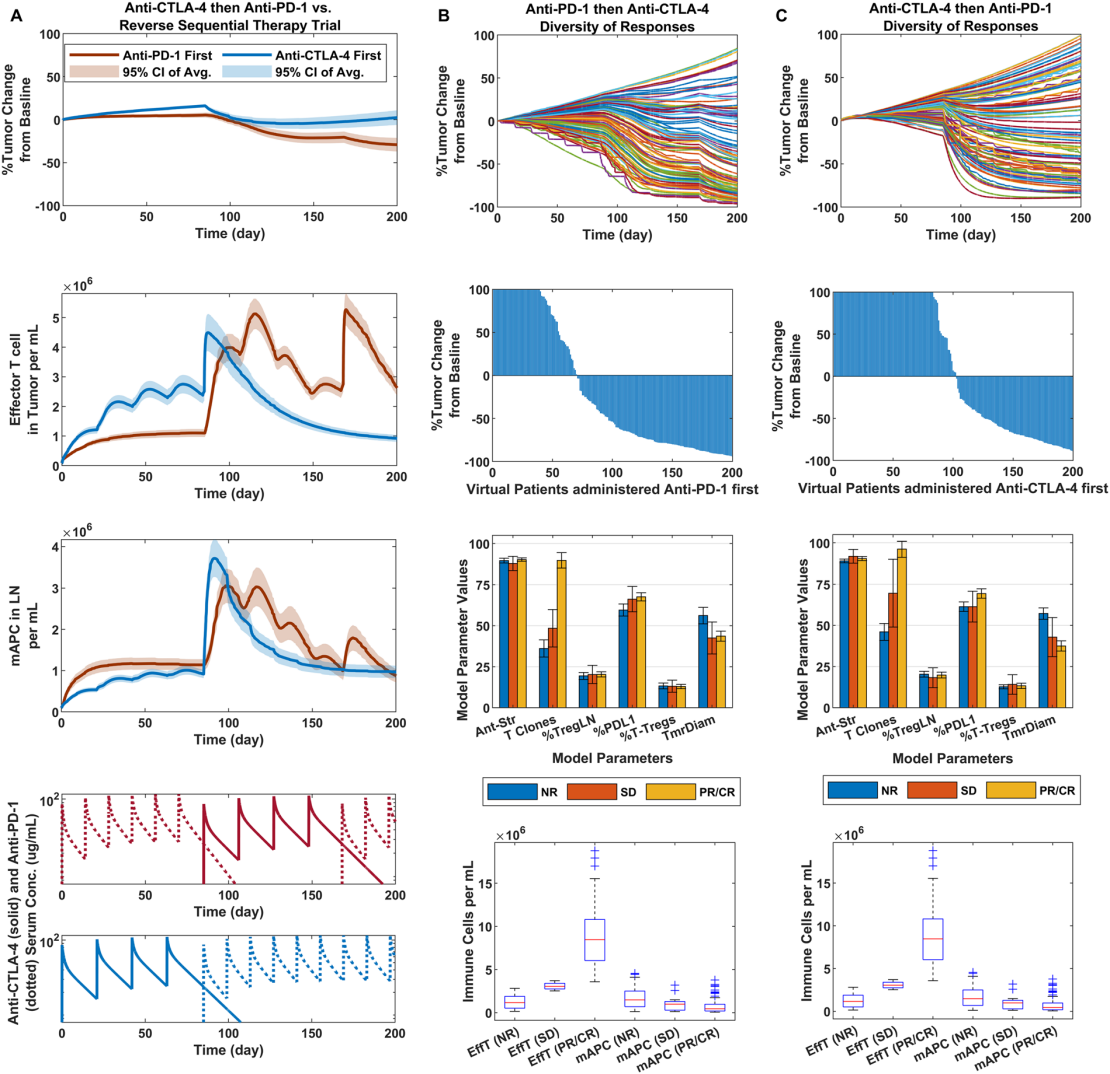
Sequential therapy of anti-PD-1 and anti-CTLA-4. (**A**) From top to bottom: Tumor response to sequential therapy with dosing as listed in phase II CheckMate 064 trial (NCT01783938) with regimens shown in bottom two figures. Following response (top), effector T cell density in the tumor (second from the top), mAPC density in the lymph nodes (third from the top) and finally, the PK of anti-PD-1 first regimen and lastly, anti-CTLA-4 first regimen. (**B**) Anti-PD-1 therapy first: Comparison of variability in responses (top), waterfall plots of responders in virtual trial (second from top), Bar graph comparison parameters varied in model for each responder type (third from top), and boxplots of Max effector T cell density in the tumor and average mAPC density in the lymph nodes (bottom) for each responder category: virtual non-responders (NR), stable disease (SD) and partial or complete responders (CR/PR). (**C**) Same as (**B**), except for anti-CTLA-4 therapy administered first.

Our final comparison to a human clinical trial was performed on a more qualitative basis. We assessed the model’s ability to emulate sequential therapy responses based on the induction therapy trial published by Weber *et al*., (ClinicalTrials.gov number, NCT01783938)^50^. Here, anti-CTLA-4 administered first followed by anti-PD-1 therapy was compared to the reverse sequence (Fig. 5). Overall, we found our results to indicate that on average (and for individual cases) anti-PD-1 administered first followed by anti-CTLA-4 (Fig. 5A, blue) produced a greater effect on tumor response than anti-CTLA-4 first followed by anti-PD-1 (Fig. 5A, red), consistent with clinical results. It was interesting to see these results, even though in both scenarios the ranges and medians of maximum effector T cells and average mAPC densities (Fig. 5B,C, bottom) were about equal. Two significantly differentiating parameters, however, were the number of T cell clones and %PD-L1 expression on the cancer cells (Fig. 5B,C, second from bottom).

### Sensitivity analysis and the role of chemokines and antigen spread in diversity of tumor responses

**Figure 6:**
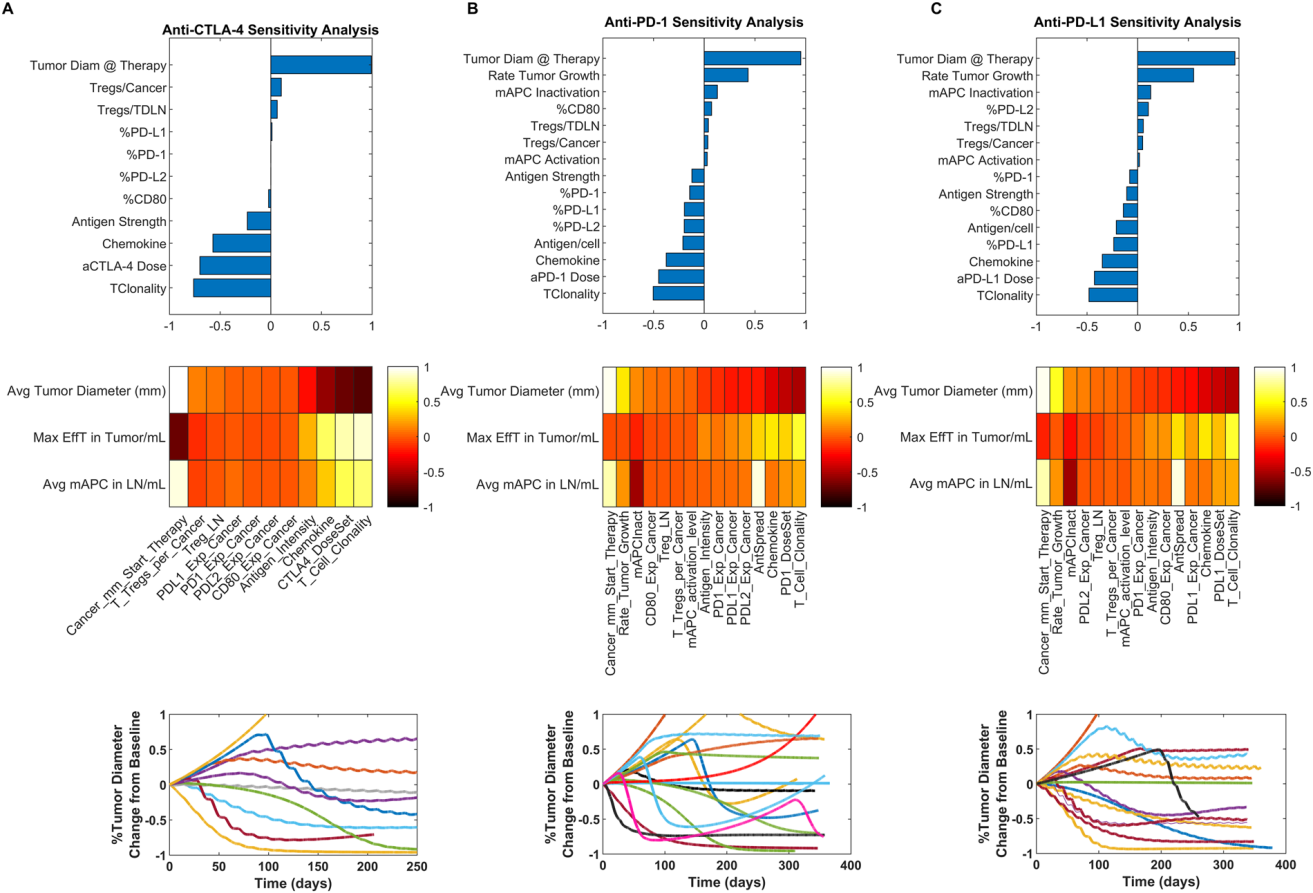
Sensitivity analysis showing (**A**) anti-CTL-4, (**B**) anti-PD-1, and (**C**) anti-PD-L1 sensitivities relative to average tumor diameter (top row); in heatmap form to average tumor diameter, max effector T cell density in tumor and average mAPC density in the lymph nodes (second row); and representations of diversity in types of responses with variation in sensitivity analysis parameters, respectively for each column.

So far, we have shown the responses to be largely monotonic; however, evidence shows that a number of cases during human tumor regression result in nonmonotonic responses, where the tumor can grow for a certain period of time and then all of a sudden rapidly regress, it can be stable and then begin to regress or grow, or the tumor can regress for a period of time and to a certain percentage and then either become stable or begin to grow again. In Fig. 6, we show that these cases are possible to simulate as well without varying any of the parameters during each simulation. We suggest that the cause of these cases may have to do more with antigen processing (and presence) and its combined effect within the tumor microenvironment than with just the physiological parameters represented in the simulations thus far that did not focus on variations in mAPC activation, antigen abundance per cell and tumor chemokine levels, as well as different combination of CD80, PD1, PDL1 and PDL2 expressed on the cancer cells. Sensitivity analyses were run for (A) CTLA-4, (B) PD-1 and (C) PD-L1 monotherapies (using the same regimens presented in previous figures) looking at the mentioned parameters and a few others (represented in Table S9); the relative effects on average tumor diameter, maximum effector T cell densities in the tumor and average mAPC densities in the lymph nodes are shown via the heatmap. In the last row of the figure, we show the diversity of responses that can be achieved with each type of therapy, given the variation in the listed parameters. We show the p-values of the sensitivity analyses via heat maps in Fig. S6.

## Discussion

Here we developed an immuno-oncology QSP model representing the biophysics of the system of a generic VP with each simulation defined by a set of parameter values. We demonstrated that it can capture several immunotherapy-related responses seen clinically, such as delayed responses (over several weeks to months), non-monotonic responses and a diversity of patient responses from non-responders to complete and partial responders. We also showed that multiple value sets of parameters in the virtual clinical trials were able to produce the same or similar types of responses, however there were certain parameters that could produce unique types of responders within specific ranges. These parameters were percent checkpoint expression levels, tumor diameter at the start of therapy and the number of T cell clones and were captured statistically in the presented figures.

The key advantage we see for this model is that it encompasses an immune response against a growing tumor at its core and is set up in such a manner that additional processes can be added without anticipation of having to fully reparameterize the entire model to make clinical predictions. In addition to the validation and simulations presented above, we further made predictions on therapy combination for which human clinical data were not available in the literature. This is shown in Fig. S2 (for CTLA-4/PD-L1 combination therapy for melanoma) and in Fig. S3 (for PD-1/PD-L1 combination therapy). Interestingly, for these, we predict a higher ORR for PD-1/PD-L1 than for CTLA-4/PD-1 despite a similar expected median at 1-year post-therapy. This may be due to checkpoint expression levels and shows that the model captures ORR and median responses as two separate but linked readouts. While we believe the model to be valid for the stated purpose, we also realize its potential in explaining seemingly contradictory results, as well as its limitations. We begin by with an attempt at explaining the surprising results of Fig. 5 (the sequential therapy trial) that we assessed qualitatively^51^.

The response lines in Fig. 5 were a representation of all the response dynamics averaged and the 90% confidence intervals represented. Effector T cell density was greater with anti-CTLA-4 therapy first due to the enhanced activation and proliferation and when anti-PD-1 was administered following it, the cells already present in the tumor were able to effectively kill the cancer cell. In the reverse case, when the switch from PD-1 therapy to CTLA-4 therapy occurred, new effector cells had to be generated to catch up with the density present when CTLA-4 therapy was administered first; however, since PD-1 therapy proved to be more potent initially in reducing the tumor size and therefore, the number of antigens (tumor burden) that is required for priming, CTLA-4 therapy promoted a greater enhancement of response from less tumor burden than was feasible with PD-1 therapy at first. Furthermore, since PD-1 therapy has less of a dose response, adding CTLA-4 therapy allowed for the full effect of the latter to contribute to a combination therapy response.

Another interesting aspect captured by this model is the effects of different checkpoint receptor expression levels on cancer cells and how this affected response to different therapies; shown in Fig. 6. We previously mentioned that PD-L1 therapy may have less efficacy than PD-1 therapy in melanoma as a result of the checkpoint expression levels. What this could further mean is that different types of tumors can have different expression levels of immune checkpoint receptors, and the way they respond to different therapies can be dependent upon the exact combinations of receptor expression levels. Thus, this model may help assist with choice of therapies and regimens for specific indications.

Lastly, despite the positive aspects of this model, several key questions were not addressed. First, we did not assess if the effector T cells that promote cytotoxic activity against the tumor replicate within the tumor; instead, we assumed that the lymph nodes were the sole producers of response. We also did not address synergy vs. additivity, how the Tregs and MDSCs function to inhibit the effector T cells and mAPCs, outside of the immune checkpoints, and how the TME changes with time, along with the seemingly important added effects from the helper T cells and other cell types, such as the NK cells, or considering the inhibitory effects of tumor associated macrophages and fibroblasts. These are additional to the assumptions defined by the models we incorporated into ours that were published by other groups referenced here. It is also important to state that our VP population is defined solely within quantitatively (or qualitatively) defined literature reported ranges and understandings, which may not reflect the true nature of the patients enrolled in each trial, as well as how the parameters may have changed for the patients as the trials proceeded. This data, which we did not have, would need to be collected for each patient over time in each trial to update and refine the model more specifically.

Interestingly, despite not having answers to these questions, we were still able to verify our model with good correlation to clinical data from several different clinical trials without having to change the parameter values in the model between runs or during the simulations. In fact, we were able to emulate several mentioned clinical trials utilizing a range of different regimens of mono-, combo- and sequential therapies targeting CTLA-4, PD-1 and PD-L1; we were able to show the utility of the model in predicting different therapeutic outcomes at different dose levels. We were able to show how different literature reported parameter values (such as T cell clonality and %PD-L1 expression on cancer cells) held true in producing different types of responders given the same therapeutic regimens. We were able to determine how the model can be further used to define an indication for a certain type of therapeutic regimen, and which patient populations may be best served given their parameter profiles, which may change dynamically. In addition, while this model was parameterized and validated for metastatic melanoma in humans, we believe that given its structure, it can also be reparameterized and validated for other types of cancers, as has been done for triple-negative breast cancer^52^ and, a modified model, for non-small cell lung cancer^53^. Lastly, this model may then assist with optimizing dosing regimens and efficacious dose selection, which would make it translationally applicable from the preclinical through the clinical phases of drug development.

## Methods

### Model structure and dynamics

We have developed a multiscale PB-QSP model for human melanoma in order to predict how the innate state of the immune system and the tumor microenvironment in a patient at any given time play a role in determining the efficacies of cancer immunotherapies in a dynamical fashion. The model was developed using a bottom-up approach where first the cellular and molecular species interactions were defined using biochemical reaction equations as the framework of the model and physiologically-derived parameters were utilized as much as possible. The focus was as follows: (1) tumor draining lymph node priming and activation of naïve T cells into effector cells in the presence of mature antigen presenting cells (mAPCs) and Tregs, (2) the subsequent migration of the effector cells to the tumor through systemic circulation, (3) interaction with a growing tumor that includes Tregs and MDSCs, in addition to cancer cells, (4) the release of TAA/TSA by the cancer cells by natural death and killing, (5) the uptake of the TAA/TSA by APCs, their maturation into mAPCs and subsequent migration back to the draining lymph node compartment for presentation to naïve effector T cells for their priming and activation, as the cycle starts again. Additionally, checkpoint receptors were defined to be expressed by the cells at points in specific processes, as defined in the literature, that influence the immune response at the respective points. Checkpoint inhibitor therapies in the model were used to block the effects of the receptor interactions and the resulting consequence (i.e., reduction in inhibitory signaling) was largely driven by the binding kinetics of the antibody to the respective receptors, as explained below.

The immunogenicity in the model was represented as explained in the introduction and described further here. It encompasses the number of T cell clones, the antigen strength and the abundance of antigens released by each cancer cell upon its death in a continuous manner, and each of the mentioned aspects were represented by a given parameter (total of 3 parameters) at the appropriate places in the model. Consequently, the total tumor burden in the model was dependent upon tumor size; with larger tumors having more cancer cells that would, at least, decay naturally and release more antigens in total at any given time; however, they would also naturally put up more resistance and present a larger growing mass for the immune system to compete against. The overall tumor response was a result of the previously mentioned factors: (1) Immunogenicity of the tumor and degree of formation of an immune response against it, (2) the suppressive mechanisms by the immune checkpoints and tumor microenvironment during tumor growth, and (3) the release of the suppressive immune checkpoints by antibody blockade against, CTLA-4, PD-1 and PD-L1 administered as monotherapies, combination therapies, or sequential therapies. Each antibody therapy regimen was examined on multiple levels, and in specific cases tumor response was compared to that reported in published human clinical trials for the given compounds.

In order to simulate virtual clinical trials, parameters defining immunogenicity and tumor resistance were varied within known physiological ranges (see Table S9), with each model run and output representing a single VP result. For immunogenicity: The antigen strength (represented on a scale from 0.1–1.0, from least to greatest) is known to be high for melanoma, and thus in the model this value is set between 0.8 and 1.0 and effects the priming and activation of T cells^5^. The number of T cell clones for melanoma was defined between 10–130, representative of what was found in a melanoma clinical trial^43^. Antigen abundance per cancer cell was set to 100 based on fitting to anti-PD-1 clinical data for each of the responder types, as described. For tumor resistance: percent of cancer cells expressing PD-L1 was varied from 40–90%, lymph node T-regulatory cells (Tregs) were varied from 5–35% of total lymph node T cells and tumor Tregs were varied from 0–0.25% of total cancer cells, while we kept three times as many Myeloid-Derived Suppressor Cells (MDSCs) as Tregs in the tumor at all times (see Supplement Tables for parameter references). Finally, the tumor size was varied from 15–80 mm in diameter (along the longest diameter) and the tumor was assumed ellipsoid, as defined in the methods.

### Priming and activation of effector T cells

The model consists of several compartments for effector T cell trafficking once they exit the TDLN following priming and activation, as described previously by Zhu *et al*. Here, we built the model encompassing the following trafficking compartments: lymph node, blood, tumor, lung, GI tract, spleen, and liver, while the remainder of the organs were lumped into the peripheral compartment^38^. For each organ, the vascular and interstitial volumes are represented, as well as the blood and lymph flow into and out of the organ. As in the physiologically-based model, effector cells can bind reversibly to the vasculature in the space of the lung, liver, spleen and tumor, attach irreversibly, becoming arrested and extravasate into the interstitial space from where they can circulate through the lymph and back into peripheral circulation. In the tumor, however, once the effector cells enter the interstitial space, they stay there to interact with the tumor cells. Elimination is set to occur in the lungs, as described in the mentioned study, and in the tumor, based on interactions with cancer and supporting cells. The antibodies are added to the central (blood) compartment and follow a minimal-PBPK layout. The cellular dynamics in the model are the prime elements that define the outcomes of the immunotherapy regimens, while the molecular dynamics are the defining factors for the cellular dynamics. Overall, the model consists of 282 ordinary differential equations (ODEs) and 218 algebraic equations and is implemented using the SimBiology plugin in MATLAB (MathWorks, Natick, MA). To ensure reproducibility of the model, the complete set of governing equations and parameters are presented in the supplement and the SBML code is provided.

In the TDLN compartment, the immune response is generated through the activation of tumor-targeting effector T cells resulting from the presentation of antigens by mature antigen presenting cells (mAPCs). Activated effector T cells migrate out of the TDLN and are trafficked as described above. In total, the priming process has been shown by intravital microscopy to occur in three distinct stages in the LN, which are incorporated in the model^54^. The possibility of a successful priming interaction between mAPCs and T cells is represented by an algebraic equation that was extrapolated from an agent-based model, which was coupled with experiments to calculate the probability of at least one successful priming interaction to occur over the designated time^55^. The final equation and an example of how it is used is shown in Eqs 1.1 and 1.2

1.1 $$\frac{d[NT1]}{dt}={k}_{Assoc,P1}[NT](P1)(SigCD28)$$

12 $$P1=1-\exp (\frac{-\,4\pi ({D}_{T})(Dif{f}_{T})(mAP{C}_{nInt})[T]({\varphi }_{NT})(Antigen)}{(TClonality)({S}_{NT\_mAPC})}{t}_{max})$$

Equation 1.1 shows that the association of Naïve T cells (*NT*) with non-interacting mAPC (*mAPC*_*nInt*_) during the first priming stage to become a single state variable (*NT*1) representing the associated state, is determined by an association rate (*k*_*Assoc,P*1_ defined by measured literature values), the number of NT undergoing the interaction, the strength of the priming co-receptor (where *SigCD*28 represents the CD80/86 interaction with CD28 on mAPCs based on receptor occupancy of 0–1.0), and the probability of a successful priming interaction (*P*1). In Eq. 1.2, *P*1 considers the number of mAPCs available to associate with the NT cells (*mAPC*_*nInt*_), as well as other factors that define a value function representing the possibility of a successful priming interaction to occur. These include: The number of NT cells available for priming (calculated from the total number of T cells in the TDLN, *T*, and the fraction which are available for priming (*ϕ*_*NT*_)), their migration or diffusion rate in the TDLN (*Diff*_*T*_), the T cell clonality (*TClonality*), the strength of the antigen landscape during presentation (*Antigen*), a scaling factor for the possibility of a successful priming interaction between an mAPC and an NT cell (*S*_*NT*_*mAPC*_), the geometry of the TDLN, the diameter of each T cell (*D*_*T*_) and the total time over which priming occurs (*t*_*max*_). The number of mAPCs available for priming at any given time is algebraically calculated. Other equations during priming govern the dissociation of mAPCs and NT during the first priming stage and the association of primed NT with free mAPCs for the second priming stage; the equations are listed in the supplement. Thus, the more mAPCs associate with each state (up to the maximum possible) and the stronger the antigen is, the more cells mature to the next stages and ultimately the higher the number of activated effector T cells are generated; this is the mAPC dose response of the T cells during priming^56^.

A successful priming interaction is one that leads to maturation of the NT cells, whereas an unsuccessful one has been shown to lead to a relatively quick dissociation between the NT and mAPCs without the maturation of the NT cells^57^. Once both stages of priming have occurred in a similar manner, the proliferation parameters were optimized to represent division of the cells three times a day over about five days at the maximum levels to produce effector T cells. When disregarding inhibitory checkpoint signals in total, the priming stage takes about a week as Naïve T cells mature to become active effector T cells^54,58^.

Once primed and activated, effector T cells leave the lymph nodes via the efferent lymphatics and make their way into the blood, from which they access the tumor and other tissues^59^. In a human body, there are about 500–600 lymph nodes, but since we consider a tumor localized to a particular region of the body, we assume that only a fraction of total lymph nodes are involved in producing an anti-tumor immune response^60,61^. In total, we approximated 20 distinct regions that have clusters of lymph nodes in the human body, and therefore we estimated that about 30–35 TDLN are involved in the anti-tumor response in a particular given region^62^. Since one TDLN is considered in the compartment in the model, in order to simulate multiple TDLN, the number of effector T cells generated in the TDLN compartment that enter the blood (or the central compartment) are multiplied by the number of TDLN considered in the model. In this manner, we assume that each TDLN functions in the same manner. Furthermore, we assume that each TDLN receives the same number of mAPCs and TAA that are transported from the tumor. Once in the blood, a chemokine factor further promotes effector T cell infiltration into the tumor^63^. In fact, immunotherapies to enhance lymphocyte-recruiting chemokine production by the tumor can also be explored in this model^64^. Furthermore, there is evidence that tumor lymphangiogenesis promotes T cell infiltration, which can be factored into the simulations through the chemokine factor^65^.

### Interactions between effector and target cells, and elimination of cancer cells

The immune response is initiated from the tumor. While in the tumor compartment, the effector T cells interact with and kill the cancer cells through cytotoxic activity, and the dead cancer cells release TAA. In general, all effector-target interactions in the model are represented by a first interaction step (where two cell types become represented by one state variable), and subsequently the effect the interaction has on the target and effector cells over the course of the dissociation step. The interaction steps in each case are defined by a validated model for effector cell killing of cancer cells^66^, and the rate of the subsequent step is defined by the measured rates of interaction between the different cell types from published literature referenced in the supplement. In Eq. 1.3, *E* refers to the effector cells producing the response, while *T* refers to the target cells. Algebraic equations govern the accounting of the cells during this process in each case, and the effect on each cell type following interaction is dependent upon the immune checkpoint signaling. *d* is the rate of association for killing per day, *λ* is the exponent of fractional cell association for killing and *s* is the steepness coefficient

1.3 $$\frac{dT}{dt}=d\frac{{(\frac{E}{T})}^{\lambda }}{s+{(\frac{E}{T})}^{\lambda }}T$$

For cancer cells specifically, their death produces the release of TAA to a defined level per cell, as described previously. In parallel, cancer cells that naturally decay during tumor growth also release TAA. Monocytes migrate into the tumor, differentiate into APCs, which pick up the TAA and become mAPCs. The mAPCs then migrate back to the TDLN via the lymphatic vessels for presentation of the TAA to Naïve and Primed Naïve T cells, during priming for effector T cell activation, and the process begins again. Additionally, the TAA that are not picked up in the tumor by APCs can flow back to the TDLN through the lymphatic vessels, where they are picked up by resident APCs, which would subsequently be involved in the priming process, leading to effector T cell activation. Altogether, this process is a feedforward loop where continuous amplification of effector T cells occurs, and the cycle continues until there are no more cancer cells left to release enough TAA that is required for priming.

### Immune checkpoint signaling and inhibitory effects

The presented scenario considers the rate of natural turnover of the different immune and cancer cell types (i.e. effector T cells, mAPCs and cancer cells) in the human body. In the model, we specifically focus on the molecular species that are either essential for our analysis of the immune response (e.g. CD28:CD80/CD86 binding, or the overall strength of the antigen landscape being presented during priming), or are the direct or indirect targets of the therapies we are testing in the model (e.g. the cognate receptors of CTLA-4, PD-1, and PD-L1 immune checkpoints).

The regulatory immune cells include the Tregs and MDSCs, which are present in the TME along with the cancer cells; Tregs are also present in the TDLN. These cells reside in their respective compartments and do not migrate from one compartment to another like the effector T cells do. Furthermore, their numbers are a function of the other cell types, which change with time within their respective compartments: In the TDLN, Tregs are defined as a certain percentage of the total number of T cell as determined experimentally by flow cytometry (5–35%), while in the tumor Treg are defined as a percentage (0–0.25%) of the number of cancer cells, and the number of MDSCs are two to four times the number of Tregs, based on literature evidence^67–69^. Note that all numerical values of the parameters here and elsewhere in the paper are baseline parameters and can be varied; these values are not the principal part of the model. Overall, these regulatory cells are known to play a role in inhibiting the anti-tumor immune response, and they carry out their activities through various molecular mechanisms, including inhibitory checkpoint receptors and other factors that target effector T cells. Cancer cells also employ inhibitory checkpoint receptors and other mechanisms as a means of protecting themselves, which slow down or inhibit effector T cell activities. The model incorporates checkpoint receptors expressed on the surface of cells and interactions between cells within the volume of the immunological synapses that are formed during cell-cell interactions^70^.

In addition, mAPCs also exhibit a variety of mechanisms that regulate the activation of effector T cells, which can stymie or boost an effective anti-tumor immune response. These mechanisms include the types and origins of the antigens that they present, the ratios of mAPC-to-Naïve and Primed Naive T cells during priming (where, generally, the greater the antigen abundance, the higher the number of mAPCs), the presence of positive immune checkpoints (including co-receptors) and (like the regulatory cells) inhibitory immune checkpoints. During antigen presentation, the B7 co-receptors (CD80 and CD86) on the mAPCs must engage CD28 on the Naïve and Primed T cells, in addition to antigen presentation, for priming to occur. Additionally, a stronger recognized antigen and a higher ratio of mAPC-to-T cells results in a greater extent of priming; i.e., a larger number of activated effector T cells are produced during priming as a result of greater activation and proliferation. Lastly, the inhibitory immune checkpoints expressed on the mAPCs, which are recognized by the cognate checkpoints on the T cells, act to limit the extent of priming and ultimately limit the number of activated effector T cells generated in the model. The specific cell types and the corresponding immune checkpoints in the model are depicted in Fig. 1B. All of the interactions between the ligands and their cognate receptors that are represented in the figure take into account experimentally determined K_d_ values for their binding, receptor expression values measured by flow cytometry (or their approximate expression levels) and the volumes in which they interact (being either within the immunological synapses, or within the entire volume of the designated compartment where the interaction is occurring). Note that in the model, we do not account for the mechanisms of antigen processing and we do not know the variety of antigens present and their binding affinity values; therefore, we use a general antigen strength value from 0.1–1.0, which for melanoma is known to be on the higher end and is so set between 0.8 and 1.0 for all simulations and fitting, as mentioned.

### Representations of tumor heterogeneity

While tumor heterogeneity is not represented on a spatial scale in this version of the model, it is represented in terms of checkpoint heterogeneity. The expression of the different checkpoints on different cancer cells within a tumor can be heterogeneously distributed, but more importantly in this analysis is the percent of cancer cells that express particular checkpoints. Considering that a cancer cell expresses a certain immune checkpoint, it would affect an effector T cell differently than if the cancer cell were to express two different immune checkpoints to the same extents. The problem translates to the fact that if we state that 60% of the cancer cells express PD-L1 for one tumor, for example, while 5% express PD-1 from an independent tumor study, we do not know if 5% of the cancer cells should express both immune checkpoints in our model (which represents a single unified tumor), or if less than 5% express both checkpoints; in other words, PD-L1 and PD-1 can be expressed on the same or distinct cancer cells and their expressions can overlap to varying extents across the tumor, which would affect the lymphocytes differently depending on which cells they interact with. Although the model is considered “well-mixed” in general, unlike spatially-resolved agent-based models^71^, local interactions are accounted for distinctly by separate compartments and submodules.

Therefore, in this model, the percent expression of each immune checkpoint is an input, and for the presented simulations we assumed that the immune checkpoints are equally and independently distributed across the tumor; this assumption can be easily relaxed. For example, if we consider checkpoints A and B, at 50% expression each across the tumor, then 25% would express A and B, 25% would express only A, 25% would express only B, and 25% would express neither A nor B. This distribution can be modified to fit whatever the experimental data determine is the correct distribution for the tumor; however, at this stage we have not seen any consensus on how the percentages of immune checkpoints across the tumor are distributed across the different cancer cells, which resulted in the stated assumption for modeling purposes. In the model, while the number of immune checkpoints considered can be expanded, we consider four different immune checkpoints that are expressed on the cancer cells that have overlapping interactions with the targets of our therapies, PD-1, PD-L1, PD-L2 and CD80, which translates to 2^n^ different checkpoint expression combinations across the tumor (where n = 4, the number of different checkpoints considered). This in turn translates to 16 unique cancer cell-effector T cell signaling interactions, assuming all effector T cells have the same expression of immune checkpoints.

The advantage of structuring the model in such a manner is that we can translate percentage of different checkpoints within the tumor that are found individually on cells into a unified single tumor model, and in the future, as more checkpoint expression data are captured from a single tumor regarding the percentage of checkpoint expressions relative to one another, they can be entered in the model. Thus, the distribution of immune checkpoints on the cancer cells in the model can be modified if need be. Furthermore, it is useful to note that in the current model and for the following analyses we assume that no particular cancer cell type which has a certain sequence of immune checkpoints expressed on is ever fully removed from the system in terms of the percent it is present in the tumor as a whole; instead, as cancer cells get killed in the model, the tumor as a whole retains the same constant heterogeneous checkpoint distribution, fraction-wise. In the future, it would be interesting to compare the results obtained here with those when certain cancer cells with specific checkpoint expressions are able to be eradicated first or with some stochastic variability in checkpoint expression based on certain criteria.

### Pharmacokinetics of immune checkpoint antibodies

Antibodies are delivered to the central compartment (blood) as a zero-order intravenous infusion over the course of one or one and a half hours, as prescribed for each antibody. The antibodies are also cleared from the central compartment through a first order elimination. The distribution of the antibody was designed using a minimal PBPK model^39^ between the central, tight and leaky tissues and lymph node compartments. Distribution between the central and tumor, and between the tumor and lymph node compartments are calculated by considering the surface area of blood or lymph vessels in the tumor volume and permeability of antibodies across that surface area, as represented in Finley *et al*.^72^; and the PK tumor compartment for the PK volume was varied with the true tumor size in the model, given this approach.

For the estimated PK parameters of all the antibodies used in this model, the concentrations of the antibodies in the central compartment were fit to available mean patient population serum concentrations taken over time, displayed in Fig. S4. For anti-PD-1, the data came from Nivolumab, for anti-PD-L1 it came from Durvalumab, and for anti-CTLA-4, it came from Ipilimumab published sources listed in the supplement. Fitting of each therapy to 3 mg/kg single dose administration is shown in Fig. S4, along with the PBPK outputs for the other tissues, including the tumor, which was found to have a concentration about 15–20% of the serum concentration within a few days, and having an increased ratio with time due to an enhanced permeability and retention (EPR) effect^73^. The concentration in the lymph was about half of the tumor concentration, or less. While target-mediated drug disposition (TMDD) was only represented for CTLA-4, there is literature evidence that TMDD for anti-PD-1 does not occur^74^ and we assumed that to be the case for anti-PD-L1 as well; although it could be easily incorporated should the need arises. The free fraction is reported as the concentration in each compartment. Once reaching their sites of activity in the TDLN or tumor, each antibody would bind to its respective target, which would influence the pharmacodynamics (PD) of the immune response and the tumor.

### Immune checkpoint signal modeling

Multiple immune checkpoints were considered to have effects on cells at specific points. The combined effect of the signaling was calculated as follows in Eq. 1.4

1.4 $${f}_{a}=\frac{{[{\sum }_{i=1}^{n}{w}_{i}{(\frac{RO}{R{O}_{max}})}_{i}]}^{m}}{1+{[{\sum }_{i=1}^{n}{w}_{i}{(\frac{RO}{R{O}_{max}})}_{i}]}^{m}}$$

1.5 $${w}_{i}=\frac{{(R{O}_{max})}_{i}}{{\sum }_{i=1}^{n}{(R{O}_{max})}_{i}}$$

This equation was based on the generalized equations derived by Chou and Talalay to determine summation of effects (*f*_*a*_) of two or more mutually exclusive drugs^75^. In order to avoid having to calculate the potency of each checkpoint interaction, *D* in the original equation was replaced by receptor occupancy (*RO*) and instead of *D50*, the maximum RO possible during signaling for each respective receptor was used (*RO*_*max*_). Lastly the weights of receptor signaling (*w*_*i*_) were assumed to take on the fraction of total receptor occupancy for all the receptors, as shown in Eq. 1.5; this turned out to be a consequence of the receptor expression levels, their binding affinities to the respective antibodies, any competing binding interactions and the concentration (or exposure) of the antibodies in the environment. For all signaling, the Hill-coefficient (*m*) was greater than 1 to represent the higher-order system, which denotes the sigmoidicity of the dose-effect curve. The same values for all the variables were used consistently throughout the model, with exception of the *RO* related values, which were calculated dynamically based upon receptor/ligand interactions.

### Parameter optimization

The Multistart function along with lsqnonlin were run using the parallel computing toolbox in MATLAB to perform global optimization on single or multiple parameters in the model in a constrained manner. This allowed for multiple local minima to be found by lsqnonlin for each parameter by starting at a defined number of randomly dispersed initial values within the constrained search range. Then, the local minima that were found were compared to one another and the approximate global minimum was found.

### Simulation settings

Since this study focuses on the several specific melanoma clinical trials with CTLA-4, PD-1 and PD-L1 therapies administered individually, in combination and sequentially, the model was parameterized for melanoma. The model reactions and rate laws are listed in Tables S1 and S2, with the descriptions in Table S3. Table S4 lists the species (or state variables) in the model, as well as the units for each and their descriptions. Lastly, the model values, units and ranges of parameters with the references are listed in Table S5 with a description for each. Table S6 contains the algebraic equations, Table S7 contains the event functions for antibody administration regimens and Table S8 lists all the compartment volumes in the model. Altogether, they provide the complete governing equations, model parameters, and associated information so that the model can be adequately reproduced; the SBML code is also included to simplify its implementation.

For virtual clinical trials, parameter ranges were as follows (all listed in Tables S5 and S9 as well with corresponding references): Tumor diameter at start of therapy (15–80 mm), Antigen strength (0.8–1.0), T cell clonality (10–130), Tregs in the TDLN (5–35% of total T cells in the TDLN), Tregs in the tumor (0–0.25% of total cancer cells), MDSCs (3 times the number of T-regs in the tumor) and %PD-L1 expression (40–90%, assuming an approximate cut-off of 50% for responders). Table S11 lists the values specific to each clinical trial simulated. For sensitivity analysis, the following ranges were used (listed in Tables S5 and S10 as well): Same ranges as mentioned above, except percent checkpoint expressions levels for PD-L1, PD-1, PD-L2 and CD80 ranged from 1–100%, Antigen abundance per cancer cell (10–100,000), T cell clonality (5–130), mAPC inactivation in TME (5–95%), chemokine factor (50–500), mAPC activation in the TDLN (0.1–1.0) and tumor diameter at start of therapy (5–80 mm) to sample a wider range of conditions.

All steady-state and dynamic solutions were calculated using the Sundials solver with the absolute tolerance and relative tolerance set to be 10^−9^ days and 10^−8^, respectively. Simulations were performed by setting a tumor diameter that is 95% of the tumor diameter at which each therapeutic regimen will begin to be administered; this was determined to be enough for the system to reach quasi-steady state prior to administration of therapy. The figures show the response only from the start of therapy, and not the tumor, immune or PK dynamics prior to it.

Tumor growth is simulated for approximately 1 year after therapy begins.

## Supplementary information

Supplementary Info
