# Supplementary material for: A QSP Model for Predicting Clinical Responses to Monotherapy, Combination and Sequential Therapy Following CTLA-4, PD-1, and PD-L1 Checkpoint Blockade
Source: Sci Rep. 2019 Aug 2;9:11286. doi: 10.1038/s41598-019-47802-4 (PMC6677731; doi:10.1038/s41598-019-47802-4)
Supplement: Supplementary file 1 — Supplementary Info [file 41598_2019_47802_MOESM1_ESM.pdf]

A QSP Model for Predicting Clinical Responses to Monotherapy, Combination and Sequential Therapy Following CTLA-4, PD-1, and PD-L1 Checkpoint Blockade

Oleg Milberg*[1], Chang Gong[1], Mohammad Jafarnejad[1], Imke H. Bartelink[2,5], Bing Wang[2], Paolo Vicini[3], Rajesh Narwal[4], Lorin Roskos[4], and Aleksander S. Popel[1,6]

[1]Department of Biomedical Engineering, Johns Hopkins University School of Medicine, Baltimore, Maryland

[2] Clinical Pharmacology, Pharmacometrics and DMPK (CPD), MedImmune, South San Francisco, California, USA

[3] Clinical Pharmacology, Pharmacometrics and DMPK, MedImmune, Cambridge, United Kingdom;

[4] MedImmune, Gaithersburg, Maryland, USA

[5] Department of Clinical Pharmacology and Pharmacy, Amsterdam UMC, Vrije Universiteit Amsterdam, The Netherlands

[6] The Sidney Kimmel Comprehensive Cancer Center, Johns Hopkins University School of Medicine, Baltimore, Maryland

Address for Correspondence:

Oleg Milberg, Ph.D.

Department of Biomedical Engineering

School of Medicine

Johns Hopkins University

720 Rutland Avenue

Baltimore, MD 21205

Telephone: 301-651-4868

Email: oleg.milberg@gmail.com

Keywords: Immuno-oncology, immunotherapy, melanoma, systems biology, computational model, T cell, quantitative systems pharmacology (QSP), pharmacokinetics (PK), pharmacodynamics (PD), checkpoint inhibitor therapies, virtual clinical trials, virtual patients, combination therapy, sequential therapy, multiscale modeling

Table S1 – Model Reactions (Start)

Reaction Number	Reaction
**1**	[Blood-Lymph].CTLA4_mabB <-> Lymph_Node.CTLA4_mab
**2**	[Blood-Lymph].CTLA4_mabB <-> Peripheral.CTLA4_mabP_leaky
**3**	[Blood-Lymph].CTLA4_mabB <-> Peripheral.CTLA4_mabP_tight
**4**	[Blood-Lymph].CTLA4_mabB <-> Tumor.CTLA4_mabt
**5**	[Blood-Lymph].CTLA4_mabB -> null
**6**	[Blood-Lymph].Effector_T_TB -> [Blood-Lymph].Effector_T_TB + [Blood-Lymph].Effector_TB
**7**	[Blood-Lymph].Effector_TB -> Liv_Spln_GI.EffT_GI_f_Vasc
**8**	[Blood-Lymph].Effector_TB -> Liv_Spln_GI.EffT_Liver_f_Vasc
**9**	[Blood-Lymph].Effector_TB -> Liv_Spln_GI.EffT_Spleen_f_Vasc
**10**	[Blood-Lymph].Effector_TB -> Lymph_Node.EffT_LN_f_Vasc
**11**	[Blood-Lymph].Effector_TB -> null
**12**	[Blood-Lymph].Effector_TB -> Peripheral.EffT_P_f_Vasc
**13**	[Blood-Lymph].Effector_TB -> Tumor.EffT_f_Vasc
**14**	[Blood-Lymph].PD1_mabb <-> Lymph_Node.PD1_mab
**15**	[Blood-Lymph].PD1_mabb <-> Peripheral.PD1_mabP_leaky
**16**	[Blood-Lymph].PD1_mabb <-> Peripheral.PD1_mabP_tight
**17**	[Blood-Lymph].PD1_mabb <-> Tumor.PD1_mabt
**18**	[Blood-Lymph].PD1_mabb -> null
**19**	[Blood-Lymph].PDL1_mabb <-> Lymph_Node.PDL1_mab
**20**	[Blood-Lymph].PDL1_mabb <-> Peripheral.PDL1_mabP_leaky
**21**	[Blood-Lymph].PDL1_mabb <-> Peripheral.PDL1_mabP_tight
**22**	[Blood-Lymph].PDL1_mabb <-> Tumor.PDL1_mabt
**23**	[Blood-Lymph].PDL1_mabb -> null
**24**	[Blood-Lymph].PDL1_mabb -> null
**25**	Liv_Spln_GI.EffT_GI_f_Vasc -> Liv_Spln_GI.EffT_Liver_f_Vasc
**26**	Liv_Spln_GI.EffT_GI_f_Vasc -> null
**27**	Liv_Spln_GI.EffT_Liver_AR_Vasc -> Liv_Spln_GI.EffT_LiverEx
**28**	Liv_Spln_GI.EffT_Liver_AR_Vasc -> null
**29**	Liv_Spln_GI.EffT_Liver_b_Vasc -> Liv_Spln_GI.EffT_Liver_AR_Vasc
**30**	Liv_Spln_GI.EffT_Liver_b_Vasc -> Liv_Spln_GI.EffT_Liver_f_Vasc
**31**	Liv_Spln_GI.EffT_Liver_b_Vasc -> null
**32**	Liv_Spln_GI.EffT_Liver_f_Vasc -> Lungs.EffT_f_LungsVasc
**33**	Liv_Spln_GI.EffT_Liver_f_Vasc -> null
**34**	Liv_Spln_GI.EffT_LiverEx -> Lymph_Node.EffT_LN_ExRec
**35**	Liv_Spln_GI.EffT_LiverEx -> null
**36**	Liv_Spln_GI.EffT_Spleen_AR_Vasc -> Liv_Spln_GI.EffT_SpleenEx
**37**	Liv_Spln_GI.EffT_Spleen_AR_Vasc -> null
**38**	Liv_Spln_GI.EffT_Spleen_b_Vasc -> Liv_Spln_GI.EffT_Spleen_AR_Vasc
**39**	Liv_Spln_GI.EffT_Spleen_b_Vasc -> Liv_Spln_GI.EffT_Spleen_f_Vasc
**40**	Liv_Spln_GI.EffT_Spleen_b_Vasc -> null
**41**	Liv_Spln_GI.EffT_Spleen_f_Vasc + Liv_Spln_GI.Spleen_Free_Sites -> Liv_Spln_GI.EffT_Spleen_b_Vasc + Liv_Spln_GI.Spleen_Free_Sites
**42**	Liv_Spln_GI.EffT_Spleen_f_Vasc -> Liv_Spln_GI.EffT_Liver_f_Vasc
**43**	Liv_Spln_GI.EffT_Spleen_f_Vasc -> null
**44**	Liv_Spln_GI.EffT_SpleenEx -> Lymph_Node.EffT_LN_ExRec
**45**	Liv_Spln_GI.EffT_SpleenEx -> null
**46**	Liv_Spln_GI.Liver_Free_Sites + Liv_Spln_GI.EffT_Liver_f_Vasc -> Liv_Spln_GI.EffT_Liver_b_Vasc + Liv_Spln_GI.Liver_Free_Sites
**47**	Lungs.EffT_AR_LungsVasc -> Lungs.EffT_LungsEx
**48**	Lungs.EffT_AR_LungsVasc -> null
**49**	Lungs.EffT_AR_LungsVasc -> null
**50**	Lungs.EffT_f_LungsVasc + Lungs.Lung_Free_Sites -> Lungs.EffT_r_LungsVasc + Lungs.Lung_Free_Sites
**51**	Lungs.EffT_f_LungsVasc -> [Blood-Lymph].Effector_TB
**52**	Lungs.EffT_f_LungsVasc -> null
**53**	Lungs.EffT_LungsEx -> Lymph_Node.EffT_LN_ExRec
**54**	Lungs.EffT_LungsEx -> null
**55**	Lungs.EffT_r_LungsVasc -> Lungs.EffT_AR_LungsVasc

Reaction Number	Reaction
56	Lungs.EffT_r_LungsVasc -> Lungs.EffT_f_LungsVasc
57	Lungs.EffT_r_LungsVasc -> null
58	Lungs.EffT_r_LungsVasc -> null
59	Lymph_Node.[PNT-Tr_CD80] + Lymph_Node.[Tr-PNT_PDL1] <-> Lymph_Node.[TrPNT_PDL1-CD80]
60	Lymph_Node.[PNT-Tr_CD80] -> null
61	Lymph_Node.[PNT-Tr_PD1] + Lymph_Node.[Tr-PNT_PDL1] <-> Lymph_Node.[TrPNT_PDL1-PD1]
62	Lymph_Node.[PNT-Tr_PD1] + Lymph_Node.PD1_mab <-> Lymph_Node.PNTTr_PD1_aPD1
63	Lymph_Node.[PNT-Tr_PD1] -> null
64	Lymph_Node.[PNT-Tr_PDL1] + Lymph_Node.PDL1_mab <-> Lymph_Node.PNTTr_PDL1_aPDL1
65	Lymph_Node.[PNT-Tr_PDL1] -> null
66	Lymph_Node.[TregLN-NT] + Lymph_Node.Naive_T -> null
67	Lymph_Node.[TregLN-NT] -> null
68	Lymph_Node.[TregLN-PNT] + Lymph_Node.Primed_Naive_T -> null
69	Lymph_Node.[TregLN-PNT] -> null
70	Lymph_Node.[TregLN-PNT1] -> Lymph_Node.[PNT-Tr_CD80] + Lymph_Node.[TregLN-PNT1]
71	Lymph_Node.[TregLN-PNT1] -> Lymph_Node.[PNT-Tr_PD1] + Lymph_Node.[TregLN-PNT1]
72	Lymph_Node.[TregLN-PNT1] -> Lymph_Node.[PNT-Tr_PDL1] + Lymph_Node.[TregLN-PNT1]
73	Lymph_Node.[TregLN-PNT1] -> Lymph_Node.[Tr-PNT_PD1] + Lymph_Node.[TregLN-PNT1]
74	Lymph_Node.[TregLN-PNT1] -> Lymph_Node.[Tr-PNT_PDL1] + Lymph_Node.[TregLN-PNT1]
75	Lymph_Node.[Tr-mAPC_CD80] -> null
76	Lymph_Node.[Tr-mAPC_CD86] -> null
77	Lymph_Node.[Tr-mAPC_CTLA4] + Lymph_Node.[Tr-mAPC_CD80] <-> Lymph_Node.TrALN_CT_CD80
78	Lymph_Node.[Tr-mAPC_CTLA4] + Lymph_Node.[Tr-mAPC_CD86] <-> Lymph_Node.TrALN_CT_CD86
79	Lymph_Node.[Tr-mAPC_CTLA4] + Lymph_Node.CTLA4_mab <-> Lymph_Node.TrALN_CT_aCT
80	Lymph_Node.[Tr-mAPC_CTLA4] -> null
81	Lymph_Node.[Tr-PNT_PD1] + Lymph_Node.[PNT-Tr_PDL1] <-> Lymph_Node.[TrPNT_PD1-L1]
82	Lymph_Node.[Tr-PNT_PD1] + Lymph_Node.PD1_mab <-> Lymph_Node.TrPNT_PD1_aPD1
83	Lymph_Node.[Tr-PNT_PD1] -> null
84	Lymph_Node.[Tr-PNT_PDL1] + Lymph_Node.PDL1_mab <-> Lymph_Node.TrPNT_PDL1_aPDL1
85	Lymph_Node.[Tr-PNT_PDL1] -> null
86	Lymph_Node.APCLN + Lymph_Node.C_DebrisLN -> Lymph_Node.mAPC
87	Lymph_Node.C_DebrisLN -> null
88	Lymph_Node.CTLA4_mab -> [Blood-Lymph].CTLA4_mabB
89	Lymph_Node.CTLA4_mab + Lymph_Node.PNT_CTLA4 <-> Lymph_Node.CTLA4_mAb_CTLA4
90	Lymph_Node.CTLA4_mAb_CTLA4 -> null
91	Lymph_Node.Effector_T -> null
92	Lymph_Node.EffT_LN_ExRec -> Lungs.EffT_f_LungsVasc
93	Lymph_Node.EffT_LN_ExRec -> null
94	Lymph_Node.EffT_LN_f_Vasc -> Lungs.EffT_f_LungsVasc
95	Lymph_Node.EffT_LN_f_Vasc -> null
96	Lymph_Node.mAPC -> Lymph_Node.mAPC_CD80 + Lymph_Node.mAPC
97	Lymph_Node.mAPC -> Lymph_Node.mAPC_CD86 + Lymph_Node.mAPC
98	Lymph_Node.mAPC -> Lymph_Node.mAPC_PD1 + Lymph_Node.mAPC
99	Lymph_Node.mAPC -> Lymph_Node.mAPC_PDL1 + Lymph_Node.mAPC
100	Lymph_Node.mAPC -> Lymph_Node.mAPC_PDL2 + Lymph_Node.mAPC
101	Lymph_Node.mAPC -> null
102	Lymph_Node.mAPC -> null
103	Lymph_Node.mAPC_CD80 -> null
104	Lymph_Node.mAPC_CD86 -> null
105	Lymph_Node.mAPC_Int_P1 -> Lymph_Node.mAPC_Int_P1 + Lymph_Node.mAPC_Int_P1_CD80
106	Lymph_Node.mAPC_Int_P1 -> Lymph_Node.mAPC_Int_P1 + Lymph_Node.mAPC_Int_P1_CD86
107	Lymph_Node.mAPC_Int_P1_CD80 -> null
108	Lymph_Node.mAPC_Int_P1_CD86 -> null
109	Lymph_Node.mAPC_Int_P2 -> Lymph_Node.mAPC_Int_P2_CD80 + Lymph_Node.mAPC_Int_P2
110	Lymph_Node.mAPC_Int_P2 -> Lymph_Node.mAPC_Int_P2_CD86 + Lymph_Node.mAPC_Int_P2
111	Lymph_Node.mAPC_Int_P2 -> Lymph_Node.mAPC_Int_P2_PD1 + Lymph_Node.mAPC_Int_P2
112	Lymph_Node.mAPC_Int_P2 -> Lymph_Node.mAPC_Int_P2_PDL1 + Lymph_Node.mAPC_Int_P2
113	Lymph_Node.mAPC_Int_P2 -> Lymph_Node.mAPC_Int_P2_PDL2 + Lymph_Node.mAPC_Int_P2
114	Lymph_Node.mAPC_Int_P2_CD80 + Lymph_Node.PNT_CTLA4 <-> Lymph_Node.NEG_Sig_PNT_CD80
115	Lymph_Node.mAPC_Int_P2_CD80 + Lymph_Node.PNT_Int_PDL1 <-> Lymph_Node.[PNT_PDL1-CD80]
116	Lymph_Node.mAPC_Int_P2_CD80 -> null
117	Lymph_Node.mAPC_Int_P2_CD86 + Lymph_Node.PNT_CTLA4 <-> Lymph_Node.NEG_Sig_PNT_CD86
118	Lymph_Node.mAPC_Int_P2_CD86 + Lymph_Node.PNT_Int_CD28 <-> Lymph_Node.POS_Sig_PNT_CD86
119	Lymph_Node.mAPC_Int_P2_CD86 -> null
120	Lymph_Node.mAPC_Int_P2_PD1 + Lymph_Node.PD1_mab <-> Lymph_Node.PD1mAb_mAPC_PD1
121	Lymph_Node.mAPC_Int_P2_PD1 -> null
122	Lymph_Node.mAPC_Int_P2_PDL1 -> null
123	Lymph_Node.mAPC_Int_P2_PDL2 + Lymph_Node.PNT_Int_PD1 <-> Lymph_Node.[PNT_PD1-PDL2]

Reaction Number	Reaction
**124**	Lymph_Node.mAPC_Int_P2_PDL2 -> null
**125**	Lymph_Node.mAPC_PD1 -> null
**126**	Lymph_Node.mAPC_PDL1 -> null
**127**	Lymph_Node.mAPC_PDL2 -> null
**128**	Lymph_Node.Naive_T -> Lymph_Node.Naive_T1
**129**	Lymph_Node.Naive_T -> null
**130**	Lymph_Node.Naive_T1 -> Lymph_Node.Naive_T
**131**	Lymph_Node.Naive_T1 -> Lymph_Node.Primed_Naive_T
**132**	Lymph_Node.NEG_Sig_PNT_CD80 -> null
**133**	Lymph_Node.NEG_Sig_PNT_CD86 -> null
**134**	Lymph_Node.NT_Int_CD28 + Lymph_Node.mAPC_Int_P1_CD80 <-> Lymph_Node.POS_Sig_NT_CD80
**135**	Lymph_Node.NT_Int_CD28 + Lymph_Node.mAPC_Int_P1_CD86 <-> Lymph_Node.POS_Sig_NT_CD86
**136**	Lymph_Node.NT_Int_CD28 -> null
**137**	Lymph_Node.NT1_Int1 -> Lymph_Node.NT1_Int1 + Lymph_Node.NT_Int_CD28
**138**	Lymph_Node.PD1_mab -> [Blood-Lymph].PD1_mabb
**139**	Lymph_Node.PDL1_mab -> [Blood-Lymph].PDL1_mabb
**140**	Lymph_Node.PDL1_mab + Lymph_Node.mAPC_Int_P2_PDL1 <-> Lymph_Node.[PDL1mAb-mAPC_PDL1]
**141**	Lymph_Node.PDL1_mab + Lymph_Node.PNT_Int_PDL1 <-> Lymph_Node.[PDL1mAb-PNT_PDL1]
**142**	Lymph_Node.PNT_CTLA4 -> null
**143**	Lymph_Node.PNT_Int_CD28 + Lymph_Node.mAPC_Int_P2_CD80 <-> Lymph_Node.POS_Sig_PNT_CD80
**144**	Lymph_Node.PNT_Int_CD28 -> null
**145**	Lymph_Node.PNT_Int_CD80 + Lymph_Node.mAPC_Int_P2_PDL1 <-> Lymph_Node.[PNT_CD80-PDL1]
**146**	Lymph_Node.PNT_Int_CD80 -> null
**147**	Lymph_Node.PNT_Int_PD1 + Lymph_Node.mAPC_Int_P2_PDL1 <-> Lymph_Node.[PNT_PD1-PDL1]
**148**	Lymph_Node.PNT_Int_PD1 + Lymph_Node.PD1_mab <-> Lymph_Node.PD1mAb_PNT_PD1
**149**	Lymph_Node.PNT_Int_PD1 -> null
**150**	Lymph_Node.PNT_Int_PDL1 + Lymph_Node.mAPC_Int_P2_PD1 <-> Lymph_Node.[PNT_PDL1-PD1]
**151**	Lymph_Node.PNT_Int_PDL1 -> null
**152**	Lymph_Node.PNT1_Int -> Lymph_Node.PNT_CTLA4 + Lymph_Node.PNT1_Int
**153**	Lymph_Node.PNT1_Int -> Lymph_Node.PNT_Int_CD28 + Lymph_Node.PNT1_Int
**154**	Lymph_Node.PNT1_Int -> Lymph_Node.PNT_Int_CD80 + Lymph_Node.PNT1_Int
**155**	Lymph_Node.PNT1_Int -> Lymph_Node.PNT1_Int + Lymph_Node.PNT_Int_PD1
**156**	Lymph_Node.PNT1_Int -> Lymph_Node.PNT1_Int + Lymph_Node.PNT_Int_PDL1
**157**	Lymph_Node.Primed_Naive_T -> Lymph_Node.Primed_Naive_T1
**158**	Lymph_Node.Primed_Naive_T1 -> Lymph_Node.Anergic_Naive_T
**159**	Lymph_Node.Primed_Naive_T1 -> Lymph_Node.Primed_Naive_T
**160**	Lymph_Node.Primed_Naive_T1 -> Lymph_Node.Prolif_Naive_T
**161**	Lymph_Node.Prolif_Naive_T -> Lymph_Node.Effector_T
**162**	Lymph_Node.Prolif_Naive_T -> null
**163**	Lymph_Node.TrALN_CT_aCT -> null
**164**	Lymph_Node.TrALN_CT_CD80 -> null
**165**	Lymph_Node.TrALN_CT_CD86 -> null
**166**	Lymph_Node.TregLN -> Lymph_Node.TregLN + Lymph_Node.TrLN_CTLA4
**167**	Lymph_Node.TregLN + Lymph_Node.mAPC1 -> Lymph_Node.TregLN_mAPC + Lymph_Node.TregLN + Lymph_Node.mAPC1
**168**	Lymph_Node.TregLN + Lymph_Node.Naive_T2 -> Lymph_Node.[TregLN-NT] + Lymph_Node.TregLN + Lymph_Node.Naive_T2
**169**	Lymph_Node.TregLN + Lymph_Node.Primed_Naive_T2 -> Lymph_Node.[TregLN-PNT] + Lymph_Node.TregLN + Lymph_Node.Primed_Naive_T2
**170**	Lymph_Node.TregLN_mAPC + Lymph_Node.mAPC -> null
**171**	Lymph_Node.TregLN_mAPC -> null
**172**	Lymph_Node.TregLN_mAPC1 -> Lymph_Node.[Tr-mAPC_CD80] + Lymph_Node.TregLN_mAPC1
**173**	Lymph_Node.TregLN_mAPC1 -> Lymph_Node.[Tr-mAPC_CD86] + Lymph_Node.TregLN_mAPC1
**174**	Lymph_Node.TregLN_mAPC1 -> Lymph_Node.[Tr-mAPC_CTLA4] + Lymph_Node.TregLN_mAPC1
**175**	Lymph_Node.TrLN_CTLA4 + Lymph_Node.CTLA4_mab <-> Lymph_Node.TrLN_CT_aCT
**176**	Lymph_Node.TrLN_CTLA4 -> null
**177**	null -> [Blood-Lymph].CTLA4_mabB
**178**	null -> [Blood-Lymph].PD1_mabb
**179**	null -> [Blood-Lymph].PDL1_mabb
**180**	null -> Lymph_Node.Naive_T
**181**	null -> Lymph_Node.Prolif_Naive_T
**182**	null -> Tumor.APC_T
**183**	null -> Tumor.Cancer
**184**	Peripheral.CTLA4_mabP_leaky <-> Lymph_Node.CTLA4_mab
**185**	Peripheral.CTLA4_mabP_tight <-> Lymph_Node.CTLA4_mab

Reaction Number	Reaction
186	Peripheral.EffT_P_f_Vasc -> Lungs.EffT_f_LungsVasc
187	Peripheral.EffT_P_f_Vasc -> null
188	Peripheral.PD1_mabP_leaky <-> Lymph_Node.PD1_mab
189	Peripheral.PD1_mabP_tight <-> Lymph_Node.PD1_mab
190	Peripheral.PDL1_mabP_leaky <-> Lymph_Node.PDL1_mab
191	Peripheral.PDL1_mabP_tight <-> Lymph_Node.PDL1_mab
192	Tumor.[C{CD80}] -> Tumor.[C{CD80}] + Tumor.[C5=CD80]
193	Tumor.[C{PD1}] -> Tumor.[C2=PD1] + Tumor.[C{PD1}]
194	Tumor.[C{PD1}{CD80}] -> Tumor.[C{PD1}{CD80}] + Tumor.[C9a=PD1]
195	Tumor.[C{PD1}{CD80}] -> Tumor.[C{PD1}{CD80}] + Tumor.[C9b=CD80]
196	Tumor.[C{PD1}{PDL1}] -> Tumor.[C6a=PD1] + Tumor.[C{PD1}{PDL1}]
197	Tumor.[C{PD1}{PDL1}] -> Tumor.[C6b=PDL1] + Tumor.[C{PD1}{PDL1}]
198	Tumor.[C{PD1}{PDL1}{CD80}] -> Tumor.[C{PD1}{PDL1}{CD80}] + Tumor.[C14a=PD1]
199	Tumor.[C{PD1}{PDL1}{CD80}] -> Tumor.[C{PD1}{PDL1}{CD80}] + Tumor.[C14b=PDL1]
200	Tumor.[C{PD1}{PDL1}{CD80}] -> Tumor.[C{PD1}{PDL1}{CD80}] + Tumor.[C14c=CD80]
201	Tumor.[C{PD1}{PDL1}{PDL2}] -> Tumor.[C{PD1}{PDL1}{PDL2}] + Tumor.[C10a=PD1]
202	Tumor.[C{PD1}{PDL1}{PDL2}] -> Tumor.[C{PD1}{PDL1}{PDL2}] + Tumor.[C10b=PDL1]
203	Tumor.[C{PD1}{PDL1}{PDL2}] -> Tumor.[C{PD1}{PDL1}{PDL2}] + Tumor.[C10c=PDL2]
204	Tumor.[C{PD1}{PDL1}{PDL2}{CD80}] -> Tumor.[C{PD1}{PDL1}{PDL2}{CD80}] + Tumor.[C16a=PD1]
205	Tumor.[C{PD1}{PDL1}{PDL2}{CD80}] -> Tumor.[C{PD1}{PDL1}{PDL2}{CD80}] + Tumor.[C16b=PDL1]
206	Tumor.[C{PD1}{PDL1}{PDL2}{CD80}] -> Tumor.[C{PD1}{PDL1}{PDL2}{CD80}] + Tumor.[C16c=PDL2]
207	Tumor.[C{PD1}{PDL1}{PDL2}{CD80}] -> Tumor.[C{PD1}{PDL1}{PDL2}{CD80}] + Tumor.[C16d=CD80]
208	Tumor.[C{PD1}{PDL2}] -> Tumor.[C{PD1}{PDL2}] + Tumor.[C7a=PD1]
209	Tumor.[C{PD1}{PDL2}] -> Tumor.[C{PD1}{PDL2}] + Tumor.[C7b=PDL2]
210	Tumor.[C{PD1}{PDL2}{CD80}] -> Tumor.[C{PD1}{PDL2}{CD80}] + Tumor.[C15a=PD1]
211	Tumor.[C{PD1}{PDL2}{CD80}] -> Tumor.[C{PD1}{PDL2}{CD80}] + Tumor.[C15b=PDL2]
212	Tumor.[C{PD1}{PDL2}{CD80}] -> Tumor.[C{PD1}{PDL2}{CD80}] + Tumor.[C15c=CD80]
213	Tumor.[C{PDL1}] -> Tumor.[C{PDL1}] + Tumor.[C3=PDL1]
214	Tumor.[C{PDL1}{CD80}] -> Tumor.[C{PDL1}{CD80}] + Tumor.[C11a=PDL1]
215	Tumor.[C{PDL1}{CD80}] -> Tumor.[C{PDL1}{CD80}] + Tumor.[C11b=CD80]
216	Tumor.[C{PDL1}{PDL2}] -> Tumor.[C{PDL1}{PDL2}] + Tumor.[C8a=PDL1]
217	Tumor.[C{PDL1}{PDL2}] -> Tumor.[C{PDL1}{PDL2}] + Tumor.[C8b=PDL2]
218	Tumor.[C{PDL1}{PDL2}{CD80}] -> Tumor.[C{PDL1}{PDL2}{CD80}] + Tumor.[C13a=PDL1]
219	Tumor.[C{PDL1}{PDL2}{CD80}] -> Tumor.[C{PDL1}{PDL2}{CD80}] + Tumor.[C13b=PDL2]
220	Tumor.[C{PDL1}{PDL2}{CD80}] -> Tumor.[C{PDL1}{PDL2}{CD80}] + Tumor.[C13c=CD80]
221	Tumor.[C{PDL2}] -> Tumor.[C{PDL2}] + Tumor.[C4=PDL2]
222	Tumor.[C{PDL2}{CD80}] -> Tumor.[C{PDL2}{CD80}] + Tumor.[C12a=PDL2]
223	Tumor.[C{PDL2}{CD80}] -> Tumor.[C{PDL2}{CD80}] + Tumor.[C12b=CD80]
224	Tumor.[C10a=PD1] + Tumor.PD1_mabt <-> Tumor.[C10a=PD1:aPD1]
225	Tumor.[C10a=PD1] -> null
226	Tumor.[C10b=PDL1] + Tumor.PDL1_mabt <-> Tumor.[C10b=PDL1:aPDL1]
227	Tumor.[C10b=PDL1] -> null
228	Tumor.[C10c=PDL2] -> null
229	Tumor.[C11a=PDL1] + Tumor.PDL1_mabt <-> Tumor.[C11a=PDL1:aPDL1]
230	Tumor.[C11a=PDL1] -> null
231	Tumor.[C11b=CD80] -> null
232	Tumor.[C12a=PDL2] -> null
233	Tumor.[C12b=CD80] -> null
234	Tumor.[C13a=PDL1] + Tumor.PDL1_mabt <-> Tumor.[C13a=PDL1:aPDL1]
235	Tumor.[C13a=PDL1] -> null
236	Tumor.[C13b=PDL2] -> null
237	Tumor.[C13c=CD80] -> null
238	Tumor.[C14a=PD1] + Tumor.[T14a=PDL1] <-> Tumor.[T14a=PDL1:PD1=C14a]
239	Tumor.[C14a=PD1] -> null
240	Tumor.[C14b=PDL1] + Tumor.[T14c=CD80] <-> Tumor.[T14c=CD80:PDL1=C14b]
241	Tumor.[C14b=PDL1] + Tumor.PDL1_mabt <-> Tumor.[C14b=PDL1:aPDL1]
242	Tumor.[C14b=PDL1] -> null
243	Tumor.[C14c=CD80] + Tumor.[T14a=PDL1] <-> Tumor.[T14a=PDL1:CD80=C14c]
244	Tumor.[C14c=CD80] -> null
245	Tumor.[C15a=PD1] + Tumor.PD1_mabt <-> Tumor.[C15a=PD1:aPD1]
246	Tumor.[C15a=PD1] -> null
247	Tumor.[C15b=PDL2] + Tumor.[T15b=PD1] <-> Tumor.[T15b=PD1:PDL2=C15b]
248	Tumor.[C15b=PDL2] -> null
249	Tumor.[C15c=CD80] + Tumor.[T15a=PDL1] <-> Tumor.[T15a=PDL1:CD80=C15c]
250	Tumor.[C15c=CD80] -> null
251	Tumor.[C16a=PD1] + Tumor.[T16a=PDL1] <-> Tumor.[T16a=PDL1:PD1=C16a]

Reaction Number	Reaction
**252**	Tumor.[C16a=PD1] + Tumor.PD1_mabt <-> Tumor.[C16a=PD1:aPD1]
**253**	Tumor.[C16a=PD1] -> null
**254**	Tumor.[C16b=PDL1] + Tumor.PDL1_mabt <-> Tumor.[C16b=PDL1:aPDL1]
**255**	Tumor.[C16b=PDL1] -> null
**256**	Tumor.[C16c=PDL2] + Tumor.[T16b=PD1] <-> Tumor.[T16b=PD1:PDL2=C16c]
**257**	Tumor.[C16c=PDL2] -> null
**258**	Tumor.[C16d=CD80] -> null
**259**	Tumor.[C2=PD1] -> null
**260**	Tumor.[C3=PDL1] -> null
**261**	Tumor.[C4=PDL2] -> null
**262**	Tumor.[C5=CD80] -> null
**263**	Tumor.[C6a=PD1] + Tumor.PD1_mabt <-> Tumor.[C6a=PD1:aPD1]
**264**	Tumor.[C6a=PD1] -> null
**265**	Tumor.[C6b=PDL1] + Tumor.[T6b=PD1] <-> Tumor.[T6b=PD1:PDL1=C6b]
**266**	Tumor.[C6b=PDL1] + Tumor.[T6c=CD80] <-> Tumor.[T6c=CD80:PDL1=C6b]
**267**	Tumor.[C6b=PDL1] + Tumor.PDL1_mabt <-> Tumor.[C6b=PDL1:aPDL1]
**268**	Tumor.[C6b=PDL1] -> null
**269**	Tumor.[C7a=PD1] -> null
**270**	Tumor.[C7b=PDL2] -> null
**271**	Tumor.[C8a=PDL1] + Tumor.[T8b=CD80] <-> Tumor.[T8b=CD80:PDL1=C8a]
**272**	Tumor.[C8a=PDL1] + Tumor.PDL1_mabt <-> Tumor.[C8a=PDL1:aPDL1]
**273**	Tumor.[C8a=PDL1] -> null
**274**	Tumor.[C8b=PDL2] -> null
**275**	Tumor.[C9a=PD1] + Tumor.[T9=PDL1] <-> Tumor.[T9=PDL1-PD1=C9a]
**276**	Tumor.[C9a=PD1] -> null
**277**	Tumor.[C9b=CD80] -> null
**278**	Tumor.[CTLA4:CD80_TrAT] -> null
**279**	Tumor.[CTLA4:CD86_TrAT] -> null
**280**	Tumor.[CTLA4_CTLA4-Trt] -> null
**281**	Tumor.[T{PD1}{80}-{PDL1}{PDL2}C] -> Tumor.[T{PD1}{80}-{PDL1}{PDL2}C] + Tumor.[T8a=PD1]
**282**	Tumor.[T{PD1}{80}-{PDL1}{PDL2}C] -> Tumor.[T{PD1}{80}-{PDL1}{PDL2}C] + Tumor.[T8b=CD80]
**283**	Tumor.[T{PD1}{CD80}-{PDL1}C] -> Tumor.[T{PD1}{CD80}-{PDL1}C] + Tumor.[T3a=PD1]
**284**	Tumor.[T{PD1}{CD80}-{PDL1}C] -> Tumor.[T{PD1}{CD80}-{PDL1}C] + Tumor.[T3b=CD80]
**285**	Tumor.[T{PD1}{L1}{80}-{PD1}{80}{L1}{L2}C] -> Tumor.[T{PD1}{L1}{80}-{PD1}{80}{L1}{L2}C] + Tumor.[T16a=PDL1]
**286**	Tumor.[T{PD1}{L1}{80}-{PD1}{80}{L1}{L2}C] -> Tumor.[T{PD1}{L1}{80}-{PD1}{80}{L1}{L2}C] + Tumor.[T16b=PD1]
**287**	Tumor.[T{PD1}{L1}{80}-{PD1}{80}{L1}{L2}C] -> Tumor.[T{PD1}{L1}{80}-{PD1}{80}{L1}{L2}C] + Tumor.[T16c=CD80]
**288**	Tumor.[T{PD1}{L1}{80}-{PD1}{80}{L1}C] -> Tumor.[T{PD1}{L1}{80}-{PD1}{80}{L1}C] + Tumor.[T14a=PDL1]
**289**	Tumor.[T{PD1}{L1}{80}-{PD1}{80}{L1}C] -> Tumor.[T{PD1}{L1}{80}-{PD1}{80}{L1}C] + Tumor.[T14b=PD1]
**290**	Tumor.[T{PD1}{L1}{80}-{PD1}{80}{L1}C] -> Tumor.[T{PD1}{L1}{80}-{PD1}{80}{L1}C] + Tumor.[T14c=CD80]
**291**	Tumor.[T{PD1}{L1}{80}-{PD1}{L1}{L2}C] -> Tumor.[T{PD1}{L1}{80}-{PD1}{L1}{L2}C] + Tumor.[T10a=PDL1]
**292**	Tumor.[T{PD1}{L1}{80}-{PD1}{L1}{L2}C] -> Tumor.[T{PD1}{L1}{80}-{PD1}{L1}{L2}C] + Tumor.[T10b=PD1]
**293**	Tumor.[T{PD1}{L1}{80}-{PD1}{L1}{L2}C] -> Tumor.[T{PD1}{L1}{80}-{PD1}{L1}{L2}C] + Tumor.[T10c=CD80]
**294**	Tumor.[T{PD1}{L1}{80}-{PD1}{L1}C] -> Tumor.[T{PD1}{L1}{80}-{PD1}{L1}C] + Tumor.[T6a=PDL1]
**295**	Tumor.[T{PD1}{L1}{80}-{PD1}{L1}C] -> Tumor.[T{PD1}{L1}{80}-{PD1}{L1}C] + Tumor.[T6b=PD1]
**296**	Tumor.[T{PD1}{L1}{80}-{PD1}{L1}C] -> Tumor.[T{PD1}{L1}{80}-{PD1}{L1}C] + Tumor.[T6c=CD80]
**297**	Tumor.[T{PD1}{L1}-{PD1}{80}{PDL2}C] -> Tumor.[T{PD1}{L1}-{PD1}{80}{PDL2}C] + Tumor.[T15a=PDL1]
**298**	Tumor.[T{PD1}{L1}-{PD1}{80}{PDL2}C] -> Tumor.[T{PD1}{L1}-{PD1}{80}{PDL2}C] + Tumor.[T15b=PD1]
**299**	Tumor.[T{PD1}{L1}-{PD1}{L2}C] -> Tumor.[T{PD1}{L1}-{PD1}{L2}C] + Tumor.[T7a=PDL1]
**300**	Tumor.[T{PD1}{L1}-{PD1}{L2}C] -> Tumor.[T{PD1}{L1}-{PD1}{L2}C] + Tumor.[T7b=PD1]
**301**	Tumor.[T{PD1}{L1}-{PDL1}{80}{L2}C] -> Tumor.[T{PD1}{L1}-{PDL1}{80}{L2}C] + Tumor.[T13a=PDL1]
**302**	Tumor.[T{PD1}{L1}-{PDL1}{80}{L2}C] -> Tumor.[T{PD1}{L1}-{PDL1}{80}{L2}C] + Tumor.[T13b=PD1]
**303**	Tumor.[T{PD1}{L1}-{PDL1}{80}C] -> Tumor.[T{PD1}{L1}-{PDL1}{80}C] + Tumor.[T11a=PDL1]
**304**	Tumor.[T{PD1}{L1}-{PDL1}{80}C] -> Tumor.[T{PD1}{L1}-{PDL1}{80}C] + Tumor.[T11b=PD1]
**305**	Tumor.[T{PD1}{L1}-{PDL2}{80}C] -> Tumor.[T{PD1}{L1}-{PDL2}{80}C] + Tumor.[T12a=PDL1]
**306**	Tumor.[T{PD1}{L1}-{PDL2}{80}C] -> Tumor.[T{PD1}{L1}-{PDL2}{80}C] + Tumor.[T12b=PD1]
**307**	Tumor.[T{PD1}-{PDL2}C] -> Tumor.[T{PD1}-{PDL2}C] + Tumor.[T4=PD1]
**308**	Tumor.[T{PDL1}-{CD80}{PD1}C] -> Tumor.[T{PDL1}-{CD80}{PD1}C] + Tumor.[T9=PDL1]
**309**	Tumor.[T{PDL1}-{CD80}C] -> Tumor.[T{PDL1}-{CD80}C] + Tumor.[T5=PDL1]
**310**	Tumor.[T{PDL1}-{PD1}C] -> Tumor.[T{PDL1}-{PD1}C] + Tumor.[T2=PDL1]
**311**	Tumor.[T10a=PDL1] + Tumor.[C10a=PD1] <-> Tumor.[T10a=PDL1:PD1=C10a]
**312**	Tumor.[T10a=PDL1] + Tumor.PDL1_mabt <-> Tumor.[T10a=PDL1:aPDL1]
**313**	Tumor.[T10a=PDL1] -> null
**314**	Tumor.[T10b=PD1] + Tumor.[C10b=PDL1] <-> Tumor.[T10b=PD1:PDL1=C10b]
**315**	Tumor.[T10b=PD1] + Tumor.[C10c=PDL2] <-> Tumor.[T10b=PD1:PDL2=C10c]
**316**	Tumor.[T10b=PD1] -> null

Reaction Number	Reaction
317	Tumor.[T10c=CD80] + Tumor.[C10b=PDL1] <-> Tumor.[T10c=CD80:PDL1=C10b]
318	Tumor.[T10c=CD80] -> null
319	Tumor.[T11a=PDL1] + Tumor.[C11b=CD80] <-> Tumor.[T11a=PDL1:CD80=C11b]
320	Tumor.[T11a=PDL1] + Tumor.PDL1_mabt <-> Tumor.[T11a=PDL1:aPDL1]
321	Tumor.[T11a=PDL1] -> null
322	Tumor.[T11b=PD1] + Tumor.[C11a=PDL1] <-> Tumor.[T11b=PD1:PDL1=C11a]
323	Tumor.[T11b=PD1] -> null
324	Tumor.[T12a=PDL1] + Tumor.[C12b=CD80] <-> Tumor.[T12a=PDL1:CD80=C12b]
325	Tumor.[T12a=PDL1] + Tumor.PDL1_mabt <-> Tumor.[T12a=PDL1:aPDL1]
326	Tumor.[T12a=PDL1] -> null
327	Tumor.[T12b=PD1] + Tumor.[C12a=PDL2] <-> Tumor.[T12b=PD1:PDL2=C12a]
328	Tumor.[T12b=PD1] -> null
329	Tumor.[T13a=PDL1] + Tumor.[C13c=CD80] <-> Tumor.[T13a=PDL1:CD80=C13c]
330	Tumor.[T13a=PDL1] -> null
331	Tumor.[T13b=PD1] + Tumor.[C13a=PDL1] <-> Tumor.[T13b=PD1:PDL1=C13a]
332	Tumor.[T13b=PD1] + Tumor.[C13b=PDL2] <-> Tumor.[T13b=PD1:PDL2=C13b]
333	Tumor.[T13b=PD1] + Tumor.PD1_mabt <-> Tumor.[T13b=PD1:aPD1]
334	Tumor.[T13b=PD1] -> null
335	Tumor.[T14a=PDL1] + Tumor.PDL1_mabt <-> Tumor.[T14a=PDL1:aPDL1]
336	Tumor.[T14a=PDL1] -> null
337	Tumor.[T14b=PD1] + Tumor.[C14b=PDL1] <-> Tumor.[T14b=PD1:PDL1=C14b]
338	Tumor.[T14b=PD1] -> null
339	Tumor.[T14c=CD80] -> null
340	Tumor.[T15a=PDL1] + Tumor.[C15a=PD1] <-> Tumor.[T15a=PDL1:PD1=C15a]
341	Tumor.[T15a=PDL1] + Tumor.PDL1_mabt <-> Tumor.[T15a=PDL1:aPDL1]
342	Tumor.[T15a=PDL1] -> null
343	Tumor.[T15b=PD1] + Tumor.PD1_mabt <-> Tumor.[T15b=PD1:aPD1]
344	Tumor.[T15b=PD1] -> null
345	Tumor.[T16a=PDL1] + Tumor.[C16d=CD80] <-> Tumor.[T16a=PDL1:CD80=C16d]
346	Tumor.[T16a=PDL1] + Tumor.PDL1_mabt <-> Tumor.[T16a=PDL1:aPDL1]
347	Tumor.[T16a=PDL1] -> null
348	Tumor.[T16b=PD1] + Tumor.[C16b=PDL1] <-> Tumor.[T16b=PD1:PDL1=C16b]
349	Tumor.[T16b=PD1] + Tumor.PD1_mabt <-> Tumor.[T16b=PD1:aPD1]
350	Tumor.[T16b=PD1] -> null
351	Tumor.[T16c=CD80] + Tumor.[C16b=PDL1] <-> Tumor.[T16c=CD80:PDL1=C16b]
352	Tumor.[T16c=CD80] -> null
353	Tumor.[T2=PDL1] + Tumor.[C2=PD1] <-> Tumor.[T2=PDL1:PD1=C2]
354	Tumor.[T2=PDL1] -> null
355	Tumor.[T3a=PD1] + Tumor.[C3=PDL1] <-> Tumor.[T3a=PD1:PDL1=C3]
356	Tumor.[T3a=PD1] + Tumor.PD1_mabt <-> Tumor.[T3a=PD1:aPD1]
357	Tumor.[T3a=PD1] -> null
358	Tumor.[T3b=CD80] + Tumor.[C3=PDL1] <-> Tumor.[T3b=CD80:PDL1=C3]
359	Tumor.[T3b=CD80] -> null
360	Tumor.[T4=PD1] + Tumor.[C4=PDL2] <-> Tumor.[T4=PD1:PDL2=C4]
361	Tumor.[T4=PD1] + Tumor.PD1_mabt <-> Tumor.[T4=PD1:aPD1]
362	Tumor.[T4=PD1] -> null
363	Tumor.[T5=PDL1] + Tumor.[C5=CD80] <-> Tumor.[T5=PDL1:CD80=C5]
364	Tumor.[T5=PDL1] + Tumor.PDL1_mabt <-> Tumor.[T5=PDL1:aPDL1]
365	Tumor.[T5=PDL1] -> null
366	Tumor.[T6a=PDL1] + Tumor.[C6a=PD1] <-> Tumor.[T6a=PDL1:PD1=C6a]
367	Tumor.[T6a=PDL1] + Tumor.PDL1_mabt <-> Tumor.[T6a=PDL1:aPDL1]
368	Tumor.[T6a=PDL1] -> null
369	Tumor.[T6b=PD1] + Tumor.PD1_mabt <-> Tumor.[T6b=PD1:aPD1]
370	Tumor.[T6b=PD1] -> null
371	Tumor.[T6c=CD80] -> null
372	Tumor.[T7a=PDL1] + Tumor.[C7a=PD1] <-> Tumor.[T7a=PDL1:PD1=C7a]
373	Tumor.[T7a=PDL1] -> null
374	Tumor.[T7b=PD1] + Tumor.[C7b=PDL2] <-> Tumor.[T7b=PD1:PDL2=C7b]
375	Tumor.[T7b=PD1] -> null
376	Tumor.[T8a=PD1] + Tumor.[C8a=PDL1] <-> Tumor.[T8a=PD1:PDL1=C8a]
377	Tumor.[T8a=PD1] + Tumor.[C8b=PDL2] <-> Tumor.[T8a=PD1:PDL2=C8b]
378	Tumor.[T8a=PD1] -> null
379	Tumor.[T8b=CD80] -> null
380	Tumor.[T9=PDL1] + Tumor.[C9b=CD80] <-> Tumor.[T9=PDL1-CD80=C9b]
381	Tumor.[T9=PDL1] + Tumor.PDL1_mabt <-> Tumor.[T9=PDL1:aPDL1]
382	Tumor.[T9=PDL1] -> null
383	Tumor.APC_T -> null
384	Tumor.C_DebrisT + Tumor.APC_T -> Tumor.mAPC_T

Reaction Number	Reaction
**385**	Tumor.C_DebrisT -> Lymph_Node.C_DebrisLN
**386**	Tumor.C_DebrisT -> null
**387**	Tumor.C_DebrisT -> null
**388**	Tumor.C_DebrisT -> null
**389**	Tumor.Cancer -> (**AntSpread**) Tumor.C_DebrisT
**390**	Tumor.CD80_mAPCT -> null
**391**	Tumor.CD80_TeffT + Tumor.PDL1_TregT <-> Tumor.[PDL1:CD80_TrTeff]
**392**	Tumor.CD80_TeffT -> null
**393**	Tumor.CD80_TeffT1 -> null
**394**	Tumor.CD86_mAPCT -> null
**395**	Tumor.CTLA4_mabt <-> Lymph_Node.CTLA4_mab
**396**	Tumor.CTLA4_TregT + Tumor.CD80_mAPCT <-> Tumor.[CTLA4:CD80_TrAT]
**397**	Tumor.CTLA4_TregT + Tumor.CD86_mAPCT <-> Tumor.[CTLA4:CD86_TrAT]
**398**	Tumor.CTLA4_TregT + Tumor.CTLA4_mabt <-> Tumor.[CTLA4_CTLA4-Trt]
**399**	Tumor.CTLA4_TregT -> null
**400**	Tumor.Effector_TT -> null
**401**	Tumor.Effector_TT -> null
**402**	Tumor.Effector_TT -> Tumor.Effector_TT_Res
**403**	Tumor.Effector_TT_C_Eng + Tumor.Cancer1 -> Tumor.Effector_TT_C_Eng + Tumor.TC1 + Tumor.Cancer1
**404**	Tumor.EffT_AR_Vasc -> null
**405**	Tumor.EffT_AR_Vasc -> Tumor.Effector_TT
**406**	Tumor.EffT_b_Vasc -> null
**407**	Tumor.EffT_b_Vasc -> Tumor.EffT_AR_Vasc
**408**	Tumor.EffT_b_Vasc -> Tumor.EffT_f_Vasc
**409**	Tumor.EffT_f_Vasc + Tumor.Tmr_Free_Sites -> Tumor.EffT_b_Vasc + Tumor.Tmr_Free_Sites
**410**	Tumor.EffT_f_Vasc -> Lungs.EffT_f_LungsVasc
**411**	Tumor.EffT_f_Vasc -> null
**412**	Tumor.mAPC_T -> Lymph_Node.mAPC
**413**	Tumor.mAPC_T -> null
**414**	Tumor.mAPC_T -> null
**415**	Tumor.mAPCT_EngTregT -> Tumor.CD80_mAPCT + Tumor.mAPCT_EngTregT
**416**	Tumor.mAPCT_EngTregT -> Tumor.CD86_mAPCT + Tumor.mAPCT_EngTregT
**417**	Tumor.MDSC_T + Tumor.Effector_TT_MDSCs -> Tumor.MDSCsT_Teff + Tumor.Effector_TT_MDSCs + Tumor.MDSC_T
**418**	Tumor.MDSCsT_EngTeff -> Tumor.PD1_MDSCsT + Tumor.MDSCsT_EngTeff
**419**	Tumor.MDSCsT_EngTeff -> Tumor.PDL1_MDSCsT + Tumor.MDSCsT_EngTeff
**420**	Tumor.MDSCsT_Teff + Tumor.Effector_TT -> null
**421**	Tumor.MDSCsT_Teff -> null
**422**	Tumor.PD1_mabt <-> Lymph_Node.PD1_mab
**423**	Tumor.PD1_mabt + Tumor.[C14a=PD1] <-> Tumor.[C14a=PD1:aPD1]
**424**	Tumor.PD1_mabt + Tumor.[C2=PD1] <-> Tumor.[C2=PD1:aPD1]
**425**	Tumor.PD1_mabt + Tumor.[C7a=PD1] <-> Tumor.[C7a=PD1:aPD1]
**426**	Tumor.PD1_mabt + Tumor.[C9a=PD1] <-> Tumor.[C9a=PD1:aPD1]
**427**	Tumor.PD1_mabt + Tumor.[T10b=PD1] <-> Tumor.[T10b=PD1:aPD1]
**428**	Tumor.PD1_mabt + Tumor.[T11b=PD1] <-> Tumor.[T11b=PD1:aPD1]
**429**	Tumor.PD1_mabt + Tumor.[T12b=PD1] <-> Tumor.[T12b=PD1:aPD1]
**430**	Tumor.PD1_mabt + Tumor.[T14b=PD1] <-> Tumor.[T14b=PD1:aPD1]
**431**	Tumor.PD1_mabt + Tumor.[T7b=PD1] <-> Tumor.[T7b=PD1:aPD1]
**432**	Tumor.PD1_mabt + Tumor.[T8a=PD1] <-> Tumor.[T8a=PD1:aPD1]
**433**	Tumor.PD1_mabt + Tumor.PD1_TeffT <-> Tumor.[PD1:aPD1_Teff]
**434**	Tumor.PD1_MDSCsT + Tumor.PD1_mabt <-> Tumor.[PD1:aPD1_MDSCs]
**435**	Tumor.PD1_MDSCsT + Tumor.PDL1_TeffT1 <-> Tumor.[PD1:PDL1_MDSCT]
**436**	Tumor.PD1_MDSCsT -> null
**437**	Tumor.PD1_TeffT + Tumor.PDL1_TregT <-> Tumor.[PDL1:PD1_TrTeff]
**438**	Tumor.PD1_TeffT -> null
**439**	Tumor.PD1_TeffT1 + Tumor.PD1_mabt <-> Tumor.[PD1:aPD1_Teff1]
**440**	Tumor.PD1_TeffT1 -> null
**441**	Tumor.PD1_TregT + Tumor.PD1_mabt <-> Tumor.[PD1:aPD1_Treg]
**442**	Tumor.PD1_TregT -> null
**443**	Tumor.PDL1_mabt <-> Lymph_Node.PDL1_mab
**444**	Tumor.PDL1_mabt + Tumor.[C3=PDL1] <-> Tumor.[C3=PDL1:aPDL1]
**445**	Tumor.PDL1_mabt + Tumor.[T13a=PDL1] <-> Tumor.[T13a=PDL1:aPDL1]
**446**	Tumor.PDL1_mabt + Tumor.[T2=PDL1] <-> Tumor.[T2=PDL1:aPDL1]
**447**	Tumor.PDL1_mabt + Tumor.[T7a=PDL1] <-> Tumor.[T7a=PDL1:aPDL1]
**448**	Tumor.PDL1_mabt + Tumor.PDL1_TeffT <-> Tumor.[PDL1:aPDL1_Teff]
**417**	Tumor.PDL1_mabt + Tumor.PDL1_TeffT1 <-> Tumor.[PDL1:aPDL1_Teff1]
**418**	Tumor.PDL1_MDSCsT + Tumor.CD80_TeffT1 <-> Tumor.[PDL1:CD80_MDSCT]

Reaction Number	Reaction
**419**	Tumor.PDL1_MDSCsT + Tumor.PD1_TeffT1 <-> Tumor.[PDL1:PD1_MDSCT]
**420**	Tumor.PDL1_MDSCsT + Tumor.PDL1_mabt <-> Tumor.[PDL1:aPDL1_MDSCs]
**421**	Tumor.PDL1_MDSCsT -> null
**422**	Tumor.PDL1_TeffT + Tumor.PD1_TregT <-> Tumor.[PD1:PDL1_TrTeff]
**423**	Tumor.PDL1_TeffT -> null
**424**	Tumor.PDL1_TeffT1 -> null
**425**	Tumor.PDL1_TregT + Tumor.PDL1_mabt <-> Tumor.[PDL1:aPDL1_Treg]
**426**	Tumor.PDL1_TregT -> null
**427**	Tumor.T_Recover_Can_Dead -> null
**428**	Tumor.TC1 + Tumor.Cancer -> (**AntSpread**) Tumor.C_DebrisT
**429**	Tumor.TC1 -> Tumor.T_Recover_Can_Dead
**430**	Tumor.TC2 + Tumor.Effector_TT -> Tumor.TC2
**431**	Tumor.Teff_EngMDSC -> Tumor.Teff_EngMDSC + Tumor.CD80_TeffT1
**432**	Tumor.Teff_EngMDSC -> Tumor.Teff_EngMDSC + Tumor.PD1_TeffT1
**433**	Tumor.Teff_EngMDSC -> Tumor.Teff_EngMDSC + Tumor.PDL1_TeffT1
**434**	Tumor.Teff_EngTregT -> Tumor.CD80_TeffT + Tumor.Teff_EngTregT
**435**	Tumor.Teff_EngTregT -> Tumor.PD1_TeffT + Tumor.Teff_EngTregT
**436**	Tumor.Teff_EngTregT -> Tumor.PDL1_TeffT + Tumor.Teff_EngTregT
**437**	Tumor.TregT + Tumor.Effector_TT_TregT -> Tumor.TregT_Teff + Tumor.Effector_TT_TregT + Tumor.TregT
**438**	Tumor.TregT_EngAPC -> Tumor.CTLA4_TregT + Tumor.TregT_EngAPC
**439**	Tumor.TregT_EngTeff -> Tumor.PD1_TregT + Tumor.TregT_EngTeff
**440**	Tumor.TregT_EngTeff -> Tumor.PDL1_TregT + Tumor.TregT_EngTeff
**441**	Tumor.TregT_Teff + Tumor.Effector_TT -> null
**442**	Tumor.TregT_Teff -> null

Table S1 – Model Reactions (End)

Table S2 – Model Reaction Rates (Start)

Reaction Number	Reaction Rate
**1**	Kpa_LNB*S_LNB*VL*f_LN_CTLA4*([Blood-Lymph].CTLA4_mabB/Vc_CTLA4-Lymph_Node.CTLA4_mab/VL)
**2**	0.67*Q_L*[Blood-Lymph].CTLA4_mabB*(1-Sigma1_CTLA4)/Vc_CTLA4 - (Peripheral.CTLA4_mabP_leaky/(0.35*ISF*KP_CTLA4))*0.33*Q_L*(1-Sigma1_CTLA4)
**3**	0.33*Q_L*[Blood-Lymph].CTLA4_mabB*(1-Sigma2_CTLA4)/Vc_CTLA4-(Peripheral.CTLA4_mabP_tight/(0.65*ISF*KP_CTLA4))*0.33*Q_L*(1-Sigma2_CTLA4)
**4**	Kpa_TB*S_TB*Vt_avg_const*[Blood-Lymph].CTLA4_mabB/Vc_PD1-Kpa_TB*S_TB*Vt_avg_const*Tumor.CTLA4_mabt/(Vex_Tmr)
**5**	Cl_CTLA4*[Blood-Lymph].CTLA4_mabB
**6**	EffT_Migrate*[Blood-Lymph].Effector_T_TB
**7**	QC_GI*[Blood-Lymph].Effector_TB/Vc_Teff
**8**	(QC_Liver-QC_GI-QC_Spleen+LC_GI+LC_Spleen)*[Blood-Lymph].Effector_TB/Vc_Teff
**9**	QC_Spleen * [Blood-Lymph].Effector_TB/Vc_Teff
**10**	QC_LN*[Blood-Lymph].Effector_TB/Vc_Teff
**11**	EffT_Turnover*[Blood-Lymph].Effector_TB
**12**	QC_Periph*[Blood-Lymph].Effector_TB/Vc_Teff
**13**	QC_Tmr*TCytokineHoming*[Blood-Lymph].Effector_TB/Vc_Teff
**14**	Kpa_LNB*S_LNB*VL*f_LN_PD1*([Blood-Lymph].PD1_mabb/Vc_PD1-Lymph_Node.PD1_mab/VL)
**15**	0.67*Q_L*PD1_mabb*(1-Sigma1_PD1)/Vc_PD1-(Peripheral.PD1_mabP_leaky/(0.35*ISF*KP_PD1))*0.33*Q_L*(1-Sigma1_PD1)
**16**	0.33*Q_L*[Blood-Lymph].PD1_mabb*(1-Sigma2_PD1)/Vc_PD1-(Peripheral.PD1_mabP_tight/(0.65*ISF*KP_PD1))*0.33*Q_L*(1-Sigma2_PD1)
**17**	Kpa_TB*S_TB*Vt_avg_const*[Blood-Lymph].PD1_mabb/Vc_PD1-Kpa_TB*S_TB*Vt_avg_const*Tumor.PD1_mabt/(Vex_Tmr)
**18**	(Cl_PD1/Vc_PD1)*[Blood-Lymph].PD1_mabb
**19**	Kpa_LNB*S_LNB*VL*f_LN_PD1*([Blood-Lymph].PDL1_mabb/Vc_PDL1-Lymph_Node.PDL1_mab/VL)
**20**	0.67*Q_L*PDL1_mabb*(1-Sigma1_PDL1)/Vc_PDL1-(Peripheral.PDL1_mabP_leaky/(0.35*ISF*KP_PDL1))*0.33*Q_L*(1-Sigma1_PDL1)
**21**	0.33*Q_L*[Blood-Lymph].PDL1_mabb*(1-Sigma2_PDL1)/Vc_PDL1-(Peripheral.PDL1_mabP_tight/(0.65*ISF*KP_PDL1))*0.33*Q_L*(1-Sigma2_PDL1)

Reaction Number	Reaction Rate
22	Kpa_TB*S_TB*Vt_avg_const*[Blood-Lymph].PDL1_mabb/Vc_PDL1-Kpa_TB*S_TB*Vt_avg_const*Tumor.PDL1_mabt/(Vex_Tmr)
23	(Cl_PDL1/Vc_PDL1)*[Blood-Lymph].PDL1_mabb
24	(Vm/Durvalumab_MW)*[Blood-Lymph].PDL1_mabb/(Km*Vc_PDL1/Durvalumab_MW+[Blood-Lymph].PDL1_mabb)
25	(QC_GI - LC_GI)*Liv_Spln_GI.EffT_GI_f_Vasc/Vv_GI
26	EffT_Turnover*Liv_Spln_GI.EffT_GI_f_Vasc
27	J_Liver*Liv_Spln_GI.EffT_Liver_AR_Vasc
28	EffT_Turnover*Liv_Spln_GI.EffT_Liver_AR_Vasc
29	AR_Liver*[Liv_Spln_GI].EffT_Liver_b_Vasc
30	kr_Periph*Liv_Spln_GI.EffT_Liver_b_Vasc
31	EffT_Turnover*Liv_Spln_GI.EffT_Liver_b_Vasc
32	(QC_Liver - LC_Liver)*Liv_Spln_GI.EffT_Liver_f_Vasc/Vv_Liver
33	EffT_Turnover*Liv_Spln_GI.EffT_Liver_f_Vasc
34	LC_Liver*Delta_Liver*Liv_Spln_GI.EffT_LiverEx/Vext_Liver
35	EffT_Turnover*Liv_Spln_GI.EffT_LiverEx
36	J_Spleen*Liv_Spln_GI.EffT_Spleen_AR_Vasc
37	EffT_Turnover*Liv_Spln_GI.EffT_Spleen_AR_Vasc
38	AR_Spleen*[Liv_Spln_GI].EffT_Spleen_b_Vasc
39	kr_Periph*Liv_Spln_GI.EffT_Spleen_b_Vasc
40	EffT_Turnover*Liv_Spln_GI.EffT_Spleen_b_Vasc
41	kf_Spleen*Liv_Spln_GI.Spleen_Free_Sites*[Liv_Spln_GI].EffT_Spleen_f_Vasc/Vv_Spleen
42	(QC_Spleen - LC_Spleen)*Liv_Spln_GI.EffT_Spleen_f_Vasc/Vv_Spleen
43	EffT_Turnover*Liv_Spln_GI.EffT_Spleen_f_Vasc
44	LC_Spleen*Delta_Spleen*Liv_Spln_GI.EffT_SpleenEx/Vext_Spleen
45	EffT_Turnover*Liv_Spln_GI.EffT_SpleenEx
46	kf_Liver*Liver_Free_Sites*[Liv_Spln_GI].EffT_Liver_f_Vasc/Vv_Liver
47	J_Lungs*Lungs.EffT_AR_LungsVasc
48	EffT_Turnover*Lungs.EffT_AR_LungsVasc
49	E_Lungs*Lungs.EffT_AR_LungsVasc
50	kf_Lungs*Lung_Free_Sites*Lungs.EffT_f_LungsVasc/Vv_Lungs
51	(QC_Lungs - LC_Lungs)*Lungs.EffT_f_LungsVasc/Vv_Lungs
52	EffT_Turnover*Lungs.EffT_f_LungsVasc
53	LC_Lungs*Delta_Lungs*Lungs.EffT_LungsEx/Vext_Lungs
54	EffT_Turnover*Lungs.EffT_LungsEx
55	AR_Lungs*Lungs.EffT_r_LungsVasc
56	kr_Lungs*Lungs.EffT_r_LungsVasc
57	EffT_Turnover*Lungs.EffT_r_LungsVasc
58	E_Lungs*Lungs.EffT_r_LungsVasc
59	[kon_PDL1_CD80]*Lymph_Node.[PNT-Tr_CD80]*Lymph_Node.[Tr-PNT_PDL1]/[Vol_Cell-Rec_Tr-PNT] - [koff_PDL1_CD80]*Lymph_Node.[TrPNT_PDL1-CD80]
60	[Exp_CD28/80/86/PD1/L1/L2]*(Lymph_Node.[PNT-Tr_CD80]+[TrPNT_PDL1-CD80])
61	[kon_PD1_PDL1]*Lymph_Node.[PNT-Tr_PD1]*Lymph_Node.[Tr-PNT_PDL1]/[Vol_Cell-Rec_Tr-PNT] - [koff_PD1_PDL1]*Lymph_Node.[TrPNT_PDL1-PD1]
62	[kon_PD1-PD1mAb]*Lymph_Node.[PNT-Tr_PD1]*Lymph_Node.PD1_mab/Vtdln - [koff_PD1-PD1mAb]*Lymph_Node.PNTTr_PD1_aPD1
63	[Exp_CD28/80/86/PD1/L1/L2]*(Lymph_Node.[PNT-Tr_PD1]+PNTTr_PD1_aPD1+[TrPNT_PDL1-PD1])
64	[kon_PDL1-PDL1mAb]*Lymph_Node.[PNT-Tr_PDL1]*Lymph_Node.PDL1_mab/Vtdln - [koff_PDL1-PDL1mAb]*Lymph_Node.PNTTr_PDL1_aPDL1
65	[Exp_CD28/80/86/PD1/L1/L2]*(Lymph_Node.[PNT-Tr_PDL1]+PNTTr_PDL1_aPDL1+[TrPNT_PD1-L1])
66	[Treg:T_IntTime]*Lymph_Node.[TregLN-NT]
67	[Treg:T_IntTime]*Lymph_Node.[TregLN-NT]
68	[Treg:T_IntTime]*Lymph_Node.[TregLN-PNT]*[Sig_TrPNT=PD1/L1/CD80]
69	[Treg:T_IntTime]*Lymph_Node.[TregLN-PNT]*(1-[Sig_TrPNT=PD1/L1/CD80])
70	[Exp_CD28/80/86/PD1/L1/L2]*Lymph_Node.[TregLN-PNT1]*(([%CD80_receptor_level_PNT]*[CD80_receptors-per-Tcell])/[Avagadro's_Num])*(1/Tr_per_T_cell)
71	[Exp_CD28/80/86/PD1/L1/L2]*Lymph_Node.[TregLN-PNT1]*(([%PD1_receptor_level_PNT]*[PD1_receptors-per-Tcell])/[Avagadro's_Num])*(1/Tr_per_T_cell)
72	[Exp_CD28/80/86/PD1/L1/L2]*Lymph_Node.[TregLN-PNT1]*(([%PDL1_receptor_level_PNT]*[PDL1_receptors-per-Tcell])/[Avagadro's_Num])*(1/Tr_per_T_cell)
73	[Exp_CD28/80/86/PD1/L1/L2]*Lymph_Node.[TregLN-PNT1]*([PD1_receptors-per-Tcell]/[Avagadro's_Num])*(1/T_per_Tr_cell)
74	[Exp_CD28/80/86/PD1/L1/L2]*Lymph_Node.[TregLN-PNT1]*([PDL1_receptors-per-Tcell]/[Avagadro's_Num])*(1/T_per_Tr_cell)
75	[Exp_CD28/80/86/PD1/L1/L2]*(Lymph_Node.[Tr-mAPC_CD80]+TrALN_CT_CD80)
76	[Exp_CD28/80/86/PD1/L1/L2]*(Lymph_Node.[Tr-mAPC_CD86]+TrALN_CT_CD86)
77	[kon_CTLA4_CD80]*Lymph_Node.[Tr-mAPC_CD80]*Lymph_Node.[Tr-mAPC_CTLA4]/[Vol_Cell-Rec_Tr-mAPC] - [koff_CTLA4_CD80]*Lymph_Node.TrALN_CT_CD80

Reaction Number	Reaction Rate
**78**	[kon_CTLA4_CD86]*Lymph_Node.[Tr-mAPC_CD86]*Lymph_Node.[Tr-mAPC_CTLA4]/[Vol_Cell-Rec_Tr-mAPC]-[koff_CTLA4_CD86]*Lymph_Node.TrALN_CT_CD86
**79**	kon_CTLA4mAb_CTLA4*Lymph_Node.[Tr-mAPC_CTLA4]*Lymph_Node.CTLA4_mab/Vtdln - koff_CTLA4mAb_CTLA4*Lymph_Node.TrALN_CT_aCT
**80**	[Exp_CTLA4]*(Lymph_Node.[Tr-mAPC_CTLA4]+[TrALN_CT_aCT]+[TrALN_CT_CD80]+[TrALN_CT_CD86])
**81**	[kon_PD1_PDL1]*Lymph_Node.[Tr-PNT_PD1]*Lymph_Node.[PNT-Tr_PDL1]/[Vol_Cell-Rec_Tr-PNT] - [koff_PD1_PDL1]*Lymph_Node.[TrPNT_PD1-L1]
**82**	[kon_PD1-PD1mAb]*Lymph_Node.[Tr-PNT_PD1]*Lymph_Node.PD1_mab/Vtdln - [koff_PD1-PD1mAb]*Lymph_Node.TrPNT_PD1_aPD1
**83**	[Exp_CD28/80/86/PD1/L1/L2]*(Lymph_Node.[Tr-PNT_PD1]+TrPNT_PD1_aPD1+[TrPNT_PD1-L1])
**84**	[kon_PDL1-PDL1mAb]*Lymph_Node.[Tr-PNT_PDL1]*Lymph_Node.PDL1_mab/Vtdln - [koff_PDL1-PDL1mAb]*Lymph_Node.TrPNT_PDL1_aPDL1
**85**	[Exp_CD28/80/86/PD1/L1/L2]*(Lymph_Node.[Tr-PNT_PDL1]+TrPNT_PDL1_aPDL1+[TrPNT_PDL1-PD1]+[TrPNT_PDL1-CD80])
**86**	Phago_Debris*Lymph_Node.APCLN*Lymph_Node.C_DebrisLN
**87**	Debris_Decay*Lymph_Node.C_DebrisLN
**88**	Q_L/VL*Lymph_Node.CTLA4_mab
**89**	kon_CTLA4mAb_CTLA4*Lymph_Node.CTLA4_mab*Lymph_Node.PNT_CTLA4/Vtdln-koff_CTLA4mAb_CTLA4*Lymph_Node.CTLA4_mAb_CTLA4
**90**	Endo_CTLA4*Lymph_Node.CTLA4_mAb_CTLA4
**91**	EffT_Migrate*Lymph_Node.Effector_T
**92**	LC_LN*Delta_LN*Lymph_Node.EffT_LN_ExRec/Vext_LN
**93**	EffT_Turnover*Lymph_Node.EffT_LN_ExRec
**94**	(QC_LN - LC_LN)*Lymph_Node.EffT_LN_f_Vasc/Vv_LN
**95**	EffT_Turnover*Lymph_Node.EffT_LN_f_Vasc
**96**	Exp_All_mAPCLN*(Lymph_Node.mAPC*([CD80_receptors-per-mAPC]/[Avagadro's_Num]))
**97**	Exp_All_mAPCLN*(Lymph_Node.mAPC*([CD86_receptors-per-mAPC]/[Avagadro's_Num]))
**98**	Exp_All_mAPCLN*Lymph_Node.mAPC*([PD1_receptors-per-mAPC]/[Avagadro's_Num])
**99**	Exp_All_mAPCLN*Lymph_Node.mAPC*([PDL1_receptors-per-mAPC]/[Avagadro's_Num])
**100**	Exp_All_mAPCLN*Lymph_Node.mAPC*([PDL2_receptors-per-mAPC]/[Avagadro's_Num])
**101**	kf_APC_turnover*Lymph_Node.mAPC
**102**	kf_TregLNS_Inact*Lymph_Node.mAPC*CTLA4Sig_Secrete
**103**	Exp_All_mAPCLN*(Lymph_Node.mAPC_CD80)
**104**	Exp_All_mAPCLN*(Lymph_Node.mAPC_CD86)
**105**	[Exp_CD28/80/86/PD1/L1/L2]*((Lymph_Node.mAPC_CD80)*(Lymph_Node.mAPC_Int_P1/(Lymph_Node.mAPC+ 1E-100*mole)))*(1/T_cells_per_mAPC)
**106**	[Exp_CD28/80/86/PD1/L1/L2]*(Lymph_Node.mAPC_CD86*(Lymph_Node.mAPC_Int_P1/(Lymph_Node.mAPC+ 1E-100*mole)))*(1/T_cells_per_mAPC)
**107**	[Exp_CD28/80/86/PD1/L1/L2]*(Lymph_Node.mAPC_Int_P1_CD80 + POS_Sig_NT_CD80)
**108**	[Exp_CD28/80/86/PD1/L1/L2]*(Lymph_Node.mAPC_Int_P1_CD86 + POS_Sig_NT_CD86)
**109**	[Exp_CD28/80/86/PD1/L1/L2]*((Lymph_Node.mAPC_CD80)*(Lymph_Node.mAPC_Int_P2/(Lymph_Node.mAPC+1E-100*mole)))*(1/T_cells_per_mAPC)
**110**	[Exp_CD28/80/86/PD1/L1/L2]*(Lymph_Node.mAPC_CD86*(Lymph_Node.mAPC_Int_P2/(Lymph_Node.mAPC+1E-100*mole)))*(1/T_cells_per_mAPC)
**111**	[Exp_CD28/80/86/PD1/L1/L2]*Lymph_Node.mAPC_PD1*(Lymph_Node.mAPC_Int_P2/(Lymph_Node.mAPC+1E-100*mole))*(1/T_cells_per_mAPC)
**112**	[Exp_CD28/80/86/PD1/L1/L2]*Lymph_Node.mAPC_PDL1*(Lymph_Node.mAPC_Int_P2/(Lymph_Node.mAPC+1E-100*mole))*(1/T_cells_per_mAPC)
**113**	[Exp_CD28/80/86/PD1/L1/L2]*Lymph_Node.mAPC_PDL2*(Lymph_Node.mAPC_Int_P2/(Lymph_Node.mAPC+1E-100*mole))*(1/T_cells_per_mAPC)
**114**	[kon_CTLA4_CD80]*Lymph_Node.mAPC_Int_P2_CD80*Lymph_Node.PNT_CTLA4/([Volume_PNT-Receptor_Int])-[koff_CTLA4_CD80]*Lymph_Node.NEG_Sig_PNT_CD80
**115**	[kon_PDL1_CD80]*Lymph_Node.mAPC_Int_P2_CD80*Lymph_Node.PNT_Int_PDL1/[Volume_PNT-Receptor_Int]-[koff_PDL1_CD80]*Lymph_Node.[PNT_PDL1-CD80]
**116**	[Exp_CD28/80/86/PD1/L1/L2]*(Lymph_Node.mAPC_Int_P2_CD80+NEG_Sig_PNT_CD80+POS_Sig_PNT_CD80+[PNT_PDL1-CD80])
**117**	[kon_CTLA4_CD86]*Lymph_Node.mAPC_Int_P2_CD86*Lymph_Node.PNT_CTLA4/([Volume_PNT-Receptor_Int])-[koff_CTLA4_CD86]*Lymph_Node.NEG_Sig_PNT_CD86
**118**	[kon_CD28_CD86]*Lymph_Node.mAPC_Int_P2_CD86*Lymph_Node.PNT_Int_CD28/([Volume_PNT-Receptor_Int])-[koff_CD28_CD86]*Lymph_Node.POS_Sig_PNT_CD86
**119**	[Exp_CD28/80/86/PD1/L1/L2]*(Lymph_Node.mAPC_Int_P2_CD86+NEG_Sig_PNT_CD86+POS_Sig_PNT_CD86)
**120**	[kon_PD1-PD1mAb]*Lymph_Node.mAPC_Int_P2_PD1*Lymph_Node.PD1_mab/Vtdln-[koff_PD1-PD1mAb]*[PD1mAb_mAPC_PD1]
**121**	[Exp_CD28/80/86/PD1/L1/L2]*(Lymph_Node.mAPC_Int_P2_PD1+PD1mAb_mAPC_PD1+[PNT_PDL1-PD1])
**122**	[Exp_CD28/80/86/PD1/L1/L2]*(Lymph_Node.mAPC_Int_P2_PDL1+[PDL1mAb-mAPC_PDL1]+[PNT_PD1-PDL1]+[PNT_CD80-PDL1])
**123**	[kon_PD1_PDL2]*Lymph_Node.mAPC_Int_P2_PDL2*Lymph_Node.PNT_Int_PD1/[Volume_PNT-Receptor_Int]-[koff_PD1_PDL2]*Lymph_Node.[PNT_PD1-PDL2]

Reaction Number	Reaction Rate
124	[Exp_CD28/80/86/PD1/L1/L2]*(Lymph_Node.mAPC_Int_P2_PDL2+[PNT_PD1-PDL2])
125	Exp_All_mAPCLN*Lymph_Node.mAPC_PD1
126	Exp_All_mAPCLN*Lymph_Node.mAPC_PDL1
127	Exp_All_mAPCLN*Lymph_Node.mAPC_PDL2
128	PrimeNT_rate*Lymph_Node.Naive_T*[Prob_NT-mAPC_Interact]*[Sig_NT=CD28]
129	EffT_InOutLN*Lymph_Node.Naive_T
130	PrimeNT1_rate*Lymph_Node.Naive_T1*(1/Antigen_Intensity)*((mAPC_Int_P1)/(mAPC_Int_P1+Naive_T1*mAPC50_per_T_cell+0.001*mole))
131	PrimeNT1_rate*Lymph_Node.Naive_T1*Antigen_Intensity*((mAPC_Int_P1)/(mAPC_Int_P1+Naive_T1*mAPC50_per_T_cell+0.001*mole))
132	Endo_CTLA4*Lymph_Node.NEG_Sig_PNT_CD80
133	Endo_CTLA4*Lymph_Node.NEG_Sig_PNT_CD86
134	[kon_CD28_CD80]*Lymph_Node.NT_Int_CD28*Lymph_Node.mAPC_Int_P1_CD80/[Volume_NT-Receptor_Int] - [koff_CD28_CD80]*Lymph_Node.POS_Sig_NT_CD80
135	[kon_CD28_CD86]*Lymph_Node.NT_Int_CD28*Lymph_Node.mAPC_Int_P1_CD86/[Volume_NT-Receptor_Int] - [koff_CD28_CD86]*Lymph_Node.POS_Sig_NT_CD86
136	[Exp_CD28/80/86/PD1/L1/L2]*(Lymph_Node.NT_Int_CD28+POS_Sig_NT_CD80+POS_Sig_NT_CD86)
137	[Exp_CD28/80/86/PD1/L1/L2]*(([CD28_receptors-per-Tcell]/[Avagadro's_Num]))*(mAPC_Int_P1/(mAPC_per_T_cell))
138	Q_L/VL*Lymph_Node.PD1_mab
139	Q_L/VL*Lymph_Node.PDL1_mab
140	[kon_PDL1-PDL1mAb]*Lymph_Node.PDL1_mab*Lymph_Node.mAPC_Int_P2_PDL1/Vtdln - [koff_PDL1-PDL1mAb]*Lymph_Node.[PDL1mAb-mAPC_PDL1]
141	[kon_PDL1-PDL1mAb]*Lymph_Node.PDL1_mab*Lymph_Node.PNT_Int_PDL1/Vtdln - [koff_PDL1-PDL1mAb]*Lymph_Node.[PDL1mAb-PNT_PDL1]
142	[Exp_CTLA4]*(Lymph_Node.PNT_CTLA4+NEG_Sig_PNT_CD80+[NEG_Sig_PNT_CD86]+[CTLA4_mAb_CTLA4])
143	[kon_CD28_CD80]*Lymph_Node.PNT_Int_CD28*Lymph_Node.mAPC_Int_P2_CD80/([Volume_PNT-Receptor_Int])-[koff_CD28_CD80]*Lymph_Node.POS_Sig_PNT_CD80
144	[Exp_CD28/80/86/PD1/L1/L2]*(Lymph_Node.PNT_Int_CD28+POS_Sig_PNT_CD80+POS_Sig_PNT_CD86)
145	[kon_PDL1_CD80]*Lymph_Node.PNT_Int_CD80*Lymph_Node.mAPC_Int_P2_PDL1/[Volume_PNT-Receptor_Int]-[koff_PDL1_CD80]*Lymph_Node.[PNT_CD80-PDL1]
146	[Exp_CD28/80/86/PD1/L1/L2]*(Lymph_Node.PNT_Int_CD80+[PNT_CD80-PDL1])
147	[kon_PD1_PDL1]*Lymph_Node.PNT_Int_PD1*Lymph_Node.mAPC_Int_P2_PDL1/[Volume_PNT-Receptor_Int]-[koff_PD1_PDL1]*Lymph_Node.[PNT_PD1-PDL1]
148	[kon_PD1-PD1mAb]*Lymph_Node.PNT_Int_PD1*Lymph_Node.PD1_mab/Vtdln-[koff_PD1-PD1mAb]*[PD1mAb_PNT_PD1]
149	[Exp_CD28/80/86/PD1/L1/L2]*(Lymph_Node.PNT_Int_PD1+PD1mAb_PNT_PD1+[PNT_PD1-PDL2]+[PNT_PD1-PDL1])
150	[kon_PD1_PDL1]*Lymph_Node.PNT_Int_PDL1*Lymph_Node.mAPC_Int_P2_PD1/[Volume_PNT-Receptor_Int]-[koff_PD1_PDL1]*Lymph_Node.[PNT_PDL1-PD1]
151	[Exp_CD28/80/86/PD1/L1/L2]*(Lymph_Node.PNT_Int_PDL1+[PDL1mAb-PNT_PDL1]+[PNT_PDL1-CD80]+[PNT_PDL1-PD1])
152	[Exp_CTLA4]*(([CTLA4_receptors-Int-PNT]/[Avagadro's_Num]))*(mAPC_Int_P2/(mAPC_per_T_cell))
153	[Exp_CD28/80/86/PD1/L1/L2]*(([CD28_receptors-per-Tcell]/[Avagadro's_Num]))*(mAPC_Int_P2/(mAPC_per_T_cell))
154	[Exp_CD28/80/86/PD1/L1/L2]*((([%CD80_receptor_level_PNT]*[CD80_receptors-per-Tcell])/[Avagadro's_Num]))*(mAPC_Int_P2/(mAPC_per_T_cell))
155	[Exp_CD28/80/86/PD1/L1/L2]*((([%PD1_receptor_level_PNT]*[PD1_receptors-per-Tcell])/[Avagadro's_Num]))*(mAPC_Int_P2/(mAPC_per_T_cell))
156	[Exp_CD28/80/86/PD1/L1/L2]*((([%PDL1_receptor_level_PNT]*[PDL1_receptors-per-Tcell])/[Avagadro's_Num]))*(mAPC_Int_P2/(mAPC_per_T_cell))
157	kf_Phase2P*Lymph_Node.Primed_Naive_T*[Prob_PNT-mAPC_Interact]
158	kf_no_prolif*Lymph_Node.Primed_Naive_T1*[Sig_PNT=CTLA4/PD1/L1/CD80]
159	PrimeTLN2_rate*Lymph_Node.Primed_Naive_T1*(1/Antigen_Intensity)*((mAPC_Int_P2)/(mAPC_Int_P2+Primed_Naive_T1*mAPC50_per_T_cell+0.001*mole))
160	PrimeTLN2_rate*Lymph_Node.Primed_Naive_T1*(1-[Sig_PNT=CTLA4/PD1/L1/CD80])*Antigen_Intensity*((mAPC_Int_P2)/(mAPC_Int_P2 +Primed_Naive_T1* mAPC50_per_T_cell +0.001*mole))
161	kf_Prolif_end*Lymph_Node.Prolif_Naive_T
162	Lymph_Node.Prolif_Naive_T*[Prolif_Thresh]
163	Endo_CTLA4*Lymph_Node.TrALN_CT_aCT
164	Endo_CTLA4*Lymph_Node.TrALN_CT_CD80
165	Endo_CTLA4*Lymph_Node.TrALN_CT_CD86
166	[Exp_CTLA4]*Lymph_Node.TregLN*([CTLA4_receptors-Tr]/[Avagadro's_Num])
167	TregLN_Engage*Lymph_Node.mAPC1*((Lymph_Node.TregLN /(Lymph_Node.mAPC1 + 1E-100*mole))^gamma_TC_Assoc)/(s_Assoc + (Lymph_Node.TregLN /(Lymph_Node.mAPC1 + 1E-100*mole))^gamma_TC_Assoc)

Reaction Number	Reaction Rate
168	TregLN_Engage*Lymph_Node.Naive_T2*((Lymph_Node.TregLN /(Lymph_Node.Naive_T2 + 1E-100*mole))^gamma_TC_Assoc)/(s_Assoc + (Lymph_Node.TregLN /(Lymph_Node.Naive_T2 + 1E-100*mole))^gamma_TC_Assoc)
169	TregLN_Engage* Lymph_Node.Primed_Naive_T2*((Lymph_Node.TregLN /(Lymph_Node.Primed_Naive_T2+ 1E-100*mole))^gamma_TC_Assoc)/(s_Assoc + (Lymph_Node.TregLN /(Lymph_Node.Primed_Naive_T2+ 1E-100*mole))^gamma_TC_Assoc)
170	[Treg:mAPC_IntTime]*Lymph_Node.TregLN_mAPC*(1-[CTLA4Sig_TrLN-mAPC])
171	[Treg:mAPC_IntTime]*Lymph_Node.TregLN_mAPC*([CTLA4Sig_TrLN-mAPC])
172	[Exp_CD28/80/86/PD1/L1/L2]*Lymph_Node.TregLN_mAPC1*([CD80_receptors-per-mAPC]/[Avagadro's_Num])*(1/Tr_cells_per_mAPC)
173	[Exp_CD28/80/86/PD1/L1/L2]*Lymph_Node.TregLN_mAPC1*([CD86_receptors-per-mAPC]/[Avagadro's_Num])*(1/Tr_cells_per_mAPC)
174	[Exp_CTLA4]*Lymph_Node.TregLN_mAPC1*([CTLA4_receptors-Tr]/[Avagadro's_Num])*(1/mAPC_per_Tr_cell)
175	kon_CTLA4mAb_CTLA4*Lymph_Node.TrLN_CTLA4*Lymph_Node.CTLA4_mab/Vtdln - koff_CTLA4mAb_CTLA4*Lymph_Node.TrLN_CT_aCT
176	[Exp_CTLA4]*(Lymph_Node.TrLN_CTLA4+[TrLN_CT_aCT])
177	(k__DoseAdmin_AntiCTLA4*[BodyWeight (kg)]*CTLA4mAb)/(Ipilimumab_MW)
178	([BodyWeight (kg)]*PD1mAb)/(Nivolumab_MW)/T_IVinject
179	(k__DoseAdmin_AntiPDL1*[BodyWeight (kg)]*PDL1mAb)/(Durvalumab_MW)
180	EffT_InOutLN*Lymph_Node.Naive_T0
181	Lymph_Node.Prolif_Naive_T*[Prolif_Fract]
182	kf_Monocytes_intoT*Monocytes
183	Rate_Tumor_Growth*Tumor.Cancer
184	(Peripheral.CTLA4_mabP_leaky/(0.35*ISF*KP_PD1))*0.67*Q_L*(1-SigmaL)-Lymph_Node.CTLA4_mab*0.67*Q_L*(1-SigmaL)/VL
185	(Peripheral.CTLA4_mabP_tight/(0.65*ISF*KP_PD1))*0.33*Q_L*(1-SigmaL)-Lymph_Node.CTLA4_mab*0.33*Q_L*(1-SigmaL)/VL
186	(QC_Periph - LC_Periph)*Peripheral.EffT_P_f_Vasc/Vv_Periph
187	EffT_Turnover*Peripheral.EffT_P_f_Vasc
188	(Peripheral.PD1_mabP_leaky/(0.35*ISF*KP_PD1))*0.67*Q_L*(1-SigmaL)-Lymph_Node.PD1_mab*0.67*Q_L*(1-SigmaL)/VL
189	(Peripheral.PD1_mabP_tight/(0.65*ISF*KP_PD1))*0.33*Q_L*(1-SigmaL)-Lymph_Node.PD1_mab*0.33*Q_L*(1-SigmaL)/VL
190	(Peripheral.PDL1_mabP_leaky/(0.35*ISF*KP_PDL1))*0.67*Q_L*(1-SigmaL)-Lymph_Node.PDL1_mab*0.67*Q_L*(1-SigmaL)/VL
191	(Peripheral.PDL1_mabP_tight/(0.65*ISF*KP_PDL1))*0.33*Q_L*(1-SigmaL)-Lymph_Node.PDL1_mab*0.33*Q_L*(1-SigmaL)/VL
192	[Exp_CD28/80/86/PD1/L1/L2]*Tumor.[C{CD80}]*([CD80_receptors_per_C_Cl]/[Avagadro's_Num])*([T_per_Cancer_Cell_Int]/[T_per_Cancer_Cell_max])
193	[Exp_CD28/80/86/PD1/L1/L2]*Tumor.[C{PD1}]*([PD1_receptors_per_C_Cl]/[Avagadro's_Num])*([T_per_Cancer_Cell_Int]/[T_per_Cancer_Cell_max])
194	[Exp_CD28/80/86/PD1/L1/L2]*Tumor.[C{PD1}{CD80}]*([PD1_receptors_per_C_Cl]/[Avagadro's_Num])*([T_per_Cancer_Cell_Int]/[T_per_Cancer_Cell_max])
195	[Exp_CD28/80/86/PD1/L1/L2]*Tumor.[C{PD1}{CD80}]*([CD80_receptors_per_C_Cl]/[Avagadro's_Num])*([T_per_Cancer_Cell_Int]/[T_per_Cancer_Cell_max])
196	[Exp_CD28/80/86/PD1/L1/L2]*Tumor.[C{PD1}{PDL1}]*([PD1_receptors_per_C_Cl]/[Avagadro's_Num])*([T_per_Cancer_Cell_Int]/[T_per_Cancer_Cell_max])
197	[Exp_CD28/80/86/PD1/L1/L2]*Tumor.[C{PD1}{PDL1}]*([PDL1_receptors_per_C_Cl]/[Avagadro's_Num])*([T_per_Cancer_Cell_Int]/[T_per_Cancer_Cell_max])
198	[Exp_CD28/80/86/PD1/L1/L2]*Tumor.[C{PD1}{PDL1}{CD80}]*([PD1_receptors_per_C_Cl]/[Avagadro's_Num])*([T_per_Cancer_Cell_Int]/[T_per_Cancer_Cell_max])
199	[Exp_CD28/80/86/PD1/L1/L2]*Tumor.[C{PD1}{PDL1}{CD80}]*([PDL1_receptors_per_C_Cl]/[Avagadro's_Num])*([T_per_Cancer_Cell_Int]/[T_per_Cancer_Cell_max])
200	[Exp_CD28/80/86/PD1/L1/L2]*Tumor.[C{PD1}{PDL1}{CD80}]*([CD80_receptors_per_C_Cl]/[Avagadro's_Num])*([T_per_Cancer_Cell_Int]/[T_per_Cancer_Cell_max])
201	[Exp_CD28/80/86/PD1/L1/L2]*Tumor.[C{PD1}{PDL1}{PDL2}]*([PD1_receptors_per_C_Cl]/[Avagadro's_Num])*([T_per_Cancer_Cell_Int]/[T_per_Cancer_Cell_max])
202	[Exp_CD28/80/86/PD1/L1/L2]*Tumor.[C{PD1}{PDL1}{PDL2}]*([PDL1_receptors_per_C_Cl]/[Avagadro's_Num])*([T_per_Cancer_Cell_Int]/[T_per_Cancer_Cell_max])
203	[Exp_CD28/80/86/PD1/L1/L2]*Tumor.[C{PD1}{PDL1}{PDL2}]*([PDL2_receptors_per_C_Cl]/[Avagadro's_Num])*([T_per_Cancer_Cell_Int]/[T_per_Cancer_Cell_max])
204	[Exp_CD28/80/86/PD1/L1/L2]*Tumor.[C{PD1}{PDL1}{PDL2}{CD80}]*([PD1_receptors_per_C_Cl]/[Avagadro's_Num])*([T_per_Cancer_Cell_Int]/[T_per_Cancer_Cell_max])
205	[Exp_CD28/80/86/PD1/L1/L2]*Tumor.[C{PD1}{PDL1}{PDL2}{CD80}]*([PDL1_receptors_per_C_Cl]/[Avagadro's_Num])*([T_per_Cancer_Cell_Int]/[T_per_Cancer_Cell_max])
206	[Exp_CD28/80/86/PD1/L1/L2]*Tumor.[C{PD1}{PDL1}{PDL2}{CD80}]*([PDL2_receptors_per_C_Cl]/[Avagadro's_Num])*([T_per_Cancer_Cell_Int]/[T_per_Cancer_Cell_max])

Reaction Number	Reaction Rate
**207**	[Exp_CD28/80/86/PD1/L1/L2]*Tumor.[C{PD1}{PDL1}{PDL2}{CD80}]*([CD80_receptors_per_C_Cl]/[Avagadro's_Num])*([T_per_Cancer_Cell_Int]/[T_per_Cancer_Cell_max])
**208**	[Exp_CD28/80/86/PD1/L1/L2]*Tumor.[C{PD1}{PDL2}]*([PD1_receptors_per_C_Cl]/[Avagadro's_Num])*([T_per_Cancer_Cell_Int]/[T_per_Cancer_Cell_max])
**209**	[Exp_CD28/80/86/PD1/L1/L2]*Tumor.[C{PD1}{PDL2}]*([PDL2_receptors_per_C_Cl]/[Avagadro's_Num])*([T_per_Cancer_Cell_Int]/[T_per_Cancer_Cell_max])
**210**	[Exp_CD28/80/86/PD1/L1/L2]*Tumor.[C{PD1}{PDL2}{CD80}]*([PD1_receptors_per_C_Cl]/[Avagadro's_Num])*([T_per_Cancer_Cell_Int]/[T_per_Cancer_Cell_max])
**211**	[Exp_CD28/80/86/PD1/L1/L2]*Tumor.[C{PD1}{PDL2}{CD80}]*([PDL2_receptors_per_C_Cl]/[Avagadro's_Num])*([T_per_Cancer_Cell_Int]/[T_per_Cancer_Cell_max])
**212**	[Exp_CD28/80/86/PD1/L1/L2]*Tumor.[C{PD1}{PDL2}{CD80}]*([CD80_receptors_per_C_Cl]/[Avagadro's_Num])*([T_per_Cancer_Cell_Int]/[T_per_Cancer_Cell_max])
**213**	[Exp_CD28/80/86/PD1/L1/L2]*Tumor.[C{PDL1}]*([PDL1_receptors_per_C_Cl]/[Avagadro's_Num])*([T_per_Cancer_Cell_Int]/[T_per_Cancer_Cell_max])
**214**	[Exp_CD28/80/86/PD1/L1/L2]*Tumor.[C{PDL1}{CD80}]*([PDL1_receptors_per_C_Cl]/[Avagadro's_Num])*([T_per_Cancer_Cell_Int]/[T_per_Cancer_Cell_max])
**215**	[Exp_CD28/80/86/PD1/L1/L2]*Tumor.[C{PDL1}{CD80}]*([CD80_receptors_per_C_Cl]/[Avagadro's_Num])*([T_per_Cancer_Cell_Int]/[T_per_Cancer_Cell_max])
**216**	[Exp_CD28/80/86/PD1/L1/L2]*Tumor.[C{PDL1}{PDL2}]*([PDL1_receptors_per_C_Cl]/[Avagadro's_Num])*([T_per_Cancer_Cell_Int]/[T_per_Cancer_Cell_max])
**217**	[Exp_CD28/80/86/PD1/L1/L2]*Tumor.[C{PDL1}{PDL2}]*([PDL2_receptors_per_C_Cl]/[Avagadro's_Num])*([T_per_Cancer_Cell_Int]/[T_per_Cancer_Cell_max])
**218**	[Exp_CD28/80/86/PD1/L1/L2]*Tumor.[C{PDL1}{PDL2}{CD80}]*([PDL1_receptors_per_C_Cl]/[Avagadro's_Num])*([T_per_Cancer_Cell_Int]/[T_per_Cancer_Cell_max])
**219**	[Exp_CD28/80/86/PD1/L1/L2]*Tumor.[C{PDL1}{PDL2}{CD80}]*([PDL2_receptors_per_C_Cl]/[Avagadro's_Num])*([T_per_Cancer_Cell_Int]/[T_per_Cancer_Cell_max])
**220**	[Exp_CD28/80/86/PD1/L1/L2]*Tumor.[C{PDL1}{PDL2}{CD80}]*([CD80_receptors_per_C_Cl]/[Avagadro's_Num])*([T_per_Cancer_Cell_Int]/[T_per_Cancer_Cell_max])
**221**	[Exp_CD28/80/86/PD1/L1/L2]*Tumor.[C{PDL2}]*([PDL2_receptors_per_C_Cl]/[Avagadro's_Num])*([T_per_Cancer_Cell_Int]/[T_per_Cancer_Cell_max])
**222**	[Exp_CD28/80/86/PD1/L1/L2]*Tumor.[C{PDL2}{CD80}]*([PDL2_receptors_per_C_Cl]/[Avagadro's_Num])*([T_per_Cancer_Cell_Int]/[T_per_Cancer_Cell_max])
**223**	[Exp_CD28/80/86/PD1/L1/L2]*Tumor.[C{PDL2}{CD80}]*([CD80_receptors_per_C_Cl]/[Avagadro's_Num])*([T_per_Cancer_Cell_Int]/[T_per_Cancer_Cell_max])
**224**	[kon_PD1-PD1mAb]*Tumor.[C10a=PD1]*Tumor.PD1_mabt/Vt - [koff_PD1-PD1mAb]*Tumor.[C10a=PD1:aPD1]
**225**	[Exp_CD28/80/86/PD1/L1/L2]*(Tumor.[C10a=PD1]+[C10a=PD1:aPD1]+[T10a=PDL1:PD1=C10a])
**226**	[kon_PDL1-PDL1mAb]*Tumor.[C10b=PDL1]*Tumor.PDL1_mabt/Vt - [koff_PDL1-PDL1mAb]*Tumor.[C10b=PDL1:aPDL1]
**227**	[Exp_CD28/80/86/PD1/L1/L2]*(Tumor.[C10b=PDL1]+[C10b=PDL1:aPDL1]+ [T10b=PD1:PDL1=C10b]+ [T10c=CD80:PDL1=C10b])
**228**	[Exp_CD28/80/86/PD1/L1/L2]*(Tumor.[C10c=PDL2] + [T10b=PD1:PDL2=C10c])
**229**	[kon_PDL1-PDL1mAb]*Tumor.[C11a=PDL1]*Tumor.PDL1_mabt/Vt - [koff_PDL1-PDL1mAb]*Tumor.[C11a=PDL1:aPDL1]
**230**	[Exp_CD28/80/86/PD1/L1/L2]*(Tumor.[C11a=PDL1]+[C11a=PDL1:aPDL1]+ [T11b=PD1:PDL1=C11a])
**231**	[Exp_CD28/80/86/PD1/L1/L2]*(Tumor.[C11b=CD80]+ [T11a=PDL1:CD80=C11b])
**232**	[Exp_CD28/80/86/PD1/L1/L2]*(Tumor.[C12a=PDL2]+ [T12b=PD1:PDL2=C12a])
**233**	[Exp_CD28/80/86/PD1/L1/L2]*(Tumor.[C12b=CD80]+ [T12a=PDL1:CD80=C12b])
**234**	[kon_PDL1-PDL1mAb]*Tumor.[C13a=PDL1]*Tumor.PDL1_mabt/Vt - [koff_PDL1-PDL1mAb]*Tumor.[C13a=PDL1:aPDL1]
**235**	[Exp_CD28/80/86/PD1/L1/L2]*(Tumor.[C13a=PDL1]+[C13a=PDL1:aPDL1]+ [T13b=PD1:PDL1=C13a])
**236**	[Exp_CD28/80/86/PD1/L1/L2]*(Tumor.[C13b=PDL2]+ [T13b=PD1:PDL2=C13b])
**237**	[Exp_CD28/80/86/PD1/L1/L2]*(Tumor.[C13c=CD80]+ [T13a=PDL1:CD80=C13c])
**238**	[kon_PD1_PDL1]*Tumor.[C14a=PD1]*Tumor.[T14a=PDL1]/[V_T:C_14] - [koff_PD1_PDL1]*Tumor.[T14a=PDL1:PD1=C14a]
**239**	[Exp_CD28/80/86/PD1/L1/L2]*(Tumor.[C14a=PD1]+[C14a=PD1:aPD1]+ [T14a=PDL1:PD1=C14a])
**240**	[kon_PDL1_CD80]*Tumor.[C14b=PDL1]*Tumor.[T14c=CD80]/[V_T:C_14] - [koff_PDL1_CD80]*Tumor.[T14c=CD80:PDL1=C14b]
**241**	[kon_PDL1-PDL1mAb]*Tumor.[C14b=PDL1]*Tumor.PDL1_mabt/Vt - [koff_PDL1-PDL1mAb]*Tumor.[C14b=PDL1:aPDL1]
**242**	[Exp_CD28/80/86/PD1/L1/L2]*(Tumor.[C14b=PDL1]+[C14b=PDL1:aPDL1]+ [T14b=PD1:PDL1=C14b]+ [T14c=CD80:PDL1=C14b])
**243**	[kon_PDL1_CD80]*Tumor.[C14c=CD80]*Tumor.[T14a=PDL1]/[V_T:C_14] - [koff_PDL1_CD80]*Tumor.[T14a=PDL1:CD80=C14c]
**244**	[Exp_CD28/80/86/PD1/L1/L2]*(Tumor.[C14c=CD80]+ [T14a=PDL1:CD80=C14c])
**245**	[kon_PD1-PD1mAb]*Tumor.[C15a=PD1]*Tumor.PD1_mabt/Vt - [koff_PD1-PD1mAb]*Tumor.[C15a=PD1:aPD1]
**246**	[Exp_CD28/80/86/PD1/L1/L2]*(Tumor.[C15a=PD1]+[C15a=PD1:aPD1]+ [T15a=PDL1:PD1=C15a])
**247**	[kon_PD1_PDL2]*Tumor.[C15b=PDL2]*Tumor.[T15b=PD1]/[V_T:C_15] - [koff_PD1_PDL2]*Tumor.[T15b=PD1:PDL2=C15b]

Reaction Number	Reaction Rate
**248**	[Exp_CD28/80/86/PD1/L1/L2]*(Tumor.[C15b=PDL2] + [T15b=PD1:PDL2=C15b])
**249**	[kon_PDL1_CD80]*Tumor.[C15c=CD80]*Tumor.[T15a=PDL1]/[V_T:C_15] - [koff_PDL1_CD80]*Tumor.[T15a=PDL1:CD80=C15c]
**250**	[Exp_CD28/80/86/PD1/L1/L2]*(Tumor.[C15c=CD80]+ [T15a=PDL1:CD80=C15c])
**251**	[kon_PD1_PDL1]*Tumor.[C16a=PD1]*Tumor.[T16a=PDL1]/[V_T:C_16] - [koff_PD1_PDL1]*Tumor.[T16a=PDL1:PD1=C16a]
**252**	[kon_PD1-PD1mAb]*Tumor.[C16a=PD1]*Tumor.PD1_mabt/Vt - [koff_PD1-PD1mAb]*Tumor.[C16a=PD1:aPD1]
**253**	[Exp_CD28/80/86/PD1/L1/L2]*(Tumor.[C16a=PD1]+[C16a=PD1:aPD1]+ [T16a=PDL1:PD1=C16a])
**254**	[kon_PDL1-PDL1mAb]*Tumor.[C16b=PDL1]*Tumor.PDL1_mabt/Vt-[koff_PDL1-PDL1mAb]*Tumor.[C16b=PDL1:aPDL1]
**255**	[Exp_CD28/80/86/PD1/L1/L2]*(Tumor.[C16b=PDL1]+[C16b=PDL1:aPDL1]+ [T16c=CD80:PDL1=C16b]+ [T16b=PD1:PDL1=C16b])
**256**	[kon_PD1_PDL2]*Tumor.[C16c=PDL2]*Tumor.[T16b=PD1]/[V_T:C_16] - [koff_PD1_PDL2]*Tumor.[T16b=PD1:PDL2=C16c]
**257**	[Exp_CD28/80/86/PD1/L1/L2]*(Tumor.[C16c=PDL2]+ [T16b=PD1:PDL2=C16c])
**258**	[Exp_CD28/80/86/PD1/L1/L2]*(Tumor.[C16d=CD80]+ [T16a=PDL1:CD80=C16d])
**259**	[Exp_CD28/80/86/PD1/L1/L2]*(Tumor.[C2=PD1]+[C2=PD1:aPD1]+[T2=PDL1:PD1=C2])
**260**	[Exp_CD28/80/86/PD1/L1/L2]*(Tumor.[C3=PDL1]+[C3=PDL1:aPDL1]+[T3a=PD1:PDL1=C3]+[T3b=CD80:PDL1=C3])
**261**	[Exp_CD28/80/86/PD1/L1/L2]*(Tumor.[C4=PDL2]+[T4=PD1:PDL2=C4])
**262**	[Exp_CD28/80/86/PD1/L1/L2]*(Tumor.[C5=CD80]+[T5=PDL1:CD80=C5])
**263**	[kon_PD1-PD1mAb]*Tumor.[C6a=PD1]*Tumor.PD1_mabt/Vt - [koff_PD1-PD1mAb]*Tumor.[C6a=PD1:aPD1]
**264**	[Exp_CD28/80/86/PD1/L1/L2]*(Tumor.[C6a=PD1]+[C6a=PD1:aPD1]+[T6a=PDL1:PD1=C6a])
**265**	[kon_PD1_PDL1]*Tumor.[C6b=PDL1]*Tumor.[T6b=PD1]/[V_T:C_6] - [koff_PD1_PDL1]*Tumor.[T6b=PD1:PDL1=C6b]
**266**	[kon_PDL1_CD80]*Tumor.[C6b=PDL1]*Tumor.[T6c=CD80]/[V_T:C_6] - [koff_PDL1_CD80]*Tumor.[T6c=CD80:PDL1=C6b]
**267**	[kon_PDL1-PDL1mAb]*Tumor.[C6b=PDL1]*Tumor.PDL1_mabt/Vt - [koff_PDL1-PDL1mAb]*Tumor.[C6b=PDL1:aPDL1]
**268**	[Exp_CD28/80/86/PD1/L1/L2]*(Tumor.[C6b=PDL1]+[C6b=PDL1:aPDL1]+[T6b=PD1:PDL1=C6b]+[T6c=CD80:PDL1=C6b])
**269**	[Exp_CD28/80/86/PD1/L1/L2]*(Tumor.[C7a=PD1]+[C7a=PD1:aPD1]+[T7a=PDL1:PD1=C7a])
**270**	[Exp_CD28/80/86/PD1/L1/L2]*(Tumor.[C7b=PDL2]+[T7b=PD1:PDL2=C7b])
**271**	[kon_PDL1_CD80]*Tumor.[C8a=PDL1]*Tumor.[T8b=CD80]/[V_T:C_8] - [koff_PDL1_CD80]*Tumor.[T8b=CD80:PDL1=C8a]
**272**	[kon_PDL1-PDL1mAb]*Tumor.[C8a=PDL1]*Tumor.PDL1_mabt/Vt - [koff_PDL1-PDL1mAb]*Tumor.[C8a=PDL1:aPDL1]
**273**	[Exp_CD28/80/86/PD1/L1/L2]*(Tumor.[C8a=PDL1]+[C8a=PDL1:aPDL1]+[T8a=PD1:PDL1=C8a]+[T8b=CD80:PDL1=C8a])
**274**	[Exp_CD28/80/86/PD1/L1/L2]*(Tumor.[C8b=PDL2]+[T8a=PD1:PDL2=C8b])
**275**	[kon_PD1_PDL1]*Tumor.[C9a=PD1]*Tumor.[T9=PDL1]/[V_T:C_9] - [koff_PD1_PDL1]*Tumor.[T9=PDL1-PD1=C9a]
**276**	[Exp_CD28/80/86/PD1/L1/L2]*(Tumor.[C9a=PD1]+[C9a=PD1:aPD1]+[T9=PDL1-PD1=C9a])
**277**	[Exp_CD28/80/86/PD1/L1/L2]*(Tumor.[C9b=CD80]+[T9=PDL1-CD80=C9b])
**278**	Endo_CTLA4*Tumor.[CTLA4:CD80_TrAT]
**279**	Endo_CTLA4*Tumor.[CTLA4:CD86_TrAT]
**280**	Endo_CTLA4*Tumor.[CTLA4_CTLA4-Trt]
**281**	[Exp_CD28/80/86/PD1/L1/L2]*Tumor.[T{PD1}{80}-{PDL1}{PDL2}C]*([PD1_receptors-per-Tcell]/[Avagadro's_Num])*([Cancer_per_T_Cell_Int]/[Cancer_per_T_Cell_max])
**282**	[Exp_CD28/80/86/PD1/L1/L2]*Tumor.[T{PD1}{80}-{PDL1}{PDL2}C]*([CD80_receptors-per-Tcell]/[Avagadro's_Num])*([Cancer_per_T_Cell_Int]/[Cancer_per_T_Cell_max])
**283**	[Exp_CD28/80/86/PD1/L1/L2]*Tumor.[T{PD1}{CD80}-{PDL1}C]*([PD1_receptors-per-Tcell]/[Avagadro's_Num])*([Cancer_per_T_Cell_Int]/[Cancer_per_T_Cell_max])
**284**	[Exp_CD28/80/86/PD1/L1/L2]*Tumor.[T{PD1}{CD80}-{PDL1}C]*([CD80_receptors-per-Tcell]/[Avagadro's_Num])*([Cancer_per_T_Cell_Int]/[Cancer_per_T_Cell_max])
**285**	[Exp_CD28/80/86/PD1/L1/L2]*Tumor.[T{PD1}{L1}{80}-{PD1}{80}{L1}{L2}C]*([PDL1_receptors-per-Tcell]/[Avagadro's_Num])*([Cancer_per_T_Cell_Int]/[Cancer_per_T_Cell_max])
**286**	[Exp_CD28/80/86/PD1/L1/L2]*Tumor.[T{PD1}{L1}{80}-{PD1}{80}{L1}{L2}C]*([PD1_receptors-per-Tcell]/[Avagadro's_Num])*([Cancer_per_T_Cell_Int]/[Cancer_per_T_Cell_max])
**287**	[Exp_CD28/80/86/PD1/L1/L2]*Tumor.[T{PD1}{L1}{80}-{PD1}{80}{L1}{L2}C]*([CD80_receptors-per-Tcell]/[Avagadro's_Num])*([Cancer_per_T_Cell_Int]/[Cancer_per_T_Cell_max])
**288**	[Exp_CD28/80/86/PD1/L1/L2]*Tumor.[T{PD1}{L1}{80}-{PD1}{80}{L1}C]*([PDL1_receptors-per-Tcell]/[Avagadro's_Num])*([Cancer_per_T_Cell_Int]/[Cancer_per_T_Cell_max])
**289**	[Exp_CD28/80/86/PD1/L1/L2]*Tumor.[T{PD1}{L1}{80}-{PD1}{80}{L1}C]*([PD1_receptors-per-Tcell]/[Avagadro's_Num])*([Cancer_per_T_Cell_Int]/[Cancer_per_T_Cell_max])
**290**	[Exp_CD28/80/86/PD1/L1/L2]*Tumor.[T{PD1}{L1}{80}-{PD1}{80}{L1}C]*([CD80_receptors-per-Tcell]/[Avagadro's_Num])*([Cancer_per_T_Cell_Int]/[Cancer_per_T_Cell_max])
**291**	[Exp_CD28/80/86/PD1/L1/L2]*Tumor.[T{PD1}{L1}{80}-{PD1}{L1}{L2}C]*([PDL1_receptors-per-Tcell]/[Avagadro's_Num])*([Cancer_per_T_Cell_Int]/[Cancer_per_T_Cell_max])
**292**	[Exp_CD28/80/86/PD1/L1/L2]*Tumor.[T{PD1}{L1}{80}-{PD1}{L1}{L2}C]*([PD1_receptors-per-Tcell]/[Avagadro's_Num])*([Cancer_per_T_Cell_Int]/[Cancer_per_T_Cell_max])
**293**	[Exp_CD28/80/86/PD1/L1/L2]*Tumor.[T{PD1}{L1}{80}-{PD1}{L1}{L2}C]*([CD80_receptors-per-Tcell]/[Avagadro's_Num])*([Cancer_per_T_Cell_Int]/[Cancer_per_T_Cell_max])
**294**	[Exp_CD28/80/86/PD1/L1/L2]*Tumor.[T{PD1}{L1}{80}-{PD1}{L1}C]*([PDL1_receptors-per-Tcell]/[Avagadro's_Num])*([Cancer_per_T_Cell_Int]/[Cancer_per_T_Cell_max])

Reaction Number	Reaction Rate
**295**	[Exp_CD28/80/86/PD1/L1/L2]*Tumor.[T{PD1}{L1}{80}-{PD1}{L1}C]*([PD1_receptors-per-Tcell]/[Avagadro's_Num])*([Cancer_per_T_Cell_Int]/[Cancer_per_T_Cell_max])
**296**	[Exp_CD28/80/86/PD1/L1/L2]*Tumor.[T{PD1}{L1}{80}-{PD1}{L1}C]*([CD80_receptors-per-Tcell]/[Avagadro's_Num])*([Cancer_per_T_Cell_Int]/[Cancer_per_T_Cell_max])
**297**	[Exp_CD28/80/86/PD1/L1/L2]*Tumor.[T{PD1}{L1}-{PD1}{80}{PDL2}C]*([PDL1_receptors-per-Tcell]/[Avagadro's_Num])*([Cancer_per_T_Cell_Int]/[Cancer_per_T_Cell_max])
**298**	[Exp_CD28/80/86/PD1/L1/L2]*Tumor.[T{PD1}{L1}-{PD1}{80}{PDL2}C]*([PD1_receptors-per-Tcell]/[Avagadro's_Num])*([Cancer_per_T_Cell_Int]/[Cancer_per_T_Cell_max])
**299**	[Exp_CD28/80/86/PD1/L1/L2]*Tumor.[T{PD1}{L1}-{PD1}{L2}C]*([PDL1_receptors-per-Tcell]/[Avagadro's_Num])*([Cancer_per_T_Cell_Int]/[Cancer_per_T_Cell_max])
**300**	[Exp_CD28/80/86/PD1/L1/L2]*Tumor.[T{PD1}{L1}-{PD1}{L2}C]*([PD1_receptors-per-Tcell]/[Avagadro's_Num])*([Cancer_per_T_Cell_Int]/[Cancer_per_T_Cell_max])
**301**	[Exp_CD28/80/86/PD1/L1/L2]*Tumor.[T{PD1}{L1}-{PDL1}{80}{L2}C]*([PDL1_receptors-per-Tcell]/[Avagadro's_Num])*([Cancer_per_T_Cell_Int]/[Cancer_per_T_Cell_max])
**302**	[Exp_CD28/80/86/PD1/L1/L2]*Tumor.[T{PD1}{L1}-{PDL1}{80}{L2}C]*([PD1_receptors-per-Tcell]/[Avagadro's_Num])*([Cancer_per_T_Cell_Int]/[Cancer_per_T_Cell_max])
**303**	[Exp_CD28/80/86/PD1/L1/L2]*Tumor.[T{PD1}{L1}-{PDL1}{80}C]*([PDL1_receptors-per-Tcell]/[Avagadro's_Num])*([Cancer_per_T_Cell_Int]/[Cancer_per_T_Cell_max])
**304**	[Exp_CD28/80/86/PD1/L1/L2]*Tumor.[T{PD1}{L1}-{PDL1}{80}C]*([PD1_receptors-per-Tcell]/[Avagadro's_Num])*([Cancer_per_T_Cell_Int]/[Cancer_per_T_Cell_max])
**305**	[Exp_CD28/80/86/PD1/L1/L2]*Tumor.[T{PD1}{L1}-{PDL2}{80}C]*([PDL1_receptors-per-Tcell]/[Avagadro's_Num])*([Cancer_per_T_Cell_Int]/[Cancer_per_T_Cell_max])
**306**	[Exp_CD28/80/86/PD1/L1/L2]*Tumor.[T{PD1}{L1}-{PDL2}{80}C]*([PD1_receptors-per-Tcell]/[Avagadro's_Num])*([Cancer_per_T_Cell_Int]/[Cancer_per_T_Cell_max])
**307**	[Exp_CD28/80/86/PD1/L1/L2]*Tumor.[T{PD1}-{PDL2}C]*([PD1_receptors-per-Tcell]/[Avagadro's_Num])*([Cancer_per_T_Cell_Int]/[Cancer_per_T_Cell_max])
**308**	[Exp_CD28/80/86/PD1/L1/L2]*Tumor.[T{PDL1}-{CD80}{PD1}C]*([PDL1_receptors-per-Tcell]/[Avagadro's_Num])*([Cancer_per_T_Cell_Int]/[Cancer_per_T_Cell_max])
**309**	[Exp_CD28/80/86/PD1/L1/L2]*Tumor.[T{PDL1}-{CD80}C]*([PDL1_receptors-per-Tcell]/[Avagadro's_Num])*([Cancer_per_T_Cell_Int]/[Cancer_per_T_Cell_max])
**310**	[Exp_CD28/80/86/PD1/L1/L2]*Tumor.[T{PDL1}-{PD1}C]*([PDL1_receptors-per-Tcell]/[Avagadro's_Num])*([Cancer_per_T_Cell_Int]/[Cancer_per_T_Cell_max])
**311**	[kon_PD1_PDL1]*Tumor.[T10a=PDL1]*Tumor.[C10a=PD1]/[V_T:C_10] - [koff_PD1_PDL1]*Tumor.[T10a=PDL1:PD1=C10a]
**312**	[kon_PDL1-PDL1mAb]*Tumor.[T10a=PDL1]*Tumor.PDL1_mabt/Vt-[koff_PDL1-PDL1mAb]*Tumor.[T10a=PDL1:aPDL1]
**313**	[Exp_CD28/80/86/PD1/L1/L2]*(Tumor.[T10a=PDL1]+[T10a=PDL1:aPDL1]+[T10a=PDL1:PD1=C10a])
**314**	[kon_PD1_PDL1]*Tumor.[T10b=PD1]*Tumor.[C10b=PDL1]/[V_T:C_10] - [koff_PD1_PDL1]*Tumor.[T10b=PD1:PDL1=C10b]
**315**	[kon_PD1_PDL2]*Tumor.[T10b=PD1]*Tumor.[C10c=PDL2]/[V_T:C_10] - [koff_PD1_PDL2]*Tumor.[T10b=PD1:PDL2=C10c]
**316**	[Exp_CD28/80/86/PD1/L1/L2]*(Tumor.[T10b=PD1]+[T10b=PD1:aPD1]+ [T10b=PD1:PDL1=C10b]+ [T10b=PD1:PDL2=C10c])
**317**	[kon_PDL1_CD80]*Tumor.[T10c=CD80]*Tumor.[C10b=PDL1]/[V_T:C_10] - [koff_PDL1_CD80]*Tumor.[T10c=CD80:PDL1=C10b]
**318**	[Exp_CD28/80/86/PD1/L1/L2]*(Tumor.[T10c=CD80]+ [T10c=CD80:PDL1=C10b])
**319**	[kon_PDL1_CD80]*Tumor.[T11a=PDL1]*Tumor.[C11b=CD80]/[V_T:C_11] - [koff_PDL1_CD80]*Tumor.[T11a=PDL1:CD80=C11b]
**320**	[kon_PDL1-PDL1mAb]*Tumor.[T11a=PDL1]*Tumor.PDL1_mabt/Vt - [koff_PDL1-PDL1mAb]*Tumor.[T11a=PDL1:aPDL1]
**321**	[Exp_CD28/80/86/PD1/L1/L2]*(Tumor.[T11a=PDL1]+[T11a=PDL1:aPDL1]+ [T11a=PDL1:CD80=C11b])
**322**	[kon_PD1_PDL1]*Tumor.[T11b=PD1]*Tumor.[C11a=PDL1]/[V_T:C_11] - [koff_PD1_PDL1]*Tumor.[T11b=PD1:PDL1=C11a]
**323**	[Exp_CD28/80/86/PD1/L1/L2]*(Tumor.[T11b=PD1]+[T11b=PD1:aPD1]+ [T11b=PD1:PDL1=C11a])
**324**	[kon_PDL1_CD80]*Tumor.[T12a=PDL1]*Tumor.[C12b=CD80]/[V_T:C_12] - [koff_PDL1_CD80]*Tumor.[T12a=PDL1:CD80=C12b]
**325**	[kon_PDL1-PDL1mAb]*Tumor.[T12a=PDL1]*Tumor.PDL1_mabt/Vt - [koff_PDL1-PDL1mAb]*Tumor.[T12a=PDL1:aPDL1]
**326**	[Exp_CD28/80/86/PD1/L1/L2]*(Tumor.[T12a=PDL1]+[T12a=PDL1:aPDL1]+ [T12a=PDL1:CD80=C12b])
**327**	[kon_PD1_PDL2]*Tumor.[T12b=PD1]*Tumor.[C12a=PDL2]/[V_T:C_12] - [koff_PD1_PDL2]*Tumor.[T12b=PD1:PDL2=C12a]
**328**	[Exp_CD28/80/86/PD1/L1/L2]*(Tumor.[T12b=PD1]+[T12b=PD1:aPD1]+ [T12b=PD1:PDL2=C12a])
**329**	[kon_PDL1_CD80]*Tumor.[T13a=PDL1]*Tumor.[C13c=CD80]/[V_T:C_13] - [koff_PDL1_CD80]*Tumor.[T13a=PDL1:CD80=C13c]
**330**	[Exp_CD28/80/86/PD1/L1/L2]*(Tumor.[T13a=PDL1]+[T13a=PDL1:aPDL1]+ [T13a=PDL1:CD80=C13c])
**331**	[kon_PD1_PDL1]*Tumor.[T13b=PD1]*Tumor.[C13a=PDL1]/[V_T:C_13] - [koff_PD1_PDL1]*Tumor.[T13b=PD1:PDL1=C13a]
**332**	[kon_PD1_PDL2]*Tumor.[T13b=PD1]*Tumor.[C13b=PDL2]/[V_T:C_13] - [koff_PD1_PDL2]*Tumor.[T13b=PD1:PDL2=C13b]

Reaction Number	Reaction Rate
**333**	[kon_PD1-PD1mAb]*Tumor.[T13b=PD1]*Tumor.PD1_mabt/Vt-[koff_PD1-PD1mAb]*Tumor.[T13b=PD1:aPD1]
**334**	[Exp_CD28/80/86/PD1/L1/L2]*(Tumor.[T13b=PD1]+[T13b=PD1:aPD1]+ [T13b=PD1:PDL2=C13b]+ [T13b=PD1:PDL1=C13a])
**335**	[kon_PDL1-PDL1mAb]*Tumor.[T14a=PDL1]*Tumor.PDL1_mabt/Vt - [koff_PDL1-PDL1mAb]*Tumor.[T14a=PDL1:aPDL1]
**336**	[Exp_CD28/80/86/PD1/L1/L2]*(Tumor.[T14a=PDL1]+[T14a=PDL1:aPDL1]+ [T14a=PDL1:PD1=C14a]+ [T14a=PDL1:CD80=C14c])
**337**	[kon_PD1_PDL1]*Tumor.[T14b=PD1]*Tumor.[C14b=PDL1]/[V_T:C_14] - [koff_PD1_PDL1]*Tumor.[T14b=PD1:PDL1=C14b]
**338**	[Exp_CD28/80/86/PD1/L1/L2]*(Tumor.[T14b=PD1]+[T14b=PD1:aPD1]+ [T14b=PD1:PDL1=C14b])
**339**	[Exp_CD28/80/86/PD1/L1/L2]*(Tumor.[T14c=CD80]+ [T14c=CD80:PDL1=C14b])
**340**	[kon_PD1_PDL1]*Tumor.[T15a=PDL1]*Tumor.[C15a=PD1]/[V_T:C_15] - [koff_PD1_PDL1]*Tumor.[T15a=PDL1:PD1=C15a]
**341**	[kon_PDL1-PDL1mAb]*Tumor.[T15a=PDL1]*Tumor.PDL1_mabt/Vt -[koff_PDL1-PDL1mAb]*Tumor.[T15a=PDL1:aPDL1]
**342**	[Exp_CD28/80/86/PD1/L1/L2]*(Tumor.[T15a=PDL1]+[T15a=PDL1:aPDL1]+ [T15a=PDL1:CD80=C15c]+ [T15a=PDL1:PD1=C15a])
**343**	[kon_PD1-PD1mAb]*Tumor.[T15b=PD1]*Tumor.PD1_mabt/Vt - [koff_PD1-PD1mAb]*Tumor.[T15b=PD1:aPD1]
**344**	[Exp_CD28/80/86/PD1/L1/L2]*(Tumor.[T15b=PD1]+[T15b=PD1:aPD1]+ [T15b=PD1:PDL2=C15b])
**345**	[kon_PDL1_CD80]*Tumor.[T16a=PDL1]*Tumor.[C16d=CD80]/[V_T:C_16] - [koff_PDL1_CD80]*Tumor.[T16a=PDL1:CD80=C16d]
**346**	[kon_PDL1-PDL1mAb]*Tumor.[T16a=PDL1]*Tumor.PDL1_mabt/Vt - [koff_PDL1-PDL1mAb]*Tumor.[T16a=PDL1:aPDL1]
**347**	[Exp_CD28/80/86/PD1/L1/L2]*(Tumor.[T16a=PDL1]+[T16a=PDL1:aPDL1]+ [T16a=PDL1:PD1=C16a]+ [T16a=PDL1:CD80=C16d])
**348**	[kon_PD1_PDL1]*Tumor.[T16b=PD1]*Tumor.[C16b=PDL1]/[V_T:C_16] - [koff_PD1_PDL1]*Tumor.[T16b=PD1:PDL1=C16b]
**349**	[kon_PD1-PD1mAb]*Tumor.[T16b=PD1]*Tumor.PD1_mabt/Vt - [koff_PD1-PD1mAb]*Tumor.[T16b=PD1:aPD1]
**350**	[Exp_CD28/80/86/PD1/L1/L2]*(Tumor.[T16b=PD1]+[T16b=PD1:aPD1]+ [T16b=PD1:PDL2=C16c]+ [T16b=PD1:PDL1=C16b])
**351**	[kon_PDL1_CD80]*Tumor.[T16c=CD80]*Tumor.[C16b=PDL1]/[V_T:C_16] - [koff_PDL1_CD80]*Tumor.[T16c=CD80:PDL1=C16b]
**352**	[Exp_CD28/80/86/PD1/L1/L2]*(Tumor.[T16c=CD80]+ [T16c=CD80:PDL1=C16b])
**353**	[kon_PD1_PDL1]*Tumor.[T2=PDL1]*Tumor.[C2=PD1]/[V_T:C_2] - [koff_PD1_PDL1]*Tumor.[T2=PDL1:PD1=C2]
**354**	[Exp_CD28/80/86/PD1/L1/L2]*(Tumor.[T2=PDL1]+[T2=PDL1:aPDL1]+[T2=PDL1:PD1=C2])
**355**	[kon_PD1_PDL1]*Tumor.[T3a=PD1]*Tumor.[C3=PDL1]/[V_T:C_3] - [koff_PD1_PDL1]*Tumor.[T3a=PD1:PDL1=C3]
**356**	[kon_PD1-PD1mAb]*Tumor.[T3a=PD1]*Tumor.PD1_mabt/Vt - [koff_PD1-PD1mAb]*Tumor.[T3a=PD1:aPD1]
**357**	[Exp_CD28/80/86/PD1/L1/L2]*(Tumor.[T3a=PD1]+[T3a=PD1:aPD1]+[T3a=PD1:PDL1=C3])
**358**	[kon_PDL1_CD80]*Tumor.[T3b=CD80]*Tumor.[C3=PDL1]/[V_T:C_3] - [koff_PDL1_CD80]*Tumor.[T3b=CD80:PDL1=C3]
**359**	[Exp_CD28/80/86/PD1/L1/L2]*(Tumor.[T3b=CD80]+[T3b=CD80:PDL1=C3])
**360**	[kon_PD1_PDL2]*Tumor.[T4=PD1]*Tumor.[C4=PDL2]/[V_T:C_4] - [koff_PD1_PDL2]*Tumor.[T4=PD1:PDL2=C4]
**361**	[kon_PD1-PD1mAb]*Tumor.[T4=PD1]*Tumor.PD1_mabt/Vt - [koff_PD1-PD1mAb]*Tumor.[T4=PD1:aPD1]
**362**	[Exp_CD28/80/86/PD1/L1/L2]*(Tumor.[T4=PD1]+[T4=PD1:aPD1]+[T4=PD1:PDL2=C4])
**363**	[kon_PDL1_CD80]*Tumor.[T5=PDL1]*Tumor.[C5=CD80]/[V_T:C_5] - [koff_PDL1_CD80]*Tumor.[T5=PDL1:CD80=C5]
**364**	[kon_PDL1-PDL1mAb]*Tumor.[T5=PDL1]*Tumor.PDL1_mabt/Vt - [koff_PDL1-PDL1mAb]*Tumor.[T5=PDL1:aPDL1]
**365**	[Exp_CD28/80/86/PD1/L1/L2]*(Tumor.[T5=PDL1]+[T5=PDL1:aPDL1]+[T5=PDL1:CD80=C5])
**366**	[kon_PD1_PDL1]*Tumor.[T6a=PDL1]*Tumor.[C6a=PD1]/[V_T:C_6] - [koff_PD1_PDL1]*Tumor.[T6a=PDL1:PD1=C6a]
**367**	[kon_PDL1-PDL1mAb]*Tumor.[T6a=PDL1]*Tumor.PDL1_mabt/Vt - [koff_PDL1-PDL1mAb]*Tumor.[T6a=PDL1:aPDL1]
**368**	[Exp_CD28/80/86/PD1/L1/L2]*(Tumor.[T6a=PDL1]+[T6a=PDL1:aPDL1]+[T6a=PDL1:PD1=C6a])
**369**	[kon_PD1-PD1mAb]*Tumor.[T6b=PD1]*Tumor.PD1_mabt/Vt - [koff_PD1-PD1mAb]*Tumor.[T6b=PD1:aPD1]
**370**	[Exp_CD28/80/86/PD1/L1/L2]*(Tumor.[T6b=PD1]+[T6b=PD1:aPD1]+[T6b=PD1:PDL1=C6b])
**371**	[Exp_CD28/80/86/PD1/L1/L2]*(Tumor.[T6c=CD80]+[T6c=CD80:PDL1=C6b])
**372**	[kon_PD1_PDL1]*Tumor.[T7a=PDL1]*Tumor.[C7a=PD1]/[V_T:C_7] - [koff_PD1_PDL1]*Tumor.[T7a=PDL1:PD1=C7a]
**373**	[Exp_CD28/80/86/PD1/L1/L2]*(Tumor.[T7a=PDL1]+[T7a=PDL1:aPDL1]+[T7a=PDL1:PD1=C7a])
**374**	[kon_PD1_PDL2]*Tumor.[T7b=PD1]*Tumor.[C7b=PDL2]/[V_T:C_7] - [koff_PD1_PDL2]*Tumor.[T7b=PD1:PDL2=C7b]
**375**	[Exp_CD28/80/86/PD1/L1/L2]*(Tumor.[T7b=PD1]+[T7b=PD1:aPD1]+[T7b=PD1:PDL2=C7b])
**376**	[kon_PD1_PDL1]*Tumor.[T8a=PD1]*Tumor.[C8a=PDL1]/[V_T:C_8] - [koff_PD1_PDL1]*Tumor.[T8a=PD1:PDL1=C8a]
**377**	[kon_PD1_PDL2]*Tumor.[T8a=PD1]*Tumor.[C8b=PDL2]/[V_T:C_8] - [koff_PD1_PDL2]*Tumor.[T8a=PD1:PDL2=C8b]
**378**	[Exp_CD28/80/86/PD1/L1/L2]*(Tumor.[T8a=PD1]+[T8a=PD1:aPD1]+[T8a=PD1:PDL1=C8a]+[T8a=PD1:PDL2=C8b])
**379**	[Exp_CD28/80/86/PD1/L1/L2]*(Tumor.[T8b=CD80]+[T8b=CD80:PDL1=C8a])
**380**	[kon_PDL1_CD80]*Tumor.[T9=PDL1]*Tumor.[C9b=CD80]/[V_T:C_9] - [koff_PDL1_CD80]*Tumor.[T9=PDL1-CD80=C9b]
**381**	[kon_PDL1-PDL1mAb]*Tumor.[T9=PDL1]*Tumor.PDL1_mabt/Vt - [koff_PDL1-PDL1mAb]*Tumor.[T9=PDL1:aPDL1]
**382**	[Exp_CD28/80/86/PD1/L1/L2]*(Tumor.[T9=PDL1]+[T9=PDL1:aPDL1]+[T9=PDL1-PD1=C9a]+[T9=PDL1-CD80=C9b])
**383**	kf_RestingMacrophage*Tumor.APC_T
**384**	Phago_Debris*Tumor.C_DebrisT*Tumor.APC_T
**385**	Debris_Transport*Tumor.C_DebrisT*(1/[Num_TDLN_Considered])*(1-[mAPC_Debis_T_Inact])
**386**	Debris_Decay*Tumor.C_DebrisT

Reaction Number	Reaction Rate
387	Debris_Transport*Tumor.C_DebrisT*[mAPC_Debis_T_Inact]
388	Debris_Transport*Tumor.C_DebrisT*(1-1/[Num_TDLN_Considered])
389	kf_CanDecay*Tumor.Cancer
390	[Exp_CD28/80/86/PD1/L1/L2]*(Tumor.CD80_mAPCT+[CTLA4:CD80_TrAT])
391	[kon_PDL1_CD80]*Tumor.CD80_TeffT*Tumor.PDL1_TregT/[Vol_Cell-Rec_Tr-TeffT] - [koff_PDL1_CD80]*Tumor.[PDL1:CD80_TrTeff]
392	[Exp_CD28/80/86/PD1/L1/L2]*(Tumor.CD80_TeffT+[PDL1:CD80_TrTeff])
393	[Exp_CD28/80/86/PD1/L1/L2]*(Tumor.CD80_TeffT1+[PDL1:CD80_MDSCT])
394	[Exp_CD28/80/86/PD1/L1/L2]*(Tumor.CD86_mAPCT+[CTLA4:CD86_TrAT])
395	Kpa_TB*S_TB*Vt_avg_const*(1.43/1.63)*Tumor.CTLA4_mabt/(Vt_Teff*Tumor_Void_Fraction)- Kpa_TB*S_TB*Vt_avg_const*(1.43/1.63)*Lymph_Node.CTLA4_mab/(VL)
396	[kon_CTLA4_CD80]*Tumor.CTLA4_TregT*Tumor.CD80_mAPCT/[Vol_Cell-Rec_Tr-APCT] - [koff_CTLA4_CD80]*Tumor.[CTLA4:CD80_TrAT]
397	[kon_CTLA4_CD86]*Tumor.CTLA4_TregT*Tumor.CD86_mAPCT/[Vol_Cell-Rec_Tr-APCT] - [koff_CTLA4_CD86]*Tumor.[CTLA4:CD86_TrAT]
398	kon_CTLA4mAb_CTLA4*Tumor.CTLA4_TregT*Tumor.CTLA4_mabt/Vt - koff_CTLA4mAb_CTLA4*Tumor.[CTLA4_CTLA4-Trt]
399	[Exp_CTLA4]*(Tumor.CTLA4_TregT+[CTLA4:CD80_TrAT]+[CTLA4:CD86_TrAT]+[CTLA4_CTLA4-Trt])
400	EffT_Turnover*Tumor.Effector_TT
401	EffT_Res_Conversion*Tumor.Effector_TT*(1-TheEnd)*.9
402	EffT_Res_Conversion*Tumor.Effector_TT*(1-TheEnd)*.1
403	CancerTEng * Tumor.Cancer1 * ((Tumor.Effector_TT_C_Eng/(Tumor.Cancer1+1E-100*mole))^gamma_TC_Assoc)/(s_Assoc + (Tumor.Effector_TT_C_Eng/(Tumor.Cancer1+1E-100*mole))^gamma_TC_Assoc)
404	EffT_Turnover*Tumor.EffT_AR_Vasc
405	J_Tmr*Tumor.EffT_AR_Vasc
406	EffT_Turnover*Tumor.EffT_b_Vasc
407	AR_Tmr*Tumor.EffT_b_Vasc
408	kr_Tmr*Tumor.EffT_b_Vasc
409	kf_Tmr*Tmr_Free_Sites*Tumor.EffT_f_Vasc/Vv_Tmr
410	(QC_Tmr - LC_Tmr)*Tumor.EffT_f_Vasc/Vv_Tmr
411	EffT_Turnover*Tumor.EffT_f_Vasc
412	mAPC_Migrate*Tumor.mAPC_T*(1/[Num_TDLN_Considered])*(1-[mAPC_Debis_T_Inact])
413	mAPC_Migrate*Tumor.mAPC_T*(1-1/[Num_TDLN_Considered])
414	mAPC_Migrate*Tumor.mAPC_T*[mAPC_Debis_T_Inact]
415	[Exp_CD28/80/86/PD1/L1/L2]*Tumor.mAPCT_EngTregT*([CD80_receptors-per-mAPC]/[Avagadro's_Num])*(1/Tr_cells_per_mAPC)
416	[Exp_CD28/80/86/PD1/L1/L2]*Tumor.mAPCT_EngTregT*([CD86_receptors-per-mAPC]/[Avagadro's_Num])*(1/Tr_cells_per_mAPC)
417	TregTMDSCEng*Tumor.Effector_TT_MDSCs*((Tumor.MDSC_T/(Tumor.Effector_TT_MDSCs + 1E-100*mole))^gamma_TC_Assoc)/(s_Assoc + (Tumor.MDSC_T/(Tumor.Effector_TT_MDSCs + 1E-100*mole))^gamma_TC_Assoc)
418	[Exp_CD28/80/86/PD1/L1/L2]*Tumor.MDSCsT_EngTeff*([PD1_receptors-per-Tcell]/[Avagadro's_Num])*(1/T_per_Tr_cell)
419	[Exp_CD28/80/86/PD1/L1/L2]*Tumor.MDSCsT_EngTeff*([PDL1_receptors-per-Tcell]/[Avagadro's_Num])*(1/T_per_Tr_cell)
420	[Treg:T_IntTime]*Tumor.MDSCsT_Teff*[Sig_MDSCTeff=Total]
421	[Treg:T_IntTime]*Tumor.MDSCsT_Teff*(1-[Sig_MDSCTeff=Total])
422	Kpa_TB*S_TB*Vt_avg_const*(1.43/1.63)*Tumor.PD1_mabt/(Vt_Teff*Tumor_Void_Fraction)- Kpa_TB*S_TB*Vt_avg_const*(1.43/1.63)*Lymph_Node.PD1_mab/(VL)
423	[kon_PD1-PD1mAb]*Tumor.PD1_mabt*Tumor.[C14a=PD1]/Vt - [koff_PD1-PD1mAb]*Tumor.[C14a=PD1:aPD1]
424	[kon_PD1-PD1mAb]*Tumor.PD1_mabt*Tumor.[C2=PD1]/Vt - [koff_PD1-PD1mAb]*Tumor.[C2=PD1:aPD1]
425	[kon_PD1-PD1mAb]*Tumor.PD1_mabt*Tumor.[C7a=PD1]/Vt - [koff_PD1-PD1mAb]*Tumor.[C7a=PD1:aPD1]
426	[kon_PD1-PD1mAb]*Tumor.PD1_mabt*Tumor.[C9a=PD1]/Vt - [koff_PD1-PD1mAb]*Tumor.[C9a=PD1:aPD1]
427	[kon_PD1-PD1mAb]*Tumor.PD1_mabt*Tumor.[T10b=PD1]/Vt - [koff_PD1-PD1mAb]*Tumor.[T10b=PD1:aPD1]
428	[kon_PD1-PD1mAb]*Tumor.PD1_mabt*Tumor.[T11b=PD1]/Vt - [koff_PD1-PD1mAb]*Tumor.[T11b=PD1:aPD1]
429	[kon_PD1-PD1mAb]*Tumor.PD1_mabt*Tumor.[T12b=PD1]/Vt - [koff_PD1-PD1mAb]*Tumor.[T12b=PD1:aPD1]
430	[kon_PD1-PD1mAb]*Tumor.PD1_mabt*Tumor.[T14b=PD1]/Vt - [koff_PD1-PD1mAb]*Tumor.[T14b=PD1:aPD1]
431	[kon_PD1-PD1mAb]*Tumor.PD1_mabt*Tumor.[T7b=PD1]/Vt - [koff_PD1-PD1mAb]*Tumor.[T7b=PD1:aPD1]
432	[kon_PD1-PD1mAb]*Tumor.PD1_mabt*Tumor.[T8a=PD1]/Vt - [koff_PD1-PD1mAb]*Tumor.[T8a=PD1:aPD1]
433	[kon_PD1-PD1mAb]*Tumor.PD1_mabt*Tumor.PD1_TeffT/Vt - [koff_PD1-PD1mAb]*Tumor.[PD1:aPD1_Teff]
434	[kon_PD1-PD1mAb]*Tumor.PD1_MDSCsT*Tumor.PD1_mabt/Vt - [koff_PD1-PD1mAb]*Tumor.[PD1:aPD1_MDSCs]
435	[kon_PD1_PDL1]*Tumor.PD1_MDSCsT*Tumor.PDL1_TeffT1/[Vol_Cell-Rec_MDSC-TeffT] - [koff_PD1_PDL1]*Tumor.[PD1:PDL1_MDSCT]
436	[Exp_CD28/80/86/PD1/L1/L2]*(Tumor.PD1_MDSCsT+[PD1:PDL1_MDSCT]+[PD1:aPD1_MDSCs])
437	[kon_PD1_PDL1]*Tumor.PD1_TeffT*Tumor.PDL1_TregT/[Vol_Cell-Rec_Tr-TeffT] - [koff_PD1_PDL1]*Tumor.[PDL1:PD1_TrTeff]
438	[Exp_CD28/80/86/PD1/L1/L2]*(Tumor.PD1_TeffT+[PDL1:PD1_TrTeff]+[PD1:aPD1_Teff])
439	[kon_PD1-PD1mAb]*Tumor.PD1_TeffT1*Tumor.PD1_mabt/Vt - [koff_PD1-PD1mAb]*Tumor.[PD1:aPD1_Teff1]
440	[Exp_CD28/80/86/PD1/L1/L2]*(Tumor.PD1_TeffT1+[PDL1:PD1_MDSCT]+[PD1:aPD1_Teff1])

Reaction Number	Reaction Rate
**441**	[kon_PD1-PD1mAb]*Tumor.PD1_TregT*Tumor.PD1_mabt/Vt - [koff_PD1-PD1mAb]*Tumor.[PD1:aPD1_Treg]
**442**	[Exp_CD28/80/86/PD1/L1/L2]*(Tumor.PD1_TregT+[PD1:PDL1_TrTeff]+[PD1:aPD1_Treg])
**443**	Kpa_TB*S_TB*Vt_avg_const*(1.43/1.63)*Tumor.PDL1_mabt/(Vt_Teff*Tumor_Void_Fraction)-Kpa_TB*S_TB*Vt_avg_const*(1.43/1.63)*Lymph_Node.PDL1_mab/(VL)
**444**	[kon_PDL1-PDL1mAb]*Tumor.PDL1_mabt*Tumor.[C3=PDL1]/Vt - [koff_PDL1-PDL1mAb]*Tumor.[C3=PDL1:aPDL1]
**445**	[kon_PDL1-PDL1mAb]*Tumor.PDL1_mabt*Tumor.[T13a=PDL1]/Vt - [koff_PDL1-PDL1mAb]*Tumor.[T13a=PDL1:aPDL1]
**446**	[kon_PDL1-PDL1mAb]*Tumor.PDL1_mabt*Tumor.[T2=PDL1]/Vt - [koff_PDL1-PDL1mAb]*Tumor.[T2=PDL1:aPDL1]
**447**	[kon_PDL1-PDL1mAb]*Tumor.PDL1_mabt*Tumor.[T7a=PDL1]/Vt - [koff_PDL1-PDL1mAb]*Tumor.[T7a=PDL1:aPDL1]
**448**	[kon_PDL1-PDL1mAb]*Tumor.PDL1_mabt*Tumor.PDL1_TeffT/Vt - [koff_PDL1-PDL1mAb]*Tumor.[PDL1:aPDL1_Teff]
**417**	[kon_PDL1-PDL1mAb]*Tumor.PDL1_mabt*Tumor.PDL1_TeffT1/Vt -[koff_PDL1-PDL1mAb]*Tumor.[PDL1:aPDL1_Teff1]
**418**	[kon_PDL1_CD80]*Tumor.PDL1_MDSCsT*Tumor.CD80_TeffT1/[Vol_Cell-Rec_MDSC-TeffT] - [koff_PDL1_CD80]*Tumor.[PDL1:CD80_MDSCT]
**419**	[kon_PD1_PDL1]*Tumor.PDL1_MDSCsT*Tumor.PD1_TeffT1/[Vol_Cell-Rec_MDSC-TeffT] - [koff_PD1_PDL1]*Tumor.[PDL1:PD1_MDSCT]
**420**	[kon_PDL1-PDL1mAb]*Tumor.PDL1_MDSCsT*Tumor.PDL1_mabt/Vt - [koff_PDL1-PDL1mAb]*Tumor.[PDL1:aPDL1_MDSCs]
**421**	[Exp_CD28/80/86/PD1/L1/L2]*(Tumor.PDL1_MDSCsT+[PDL1:CD80_MDSCT]+[PDL1:PD1_MDSCT]+[PDL1:aPDL1_MDSCs])
**422**	[kon_PD1_PDL1]*Tumor.PDL1_TeffT*Tumor.PD1_TregT/[Vol_Cell-Rec_Tr-TeffT] - [koff_PD1_PDL1]*Tumor.[PD1:PDL1_TrTeff]
**423**	[Exp_CD28/80/86/PD1/L1/L2]*(Tumor.PDL1_TeffT+[PD1:PDL1_TrTeff]+[PDL1:aPDL1_Teff])
**424**	[Exp_CD28/80/86/PD1/L1/L2]*(Tumor.PDL1_TeffT1+[PD1:PDL1_MDSCT]+[PDL1:aPDL1_Teff1])
**425**	[kon_PDL1-PDL1mAb]*Tumor.PDL1_TregT*Tumor.PDL1_mabt/Vt - [koff_PDL1-PDL1mAb]*Tumor.[PDL1:aPDL1_Treg]
**426**	[Exp_CD28/80/86/PD1/L1/L2]*(Tumor.PDL1_TregT+[PDL1:CD80_TrTeff]+[PDL1:PD1_TrTeff]+[PDL1:aPDL1_Treg])
**427**	kf_TRecover*Tumor.T_Recover_Can_Dead*(1-Total_TC_Sig)
**428**	CancerTInt*Tumor.TC1*(1-Total_TC_Sig*[%Sig_Inhibit_Cancer])
**429**	CancerTInt*Tumor.TC1*Total_TC_Sig*[%Sig_Inhibit_Cancer]
**430**	CancerTInt*Tumor.TC2* Total_TC_Sig*[%Sig_Inhibit_Cancer]
**431**	[Exp_CD28/80/86/PD1/L1/L2]*Tumor.Teff_EngMDSC*([CD80_receptors-per-Tcell]/[Avagadro's_Num])*(1/Tr_per_T_cell)
**432**	[Exp_CD28/80/86/PD1/L1/L2]*Tumor.Teff_EngMDSC*([PD1_receptors-per-Tcell]/[Avagadro's_Num])*(1/Tr_per_T_cell)
**433**	[Exp_CD28/80/86/PD1/L1/L2]*Tumor.Teff_EngMDSC*([PDL1_receptors-per-Tcell]/[Avagadro's_Num])*(1/Tr_per_T_cell)
**434**	[Exp_CD28/80/86/PD1/L1/L2]*Tumor.Teff_EngTregT*([CD80_receptors-per-Tcell]/[Avagadro's_Num])*(1/Tr_per_T_cell)
**435**	[Exp_CD28/80/86/PD1/L1/L2]*Tumor.Teff_EngTregT*([PD1_receptors-per-Tcell]/[Avagadro's_Num])*(1/Tr_per_T_cell)
**436**	[Exp_CD28/80/86/PD1/L1/L2]*Tumor.Teff_EngTregT*([PDL1_receptors-per-Tcell]/[Avagadro's_Num])*(1/Tr_per_T_cell)
**437**	TregTMDSCEng*Tumor.Effector_TT_TregT *((TregT/(Tumor.Effector_TT_TregT + 1E-20*mole))^gamma_TC_Assoc)/(s_Assoc + (TregT/(Tumor.Effector_TT_TregT + 1E-20*mole))^gamma_TC_Assoc)
**438**	[Exp_CTLA4]*Tumor.TregT_EngAPC*([CTLA4_receptors-Tr]/[Avagadro's_Num])*(1/mAPC_per_Tr_cell)
**439**	[Exp_CD28/80/86/PD1/L1/L2]*Tumor.TregT_EngTeff*([PD1_receptors-per-Tcell]/[Avagadro's_Num])*(1/T_per_Tr_cell)
**440**	[Exp_CD28/80/86/PD1/L1/L2]*Tumor.TregT_EngTeff*([PDL1_receptors-per-Tcell]/[Avagadro's_Num])*(1/T_per_Tr_cell)
**441**	[Treg:T_IntTime]*Tumor.TregT_Teff*[Sig_TrTeff=Total]
**442**	[Treg:T_IntTime]*Tumor.TregT_Teff*(1-[Sig_TrTeff=Total])

Table S2 – Model Reaction Rates (End)

Table S3 – Model Reaction and Rate Descriptions (Start)

Reaction Number	Reaction Description
**1**	Distribution of Anti-CTLA-4 mAb between the central and lymph node compartments
**2**	Distribution of Anti-CTLA-4 mAb between the central and leaky tissues
**3**	Distribution of Anti-CTLA-4 mAb between the central and tight tissues
**4**	Distribution of Anti-CTLA-4 mAb between the central and tumor compartments
**5**	Clearance of Anti-CTLA-4 mAb from the central compartment
**6**	The generation of Effector T cells and their migration into the blood/plasma from the designated number of lymph nodes as a multiple of that from a single lymph node
**7**	Trafficking of free Effector T cells from blood to GI vasculature
**8**	Trafficking of free Effector T cells from blood to Liver vasculature
**9**	Trafficking of free Effector T cells from blood to Spleen vasculature
**10**	Trafficking of free Effector T cells from blood to Lymph Node vasculature
**11**	The natural turnover of Effector T cells in the blood
**12**	Trafficking of free Effector T cells from blood to Peripheral (other tissues not directly accounted for) vasculature
**13**	Trafficking of free Effector T cells from blood to Tumor vasculature
**14**	Distribution of Anti-PD-1 mAb between the central and lymph node compartments
**15**	Distribution of Anti-PD-1 mAb between the central and leaky tissues
**16**	Distribution of Anti-PD-1 mAb between the central and tight tissues
**17**	Distribution of Anti-PD-1 mAb between the central and tumor compartments

Reaction Number	Reaction Description
**18**	Clearance of Anti-PD-1 mAb from the central compartment
**19**	Distribution of Anti-PD-L1 mAb between the central and lymph node compartments
**20**	Distribution of Anti-PD-L1 mAb between the central and leaky tissues
**21**	Distribution of Anti-PD-L1 mAb between the central and tight tissues
**22**	Distribution of Anti-PD-L1 mAb between the central and tumor compartments
**23**	Clearance of Anti-PD-L1 mAb from the central compartment
**24**	Saturation of Anti-PD-L1 mAb clearance from the central compartment
**25**	Trafficking of Effector T cells from GI to Liver vasculature
**26**	Turnover of free Effector T cells in the GI vasculature
**27**	Extravasation of arrested Effector T cells from the Liver vasculature
**28**	Turnover of arrested Effector T cells in the Liver vasculature
**29**	Arrest of bound Effector T cells in the Liver vasculature
**30**	Detachment of bound Effector T cells in the Liver vasculature
**31**	Turnover of bound Effector T cells in the liver vasculature
**32**	Trafficking of free Effector T cells from Liver to Lung vasculature
**33**	Turnover of free Effector T cells in the liver vasculature
**34**	Trafficking of free Effector T cells from Liver to lymph node extravascular space
**35**	Turnover of Effector T cells in the Liver extravascular space
**36**	Extravasation of arrested Effector T cells from the Spleen vasculature
**37**	Turnover of arrested Effector T cells in the Spleen vasculature
**38**	Arrest of bound Effector T cells in the Spleen vasculature
**39**	Detachment of bound Effector T cells in the Spleen vasculature
**40**	Turnover of bound Effector T cells in the Spleen vasculature
**41**	Binding or free Effector T cells to the Spleen vasculature
**42**	Trafficking of free Effector T cells from Spleen to Liver vasculature
**43**	Turnover of free Effector T cells in the Spleen vasculature
**44**	Trafficking of free Effector T cells from Spleen to lymph node extravascular space
**45**	Turnover of Effector T cells in the Spleen extravascular space
**46**	Binding or free Effector T cells to the Liver vasculature
**47**	Extravasation of arrested Effector T cells from the Lungs vasculature
**48**	Turnover of arrested Effector T cells in the Lungs vasculature
**49**	Depletion of arrested Effector T cells in the Lungs vasculature
**50**	Binding or free Effector T cells to the Lungs vasculature
**51**	Trafficking of free Effector T cells from Lungs to (arterial) Blood
**52**	Turnover of free Effector T cells in the Lungs vasculature
**53**	Trafficking of free Effector T cells from Lungs to lymph node extravascular space
**54**	Turnover of Effector T cells in the Lungs extravascular space
**55**	Arrest of bound Effector T cells in the Lungs vasculature
**56**	Detachment of bound Effector T cells in the Lungs vasculature
**57**	Turnover of bound Effector T cells in the Lungs vasculature
**58**	Depletion of bound Effector T cells in the Lungs vasculature
**59**	Interaction at the immunological synapse between CD80 on Primed Naive T cells and PD-L1 on T Regulatory cells in the lymph node
**60**	CD80 expression threshold on Primed Naive T cells for interaction with T Regulatory cells in the lymph node
**61**	Interaction at the immunological synapse between PD-1 on Primed Naive T cells and PD-L1 on T Regulatory cells in the lymph node
**62**	Interaction at the immunological synapse between PD-1 on Primed Naive T cells and Anti-PD-1 mAb in the lymph node
**63**	PD-1 expression threshold on Primed Naive T cells for interaction with T Regulatory cells in the lymph node
**64**	Interaction at the immunological synapse between PD-L1 on Primed Naive T cells and Anti-PD-L1 mAb in the lymph node
**65**	PD-L1 expression threshold on Primed Naive T cells for interaction with T Regulatory cells in the lymph node
**66**	Inactivation of Naïve T cells by T Regulatory cells in the lymph node
**67**	Dissociation of T Regulatory cells from Naïve T cells in the lymph node without inactivation of the latter
**68**	Inactivation of Primed Naïve T cells by T Regulatory cells in the lymph node
**69**	Dissociation of T Regulatory cells from Primed Naïve T cells in the lymph node without inactivation of the latter
**70**	CD80 expression by Primed Naive T cells for interaction with T Regulatory cells in the lymph node
**71**	PD-1 expression by Primed Naive T cells for interaction with T Regulatory cells in the lymph node
**72**	PD-L1 expression by Primed Naive T cells for interaction with T Regulatory cells in the lymph node
**73**	PD-1 expression by T Regulatory cells for interaction with Primed Naive T cells in the lymph node
**74**	PD-L1 expression by T Regulatory cells for interaction with Primed Naive T cells in the lymph node
**75**	CD80 expression threshold on mAPCs for interaction with T Regulatory cells in the lymph node
**76**	CD86 expression threshold on mAPCs for interaction with T Regulatory cells in the lymph node
**77**	Interaction at the immunological synapse between CD80 on mAPCs and CTLA-4 on T Regulatory cells in the lymph node
**78**	Interaction at the immunological synapse between CD86 on mAPCs and CTLA-4 on T Regulatory cells in the lymph node
**79**	Interaction at the immunological synapse between CTLA-4 on T Regulatory cells and Anti-CTLA-4 mAb in the lymph node
**80**	CTLA-4 expression threshold on T Regulatory cells for interaction with mAPCs in the lymph node
**81**	Interaction at the immunological synapse between PD-L1 on Primed Naive T cells and PD-1 on T Regulatory cells in the lymph node
**82**	Interaction at the immunological synapse between PD-1 on T Regulatory cells and Anti-PD-1 mAb in the lymph node

Reaction Number	Reaction Description
**83**	PD-1 expression threshold on Regulatory cells for interaction with Primed Naive T cells in the lymph node
**84**	Interaction at the immunological synapse between PD-L1 on T Regulatory cells and Anti-PD-L1 mAb in the lymph node
**85**	Interaction at the immunological synapse between PD-L1 on T Regulatory cells and Anti-PD-1 mAb in the lymph node
**86**	Phagocytosis of tumor debris by resident APCs in the lymph node
**87**	Decay of tumor debris in the lymph nodes
**88**	Lymph return of Anti-CTLA-4 to the blood
**89**	Interaction at the immunological synapse between CTLA-4 on Primed Naïve T cells during priming interactions with mAPCS and Anti-CTLA-4 mAb in the lymph node
**90**	Internalization of the CTLA-4 receptor on Primed Naïve T cells following its binding to Anti-CTLA-4 mAb
**91**	Migration of Effector T cells from the lymph node into the blood (removal linked to regeneration by algebraic equation below)
**92**	Return of Effector T cells from lymph node extravascular space to the Lungs vascular space
**93**	Turnover of Effector T cells in the lymph node extravascular space
**94**	Trafficking of free Effector T cells from lymph node vascular space to the Lungs vascular space
**95**	Turnover of free Effector T cells in the lymph node vascular space
**96**	CD80 expression by total mAPCs in the lymph node
**97**	CD86 expression by total mAPCs in the lymph node
**98**	PD-1 expression by total mAPCs in the lymph node
**99**	PD-L1 expression by total mAPCs in the lymph node
**100**	PD-L2 expression by total mAPCs in the lymph node
**101**	Natural turnover of mAPCs in the lymph node
**102**	Inactivation of mAPCs in the lymph node as a result of CTLA-4 secreted from T Regulatory cells in the lymph nodes binding to CD80 and CD86
**103**	CD80 expression threshold on mAPCs in the lymph node
**104**	CD86 expression threshold on mAPCs in the lymph node
**105**	CD80 expression by mAPCs that interact with Naive T cells in the lymph node
**106**	CD86 expression by mAPCs that interact with Naive T cells in the lymph node
**107**	CD80 expression threshold on mAPCs for interaction with Naive T cells in the lymph node
**108**	CD86 expression threshold on mAPCs for interaction with Naive T cells in the lymph node
**109**	CD80 expression by mAPCs that interact with Primed Naive T cells in the lymph node
**110**	CD86 expression by mAPCs that interact with Primed Naive T cells in the lymph node
**111**	PD-1 expression by mAPCs that interact with Primed Naive T cells in the lymph node
**112**	PD-L1 expression by mAPCs that interact with Primed Naive T cells in the lymph node
**113**	PD-L2 expression by mAPCs that interact with Primed Naive T cells in the lymph node
**114**	Interaction at the immunological synapse between CD80 on mAPCs and CTLA-4 on Primed Naïve T cells in the lymph node
**115**	Interaction at the immunological synapse between CD80 on mAPCs and PD-L1 on Primed Naïve T cells in the lymph node
**116**	CD80 expression threshold on mAPCs for interaction with Primed Naive T cells in the lymph node
**117**	Interaction at the immunological synapse between CD86 on mAPCs and CTLA-4 on Primed Naïve T cells in the lymph node
**118**	Interaction at the immunological synapse between CD86 on mAPCs and CD28 on Primed Naïve T cells in the lymph node
**119**	CD86 expression threshold on mAPCs for interaction with Primed Naive T cells in the lymph node
**120**	Interaction at the immunological synapse between PD-1 on mAPCs cells during priming interactions with Primed Naïve T cells and Anti-PD-1 mAb in the lymph node
**121**	PD-1 expression threshold on mAPCs for interaction with Primed Naive T cells in the lymph node
**122**	PD-L1 expression threshold on mAPCs for interaction with Primed Naive T cells in the lymph node
**123**	Interaction at the immunological synapse between PD-L2 on mAPCs and PD-1 on Primed Naïve T cells in the lymph node
**124**	PD-L2 expression threshold on mAPCs for interaction with Primed Naive T cells in the lymph node
**125**	PD-1 expression threshold on total mAPCs in the lymph node
**126**	PD-L1 expression threshold on total mAPCs in the lymph node
**127**	PD-L2 expression threshold on total mAPCs in the lymph node
**128**	Engagement of Naïve T cells in the first priming phase in the lymph node
**129**	Naïve T cells cycling out of the lymph node
**130**	Naïve T cells being disengaged in the first priming phase without undergoing successful priming in the lymph node
**131**	Naïve T cells successfully undergoing the first priming phase in the lymph node
**132**	Transendocytosis of the CD80 receptors on mAPCs through CTLA-4 binding and internalization on Primed Naïve T cells in the lymph node
**133**	Transendocytosis of the CD86 receptors on mAPCs through CTLA-4 binding and internalization on Primed Naïve T cells in the lymph node
**134**	Interaction at the immunological synapse between CD80 on mAPCs cells and CD28 on Naïve T cells in the lymph node
**135**	Interaction at the immunological synapse between CD86 on mAPCs cells and CD28 on Naïve T cells in the lymph node
**136**	CD28 expression threshold on Naïve T cells in the lymph node
**137**	Naïve T cells expressing CD28 in the lymph node
**138**	Lymph return of Anti-PD-1 to the blood
**139**	Lymph return of Anti-PD-L1 to the blood
**140**	Interaction at the immunological synapse between PD-L1 on mAPCs involved in the second priming phase and Anti-PD-L1 mAb in the lymph node
**141**	Interaction at the immunological synapse between PD-L1 on Primed Naïve T cells involved in the second priming phase with mAPCs and Anti-PD-L1 mAb in the lymph node
**142**	CTLA-4 expression threshold on Primed Naïve T cells for interaction with mAPCs in the lymph node
**143**	Interaction at the immunological synapse between CD80 on mAPCs and CD28 on Primed Naïve T cells in the lymph node

Reaction Number	Reaction Description
**144**	CD28 expression threshold on Primed Naïve T cells for interaction with mAPCs in the lymph node
**145**	Interaction at the immunological synapse between PD-L1 on mAPCs and CD80 on Primed Naïve T cells in the lymph node
**146**	CD80 expression threshold on Primed Naïve T cells for interaction with mAPCs in the lymph node
**147**	Interaction at the immunological synapse between PD-L1 on mAPCs and PD-1 on Primed Naïve T cells in the lymph node
**148**	Interaction at the immunological synapse between PD-1 on Primed Naïve T cells involved in the second priming phase with mAPCs and Anti-PD-1 mAb in the lymph node
**149**	PD-1 expression threshold on Primed Naïve T cells for interaction with mAPCs in the lymph node
**150**	Interaction at the immunological synapse between PD-1 on mAPCs and PD-L1 on Primed Naïve T cells in the lymph node
**151**	PD-L1 expression threshold on Primed Naïve T cells for interaction with mAPCs in the lymph node
**152**	CTLA-4 expression by Primed Naïve T cells for interaction with mAPCs in the lymph node
**153**	CD28 expression by Primed Naïve T cells for interaction with mAPCs in the lymph node
**154**	CD80 expression by Primed Naïve T cells for interaction with mAPCs in the lymph node
**155**	PD-1 expression by Primed Naïve T cells for interaction with mAPCs in the lymph node
**156**	PD-L1 expression by Primed Naïve T cells for interaction with mAPCs in the lymph node
**157**	Primed Naïve T cells engaging in the second priming phase in the lymph node
**158**	Primed Naïve T cells unsuccessfully undergoing the second priming phase to become anergic T cells in the lymph node
**159**	Primed Naïve T cells engaged in the second priming phase disengaging form being primed in the lymph node
**160**	Primed Naïve T cells engaged in the second priming phase successfully undergoing priming to become Proliferating T cells in the lymph node
**161**	Proliferating T cells becoming fully activated Effector T cells in the lymph node
**162**	Sets the proliferation threshold for Proliferating T cells in the lymph node
**163**	Internalization of the Anti-CTLA-4 antibodies bound to CTLA-4 receptors on T Regulatory cells in the lymph node
**164**	Transendocytosis of the CD80 receptors on mAPCs through CTLA-4 binding and internalization on T Regulatory cells in the lymph node
**165**	Transendocytosis of the CD86 receptors on mAPCs through CTLA-4 binding and internalization on T Regulatory cells in the lymph node
**166**	CTLA-4 expression on the surface of non-interacting T Regulatory cells in the lymph node
**167**	Interaction between T Regulatory cells and mAPCs in lymph node
**168**	Interaction between T Regulatory cells and Naïve T cells in lymph node
**169**	Interaction between T Regulatory cells and Primed Naïve T cells in lymph node
**170**	Dissociation of T Regulatory cells from mAPCs in the lymph node with inactivation of the latter
**171**	Dissociation of T Regulatory cells from mAPCs in the lymph node without inactivation of the latter
**172**	CD80 expression by mAPCs for interaction with T Regulatory cells in the lymph node
**173**	CD86 expression by mAPCs for interaction with T Regulatory cells in the lymph node
**174**	CTLA-4 expression by T Regulatory cells for interaction with mAPCs in the lymph node
**175**	Binding of Anti-CTLA-4 to CTLA-4 expressed on the surface of non-interacting T Regulatory in the lymph node
**176**	CTLA-4 expression threshold on non-interacting T Regulatory cells in the lymph node
**177**	Administration of Anti-CTLA-4 mAb into the central compartment
**178**	Administration of Anti-PD-1 mAb into the central compartment
**179**	Administration of Anti-PD-L1 mAb into the central compartment
**180**	Naïve T cells cycling into the lymph node
**181**	The proliferation rate of the Proliferating T cells in the lymph node
**182**	Appearance of monocytes in the tumor
**183**	Proliferation of cancer cells in the tumor
**184**	Distribution of Anti-CTLA-4 mAb between leaky tissues and lymph node
**185**	Distribution of Anti-CTLA-4 mAb between tight tissues and lymph node
**186**	Trafficking of free Effector T cells from Peripheral tissues vasculature to the lung vasculature
**187**	Turnover of free Effector T cells in the Peripheral tissues vasculature
**188**	Distribution of Anti-PD-1 mAb between leaky tissues and lymph node
**189**	Distribution of Anti-PD-1 mAb between tight tissues and lymph node
**190**	Distribution of Anti-PD-L1 mAb between leaky tissues and lymph node
**191**	Distribution of Anti-PD-L1 mAb between tight tissues and lymph node
**192**	CD80 expression by the T5 subtype of cancer cells in the tumor
**193**	PD-1 expression by the T2 subtype of cancer cells in the tumor
**194**	PD-1 expression by the T9 subtype of cancer cells in the tumor
**195**	CD80 expression by the T9 subtype of cancer cells in the tumor
**196**	PD-1 expression by the T6 subtype of cancer cells in the tumor
**197**	PD-L1 expression by the T6 subtype of cancer cells in the tumor
**198**	PD-1 expression by the T14 subtype of cancer cells in the tumor
**199**	PD-L1 expression by the T14 subtype of cancer cells in the tumor
**200**	CD80 expression by the T14 subtype of cancer cells in the tumor
**201**	PD-1 expression by the T10 subtype of cancer cells in the tumor
**202**	PD-L1 expression by the T10 subtype of cancer cells in the tumor
**203**	PD-L2 expression by the T10 subtype of cancer cells in the tumor
**204**	PD-1 expression by the T16 subtype of cancer cells in the tumor
**205**	PD-L1 expression by the T16 subtype of cancer cells in the tumor
**206**	PD-L2 expression by the T16 subtype of cancer cells in the tumor
**207**	CD80 expression by the T16 subtype of cancer cells in the tumor

Reaction Number	Reaction Description
**208**	PD-1 expression by the T7 subtype of cancer cells in the tumor
**209**	PD-L2 expression by the T7 subtype of cancer cells in the tumor
**210**	PD-1 expression by the T15 subtype of cancer cells in the tumor
**211**	PD-L2 expression by the T15 subtype of cancer cells in the tumor
**212**	CD80 expression by the T15 subtype of cancer cells in the tumor
**213**	PD-L1 expression by the T3 subtype of cancer cells in the tumor
**214**	PD-L1 expression by the T11 subtype of cancer cells in the tumor
**215**	CD80 expression by the T11 subtype of cancer cells in the tumor
**216**	PD-L1 expression by the T8 subtype of cancer cells in the tumor
**217**	PD-L2 expression by the T8 subtype of cancer cells in the tumor
**218**	PD-L1 expression by the T13 subtype of cancer cells in the tumor
**219**	PD-L2 expression by the T13 subtype of cancer cells in the tumor
**220**	CD80 expression by the T13 subtype of cancer cells in the tumor
**221**	PD-L2 expression by the T4 subtype of cancer cells in the tumor
**222**	PD-L2 expression by the T12 subtype of cancer cells in the tumor
**223**	CD80 expression by the T12 subtype of cancer cells in the tumor
**224**	Interaction at the immunological synapse between PD-1 expressed by the T10 subtype of cancer cells and Anti-PD-1 in the tumor
**225**	PD-1 expression threshold by the T10 subtype of cancer cells in the tumor
**226**	Interaction at the immunological synapse between PD-L1 expressed by the T10 subtype of cancer cells and Anti-PD-L1 in the tumor
**227**	PD-L1 expression threshold by the T10 subtype of cancer cells in the tumor
**228**	PD-L2 expression threshold by the T10 subtype of cancer cells in the tumor
**229**	Interaction at the immunological synapse between PD-L1 expressed by the T11 subtype of cancer cells and Anti-PD-L1 in the tumor
**230**	PD-L1 expression threshold by the T11 subtype of cancer cells in the tumor
**231**	CD80 expression threshold by the T11 subtype of cancer cells in the tumor
**232**	PD-L2 expression threshold by the T12 subtype of cancer cells in the tumor
**233**	CD80 expression threshold by the T12 subtype of cancer cells in the tumor
**234**	Interaction at the immunological synapse between PD-L1 expressed by the T13 subtype of cancer cells and Anti-PD-L1 in the tumor
**235**	PD-L1 expression threshold by the T13 subtype of cancer cells in the tumor
**236**	PD-L2 expression threshold by the T13 subtype of cancer cells in the tumor
**237**	CD80 expression by the T13 subtype of cancer cells in the tumor
**238**	Interaction at the immunological synapse between PD-1 expressed by the T14 subtype of cancer cells and PD-L1 on the complementary Effector T cells in the tumor
**239**	PD-1 expression threshold by the T14 subtype of cancer cells in the tumor
**240**	Interaction at the immunological synapse between PD-L1 expressed by the T14 subtype of cancer cells and CD80 on the complementary Effector T cells in the tumor
**241**	Interaction at the immunological synapse between PD-L1 expressed by the T14 subtype of cancer cells and Anti-PD-L1 in the tumor
**242**	PD-L1 expression threshold by the T14 subtype of cancer cells in the tumor
**243**	Interaction at the immunological synapse between CD80 expressed by the T14 subtype of cancer cells and PD-L1 on the complementary Effector T cells in the tumor
**244**	CD80 expression threshold by the T14 subtype of cancer cells in the tumor
**245**	Interaction at the immunological synapse between PD-1 expressed by the T15 subtype of cancer cells and Anti-PD-1 in the tumor
**246**	PD-1 expression threshold by the T15 subtype of cancer cells in the tumor
**247**	Interaction at the immunological synapse between PD-L2 expressed by the T15 subtype of cancer cells and PD-1 on the complementary Effector T cells in the tumor
**248**	PD-L2 expression threshold by the T15 subtype of cancer cells in the tumor
**249**	Interaction at the immunological synapse between CD80 expressed by the T15 subtype of cancer cells and PD-L1 on the complementary Effector T cells in the tumor
**250**	CD80 expression threshold by the T15 subtype of cancer cells in the tumor
**251**	Interaction at the immunological synapse between PD-1 expressed by the T16 subtype of cancer cells and PD-L1 on the complementary Effector T cells in the tumor
**252**	Interaction at the immunological synapse between PD-1 expressed by the T16 subtype of cancer cells and Anti-PD-1 in the tumor
**253**	PD-1 expression threshold by the T16 subtype of cancer cells in the tumor
**254**	Interaction at the immunological synapse between PD-L1 expressed by the T16 subtype of cancer cells and Anti-PD-L1 in the tumor
**255**	PD-L1 expression threshold by the T16 subtype of cancer cells in the tumor
**256**	Interaction at the immunological synapse between PD-L2 expressed by the T16 subtype of cancer cells and PD-1 on the complementary Effector T cells in the tumor
**257**	PD-L2 expression threshold by the T16 subtype of cancer cells in the tumor
**258**	CD80 expression threshold by the T16 subtype of cancer cells in the tumor
**259**	PD-1 expression threshold by the T2 subtype of cancer cells in the tumor
**260**	PD-L1 expression threshold by the T3 subtype of cancer cells in the tumor

Reaction Number	Reaction Description
**261**	PD-L2 expression threshold by the T4 subtype of cancer cells in the tumor
**262**	CD80 expression threshold by the T5 subtype of cancer cells in the tumor
**263**	Interaction at the immunological synapse between PD-L2 expressed by the T6 subtype of cancer cells and PD-1 on the complementary Effector T cells in the tumor
**264**	PD-1 expression threshold by the T6 subtype of cancer cells in the tumor
**265**	Interaction at the immunological synapse between PD-L1 expressed by the T6 subtype of cancer cells and PD-1 on the complementary Effector T cells in the tumor
**266**	Interaction at the immunological synapse between PD-L1 expressed by the T6 subtype of cancer cells and CD80 on the complementary Effector T cells in the tumor
**267**	Interaction at the immunological synapse between PD-L1 expressed by the T6 subtype of cancer cells and Anti-PD-L1 in the tumor
**268**	PD-L1 expression threshold by the T6 subtype of cancer cells in the tumor
**269**	PD-1 expression threshold by the T7 subtype of cancer cells in the tumor
**270**	PD-L2 expression threshold by the T7 subtype of cancer cells in the tumor
**271**	Interaction at the immunological synapse between PD-L1 expressed by the T8 subtype of cancer cells and CD80 on Effector T cells in the tumor
**272**	Interaction at the immunological synapse between PD-L1 expressed by the T8 subtype of cancer cells and Anti-PD-L1 in the tumor
**273**	PD-L1 expression threshold by the T8 subtype of cancer cells in the tumor
**274**	PD-L2 expression threshold by the T8 subtype of cancer cells in the tumor
**275**	Interaction at the immunological synapse between PD-1 expressed by the T9 subtype of cancer cells and PD-L1 on the complementary Effector T cells in the tumor
**276**	PD-1 expression threshold by the T9 subtype of cancer cells in the tumor
**277**	CD80 expression threshold by the T9 subtype of cancer cells in the tumor
**278**	Transendocytosis of the CD80 receptors on mAPCs through CTLA-4 binding and internalization on T Regulatory cells in the tumor
**279**	Transendocytosis of the CD86 receptors on mAPCs through CTLA-4 binding and internalization on T Regulatory cells in the tumor
**280**	Internalization of the Anti-CTLA-4 antibodies bound to CTLA-4 receptors on T Regulatory cells in the tumor
**281**	PD-1 expression by Effector T cells interacting with the T8 subtype of cancer cells in the tumor
**282**	CD80 expression by Effector T cells interacting with the T8 subtype of cancer cells in the tumor
**283**	PD-1 expression by Effector T cells interacting with the T3 subtype of cancer cells in the tumor
**284**	CD80 expression by Effector T cells interacting with the T3 subtype of cancer cells in the tumor
**285**	PD-L1 expression by Effector T cells interacting with the T16 subtype of cancer cells in the tumor
**286**	PD-1 expression by Effector T cells interacting with the T16 subtype of cancer cells in the tumor
**287**	CD80 expression by Effector T cells interacting with the T16 subtype of cancer cells in the tumor
**288**	PD-L1 expression by Effector T cells interacting with the T14 subtype of cancer cells in the tumor
**289**	PD-1 expression by Effector T cells interacting with the T14 subtype of cancer cells in the tumor
**290**	CD80 expression by Effector T cells interacting with the T14 subtype of cancer cells in the tumor
**291**	PD-L1 expression by Effector T cells interacting with the T10 subtype of cancer cells in the tumor
**292**	PD-1 expression by Effector T cells interacting with the T10 subtype of cancer cells in the tumor
**293**	CD80 expression by Effector T cells interacting with the T10 subtype of cancer cells in the tumor
**294**	PD-L1 expression by Effector T cells interacting with the T6 subtype of cancer cells in the tumor
**295**	PD-1 expression by Effector T cells interacting with the T6 subtype of cancer cells in the tumor
**296**	CD80 expression by Effector T cells interacting with the T6 subtype of cancer cells in the tumor
**297**	PD-L1 expression by Effector T cells interacting with the T15 subtype of cancer cells in the tumor
**298**	PD-1 expression by Effector T cells interacting with the T15 subtype of cancer cells in the tumor
**299**	PD-L1 expression by Effector T cells interacting with the T7 subtype of cancer cells in the tumor
**300**	PD-1 expression by Effector T cells interacting with the T7 subtype of cancer cells in the tumor
**301**	PD-L1 expression by Effector T cells interacting with the T13 subtype of cancer cells in the tumor
**302**	PD-1 expression by Effector T cells interacting with the T13 subtype of cancer cells in the tumor
**303**	PD-L1 expression by Effector T cells interacting with the T11 subtype of cancer cells in the tumor
**304**	PD-1 expression by Effector T cells interacting with the T11 subtype of cancer cells in the tumor
**305**	PD-L1 expression by Effector T cells interacting with the T12 subtype of cancer cells in the tumor
**306**	PD-1 expression by Effector T cells interacting with the T12 subtype of cancer cells in the tumor
**307**	PD-1 expression by Effector T cells interacting with the T4 subtype of cancer cells in the tumor
**308**	PD-L1 expression by Effector T cells interacting with the T9 subtype of cancer cells in the tumor
**309**	PD-L1 expression by Effector T cells interacting with the T5 subtype of cancer cells in the tumor
**310**	PD-L1 expression by Effector T cells interacting with the T2 subtype of cancer cells in the tumor
**311**	Interaction at the immunological synapse between PD-1 expressed by the T10 subtype of cancer cells and PD-L1 on the complementary Effector T cells in the tumor
**312**	Interaction at the immunological synapse between PD-L1 expressed by the Effector T cells interacting with the T10 subtype of cancer cells and Anti-PD-L1 in the tumor
**313**	PD-L1 expression threshold by the Effector T cells interacting with the T10 subtype of cancer cells in the tumor
**314**	Interaction at the immunological synapse between PD-L1 expressed by the T10 subtype of cancer cells and PD-1 on the complementary Effector T cells in the tumor
**315**	Interaction at the immunological synapse between PD-L2 expressed by the T10 subtype of cancer cells and PD-1 on the complementary Effector T cells in the tumor

Reaction Number	Reaction Description
**316**	PD-1 expression threshold by the Effector T cells interacting with the T10 subtype of cancer cells in the tumor
**317**	Interaction at the immunological synapse between PD-L1 expressed by the T10 subtype of cancer cells and CD80 on the complementary Effector T cells in the tumor
**318**	CD80 expression threshold by the Effector T cells interacting with the T10 subtype of cancer cells in the tumor
**319**	Interaction at the immunological synapse between PD-L1 expressed by the T11 subtype of cancer cells and CD80 on the complementary Effector T cells in the tumor
**320**	Interaction at the immunological synapse between PD-L1 expressed by the Effector T cells interacting with the T11 subtype of cancer cells and Anti-PD-L1 in the tumor
**321**	PD-L1 expression threshold by the Effector T cells interacting with the T11 subtype of cancer cells in the tumor
**322**	Interaction at the immunological synapse between PD-L1 expressed by the T11 subtype of cancer cells and PD-1 on the complementary Effector T cells in the tumor
**323**	PD-1 expression threshold by the Effector T cells interacting with the T11 subtype of cancer cells in the tumor
**324**	Interaction at the immunological synapse between CD80 expressed by the T12 subtype of cancer cells and PD-L1 on the complementary Effector T cells in the tumor
**325**	Interaction at the immunological synapse between PD-L1 expressed by the Effector T cells interacting with the T12 subtype of cancer cells and Anti-PD-L1 in the tumor
**326**	PD-L1 expression threshold by the Effector T cells interacting with the T12 subtype of cancer cells in the tumor
**327**	Interaction at the immunological synapse between PD-L2 expressed by the T12 subtype of cancer cells and PD-1 on the complementary Effector T cells in the tumor
**328**	PD-1 expression threshold by the Effector T cells interacting with the T12 subtype of cancer cells in the tumor
**329**	Interaction at the immunological synapse between CD80 expressed by the T13 subtype of cancer cells and PD-L1 on the complementary Effector T cells in the tumor
**330**	PD-L1 expression threshold by the Effector T cells interacting with the T13 subtype of cancer cells in the tumor
**331**	Interaction at the immunological synapse between PD-L1 expressed by the T13 subtype of cancer cells and PD-1 on the complementary Effector T cells in the tumor
**332**	Interaction at the immunological synapse between PD-L2 expressed by the T13 subtype of cancer cells and PD-1 on the complementary Effector T cells in the tumor
**333**	Interaction at the immunological synapse between PD-1 expressed by the Effector T cells interacting with the T13 subtype of cancer cells and Anti-PD-1 in the tumor
**334**	PD-1 expression threshold by the Effector T cells interacting with the T13 subtype of cancer cells in the tumor
**335**	Interaction at the immunological synapse between PD-L1 expressed by the Effector T cells interacting with the T14 subtype of cancer cells and Anti-PD-L1 in the tumor
**336**	PD-L1 expression threshold by the Effector T cells interacting with the T14 subtype of cancer cells in the tumor
**337**	Interaction at the immunological synapse between PD-L1 expressed by the T14 subtype of cancer cells and PD-1 on the complementary Effector T cells in the tumor
**338**	PD-1 expression threshold by the Effector T cells interacting with the T14 subtype of cancer cells in the tumor
**339**	CD80 expression threshold by the Effector T cells interacting with the T14 subtype of cancer cells in the tumor
**340**	Interaction at the immunological synapse between PD-1 expressed by the T15 subtype of cancer cells and PD-L1 on the complementary Effector T cells in the tumor
**341**	Interaction at the immunological synapse between PD-L1 expressed by the Effector T cells interacting with the T15 subtype of cancer cells and Anti-PD-L1 in the tumor
**342**	PD-L1 expression threshold by the Effector T cells interacting with the T15 subtype of cancer cells in the tumor
**343**	Interaction at the immunological synapse between PD-1 expressed by the Effector T cells interacting with the T15 subtype of cancer cells and Anti-PD-1 in the tumor
**344**	PD-1 expression threshold by the Effector T cells interacting with the T15 subtype of cancer cells in the tumor
**345**	Interaction at the immunological synapse between CD80 expressed by the T16 subtype of cancer cells and PD-L1 on the complementary Effector T cells in the tumor
**346**	Interaction at the immunological synapse between PD-L1 expressed by the Effector T cells interacting with the T16 subtype of cancer cells and Anti-PD-L1 in the tumor
**347**	PD-L1 expression threshold by the Effector T cells interacting with the T16 subtype of cancer cells in the tumor
**348**	Interaction at the immunological synapse between PD-L1 expressed by the T16 subtype of cancer cells and PD-1 on the complementary Effector T cells in the tumor
**349**	Interaction at the immunological synapse between PD-1 expressed by the Effector T cells interacting with the T16 subtype of cancer cells and Anti-PD-1 in the tumor
**350**	PD-1 expression threshold by the Effector T cells interacting with the T16 subtype of cancer cells in the tumor
**351**	Interaction at the immunological synapse between PD-L1 expressed by the T16 subtype of cancer cells and CD80 on the complementary Effector T cells in the tumor
**352**	CD80 expression threshold by the Effector T cells interacting with the T16 subtype of cancer cells in the tumor
**353**	Interaction at the immunological synapse between PD-1 expressed by the T2 subtype of cancer cells and PD-L1 on the complementary Effector T cells in the tumor
**354**	PD-L1 expression threshold by the Effector T cells interacting with the T2 subtype of cancer cells in the tumor
**355**	Interaction at the immunological synapse between PD-L1 expressed by the T3 subtype of cancer cells and PD-1 on the complementary Effector T cells in the tumor
**356**	Interaction at the immunological synapse between PD-1 expressed by the Effector T cells interacting with the T3 subtype of cancer cells and Anti-PD-1 in the tumor
**357**	PD-1 expression threshold by the Effector T cells interacting with the T3 subtype of cancer cells in the tumor
**358**	Interaction at the immunological synapse between PD-L1 expressed by the T3 subtype of cancer cells and CD80 on the complementary Effector T cells in the tumor

Reaction Number	Reaction Description
**359**	CD80 expression threshold by the Effector T cells interacting with the T3 subtype of cancer cells in the tumor
**360**	Interaction at the immunological synapse between PD-L2 expressed by the T4 subtype of cancer cells and PD-1 on the complementary Effector T cells in the tumor
**361**	Interaction at the immunological synapse between PD-1 expressed by the Effector T cells interacting with the T4 subtype of cancer cells and Anti-PD-1 in the tumor
**362**	PD-1 expression threshold by the Effector T cells interacting with the T4 subtype of cancer cells in the tumor
**363**	Interaction at the immunological synapse between CD80 expressed by the T5 subtype of cancer cells and PD-L1 on the complementary Effector T cells in the tumor
**364**	Interaction at the immunological synapse between PD-L1 expressed by the Effector T cells interacting with the T5 subtype of cancer cells and Anti-PD-L1 in the tumor
**365**	PD-L1 expression threshold by the Effector T cells interacting with the T5 subtype of cancer cells in the tumor
**366**	Interaction at the immunological synapse between PD-1 expressed by the T6 subtype of cancer cells and PD-L1 on the complementary Effector T cells in the tumor
**367**	Interaction at the immunological synapse between PD-L1 expressed by the Effector T cells interacting with the T6 subtype of cancer cells and Anti-PD-L1 in the tumor
**368**	PD-L1 expression threshold by the Effector T cells interacting with the T6 subtype of cancer cells in the tumor
**369**	Interaction at the immunological synapse between PD-1 expressed by the Effector T cells interacting with the T6 subtype of cancer cells and Anti-PD-1 in the tumor
**370**	PD-1 expression threshold by the Effector T cells interacting with the T6 subtype of cancer cells in the tumor
**371**	CD80 expression threshold by the Effector T cells interacting with the T6 subtype of cancer cells in the tumor
**372**	Interaction at the immunological synapse between PD-1 expressed by the T7 subtype of cancer cells and PD-L1 on the complementary Effector T cells in the tumor
**373**	PD-L1 expression threshold by the Effector T cells interacting with the T7 subtype of cancer cells in the tumor
**374**	Interaction at the immunological synapse between PD-L2 expressed by the T7 subtype of cancer cells and PD-1 on the complementary Effector T cells in the tumor
**375**	PD-1 expression threshold by the Effector T cells interacting with the T7 subtype of cancer cells in the tumor
**376**	Interaction at the immunological synapse between PD-L1 expressed by the T8 subtype of cancer cells and PD-1 on the complementary Effector T cells in the tumor
**377**	Interaction at the immunological synapse between PD-L2 expressed by the T8 subtype of cancer cells and PD-1 on the complementary Effector T cells in the tumor
**378**	PD-1 expression threshold by the Effector T cells interacting with the T8 subtype of cancer cells in the tumor
**379**	CD80 expression threshold by the Effector T cells interacting with the T8 subtype of cancer cells in the tumor
**380**	Interaction at the immunological synapse between CD80 expressed by the T9 subtype of cancer cells and PD-L1 on the complementary Effector T cells in the tumor
**381**	Interaction at the immunological synapse between PD-L1 expressed by the Effector T cells interacting with the T9 subtype of cancer cells and Anti-PD-L1 in the tumor
**382**	PD-L1 expression threshold by the Effector T cells interacting with the T9 subtype of cancer cells in the tumor
**383**	Turnover of resting macrophages
**384**	Phagocytosis of tumor debris by resting APCs in the lymph node
**385**	Transport of tumor debris from the tumor to the lymph node
**386**	Decay of tumor debris in the tumor
**387**	The distribution of tumor debris away from any of the considered lymph nodes
**388**	Accounting for the distribution of tumor debris from the tumor to the lymph nodes other than the lymph node compartment in the model
**389**	Natural turnover of cancer cells in the tumor
**390**	CD80 expression threshold by the mAPCs interacting with the T Regulatory cells in the tumor
**391**	Interaction at the immunological synapse between CD80 expressed by the Effector T cells and PD-L1 on the T Regulatory cells in the tumor
**392**	CD80 expression threshold by the Effector T cells interacting with the T Regulatory cells in the tumor
**393**	CD80 expression threshold by the Effector T cells interacting with the MDSCs in the tumor
**394**	CD86 expression threshold by the mAPCs interacting with the T Regulatory cells in the tumor
**395**	Flow of Anti-CTLA-4 mAb between the tumor and lymph node
**396**	Interaction at the immunological synapse between CD80 expressed by the mAPCs and CTLA-4 on the T Regulatory cells in the tumor
**397**	Interaction at the immunological synapse between CD86 expressed by the mAPCs and CTLA-4 on the T Regulatory cells in the tumor
**398**	Interaction at the immunological synapse between CTLA-4 expressed by the T Regulatory cells and Anti-CTLA-4 in the tumor
**399**	CTLA-4 expression threshold by the T Regulatory cells interacting with the mAPCs in the tumor
**400**	Turnover of Effector T cells in the tumor
**401**	Apoptosis of Effector T cells following complete cancer cell death in the tumor
**402**	Conversion of Effector T cells into Resident Effector Memory cells following complete cancer cell death in the tumor
**403**	Association of Effector T cells with cancer cells in the tumor
**404**	Turnover of arrested Effector T cells in Tumor vasculature
**405**	Extravasation of arrested Effector T cells into Tumor interstitium
**406**	Turnover of bound Effector T cells in Tumor vasculature
**407**	Arrest of bound Effector T cells in the Tumor vasculature
**408**	Detachment of bound Effector T cells in the Tumor vasculature

Reaction Number	Reaction Description
**409**	Binding or free Effector T cells to the Tumor vasculature
**410**	Trafficking of free Effector T cells from the Tumor vasculature to the Lungs vasculature
**411**	Turnover of free Effector T cells in Tumor vasculature
**412**	Transport of mAPCs from the tumor to the lymph node
**413**	Accounting for the distribution of mAPCs from the tumor to the lymph nodes other than the lymph node compartment in the model
**414**	The distribution of mAPCs away from any of the considered lymph nodes
**415**	CD80 expression by mAPCs interacting with the T Regulatory cells in the tumor
**416**	CD86 expression by mAPCs interacting with the T Regulatory cells in the tumor
**417**	Association of Effector T cells with MDSCs in the tumor
**418**	PD-1 expression by MDSCs interacting with the Effector T cells in the tumor
**419**	PD-L1 expression by MDSCs interacting with the Effector T cells in the tumor
**420**	Dissociation of MDSCs from Effector T cells with inactivation of the latter in the tumor
**421**	Dissociation of MDSCs from Effector T cells without inactivation of the latter in the tumor
**422**	Distribution of Anti-PD-1 mAb between Tumor and Lymph Node
**423**	Interaction at the immunological synapse between PD-1 expressed by the T14 subtype of cancer cells and Anti-PD-1 in the tumor
**424**	Interaction at the immunological synapse between PD-1 expressed by the T2 subtype of cancer cells and Anti-PD-1 in the tumor
**425**	Interaction at the immunological synapse between PD-1 expressed by the T7 subtype of cancer cells and Anti-PD-1 in the tumor
**426**	Interaction at the immunological synapse between PD-1 expressed by the T9 subtype of cancer cells and Anti-PD-1 in the tumor
**427**	Interaction at the immunological synapse between PD-1 expressed by the Effector T cells interacting with the T10 subtype of cancer cells and Anti-PD-1 in the tumor
**428**	Interaction at the immunological synapse between PD-1 expressed by the Effector T cells interacting with the T11 subtype of cancer cells and Anti-PD-1 in the tumor
**429**	Interaction at the immunological synapse between PD-1 expressed by the Effector T cells interacting with the T12 subtype of cancer cells and Anti-PD-1 in the tumor
**430**	Interaction at the immunological synapse between PD-1 expressed by the Effector T cells interacting with the T14 subtype of cancer cells and Anti-PD-1 in the tumor
**431**	Interaction at the immunological synapse between PD-1 expressed by the Effector T cells interacting with the T7 subtype of cancer cells and Anti-PD-1 in the tumor
**432**	Interaction at the immunological synapse between PD-1 expressed by the Effector T cells interacting with the T8 subtype of cancer cells and Anti-PD-1 in the tumor
**433**	Interaction at the immunological synapse between PD-1 expressed by the Effector T cells interacting with the T Regulatory cells and Anti-PD-1 in the tumor
**434**	Interaction at the immunological synapse between PD-1 expressed by the MDSCs interacting with Effector T cells and Anti-PD-1 in the tumor
**435**	Interaction at the immunological synapse between PD-L1 expressed by the Effector T cells and PD-1 on the MDSCs in the tumor
**436**	PD-1 expression threshold by the MDSCs interacting with the Effector T cells in the tumor
**437**	Interaction at the immunological synapse between PD-1 expressed by the Effector T cells and PD-L1 on the T Regulatory cells in the tumor
**438**	PD-1 expression threshold by the Effector T cells interacting with the T Regulatory cells in the tumor
**439**	Interaction at the immunological synapse between PD-1 expressed by the Effector T cells interacting with the MDSCs and Anti-PD-1 in the tumor
**440**	PD-1 expression threshold by the Effector T cells interacting with the MDSCs in the tumor
**441**	Interaction at the immunological synapse between PD-1 expressed by the T Regulatory cells interacting with the Effector T cells and Anti-PD-1 in the tumor
**442**	PD-1 expression threshold by the T Regulatory cells interacting with the Effector T cells in the tumor
**443**	Distribution of Anti-PD-L1 mAb between Tumor and Lymph Node
**444**	Interaction at the immunological synapse between PD-L1 expressed by the T3 subtype of cancer cells and Anti-PD-L1 in the tumor
**445**	Interaction at the immunological synapse between PD-L1 expressed by the Effector T cells interacting with the T13 subtype of cancer cells and Anti-PD-L1 in the tumor
**446**	Interaction at the immunological synapse between PD-L1 expressed by the Effector T cells interacting with the T2 subtype of cancer cells and Anti-PD-L1 in the tumor
**447**	Interaction at the immunological synapse between PD-L1 expressed by the Effector T cells interacting with the T7 subtype of cancer cells and Anti-PD-L1 in the tumor
**448**	Interaction at the immunological synapse between PD-L1 expressed by the Effector T cells interacting with the T Regulatory cells and Anti-PD-L1 in the tumor
**417**	Interaction at the immunological synapse between PD-L1 expressed by the Effector T cells interacting with the MDSCs and Anti-PD-L1 in the tumor
**418**	Interaction at the immunological synapse between CD80 expressed by the Effector T cells and PD-L1 on the MDSCs in the tumor
**419**	Interaction at the immunological synapse between PD-1 expressed by the Effector T cells and PD-L1 on the MDSCs in the tumor

Reaction Number	Reaction Description
**420**	Interaction at the immunological synapse between PD-L1 expressed by the MDSCs interacting with the Effector T cells and Anti-PD-L1 in the tumor
**421**	PD-L1 expression threshold by the MDSCs interacting with the Effector T cells in the tumor
**422**	Interaction at the immunological synapse between PD-L1 expressed by the Effector T cells and PD-1 on the T Regulatory cells in the tumor
**423**	PD-L1 expression threshold by the Effector T cells interacting with the T Regulatory cells in the tumor
**424**	PD-1 expression threshold by the Effector T cells interacting with the MDSCs in the tumor
**425**	Interaction at the immunological synapse between PD-L1 expressed by the T Regulatory cells interacting with the Effector T cells and Anti-PD-L1 in the tumor
**426**	PD-1 expression threshold by the T Regulatory cells interacting with the Effector T cells in the tumor
**427**	Recovery of Effector T cells from the delay state following interaction with cancer cells in the tumor
**428**	Generation of cancer debris by killing of the cancer cells by the Effector T cells in the tumor
**429**	Dissociation of Effector T cells from the cancer cells into a delay state in the tumor
**430**	Inactivation of Effector T cells as a result of interactions with the cancer cells in the tumor
**431**	CD80 expression by Effector T cells interacting with the MDSCs in the tumor
**432**	PD-1 expression by Effector T cells interacting with the MDSCs in the tumor
**433**	PD-L1 expression by Effector T cells interacting with the MDSCs in the tumor
**434**	CD80 expression by Effector T cells interacting with the T Regulatory cells in the tumor
**435**	PD-1 expression by Effector T cells interacting with the T Regulatory cells in the tumor
**436**	PD-L1 expression by Effector T cells interacting with the T Regulatory cells in the tumor
**437**	Association of Effector T cells with T Regulatory cells in the tumor
**438**	CTLA-4 expression by T Regulatory cells interacting with the mAPCs in the tumor
**439**	PD-1 expression by T Regulatory cells interacting with the Effector T cells in the tumor
**440**	PD-L1 expression by T Regulatory cells interacting with the Effector T cells in the tumor
**441**	Dissociation of T Regulatory cells from Effector T cells with inactivation of the latter in the tumor
**442**	Dissociation of T Regulatory cells from Effector T cells without inactivation of the latter in the tumor

Table S3 – Model Reaction and Rate Descriptions (End)

Table S4 – Definition of species in the model (Start)

Compartment Location	Variable Name	Units	Variable Definition
**Blood-Lymph**	CTLA4_mabB	mole	Anti-CTLA-4 antibody in the blood
**Blood-Lymph**	CTLA4_mabB_ugml	µg/ml	Serum concentration of Anti-CTLA-4
**Blood-Lymph**	PD1_mabb	mole	Anti-PD-1 antibody in the blood
**Blood-Lymph**	PDL1_mabb	mole	Anti-PD-L1 antibody in the blood
**Blood-Lymph**	PDL1_mabB_ugml	µg/ml	Serum concentration of Anti-PD-L1
**Blood-Lymph**	PD1_mabB_ugml	µg/ml	Serum concentration of Anti-PD-1
**Blood-Lymph**	Effector_TB	cell	Effector T cells that have been generated from all the lymph nodes considered that have migrated into the blood
**Blood-Lymph**	Effector_T_TOTAL	cell	The total number of Effector T cells present that have exited the lymph nodes
**Blood-Lymph**	Effector_T_TB	cell	Effector T cells that have been generated from all the lymph nodes considered that are in the process of migrating into the blood
**Lungs**	EffT_f_LungsVasc	cell	Free effector T cells in the Lung vasculature
**Lungs**	EffT_r_LungsVasc	cell	Bound effector T cells in the Lung vasculature
**Lungs**	EffT_AR_LungsVasc	cell	Arrested effector T cells in the Lung vasculature
**Lungs**	EffT_LungsEx	cell	Effector T cells in the Lung interstitium
**Lungs**	Lung_Free_Sites	cell	Available binding sites for cells in the Lung vasculature
**Liv_Spln_GI**	EffT_Liver_f_Vasc	cell	Free effector T cells in the Liver vasculature
**Liv_Spln_GI**	EffT_LiverEx	cell	Effector T cells in the Liver interstitium
**Liv_Spln_GI**	EffT_Liver_b_Vasc	cell	Bound effector T cells in the Liver vasculature
**Liv_Spln_GI**	EffT_Liver_AR_Vasc	cell	Arrested effector T cells in the Liver vasculature
**Liv_Spln_GI**	Liver_Free_Sites	cell	Available binding sites for cells in the Liver vasculature
**Liv_Spln_GI**	EffT_Spleen_f_Vasc	cell	Free effector T cells in the Spleen vasculature
**Liv_Spln_GI**	EffT_GI_f_Vasc	cell	Free effector T cells in the GI vasculature
**Liv_Spln_GI**	EffT_SpleenEx	cell	Effector T cells in the Spleen interstitium
**Liv_Spln_GI**	EffT_Spleen_b_Vasc	cell	Bound effector T cells in the Spleen vasculature
**Liv_Spln_GI**	EffT_Spleen_AR_Vasc	cell	Arrested effector T cells in the Spleen vasculature
**Liv_Spln_GI**	Spleen_Free_Sites	cell	Available binding sites for cells in the Spleen vasculature
**Lymph_Node**	Naive_T	cell	Naïve T cells that undergo the first phase of priming in the Lymph Nodes
**Lymph_Node**	Primed_Naive_T	cell	Primed Naive T cells that undergo the second phase of priming in the Lymph Nodes

Compartment Location	Variable Name	Units	Variable Definition
**Lymph_Node**	PNT_Int_CD28	mole	CD28 expressed by interacting Primed Naive T cells engaged in the second priming phase
**Lymph_Node**	mAPC	cell	Total number of mature antigen presenting cells (mAPCs) in the lymph node compartment
**Lymph_Node**	Effector_T	cell	Effector T cells that are generate in the lymph node compartment (considering one lymph node)
**Lymph_Node**	CTLA4_mab	mole	Anti-CTLA-4 antibody in the lymph node
**Lymph_Node**	Naive_T0	cell	Naive T cells that can undergo priming in the lymph node
**Lymph_Node**	Prolif_Naive_T	cell	Proliferating Naive T cells that have undergone the second phase of priming in the lymph node
**Lymph_Node**	Anergic_Naive_T	cell	Primed Naive T cells that do not undergo proliferation following the second phase of priming and are deemed to be anergic T cells in the lymph node
**Lymph_Node**	PD1_mabLN_ugml	µg/ml	Concentration of anti-PD-1 antibody in the lymph node
**Lymph_Node**	CTLA4_mab_ugml	µg/ml	Concentration of anti-CTLA-4 antibody in the lymph node
**Lymph_Node**	PDL1_mabLN_ugml	µg/ml	Concentration of anti-PD-L1 antibody in the lymph node
**Lymph_Node**	EffT_LN_ExRec	cell	Effector T cells in the lymph node interstitium
**Lymph_Node**	EffT_LN_f_Vasc	cell	Free effector T cells in the lymph node vasculature
**Lymph_Node**	mAPC1	cell	mAPCs in lymph node interacting with T regulatory cells
**Lymph_Node**	Naive_T2	cell	Naïve T cells in the lymph node interacting with T regulatory cells
**Lymph_Node**	Primed_Naive_T2	cell	Primed naïve T cells in the lymph node interacting with T regulatory cells
**Lymph_Node**	mAPC_cells_per_ml	cell/ml	Calculation of mAPC in the lymph node
**Lymph_Node**	PD1_mab	mole	Anti-PD-1 antibody in the lymph node
**Lymph_Node**	PDL1_mab	mole	Anti-PD-L1 antibody in the lymph node
**Lymph_Node**	Naive_T1	cell	Naive T cells engaged in the first priming phase in the lymph node
**Lymph_Node**	Primed_Naive_T1	cell	Primed Naive T cells engaged in the second priming phase in the lymph node
**Lymph_Node**	mAPC_Total_Calc_Pr	cell	mAPCs in the lymph node that are not engaged in priming
**Lymph_Node**	Total_T_CD8-CD4	cell	Total number of all T cells in the lymph node
**Lymph_Node**	PNT1_Int	cell	Primed Naive T cells engaged in the second priming phase in the lymph node that are accounting for the expression of immune checkpoints
**Lymph_Node**	PNT_CTLA4	mole	CTLA-4 expressed by interacting Primed Naive T cells engaged in the second priming phase
**Lymph_Node**	mAPC_Int_P1	cell	mAPCs interacting with Naive T cells in the first phase of priming
**Lymph_Node**	mAPC_Int_P2	cell	mAPCs interacting with Primed Naive T cells in the second phase of priming
**Lymph_Node**	mAPC_Int_P2_CD80	mole	CD80 receptors expressed by mAPCs that are involved in the second priming phase interactions
**Lymph_Node**	mAPC_Int_P2_CD86	mole	CD86 receptors expressed by mAPCs that are involved in the second priming phase interactions
**Lymph_Node**	POS_Sig_PNT_CD80	mole	Receptor-receptor interactions at the immunological synapse between CD28 expressed on Primed Naive T cells and CD80 expressed on mAPCs during the second phase of priming
**Lymph_Node**	POS_Sig_PNT_CD86	mole	Receptor-receptor interactions at the immunological synapse between CD28 expressed on Primed Naive T cells and CD86 expressed on mAPCs during the second phase of priming
**Lymph_Node**	NEG_Sig_PNT_CD80	mole	Receptor-receptor interactions at the immunological synapse between CTLA-4 expressed on Primed Naive T cells and CD80 expressed on mAPCs during the second phase of priming
**Lymph_Node**	NEG_Sig_PNT_CD86	mole	Receptor-receptor interactions at the immunological synapse between CTLA-4 expressed on Primed Naive T cells and CD80 expressed on mAPCs during the second phase of priming
**Lymph_Node**	CTLA4_mAb_CTLA4	mole	Heterogeneous receptor-antibody interactions at the immunological synapse between CTLA-4 expressed on Primed Naive T cells during the second priming phase and

Compartment Location	Variable Name	Units	Variable Definition
			Anti-CTLA-4 mAb delivered to the lymph node by way of IV injection into the blood
**Lymph_Node**	mAPC_Int_P2_PDL1	mole	PD-L1 receptors expressed by mAPCs that are involved in the second priming phase interactions
**Lymph_Node**	mAPC_Int_P2_PDL2	mole	PD-L2 receptors expressed by mAPCs that are involved in the second priming phase interactions
**Lymph_Node**	mAPC_Int_P2_PD1	mole	PD-1 receptors expressed by mAPCs that are involved in the second priming phase interactions
**Lymph_Node**	PNT_Int_CD80	mole	CD80 expressed by interacting Primed Naive T cells engaged in the second priming phase
**Lymph_Node**	PNT_Int_PD1	mole	PD-1 expressed by interacting Primed Naive T cells engaged in the second priming phase
**Lymph_Node**	PNT_Int_PDL1	mole	PD-L1 expressed by interacting Primed Naive T cells engaged in the second priming phase
**Lymph_Node**	PNT_CD80-PDL1	mole	Receptor-receptor interactions at the immunological synapse between CD80 expressed on Primed Naive T cells and PD-L1 expressed on mAPCs during the second phase of priming
**Lymph_Node**	PNT_PD1-PDL2	mole	Receptor-receptor interactions at the immunological synapse between PD-1 expressed on Primed Naive T cells and PD-L2 expressed on mAPCs during the second phase of priming
**Lymph_Node**	PNT_PD1-PDL1	mole	Receptor-receptor interactions at the immunological synapse between PD-1 expressed on Primed Naive T cells and PD-L1 expressed on mAPCs during the second phase of priming
**Lymph_Node**	PNT_PDL1-CD80	mole	Receptor-receptor interactions at the immunological synapse between PD-L1 expressed on Primed Naive T cells and CD80 expressed on mAPCs during the second phase of priming
**Lymph_Node**	PNT_PDL1-PD1	mole	Receptor-receptor interactions at the immunological synapse between PD-L1 expressed on Primed Naive T cells and PD-1 expressed on mAPCs during the second phase of priming
**Lymph_Node**	PDL1mAb-PNT_PDL1	mole	Heterogenous receptor-antibody interactions at the immunological synapse between PD-L1 expressed on Primed Naive T cells during the second priming phase and Anti-PD-L1 mAb delivered to the lymph node by way of IV injection into the blood
**Lymph_Node**	PDL1mAb-mAPC_PDL1	mole	Heterogeneous receptor-antibody interactions at the immunological synapse between PD-L1 expressed on mAPCs during the second priming phase and Anti-PD-L1 mAb delivered to the lymph node by way of IV injection into the blood
**Lymph_Node**	PD1mAb_mAPC_PD1	mole	Heterogeneous receptor-antibody interactions at the immunological synapse between PD-1 expressed on mAPCs during the second priming phase and Anti-PD-1 mAb delivered to the lymph node by way of IV injection into the blood
**Lymph_Node**	PD1mAb_PNT_PD1	mole	Heterogeneous receptor-antibody interactions at the immunological synapse between PD-1 expressed on Primed Naive T cells during the second priming phase and Anti-PD-1 mAb delivered to the lymph node by way of IV injection into the blood
**Lymph_Node**	mAPC_PD1	mole	Total PD-1 expression by all mAPCs in the lymph node
**Lymph_Node**	mAPC_PDL2	mole	Total PD-L2 expression by all mAPCs in the lymph node
**Lymph_Node**	mAPC_PDL1	mole	Total PD-L1 expression by all mAPCs in the lymph node
**Lymph_Node**	mAPC_CD86	mole	Total CD86 expression by all mAPCs in the lymph node
**Lymph_Node**	mAPC_CD80	mole	Total CD80 expression by all mAPCs in the lymph node
**Lymph_Node**	TregLN-NT	cell	T Regulatory cells in the lymph node that have engaged in interacting with Naïve T cells
**Lymph_Node**	TregLN-PNT	cell	T Regulatory cells in the lymph node that have engaged in interacting with Primed Naïve T cells
**Lymph_Node**	TregLN_mAPC	cell	T Regulatory cells in the lymph node that have engaged in interacting with mAPCs

Compartment Location	Variable Name	Units	Variable Definition
**Lymph_Node**	TregLN	cell	Total number of T Regulatory cells that able to engage with other cell types in the lymph node
**Lymph_Node**	Tr-mAPC_CTLA4	mole	CTLA-4 receptors expressed by T Regulatory cells in the lymph node
**Lymph_Node**	Tr-PNT_PD1	mole	PD-1 receptors expressed by T Regulatory cells in the lymph node
**Lymph_Node**	Tr-PNT_PDL1	mole	PD-L1 receptors expressed by T Regulatory cells in the lymph node
**Lymph_Node**	PNT-Tr_PDL1	mole	PD-L1 receptors expressed by Primed Naïve T cells in the lymph node during engagement with T Regulatory cells
**Lymph_Node**	PNT-Tr_PD1	mole	PD-1 receptors expressed by Primed Naïve T cells in the lymph node during engagement with T Regulatory cells
**Lymph_Node**	PNT-Tr_CD80	mole	CD80 receptors expressed by Primed Naïve T cells in the lymph node during engagement with T Regulatory cells
**Lymph_Node**	TrALN_CT_CD80	mole	Receptor-receptor interactions at the immunological synapse between CTLA-4 expressed on T Regulatory cells and CD80 expressed on mAPCs in the lymph node
**Lymph_Node**	TrALN_CT_CD86	mole	Receptor-receptor interactions at the immunological synapse between CTLA-4 expressed on T Regulatory cells and CD86 expressed on mAPCs in the lymph node
**Lymph_Node**	TrPNT_PD1-L1	mole	Receptor-receptor interactions at the immunological synapse between PD-1 expressed on T Regulatory cells and PD-L1 expressed on Primed Naïve T cells in the lymph node
**Lymph_Node**	TrPNT_PDL1-PD1	mole	Receptor-receptor interactions at the immunological synapse between PD-L1 expressed on T Regulatory cells and PD-1 expressed on Primed Naïve T cells in the lymph node
**Lymph_Node**	TrPNT_PDL1-CD80	mole	Receptor-receptor interactions at the immunological synapse between PD-L1 expressed on T Regulatory cells and CD80 expressed on Primed Naïve T cells in the lymph node
**Lymph_Node**	TrALN_CT_aCT	mole	Heterogeneous receptor-antibody interactions at the immunological synapse between CTLA-4 expressed on T Regulatory cells and Anti-CTLA-4 mAb delivered to the lymph node by way of IV injection into the blood
**Lymph_Node**	TrPNT_PD1_aPD1	mole	Heterogeneous receptor-antibody interactions at the immunological synapse between PD-1 expressed on T Regulatory cells and Anti-PD-1 mAb delivered to the lymph node by way of IV injection into the blood
**Lymph_Node**	PNTTr_PDL1_aPDL1	mole	Heterogeneous receptor-antibody interactions at the immunological synapse between PD-L1 expressed on Primed Naïve T cells and Anti-PD-L1 mAb delivered to the lymph node by way of IV injection into the blood
**Lymph_Node**	TrPNT_PDL1_aPDL1	mole	Heterogeneous receptor-antibody interactions at the immunological synapse between PD-L1 expressed on T Regulatory cells and Anti-PD-L1 mAb delivered to the lymph node by way of IV injection into the blood
**Lymph_Node**	PNTTr_PD1_aPD1	mole	Heterogeneous receptor-antibody interactions at the immunological synapse between PD-1 expressed on Primed Naïve T cells and Anti-PD-1 mAb delivered to the lymph node by way of IV injection into the blood
**Lymph_Node**	Tr-mAPC_CD80	mole	CD80 receptors expressed by mACPs that are interacting with T Regulatory cells in the lymph node
**Lymph_Node**	Tr-mAPC_CD86	mole	CD86 receptors expressed by mACPs that are interacting with T Regulatory cells in the lymph node
**Lymph_Node**	TregLN_mAPC1	cell	T Regulatory cells in the lymph node that have engaged in interacting with mAPCs in the lymph node that are accounting for the expression of immune checkpoints
**Lymph_Node**	TregLN-PNT1	cell	T Regulatory cells in the lymph node that have engaged in interacting with Primed Naïve T cells in the lymph node that are accounting for the expression of immune checkpoints
**Lymph_Node**	C_DebrisLN	cell	Cancer debris that has been transported to the lymph node
**Lymph_Node**	APCLN	cell	Resident antigen presenting cells in the lymph node that have not yet phagocytosed tumor debris

Compartment Location	Variable Name	Units	Variable Definition
**Lymph_Node**	TrLN_CTLA4	mole	CTLA-4 expressed on the surface of non-interacting T Regulatory cells
**Lymph_Node**	TrLN_CT_aCT	mole	Receptor-antibody interactions between CTLA-4 expressed on T Regulatory cells and Anti-CTLA-4 mAb delivered to the lymph node by way of IV injection into the blood
**Lymph_Node**	PNT1_Int1	cell	Naive T cells undergoing the first priming phase in the lymph node that are accounting for the expression of immune checkpoints
**Lymph_Node**	NT1_Int1	cell	Naive T cells undergoing the first priming phase in the lymph node that are accounting for the expression of CD28
**Lymph_Node**	NT_Int_CD28	mole	CD28 expressed to the immunological synapse of Naive T cells that are undergoing the first phase of priming
**Lymph_Node**	mAPC_Int_P1_CD86	mole	CD86 receptors expressed by mAPCs that are involved in the first priming phase interactions with Naïve T cells
**Lymph_Node**	mAPC_Int_P1_CD80	mole	CD80 receptors expressed by mAPCs that are involved in the first priming phase interactions with Naïve T cells
**Lymph_Node**	POS_Sig_NT_CD86	mole	Receptor-receptor interactions at the immunological synapse between CD28 expressed on Naive T cells and CD86 expressed on mAPCs during the first phase of priming
**Lymph_Node**	POS_Sig_NT_CD80	mole	Receptor-receptor interactions at the immunological synapse between CD28 expressed on Naive T cells and CD86 expressed on mAPCs during the first phase of priming
**Peripheral**	CTLA4_leaky_ugml	μg/ml	Concentration of anti-CTLA-4 in leaky tissue
**Peripheral**	CTLA4_mabP_leaky	mole	Amount of anti-CTLA-4 in leaky tissue
**Peripheral**	CTLA4_mabP_tight	mole	Amount of anti-CTLA-4 in tight tissue
**Peripheral**	CTLA4_tight_ugml	μg/ml	Concentration of anti-CTLA-4 in tight tissue
**Peripheral**	EffT_P_f_Vasc	cell	Free effector T cells in the peripheral vasculature
**Peripheral**	PD1_leaky_ugml	μg/ml	Concentration of anti-PD-1 in leaky tissue
**Peripheral**	PD1_mabP_leaky	mole	Amount of anti-PD-1 in leaky tissue
**Peripheral**	PD1_mabP_tight	mole	Amount of anti-PD-1 in tight tissue
**Peripheral**	PD1_tight_ugml	μg/ml	Concentration of anti-PD-1 in tight tissue
**Peripheral**	PDL1_leaky_ugml	μg/ml	Concentration of anti-PD-L1 in leaky tissue
**Peripheral**	PDL1_mabP_leaky	mole	Amount of anti-PD-L1 in leaky tissue
**Peripheral**	PDL1_mabP_tight	mole	Amount of anti-PD-L1 in tight tissue
**Peripheral**	PDL1_tight_ugml	μg/ml	Concentration of anti-PD-L1 in tight tissue
**Tumor**	PD1_mabt	mole	Anti-PD-1 antibody in the tumor compartment
**Tumor**	PDL1_mabt	mole	Anti-PD-L1 antibody in the tumor compartment
**Tumor**	CTLA4_mabt	mole	Anti-CTLA-4 antibody in the tumor compartment
**Tumor**	Effector_TT	cell	Effector T cells in the tumor microenvironment
**Tumor**	Cancer	cell	Cancer cells in the tumor
**Tumor**	Effector_TT_C_Eng	cell	Effector T cells that can engage with cancer cells in the tumor microenvironment
**Tumor**	TC1	cell	Cancer cells that are engaged with Effector T cells in the tumor microenvironment
**Tumor**	T_Recover_Can_Dead	cell	Effector T cells that have disengaged from the cancer cells in the tumor and are in delay state prior to being able to bind other cancer cells
**Tumor**	C_DebrisT	cell	Cancer debris in the tumor that has resulted from cancer death
**Tumor**	TC2	cell	Effector T cells that are engaged with cancer cells in the tumor microenvironment
**Tumor**	Monocytes	cell	Monocytes that migrate into the tumor and differentiate into APCs
**Tumor**	APC_T	cell	Antigen Presenting Cells (APCs) in the tumor that can phagocytose tumor debris (tumor antigens) and become mature APCs
**Tumor**	mAPC_T	cell	Mature Antigen Presenting Cells (mAPCs) in the tumor that resulted from APCs that have phagocytosed tumor debris
**Tumor**	PD1_mabt	mole	Moles of anti-PD-1 antibody in the tumor interstitium
**Tumor**	PD1_mabT_ugml	μg/ml	Concentration of anti-PD-1 antibody in the tumor interstitium

Compartment Location	Variable Name	Units	Variable Definition
**Tumor**	CTLA4_mabt_ugml	µg/ml	Concentration of anti-CTLA-4 antibody in the tumor interstitium
**Tumor**	PDL1_mabT_ugml	µg/ml	Concentration of anti-PD-L1 antibody in the tumor interstitium
**Tumor**	EffT_f_Vasc	cell	Free effector T cells in the tumor vasculature
**Tumor**	Tmr_Free_Sites	cell	Available binding sites for cells in the tumor vasculature
**Tumor**	EffT_b_Vasc	cell	Bound effector T cells in the tumor vasculature
**Tumor**	EffT_AR_Vasc	cell	Arrested effector T cells in the tumor vasculature
**Tumor**	Cancer1	cell	Cancer cells that can engage with Effector T cells in the tumor
**Tumor**	T{PD1}{CD80}-{PDL1}C	cell	Effector T cells in the tumor that interact with the T3 subgroup of cancer cells expressing PD-L1 only, and other unknown factors
**Tumor**	T{PD1}-{PDL2}C	cell	Effector T cells in the tumor that interact with the T4 subgroup of cancer cells expressing PD-L2 only, and other unknown factors
**Tumor**	T{PDL1}-{PD1}C	cell	Effector T cells in the tumor that interact with the T2 subgroup of cancer cells expressing PD-1 only, and other unknown factors
**Tumor**	T{PDL1}-{CD80}C	cell	Effector T cells in the tumor that interact with the T5 subgroup of cancer cells expressing CD80 only, and other unknown factors
**Tumor**	T{PD1}{L1}{80}-{PD1}{L1}C	cell	Effector T cells in the tumor that interact with the T6 subgroup of cancer cells expressing PD-1 and PD-L1 only, and other unknown factors
**Tumor**	T{PD1}{80}-{PDL1}{PDL2}C	cell	Effector T cells in the tumor that interact with the T8 subgroup of cancer cells expressing PD-L1 and PD-L2 only, and other unknown factors
**Tumor**	T{PD1}{L1}-{PD1}{L2}C	cell	Effector T cells in the tumor that interact with the T7 subgroup of cancer cells expressing PD-1 and PD-L2 only, and other unknown factors
**Tumor**	T{PD1}{L1}{80}-{PD1}{L1}{L2}C	cell	Effector T cells in the tumor that interact with the T10 subgroup of cancer cells expressing PD-1, PD-L1 and PD-L2 only, and other unknown factors
**Tumor**	T{PDL1}-{CD80}{PD1}C	cell	Effector T cells in the tumor that interact with the T9 subgroup of cancer cells expressing CD80 and PD-1 only, and other unknown factors
**Tumor**	T{PD1}{L1}-{PDL1}{80}C	cell	Effector T cells in the tumor that interact with the T11 subgroup of cancer cells expressing PD-L1 and CD80 only, and other unknown factors
**Tumor**	T{PD1}{L1}-{PDL2}{80}C	cell	Effector T cells in the tumor that interact with the T12 subgroup of cancer cells expressing PD-L2 and CD80 only, and other unknown factors
**Tumor**	T{PD1}{L1}-{PDL1}{80}{L2}C	cell	Effector T cells in the tumor that interact with the T13 subgroup of cancer cells expressing PD-L1, CD80 and PD-L2 only, and other unknown factors
**Tumor**	T{PD1}{L1}{80}-{PD1}{80}{L1}C	cell	Effector T cells in the tumor that interact with the T14 subgroup of cancer cells expressing PD-1, CD80 and PD-L1 only, and other unknown factors
**Tumor**	T{PD1}{L1}-{PD1}{80}{PDL2}C	cell	Effector T cells in the tumor that interact with the T15 subgroup of cancer cells expressing PD-1, CD80 and PD-L2 only, and other unknown factors
**Tumor**	T{PD1}{L1}{80}-{PD1}{80}{L1}{L2}C	cell	Effector T cells in the tumor that interact with the T16 subgroup of cancer cells expressing PD-1, CD80, PD-L1 and PD-L2 only, and other unknown factors
**Tumor**	T2=PDL1	mole	PD-L1 expressed by Effector T cells that interact with T2 subgroup of cancer cells
**Tumor**	T3a=PD1	mole	PD-1 expressed by Effector T cells that interact with T3 subgroup of cancer cells
**Tumor**	T4=PD1	mole	PD-1 expressed by Effector T cells that interact with T4 subgroup of cancer cells
**Tumor**	T5=PDL1	mole	PD-L1 expressed by Effector T cells that interact with T5 subgroup of cancer cells
**Tumor**	T3b=CD80	mole	CD80 expressed by Effector T cells that interact with T3 subgroup of cancer cells

Compartment Location	Variable Name	Units	Variable Definition
**Tumor**	T7a=PDL1	mole	PD-L1 expressed by Effector T cells that interact with T7 subgroup of cancer cells
**Tumor**	T10b=PD1	mole	PD-1 expressed by Effector T cells that interact with T10 subgroup of cancer cells
**Tumor**	T7b=PD1	mole	PD-1 expressed by Effector T cells that interact with T7 subgroup of cancer cells
**Tumor**	T6a=PDL1	mole	PD-L1 expressed by Effector T cells that interact with T6 subgroup of cancer cells
**Tumor**	T6c=CD80	mole	CD80 expressed by Effector T cells that interact with T6 subgroup of cancer cells
**Tumor**	T6b=PD1	mole	PD-1 expressed by Effector T cells that interact with T6 subgroup of cancer cells
**Tumor**	T11a=PDL1	mole	PD-L1 expressed by Effector T cells that interact with T11 subgroup of cancer cells
**Tumor**	T11b=PD1	mole	PD-1 expressed by Effector T cells that interact with T11 subgroup of cancer cells
**Tumor**	T10c=CD80	mole	CD80 expressed by Effector T cells that interact with T10 subgroup of cancer cells
**Tumor**	T12b=PD1	mole	PD-1 expressed by Effector T cells that interact with T12 subgroup of cancer cells
**Tumor**	T13a=PDL1	mole	PD-L1 expressed by Effector T cells that interact with T13 subgroup of cancer cells
**Tumor**	T12a=PDL1	mole	PD-L1 expressed by Effector T cells that interact with T12 subgroup of cancer cells
**Tumor**	T14c=CD80	mole	CD80 expressed by Effector T cells that interact with T14 subgroup of cancer cells
**Tumor**	T15b=PD1	mole	PD-1 expressed by Effector T cells that interact with T15 subgroup of cancer cells
**Tumor**	T15a=PDL1	mole	PD-L1 expressed by Effector T cells that interact with T15 subgroup of cancer cells
**Tumor**	T13b=PD1	mole	PD-1 expressed by Effector T cells that interact with T13 subgroup of cancer cells
**Tumor**	T14b=PD1	mole	PD-1 expressed by Effector T cells that interact with T14 subgroup of cancer cells
**Tumor**	T14a=PDL1	mole	PD-L1 expressed by Effector T cells that interact with T14 subgroup of cancer cells
**Tumor**	T16b=PD1	mole	PD-1 expressed by Effector T cells that interact with T16 subgroup of cancer cells
**Tumor**	T16c=CD80	mole	CD80 expressed by Effector T cells that interact with T16 subgroup of cancer cells
**Tumor**	T16a=PDL1	mole	PD-L1 expressed by Effector T cells that interact with T16 subgroup of cancer cells
**Tumor**	T8a=PD1	mole	PD-1 expressed by Effector T cells that interact with T8 subgroup of cancer cells
**Tumor**	T8b=CD80	mole	CD80 expressed by Effector T cells that interact with T8 subgroup of cancer cells
**Tumor**	T9=PDL1	mole	PD-L1 expressed by Effector T cells that interact with T9 subgroup of cancer cells
**Tumor**	T10a=PDL1	mole	PD-L1 expressed by Effector T cells that interact with T10 subgroup of cancer cells
**Tumor**	C10a=PD1	mole	PD-1 expressed by the T10 subtype of cancer cells that interact with Effector T cells in the tumor
**Tumor**	C9b=CD80	mole	CD80 expressed by the T9 subtype of cancer cells that interact with Effector T cells in the tumor
**Tumor**	C9a=PD1	mole	PD-1 expressed by the T9 subtype of cancer cells that interact with Effector T cells in the tumor
**Tumor**	C8b=PDL2	mole	PD-L2 expressed by the T8 subtype of cancer cells that interact with Effector T cells in the tumor
**Tumor**	C16a=PD1	mole	PD-1 expressed by the T16 subtype of cancer cells that interact with Effector T cells in the tumor
**Tumor**	C16c=PDL2	mole	PD-L2 expressed by the T16 subtype of cancer cells that interact with Effector T cells in the tumor
**Tumor**	C16b=PDL1	mole	PD-L1 expressed by the T16 subtype of cancer cells that interact with Effector T cells in the tumor

Compartment Location	Variable Name	Units	Variable Definition
**Tumor**	C14a=PD1	mole	PD-1 expressed by the T14 subtype of cancer cells that interact with Effector T cells in the tumor
**Tumor**	C14b=PDL1	mole	PD-L1 expressed by the T14 subtype of cancer cells that interact with Effector T cells in the tumor
**Tumor**	C13b=PDL2	mole	PD-L2 expressed by the T13 subtype of cancer cells that interact with Effector T cells in the tumor
**Tumor**	C15a=PD1	mole	PD-1 expressed by the T15 subtype of cancer cells that interact with Effector T cells in the tumor
**Tumor**	C15b=PDL2	mole	PD-L2 expressed by the T15 subtype of cancer cells that interact with Effector T cells in the tumor
**Tumor**	C14c=CD80	mole	CD80 expressed by the T14 subtype of cancer cells that interact with Effector T cells in the tumor
**Tumor**	C12a=PDL2	mole	PD-L2 expressed by the T12 subtype of cancer cells that interact with Effector T cells in the tumor
**Tumor**	C13a=PDL1	mole	PD-L1 expressed by the T13 subtype of cancer cells that interact with Effector T cells in the tumor
**Tumor**	C12b=CD80	mole	CD80 expressed by the T12 subtype of cancer cells that interact with Effector T cells in the tumor
**Tumor**	C10c=PDL2	mole	PD-L2 expressed by the T10 subtype of cancer cells that interact with Effector T cells in the tumor
**Tumor**	C11b=CD80	mole	CD80 expressed by the T11 subtype of cancer cells that interact with Effector T cells in the tumor
**Tumor**	C11a=PDL1	mole	PD-L1 expressed by the T11 subtype of cancer cells that interact with Effector T cells in the tumor
**Tumor**	C6b=PDL1	mole	PD-L1 expressed by the T6 subtype of cancer cells that interact with Effector T cells in the tumor
**Tumor**	C7a=PD1	mole	PD-1 expressed by the T7 subtype of cancer cells that interact with Effector T cells in the tumor
**Tumor**	C6a=PD1	mole	PD-1 expressed by the T6 subtype of cancer cells that interact with Effector T cells in the tumor
**Tumor**	C8a=PDL1	mole	PD-L1 expressed by the T8 subtype of cancer cells that interact with Effector T cells in the tumor
**Tumor**	C10b=PDL1	mole	PD-L1 expressed by the T10 subtype of cancer cells that interact with Effector T cells in the tumor
**Tumor**	C7b=PDL2	mole	PD-L2 expressed by the T7 subtype of cancer cells that interact with Effector T cells in the tumor
**Tumor**	C5=CD80	mole	CD80 expressed by the T5 subtype of cancer cells that interact with Effector T cells in the tumor
**Tumor**	C4=PDL2	mole	PD-L2 expressed by the T4 subtype of cancer cells that interact with Effector T cells in the tumor
**Tumor**	C3=PDL1	mole	PD-L1 expressed by the T3 subtype of cancer cells that interact with Effector T cells in the tumor
**Tumor**	C2=PD1	mole	PD-1 expressed by the T2 subtype of cancer cells that interact with Effector T cells in the tumor
**Tumor**	C{PD1}	cell	T2 subgroup of cancer cells expressing PD-1 only, and other unknown factors
**Tumor**	C{PDL1}	cell	T3 subgroup of cancer cells expressing PD-L1 only, and other unknown factors
**Tumor**	C{PDL2}	cell	T4 subgroup of cancer cells expressing PD-L2 only, and other unknown factors
**Tumor**	C{CD80}	cell	T5 subgroup of cancer cells expressing CD80 only, and other unknown factors
**Tumor**	C{PD1}{PDL1}	cell	T6 subgroup of cancer cells expressing PD-1 and PD-L1 only, and other unknown factors
**Tumor**	C{PD1}{PDL2}	cell	T7 subgroup of cancer cells expressing PD-1 and PD-L2 only, and other unknown factors
**Tumor**	C{PDL1}{PDL2}	cell	T8 subgroup of cancer cells expressing PD-L1 and PD-L2 only, and other unknown factors
**Tumor**	C{PD1}{CD80}	cell	T9 subgroup of cancer cells expressing PD-1 and CD80 only, and other unknown factors
**Tumor**	C{PD1}{PDL1}{PDL2}	cell	T10 subgroup of cancer cells expressing PD-1, PD-L1 and PD-L2 only, and other unknown factors
**Tumor**	C{PDL1}{CD80}	cell	T11 subgroup of cancer cells expressing PD-L1 and CD80 only, and other unknown factors

Compartment Location	Variable Name	Units	Variable Definition
**Tumor**	C{PDL2}{CD80}	cell	T12 subgroup of cancer cells expressing PD-L2 and CD80 only, and other unknown factors
**Tumor**	C{PDL1}{PDL2}{CD80}	cell	T13 subgroup of cancer cells expressing PD-L1, PDL-L2 and CD80 only, and other unknown factors
**Tumor**	C{PD1}{PDL1}{CD80}	cell	T14 subgroup of cancer cells expressing PD-1, PD-L1 and CD80 only, and other unknown factors
**Tumor**	C{PD1}{PDL2}{CD80}	cell	T15 subgroup of cancer cells expressing PD-1, PD-L2 and CD80 only, and other unknown factors
**Tumor**	C{PD1}{PDL1}{PDL2}{CD80}	cell	T16 subgroup of cancer cells expressing PD-1, PD-L1, PD-L2 and CD80 only, and other unknown factors
**Tumor**	C13c=CD80	mole	CD80 expressed by the T13 subtype of cancer cells that interact with Effector T cells in the tumor
**Tumor**	C15c=CD80	mole	CD80 expressed by the T15 subtype of cancer cells that interact with Effector T cells in the tumor
**Tumor**	C16d=CD80	mole	CD80 expressed by the T16 subtype of cancer cells that interact with Effector T cells in the tumor
**Tumor**	T2=PDL1:PD1=C2	mole	Receptor-receptor interactions at the immunological synapse between PD-1 expressed by the T2 subtype of cancer cells and PD-L1 expressed on Effector T cells
**Tumor**	T3a=PD1:PDL1=C3	mole	Receptor-receptor interactions at the immunological synapse between PD-L1 expressed by the T3 subtype of cancer cells and PD-1 expressed on Effector T cells
**Tumor**	T3b=CD80:PDL1=C3	mole	Receptor-receptor interactions at the immunological synapse between PD-L1 expressed by the T3 subtype of cancer cells and CD80 expressed on Effector T cells
**Tumor**	T4=PD1:PDL2=C4	mole	Receptor-receptor interactions at the immunological synapse between PD-L2 expressed by the T4 subtype of cancer cells and PD-1 expressed on Effector T cells
**Tumor**	T5=PDL1:CD80=C5	mole	Receptor-receptor interactions at the immunological synapse between CD80 expressed by the T5 subtype of cancer cells and PD-L1 expressed on Effector T cells
**Tumor**	T6a=PDL1:PD1=C6a	mole	Receptor-receptor interactions at the immunological synapse between PD-1 expressed by the T6 subtype of cancer cells and PD-L1 expressed on Effector T cells
**Tumor**	T6c=CD80:PDL1=C6b	mole	Receptor-receptor interactions at the immunological synapse between PD-L1 expressed by the T6 subtype of cancer cells and CD80 expressed on Effector T cells
**Tumor**	T6b=PD1:PDL1=C6b	mole	Receptor-receptor interactions at the immunological synapse between PD-L1 expressed by the T6 subtype of cancer cells and PD-1 expressed on Effector T cells
**Tumor**	T7a=PDL1:PD1=C7a	mole	Receptor-receptor interactions at the immunological synapse between PD-1 expressed by the T7 subtype of cancer cells and PD-L1 expressed on Effector T cells
**Tumor**	T7b=PD1:PDL2=C7b	mole	Receptor-receptor interactions at the immunological synapse between PD-L2 expressed by the T7 subtype of cancer cells and PD-1 expressed on Effector T cells
**Tumor**	T8a=PD1:PDL2=C8b	mole	Receptor-receptor interactions at the immunological synapse between PD-L2 expressed by the T8 subtype of cancer cells and PD-1 expressed on Effector T cells
**Tumor**	T8a=PD1:PDL1=C8a	mole	Receptor-receptor interactions at the immunological synapse between PD-L1 expressed by the T8 subtype of cancer cells and PD-1 expressed on Effector T cells
**Tumor**	T8b=CD80:PDL1=C8a	mole	Receptor-receptor interactions at the immunological synapse between PD-L1 expressed by the T8 subtype of cancer cells and CD80 expressed on Effector T cells
**Tumor**	T9=PDL1-PD1=C9a	mole	Receptor-receptor interactions at the immunological synapse between PD-1 expressed by the T9 subtype of cancer cells and PD-L1 expressed on Effector T cells
**Tumor**	T9=PDL1-CD80=C9b	mole	Receptor-receptor interactions at the immunological synapse between CD80 expressed by the T9 subtype of cancer cells and PD-L1 expressed on Effector T cells
**Tumor**	T10a=PDL1:PD1=C10a	mole	Receptor-receptor interactions at the immunological synapse between PD-1 expressed by the T10 subtype of cancer cells and PD-L1 expressed on Effector T cells

Compartment Location	Variable Name	Units	Variable Definition
**Tumor**	T10b=PD1:PDL1=C10b	mole	Receptor-receptor interactions at the immunological synapse between PD-L1 expressed by the T10 subtype of cancer cells and PD-1 expressed on Effector T cells
**Tumor**	T10b=PD1:PDL2=C10c	mole	Receptor-receptor interactions at the immunological synapse between PD-L2 expressed by the T10 subtype of cancer cells and PD-1 expressed on Effector T cells
**Tumor**	T10c=CD80:PDL1=C10b	mole	Receptor-receptor interactions at the immunological synapse between PD-L1 expressed by the T10 subtype of cancer cells and CD80 expressed on Effector T cells
**Tumor**	T11b=PD1:PDL1=C11a	mole	Receptor-receptor interactions at the immunological synapse between PD-L1 expressed by the T11 subtype of cancer cells and PD-1 expressed on Effector T cells
**Tumor**	T11a=PDL1:CD80=C11b	mole	Receptor-receptor interactions at the immunological synapse between CD80 expressed by the T11 subtype of cancer cells and PD-L1 expressed on Effector T cells
**Tumor**	T12b=PD1:PDL2=C12a	mole	Receptor-receptor interactions at the immunological synapse between PD-L2 expressed by the T12 subtype of cancer cells and PD-1 expressed on Effector T cells
**Tumor**	T12a=PDL1:CD80=C12b	mole	Receptor-receptor interactions at the immunological synapse between CD80 expressed by the T12 subtype of cancer cells and PD-L1 expressed on Effector T cells
**Tumor**	T13b=PD1:PDL1=C13a	mole	Receptor-receptor interactions at the immunological synapse between PD-L1 expressed by the T13 subtype of cancer cells and PD-1 expressed on Effector T cells
**Tumor**	T13a=PDL1:CD80=C13c	mole	Receptor-receptor interactions at the immunological synapse between CD80 expressed by the T13 subtype of cancer cells and PD-L1 expressed on Effector T cells
**Tumor**	T13b=PD1:PDL2=C13b	mole	Receptor-receptor interactions at the immunological synapse between PD-L2 expressed by the T13 subtype of cancer cells and PD-1 expressed on Effector T cells
**Tumor**	T14a=PDL1:PD1=C14a	mole	Receptor-receptor interactions at the immunological synapse between PD-1 expressed by the T14 subtype of cancer cells and PD-L1 expressed on Effector T cells
**Tumor**	T14a=PDL1:CD80=C14c	mole	Receptor-receptor interactions at the immunological synapse between CD80 expressed by the T14 subtype of cancer cells and PD-L1 expressed on Effector T cells
**Tumor**	T14b=PD1:PDL1=C14b	mole	Receptor-receptor interactions at the immunological synapse between PD-L1 expressed by the T14 subtype of cancer cells and PD-1 expressed on Effector T cells
**Tumor**	T14c=CD80:PDL1=C14b	mole	Receptor-receptor interactions at the immunological synapse between CD80 expressed by the T14 subtype of cancer cells and PD-L1 expressed on Effector T cells
**Tumor**	T15a=PDL1:CD80=C15c	mole	Receptor-receptor interactions at the immunological synapse between CD80 expressed by the T15 subtype of cancer cells and PD-L1 expressed on Effector T cells
**Tumor**	T15a=PDL1:PD1=C15a	mole	Receptor-receptor interactions at the immunological synapse between PD-1 expressed by the T15 subtype of cancer cells and PD-L1 expressed on Effector T cells
**Tumor**	T15b=PD1:PDL2=C15b	mole	Receptor-receptor interactions at the immunological synapse between PD-L2 expressed by the T15 subtype of cancer cells and PD-1 expressed on Effector T cells
**Tumor**	T16b=PD1:PDL2=C16c	mole	Receptor-receptor interactions at the immunological synapse between PD-L2 expressed by the T15 subtype of cancer cells and PD-1 expressed on Effector T cells
**Tumor**	T16b=PD1:PDL1=C16b	mole	Receptor-receptor interactions at the immunological synapse between PD-L1 expressed by the T15 subtype of cancer cells and PD-1 expressed on Effector T cells
**Tumor**	T16c=CD80:PDL1=C16b	mole	Receptor-receptor interactions at the immunological synapse between PD-L1 expressed by the T15 subtype of cancer cells and CD80 expressed on Effector T cells
**Tumor**	T16a=PDL1:PD1=C16a	mole	Receptor-receptor interactions at the immunological synapse between PD-1 expressed by the T15 subtype of cancer cells and PD-L1 expressed on Effector T cells
**Tumor**	T16a=PDL1:CD80=C16d	mole	Receptor-receptor interactions at the immunological synapse between CD80 expressed by the T15 subtype of cancer cells and PD-L1 expressed on Effector T cells

Compartment Location	Variable Name	Units	Variable Definition
**Tumor**	T2=PDL1:aPDL1	mole	Heterogeneous receptor-antibody interactions at the immunological synapse between PD-L1 expressed by Effector T cells that interact with the T2 subtype of cancer cells and Anti-PD-L1 mAb delivered to the tumor by way of IV injection into the blood
**Tumor**	T3a=PD1:aPD1	mole	Heterogeneous receptor-antibody interactions at the immunological synapse between PD-1 expressed by Effector T cells that interact with the T3 subtype of cancer cells and Anti-PD-L1 mAb delivered to the tumor by way of IV injection into the blood
**Tumor**	C3=PDL1:aPDL1	mole	Heterogeneous receptor-antibody interactions at the immunological synapse between PD-L1 expressed by the T3 subtype of cancer cells and Anti-PD-L1 mAb delivered to the tumor by way of IV injection into the blood
**Tumor**	T5=PDL1:aPDL1	mole	Heterogeneous receptor-antibody interactions at the immunological synapse between PD-L1 expressed by Effector T cells that interact with the T5 subtype of cancer cells and Anti-PD-L1 mAb delivered to the tumor by way of IV injection into the blood
**Tumor**	T6a=PDL1:aPDL1	mole	Heterogeneous receptor-antibody interactions at the immunological synapse between PD-L1 expressed by Effector T cells that interact with the T6 subtype of cancer cells and Anti-PD-L1 mAb delivered to the tumor by way of IV injection into the blood
**Tumor**	C6b=PDL1:aPDL1	mole	Heterogeneous receptor-antibody interactions at the immunological synapse between PD-L1 expressed by the T6 subtype of cancer cells and Anti-PD-L1 mAb delivered to the tumor by way of IV injection into the blood
**Tumor**	T10a=PDL1:aPDL1	mole	Heterogeneous receptor-antibody interactions at the immunological synapse between PD-L1 expressed by Effector T cells that interact with the T10 subtype of cancer cells and Anti-PD-L1 mAb delivered to the tumor by way of IV injection into the blood
**Tumor**	C10b=PDL1:aPDL1	mole	Heterogeneous receptor-antibody interactions at the immunological synapse between PD-L1 expressed by the T10 subtype of cancer cells and Anti-PD-L1 mAb delivered to the tumor by way of IV injection into the blood
**Tumor**	C11a=PDL1:aPDL1	mole	Heterogeneous receptor-antibody interactions at the immunological synapse between PD-L1 expressed by the T11 subtype of cancer cells and Anti-PD-L1 mAb delivered to the tumor by way of IV injection into the blood
**Tumor**	T11a=PDL1:aPDL1	mole	Heterogeneous receptor-antibody interactions at the immunological synapse between PD-L1 expressed by Effector T cells that interact with the T11 subtype of cancer cells and Anti-PD-L1 mAb delivered to the tumor by way of IV injection into the blood
**Tumor**	T12a=PDL1:aPDL1	mole	Heterogeneous receptor-antibody interactions at the immunological synapse between PD-L1 expressed by Effector T cells that interact with the T12 subtype of cancer cells and Anti-PD-L1 mAb delivered to the tumor by way of IV injection into the blood
**Tumor**	T7a=PDL1:aPDL1	mole	Heterogeneous receptor-antibody interactions at the immunological synapse between PD-L1 expressed by Effector T cells that interact with the T7 subtype of cancer cells and Anti-PD-L1 mAb delivered to the tumor by way of IV injection into the blood
**Tumor**	C8a=PDL1:aPDL1	mole	Heterogeneous receptor-antibody interactions at the immunological synapse between PD-L1 expressed by the T8 subtype of cancer cells and Anti-PD-L1 mAb delivered to the tumor by way of IV injection into the blood
**Tumor**	T9=PDL1:aPDL1	mole	Heterogeneous receptor-antibody interactions at the immunological synapse between PD-L1 expressed by Effector T cells that interact with the T9 subtype of cancer cells and Anti-PD-L1 mAb delivered to the tumor by way of IV injection into the blood

Compartment Location	Variable Name	Units	Variable Definition
**Tumor**	C13a=PDL1:aPDL1	mole	Heterogeneous receptor-antibody interactions at the immunological synapse between PD-L1 expressed by the T13 subtype of cancer cells and Anti-PD-L1 mAb delivered to the tumor by way of IV injection into the blood
**Tumor**	T13a=PDL1:aPDL1	mole	Heterogeneous receptor-antibody interactions at the immunological synapse between PD-L1 expressed by Effector T cells that interact with the T13 subtype of cancer cells and Anti-PD-L1 mAb delivered to the tumor by way of IV injection into the blood
**Tumor**	T14a=PDL1:aPDL1	mole	Heterogeneous receptor-antibody interactions at the immunological synapse between PD-L1 expressed by Effector T cells that interact with the T14 subtype of cancer cells and Anti-PD-L1 mAb delivered to the tumor by way of IV injection into the blood
**Tumor**	C14b=PDL1:aPDL1	mole	Heterogeneous receptor-antibody interactions at the immunological synapse between PD-L1 expressed by the T14 subtype of cancer cells and Anti-PD-L1 mAb delivered to the tumor by way of IV injection into the blood
**Tumor**	T15a=PDL1:aPDL1	mole	Heterogeneous receptor-antibody interactions at the immunological synapse between PD-L1 expressed by Effector T cells that interact with the T15 subtype of cancer cells and Anti-PD-L1 mAb delivered to the tumor by way of IV injection into the blood
**Tumor**	T16a=PDL1:aPDL1	mole	Heterogeneous receptor-antibody interactions at the immunological synapse between PD-L1 expressed by Effector T cells that interact with the T16 subtype of cancer cells and Anti-PD-L1 mAb delivered to the tumor by way of IV injection into the blood
**Tumor**	C16b=PDL1:aPDL1	mole	Heterogeneous receptor-antibody interactions at the immunological synapse between PD-L1 expressed by the T16 subtype of cancer cells and Anti-PD-L1 mAb delivered to the tumor by way of IV injection into the blood
**Tumor**	C2=PD1:aPD1	mole	Heterogeneous receptor-antibody interactions at the immunological synapse between PD-1 expressed by the T2 subtype of cancer cells and Anti-PD-1 mAb delivered to the tumor by way of IV injection into the blood
**Tumor**	T4=PD1:aPD1	mole	Heterogeneous receptor-antibody interactions at the immunological synapse between PD-1 expressed by Effector T cells that interact with the T4 subtype of cancer cells and Anti-PD-L1 mAb delivered to the tumor by way of IV injection into the blood
**Tumor**	C6a=PD1:aPD1	mole	Heterogeneous receptor-antibody interactions at the immunological synapse between PD-1 expressed by the T6 subtype of cancer cells and Anti-PD-1 mAb delivered to the tumor by way of IV injection into the blood
**Tumor**	T6b=PD1:aPD1	mole	Heterogeneous receptor-antibody interactions at the immunological synapse between PD-1 expressed by Effector T cells that interact with the T6 subtype of cancer cells and Anti-PD-L1 mAb delivered to the tumor by way of IV injection into the blood
**Tumor**	C7a=PD1:aPD1	mole	Heterogeneous receptor-antibody interactions at the immunological synapse between PD-1 expressed by the T7 subtype of cancer cells and Anti-PD-1 mAb delivered to the tumor by way of IV injection into the blood
**Tumor**	T7b=PD1:aPD1	mole	Heterogeneous receptor-antibody interactions at the immunological synapse between PD-1 expressed by Effector T cells that interact with the T7 subtype of cancer cells and Anti-PD-L1 mAb delivered to the tumor by way of IV injection into the blood
**Tumor**	T8a=PD1:aPD1	mole	Heterogeneous receptor-antibody interactions at the immunological synapse between PD-1 expressed by Effector T cells that interact with the T8 subtype of cancer cells and Anti-PD-L1 mAb delivered to the tumor by way of IV injection into the blood

Compartment Location	Variable Name	Units	Variable Definition
**Tumor**	C9a=PD1:aPD1	mole	Heterogeneous receptor-antibody interactions at the immunological synapse between PD-1 expressed by the T9 subtype of cancer cells and Anti-PD-1 mAb delivered to the tumor by way of IV injection into the blood
**Tumor**	C10a=PD1:aPD1	mole	Heterogeneous receptor-antibody interactions at the immunological synapse between PD-1 expressed by the T10 subtype of cancer cells and Anti-PD-1 mAb delivered to the tumor by way of IV injection into the blood
**Tumor**	T10b=PD1:aPD1	mole	Heterogeneous receptor-antibody interactions at the immunological synapse between PD-1 expressed by Effector T cells that interact with the T10 subtype of cancer cells and Anti-PD-L1 mAb delivered to the tumor by way of IV injection into the blood
**Tumor**	T11b=PD1:aPD1	mole	Heterogeneous receptor-antibody interactions at the immunological synapse between PD-1 expressed by Effector T cells that interact with the T11 subtype of cancer cells and Anti-PD-L1 mAb delivered to the tumor by way of IV injection into the blood
**Tumor**	T13b=PD1:aPD1	mole	Heterogeneous receptor-antibody interactions at the immunological synapse between PD-1 expressed by Effector T cells that interact with the T13 subtype of cancer cells and Anti-PD-L1 mAb delivered to the tumor by way of IV injection into the blood
**Tumor**	C14a=PD1:aPD1	mole	Heterogeneous receptor-antibody interactions at the immunological synapse between PD-1 expressed by the T14 subtype of cancer cells and Anti-PD-1 mAb delivered to the tumor by way of IV injection into the blood
**Tumor**	T14b=PD1:aPD1	mole	Heterogeneous receptor-antibody interactions at the immunological synapse between PD-1 expressed by Effector T cells that interact with the T14 subtype of cancer cells and Anti-PD-L1 mAb delivered to the tumor by way of IV injection into the blood
**Tumor**	C15a=PD1:aPD1	mole	Heterogeneous receptor-antibody interactions at the immunological synapse between PD-1 expressed by the T15 subtype of cancer cells and Anti-PD-1 mAb delivered to the tumor by way of IV injection into the blood
**Tumor**	T15b=PD1:aPD1	mole	Heterogeneous receptor-antibody interactions at the immunological synapse between PD-1 expressed by Effector T cells that interact with the T15 subtype of cancer cells and Anti-PD-L1 mAb delivered to the tumor by way of IV injection into the blood
**Tumor**	C16a=PD1:aPD1	mole	Heterogeneous receptor-antibody interactions at the immunological synapse between PD-1 expressed by the T16 subtype of cancer cells and Anti-PD-1 mAb delivered to the tumor by way of IV injection into the blood
**Tumor**	T16b=PD1:aPD1	mole	Heterogeneous receptor-antibody interactions at the immunological synapse between PD-1 expressed by Effector T cells that interact with the T16 subtype of cancer cells and Anti-PD-L1 mAb delivered to the tumor by way of IV injection into the blood
**Tumor**	T12b=PD1:aPD1	mole	Heterogeneous receptor-antibody interactions at the immunological synapse between PD-1 expressed by Effector T cells that interact with the T12 subtype of cancer cells and Anti-PD-L1 mAb delivered to the tumor by way of IV injection into the blood
**Tumor**	TregT	cell	Total number of T Regulatory cells that able to engage with Effector T cells and mAPCs in the tumor
**Tumor**	TregT_Teff	cell	T Regulatory cells in the tumor that have engaged in interacting with Effector T cells
**Tumor**	MDSC_T	cell	Total number of MDSCs that able to engage with Effector T cells in the tumor
**Tumor**	MDSCsT_Teff	cell	MDSCs in the tumor that have engaged in interacting with Effector T cells
**Tumor**	MDSCsT_EngTeff	cell	MDSCs in the tumor that have engaged in interacting with Effector T cells that are accounting for the expression of PD-1 and PD-L1

Compartment Location	Variable Name	Units	Variable Definition
**Tumor**	TregT_EngAPC	cell	T Regulatory cells in the tumor that have engaged in interacting with mAPCs that are accounting for the expression of CTLA-4
**Tumor**	mAPCT_EngTregT	cell	mAPCs in the tumor that have engaged in interacting with T Regulatory cells that are accounting for the expression of CD80 and CD86
**Tumor**	Teff_EngTregT	cell	Effector T cells in the tumor that have engaged in interacting with T Regulatory cells that are accounting for the expression of CD80, PD-1 and PD-L1
**Tumor**	PD1_TeffT	mole	PD-1 expressed by Effector T cells that interact with T Regulatory cells in the tumor
**Tumor**	PDL1_TeffT	mole	PD-L1 expressed by Effector T cells that interact with T Regulatory cells in the tumor
**Tumor**	CD80_TeffT	mole	CD80 expressed by Effector T cells that interact with T Regulatory cells in the tumor
**Tumor**	CD80_mAPCT	mole	CD80 expressed by mAPCs that interact with T Regulatory cells in the tumor
**Tumor**	PDL1_MDSCsT	mole	PD-L1 expressed by MDSCs that interact with Effector T cells in the tumor
**Tumor**	PD1_MDSCsT	mole	PD-1 expressed by MDSCs that interact with Effector T cells in the tumor
**Tumor**	CD86_mAPCT	mole	CD86 expressed by mAPCs that interact with T Regulatory cells in the tumor
**Tumor**	PD1_TregT	mole	PD-1 expressed by T Regulatory cells that interact with Effector T cells in the tumor
**Tumor**	PDL1_TregT	mole	PD-L1 expressed by T Regulatory cells that interact with Effector T cells in the tumor
**Tumor**	CTLA4_TregT	mole	CTLA-4 expressed by T Regulatory cells that interact with mAPCs in the tumor
**Tumor**	CTLA4:CD80_TrAT	mole	Receptor-receptor interactions at the immunological synapse between CD80 expressed by mAPCs and CTLA-4 expressed on T Regulatory cells in the tumor
**Tumor**	CTLA4:CD86_TrAT	mole	Receptor-receptor interactions at the immunological synapse between CD86 expressed by mAPCs and CTLA-4 expressed on T Regulatory cells in the tumor
**Tumor**	CTLA4_CTLA4-Trt	mole	Heterogeneous receptor-antibody interactions at the immunological synapse between CTLA-4 expressed by T Regulatory cells that interact with the mAPCs and Anti-PD-L1 mAb delivered to the tumor by way of IV injection into the blood
**Tumor**	PDL1:PD1_TrTeff	mole	Receptor-receptor interactions at the immunological synapse between PD-1 expressed by the Effector T cells and PD-L1 expressed on T Regulatory cells in the tumor
**Tumor**	PDL1:CD80_TrTeff	mole	Receptor-receptor interactions at the immunological synapse between CD80 expressed by the Effector T cells and PD-L1 expressed on T Regulatory cells in the tumor
**Tumor**	PD1:PDL1_TrTeff	mole	Receptor-receptor interactions at the immunological synapse between PD-L1 expressed by the Effector T cells and PD-1 expressed on T Regulatory cells in the tumor
**Tumor**	PDL1:CD80_MDSCT	mole	Receptor-receptor interactions at the immunological synapse between CD80 expressed by the Effector T cells and PD-L1 expressed on MDSCs in the tumor
**Tumor**	PDL1:PD1_MDSCT	mole	Receptor-receptor interactions at the immunological synapse between PD-1 expressed by the Effector T cells and PD-L1 expressed on MDSCs cells in the tumor
**Tumor**	PD1:PDL1_MDSCT	mole	Receptor-receptor interactions at the immunological synapse between PD-L1 expressed by the Effector T cells and PD-1 expressed on MDSCs in the tumor
**Tumor**	PD1:aPD1_Teff	mole	Heterogeneous receptor-antibody interactions at the immunological synapse between PD-1 expressed by Effector T cells that interact with the T Regulatory cells and Anti-PD-1 mAb delivered to the tumor by way of IV injection into the blood
**Tumor**	PDL1:aPDL1_Teff	mole	Heterogeneous receptor-antibody interactions at the immunological synapse between PD-L1 expressed by Effector T cells that interact with the T Regulatory cells

Compartment Location	Variable Name	Units	Variable Definition
			and Anti-PD-L1 mAb delivered to the tumor by way of IV injection into the blood
**Tumor**	PDL1:aPDL1_Treg	mole	Heterogeneous receptor-antibody interactions at the immunological synapse between PD-L1 expressed by T Regulatory cells that interact with Effector T cells and Anti-PD-L1 mAb delivered to the tumor by way of IV injection into the blood
**Tumor**	PD1:aPD1_Treg	mole	Heterogeneous receptor-antibody interactions at the immunological synapse between PD-1 expressed by T Regulatory cells that interact with Effector T cells and Anti-PD-1 mAb delivered to the tumor by way of IV injection into the blood
**Tumor**	PD1:aPD1_MDSCs	mole	Heterogeneous receptor-antibody interactions at the immunological synapse between PD-1 expressed by MDSCs that interact with Effector T cells and Anti-PD-1 mAb delivered to the tumor by way of IV injection into the blood
**Tumor**	PDL1:aPDL1_MDSCs	mole	Heterogeneous receptor-antibody interactions at the immunological synapse between PD-L1 expressed by MDSCs that interact with Effector T cells and Anti-PD-L1 mAb delivered to the tumor by way of IV injection into the blood
**Tumor**	TregT_EngTeff	cell	T Regulatory cells in the tumor that have engaged in interacting with Effector T cells that are accounting for the expression of PD-1 and PD-L1
**Tumor**	Teff_EngMDSC	cell	Effector T cells in the tumor that have engaged in interacting with MDSCs that are accounting for the expression of CD80, PD-1 and PD-L1
**Tumor**	CD80_TeffT1	mole	CD80 expressed by Effector T cells that interact with MDSCs in the tumor
**Tumor**	PDL1_TeffT1	mole	PD-L1 expressed by Effector T cells that interact with MDSCs in the tumor
**Tumor**	PD1_TeffT1	mole	PD-1 expressed by Effector T cells that interact with MDSCs in the tumor
**Tumor**	PD1:aPD1_Teff1	mole	Heterogeneous receptor-antibody interactions at the immunological synapse between PD-1 expressed by Effector T cells that interact with the MDSCs and Anti-PD-1 mAb delivered to the tumor by way of IV injection into the blood
**Tumor**	PDL1:aPDL1_Teff1	mole	Heterogeneous receptor-antibody interactions at the immunological synapse between PD-L1 expressed by Effector T cells that interact with the MDSCs and Anti-PD-L1 mAb delivered to the tumor by way of IV injection into the blood
**Tumor**	Effector_TT_TregT	cell	Total number of Effector T cells that able to engage with T Regulatory cells in the tumor
**Tumor**	Effector_TT_MDSCs	cell	Total number of Effector T cells that able to engage with MDSCs in the tumor
**Tumor**	Effector_TT_Res	cell	Effector T cells that have become Resident Effector Memory cells after the tumor has died
**Tumor**	Effector_TT_Count	cell	The total number of Effector and Resident Effector Memory cells
**Tumor**	Effector_TT_per_Treg	cell/ cell	The number of Effector T cells per T Regulatory cells in the tumor
**Tumor**	Effector_TT_per_mL	cell/ mm^3	The volumetric density of Effector T cells per milliliter in the tumor

Table S4 – Definition of species in the model (End)

Table S5 – Model Parameters (Start)

Variable	Value	Units	Source	Description
**%_NT_LN**	55	dimensionless	[1, 2]	Percent Naïve T cells of total T cells in each lymph node (taken as all CD3+ cells)

Variable	Value	Units	Source	Description
**%_Tr_LN**	5-35	dimensionless	[1, 3-5]	Percent Regulatory T cells of total T cells in each lymph node
**%{CD80}{PD1}C**	Calculated	dimensionless	Calculated; see algebraic equations below	Fraction of cancer cells expressing CD80 and PD-1 checkpoint receptors only
**%{CD80}C**	Calculated	dimensionless	Calculated; see algebraic equations below	Fraction of cancer cells expressing the CD80 checkpoint receptor only
**%{Other}C**	Calculated	dimensionless	Calculated; see algebraic equations below	Fraction of cancer cells expressing only unknown factors, other than CD80, PD-1, PD-L1 and PD-L2
**%{PD1}{80}{L1}{L2}C**	Calculated	dimensionless	Calculated; see algebraic equations below	Fraction of cancer cells expressing CD80, PD-1, PD-L1 and PD-L2 checkpoint receptors only
**%{PD1}{80}{L1}C**	Calculated	dimensionless	Calculated; see algebraic equations below	Fraction of cancer cells expressing CD80, PD-1, and PD-L1 checkpoint receptors only
**%{PD1}{80}{PDL2}C**	Calculated	dimensionless	Calculated; see algebraic equations below	Fraction of cancer cells expressing CD80, PD-1, and PD-L2 checkpoint receptors only
**%{PD1}{L1}{L2}C**	Calculated	dimensionless	Calculated; see algebraic equations below	Fraction of cancer cells expressing PD-1, PD-L1 and PD-L2 checkpoint receptors only
**%{PD1}{L1}C**	Calculated	dimensionless	Calculated; see algebraic equations below	Fraction of cancer cells expressing PD-1, and PD-L1 checkpoint receptors only
**%{PD1}{L2}C**	Calculated	dimensionless	Calculated; see algebraic equations below	Fraction of cancer cells expressing PD-1, and PD-L2 checkpoint receptors only
**%{PD1}C**	Calculated	dimensionless	Calculated; see algebraic equations below	Fraction of cancer cells expressing the PD-1 checkpoint receptor only
**%{PDL1}{80}{L2}C**	Calculated	dimensionless	Calculated; see algebraic equations below	Fraction of cancer cells expressing CD80, PD-L1, and PD-L1 checkpoint receptors only
**%{PDL1}{80}C**	Calculated	dimensionless	Calculated; see algebraic equations below	Fraction of cancer cells expressing CD80 and PD-L1 checkpoint receptors only
**%{PDL1}{PDL2}C**	Calculated	dimensionless	Calculated; see algebraic equations below	Fraction of cancer cells expressing PD-L1, and PD-L2 checkpoint receptors only
**%{PDL1}C**	Calculated	dimensionless	Calculated; see algebraic equations below	Fraction of cancer cells expressing the PD-L1 checkpoint receptor only
**%{PDL2}{80}C**	Calculated	dimensionless	Calculated; see algebraic equations below	Fraction of cancer cells expressing CD80 and PD-L2 checkpoint receptors only
**%{PDL2}C**	Calculated	dimensionless	Calculated; see algebraic equations below	Fraction of cancer cells expressing the PD-L2 checkpoint receptor only
**%CD80_Exp_Cancer**	5 (mean)	dimensionless	[6-8]	Percent of cancer cells of total cancer cells that are CD80 positive that interact with the Effector T cells in the tumor compartment
**%CD80_receptor_level_PNT**	10	dimensionless	Assumed low	Percent of maximum CD80 receptor expression on Primed Naive T cells in the lymph nodes
**%PD1_Exp_Cancer**	10 (mean)	dimensionless	[9]	Percent of cancer cells of total cancer cells that are PD-1 positive that interact with the Effector T cells in the tumor compartment
**%PD1_receptor_level_PNT**	25	dimensionless	[10]	Percent of maximum PD-1 receptor expression on Primed Naive T cells in the lymph nodes
**%PDL1_Exp_Cancer**	60	dimensionless	[11]	Percent of cancer cells of total cancer cells that are PD-L1 positive that interact with the Effector T cells in the tumor compartment
**%PDL1_receptor_level_PNT**	25	dimensionless	Assumed low	Percent of maximum PD-L1 receptor expression on Primed Naive T cells in the lymph nodes
**%PDL2_Exp_Cancer**	20 (mean)	dimensionless	[12]	Percent of cancer cells of total cancer cells that are PD-L2 positive that interact with the Effector T cells in the tumor compartment

Variable	Value	Units	Source	Description
**%Sig_Inhibit_Cancer**	0.65	dimensionless	Set to fit response	The percent effect that total immune checkpoints on the cancer cells have on inactivating the Effector T cells in the tumor
**%T_Tregs_per_Cancer**	0-0.25	dimensionless	Assumed	Defines the percentage of Regulatory T cells in the tumor compartment as a percent of total tumor cells
**Adhesion_D_Lung_Liv_spln**	1E9	cell/ml	[13]	Effector T cell adhesion density in Lung, Liver, or Spleen vasculature
**Adhesion_Density_Tmr**	5E8	cell/ml	[13]	Effector T cell adhesion density in Tumor vasculature
**AntSpread**	x = 1 to 5 (Where it is used as 10^x)	dimensionless	Estimated during fitting to anti-PD-1 data and varied across range	Sets the number of TAA/TSA released per cancer cell upon natural death and decay or following cytotoxic killing. The value is used to set a multiple of cancer cells in the formation of debris for APC maturation.
**Antigen_Intensity**	Assigned from 0.8-1.0 for melanoma	dimensionless	Upper range of cancer types [14]	The strength of the tumor antigens that are involved in priming of the T cells in the lymph nodes
**AR_Liver**	1.5	1/minute	[13]	Effector T cell arrest rate in Liver vasculature
**AR_Lungs**	0.0027	1/minute	[13]	Effector T cell arrest rate in Lungs vasculature
**AR_Spleen**	0.0036	1/minute	[13]	Effector T cell arrest rate in Spleen vasculature
**AR_Tmr**	1	1/minute	[13]	Effector T cell arrest rate in Tumor vasculature
**Avogadro's_Num**	6.022E+23	molecules /mole	Exact value	Avogadro's number – converts receptor numbers to moles
**BodyWeight (kg)**	80	kg	[15]	Body weight of an average human being
**Cancer_Cell_Diam_um**	12	µm	[16, 17]	Diameter of each cancer cell when considering each cell's volume to be represented by a sphere
**Cancer_Cell_Vol_mm3**	9.0478E-7	mm^3	Calculated; see algebraic equations below	Volume of each cancer cell when the diameter is that of a sphere
**Cancer_Diam_mm**	Calculated	mm	Calculated; see algebraic equations below	Diameter of the entire tumor – considering all cells and a void fraction
**Cancer_mm_Start_Therapy**	Assigned	mm	Assigned	Diameter of the tumor when to start the therapeutic regimen
**Cancer_per_T_Cell_Int**	Set to 1	cell/cell	Variable	Number of cancer cells that interact with each Effector T cell in the model
**Cancer_per_T_Cell_max**	4	cell/cell	[18, 19]	Maximum number of cancer cells that can interact with each Effector T cell in the model; assuming that each Effector T cell uses 25% of its surface area to interact with each cancer cell (can be up to 50%) [19].
**Cancer_Vol_cm3**	Calculated	cm^3	Calculated; see algebraic equations below	Volume of the entire tumor – considering all cells and a void fraction
**CancerTEng**	2.079	1/hour	[20]	Rate constant defining the half-life of engagement between cancer cells and Effector T cells in the tumor (at a migration rate of 5-10 µm/min, T cells encounter and engage with a cancer cell approximately every 15 minutes to 1 hour).
**CancerTInt**	0.03465	1/minute	[20]	Rate constant defining the half-life of dissociation between Effector T cells and cancer cells (thus, determining total time of interaction)
**CD28_POS-Sig_NT**	Calculated	dimensionless	Calculated with RO; see algebraic equations below	Fraction of total CD28 receptors on Naïve T cells that are involved in interacting with the CD80 and CD86 receptors on mAPCs during priming in the lymph nodes
**CD28_POS-to-Total**	Calculated	dimensionless	Calculated with RO; see algebraic equations below	Fraction of total CD28 receptors on Primed Naïve T cells that are involved in interacting with the CD80 and CD86 receptors on mAPCs during priming in the lymph nodes
**CD28_receptors-per-Tcell**	15000	molecules/cell	[21]	Number of CD28 receptors expressed on each T cell during priming
**CD80_PNT_NEG-to-Max**	Calculated	dimensionless	Calculated with RO; see algebraic equations below	Fraction of total CD80 receptors on Primed Naïve T cells that are involved in interacting with the PD-L1 receptors on mAPCs during priming in the lymph nodes

Variable	Value	Units	Source	Description
**CD80_receptors_per_C_Cl**	30000	molecules/cell	Assumed same as T cells	Number of CD80 receptors expressed on each cancer cell (assumed same as for T cells).
**CD80_receptors-per-mAPC**	130000	molecules/cell	[22]	Maximum number of CD80 receptors expressed on each mAPC
**CD80_receptors-per-Tcell**	30000	molecules/cell	[23, 24]	Number of CD80 receptors expressed on each T cell (by acquisition of CD80 from mAPCs over two rounds of priming) [24].
**CD80Sig_Tr-PNT**	Calculated	dimensionless	Calculated with RO; see algebraic equations below	Fraction of total CD80 receptors on Primed Naïve T cells that are involved in interacting with the PD-L1 receptors on Regulatory T cells during priming in the lymph nodes
**CD86_receptors-per-mAPC**	208000	molecules/cell	[22]	Maximum number of CD86 receptors expressed on each mAPC
**Chemokine**	100	dimensionless	Estimated during anti-PD-1 therapy fitting; varied in sensitivity analysis	The chemokine factor to promote extravasation of effector T cells into the tumor
**Cl_CTLA4**	0.00373614 1	1/hour	[15]	Clearance rate of Anti-CTLA-4 mAb from the central compartment
**Cl_PD1**	0.01457072 2	liter/hour	[25]	Clearance rate of Anti-PD-1 mAb from the central compartment
**Cl_PDL1**	0.01653012 9	liter/hour	[26, 27]	Clearance rate of Anti-PD-L1 mAb from the central compartment
**Copies-per-T_Cell_Clone**	10-130	dimensionless	[28-30]	Number of copies of each Naïve T cell clone in the lymph nodes.
**CTLA4_change_schedule**	Based on regimen	day	[15] Based on regimen	Time in simulation to switch schedule in regimen for Anti-CTLA-4 therapy
**CTLA4_counter_off**	Based on regimen	day	[15] Based on regimen	Time in simulation to stop Anti-CTLA-4 dose
**CTLA4_counter_on**	Based on regimen	day	[15] Based on regimen	Time in simulation to start Anti-CTLA-4 dose
**CTLA4_DoseSet**	Based on regimen	mg/kg	Set dose	Dose of anti-CTLA-4 therapy
**CTLA4_NEG-to-Total**	Calculated; see algebraic equations below	dimensionless	Calculated with RO; see algebraic equations below	Fraction of total CTLA-4 receptors on Primed Naïve T cells that are involved in interacting with the CD80 and CD86 receptors on mAPCs during priming in the lymph nodes
**CTLA4_receptors-Int-PNT**	Calculated; see algebraic equations below	molecules/cell	[31]	Maximum number of CTLA-4 receptors that can be recruited to the immunological synapse by Primed Naive T cells when interacting with mAPCs
**CTLA4_receptors-nInt-PNT**	Calculated; see algebraic equations below	molecules/cell	[32]	Number of CTLA-4 receptors that are expressed on the surface of non-interacting Primed Naive T cells
**CTLA4_receptors-Tr**	Calculated; see algebraic equations below	molecules/cell	Assumed dependent on CD28/CTLA4 ratio	Maximum number of CTLA-4 receptors on T-regulatory cells
**CTLA4mAb**	Based on regimen	mg/kg	[15] Based on regimen	Dose of Anti-CTLA-4 being delivered
**CTLA4mAb_Dose**	Based on regimen	mg/kg	[15] Based on regimen	Dose of Anti-CTLA-4 to deliver based on the current schedule
**CTLA4mAb_New_Dose**	Based on regimen	mg/kg	[15] Based on regimen	Dose of Anti-CTLA-4 to be delivered when schedule is changed
**CTLA4Sig_TrLN**	Calculated	dimensionless	Calculated with RO; see algebraic equations below	Fraction of CTLA-4 receptors expressed on T Regulatory cells that are occupied by Anti-CTLA-4 mAb in the lymph nodes
**CTLA4Sig_TrLN-mAPC**	Calculated	dimensionless	Calculated with RO; see algebraic equations below	Fraction of CTLA-4 receptors expressed on T Regulatory cells that are interacting with CD80 and CD86 on the mAPCs in the lymph nodes
**CTLA4-to-CD28_Ratio_Int**	Calculated	dimensionless	[31]	Ratio of CTLA-4 to CD28 expression on interacting Primed Naive T cells
**CTLA4-to-CD28_Ratio_nInt**	0.035	dimensionless	[32]	Ratio of CTLA-4 to CD28 expression on non-interacting Primed Naive T cells

Variable	Value	Units	Source	Description
**CTLA4-to-CD28_Ratio_Tr**	0.5	dimensionless	[33]	CTLA-4 expression levels on T Regulatory cells relative to CD28 expression on Primed Naive T cells
**day**	1	day	Unit assignment	Assigns value of 1 day to the term day in the model
**Debris_Decay**	2	1/day	[34, 35]	Half-life of tumor debris and non-phagocytosed tumor antigens
**Debris_Transport**	3	1/day	[34]	Transport rate of tumor debris by the lymphatics to the lymph nodes (based on "permeation of tumor debris and the blood")
**Delta_Liver**	1	dimensionless	[13]	Fraction of Effector T cells in extravascular space of Liver that can recirculate
**Delta_LN**	0.018	dimensionless	[13]	Fraction of Effector T cells in extravascular space of Lymph Nodes that can recirculate
**Delta_Lungs**	1	dimensionless	[13]	Fraction of Effector T cells in extravascular space of Lungs that can recirculate
**Delta_Spleen**	1	dimensionless	[13]	Fraction of Effector T cells in extravascular space of Spleen that can recirculate
**Dose_sched_CTLA4**	Based on regimen	day	[15] Based on regimen	Time between sequential doses of Anti-CTLA-4 in a regimen
**Dose_sched_PD1**	Based on regimen	day	[25] Based on regimen	Time between sequential doses of Anti-PD-1 in a regimen
**Dose_sched_PDL1**	Based on regimen	day	[26, 27] Based on regimen	Time between sequential doses of Anti-PD-L1 in a regimen
**Durvalumab_MW**	146300000	mg/mole	[36-39]	Molecular weight of Anti-PD-L1 antibody, Durvalumab
**E_Lungs**	0.0019	1/minute	[13]	Effector T cell depletion rate
**EffT_InOutLN**	0.0693	1/hour	[40]	Rate constant defining the half-life of Naïve T cell migration into and out of the lymph nodes
**EffT_Migrate**	0.9	1/day	[34, 41]	Rate constant defining the half-life of Effector T cell migration from the lymph nodes to the blood
**EffT_Res_Conversion**	0.1155	1/minute	[42, 43]	Rate constant defining the half-life by which Effector T cells in the tumor compartment undergo apoptosis (90%) and become resident Effector T cells (10%) following the death of the tumor [42].
**EffT_Turnover**	0.02	1/day	[34, 35, 44, 45]	Rate constant defining the half-life of Effector T cells
**Endo_CTLA4**	0.3465	1/minute	[46, 47]	Rate constant defining the half-life of (trans)endocytosis of CTLA-4 on T cells
**EndTherapy**	1	dimensionless	Assigned when to stop therapy	Parameter used to define when to stop therapy after tumor becomes smaller than a certain size
**Exp_All_mAPCLN**	69.3	1/second	Assumed to be very fast	Rate constant defining the half-life of expression of receptors on mAPCs that are part of the mAPC count
**Exp_CD28/80/86/PD1/L1/L2**	2.772	1/minute	[31, 48]	Rate constant defining the half-life of expression of CD28, CD80, CD86, PD-1, PD-L1 and PD-L2 to the immunological synapse by all interacting T cells, mAPCs and cancer cells
**Exp_CTLA4**	0.0462	1/minute	[49]	Rate constant defining the half-life of expression of CTLA-4 to the immunological synapse by all interacting T cells
**f_LN_CTLA4**	0.2	dimensionless	[50]	Assume that due to high cell numbers and packing, that the LN only allows for 3% interstitial space for antibody transport.
**f_LN_PD1**	0.1	dimensionless	[50]	Assume that due to high cell numbers and packing, that the LN only allows for 3% interstitial space for antibody transport.
**Frac_CD80_Exp_Cancer**	Set value	dimensionless	Varied parameter	Fraction form of %CD80_Exp_Cancer
**Frac_PD1_Exp_Cancer**	Set value	dimensionless	Varied parameter	Fraction form of %PD1_Exp_Cancer
**Frac_PDL1_Exp_Cancer**	Set value	dimensionless	Varied parameter	Fraction form of %PDL1_Exp_Cancer
**Frac_PDL2_Exp_Cancer**	Set value	dimensionless	Varied parameter	Fraction form of %PDL2_Exp_Cancer
**Frac_Vv_Tmr**	0.07	dimensionless	[13]	Fraction of vascular of total Tumor
**gamma_Sig**	3	dimensionless	Set for RO-based dose response potency	Gamma for total CTLA4 signaling in Tumor
**gamma_Sig1**	7	dimensionless	Set for RO-based dose response potency	Gamma for total CD80/PD1/PDL1 total signaling in Tumor

Variable	Value	Units	Source	Description
**gamma_SigTr**	3	dimensionless	Set for RO-based dose response potency	Gamma for total CD80/PD1/PDL1 total signaling in Lymph Node
**gamma_TC_Assoc**	0.9	dimensionless	[51]	Potency of Effector engagement/killing between Effector and target cells
**Ipilimumab_MW**	148634914	mg/mole	[52, 53]	Molecular weight of Anti-CTLA-4 antibody, Ipilimumab
**IS_Scaling**	2	dimensionless	[54, 55]	Sets the immunological synapse diameter to 30nm, from 15nm.
**ISF**	15.9	liter	[56]	Volume of interstitial fluid (ISF) is tissues
**J_Liver**	0.0029	1/minute	[13]	Effector T cell transmigration rate in Liver
**J_Lungs**	0.0029	1/minute	[13]	Effector T cell transmigration rate in Lungs
**J_Spleen**	0.0029	1/minute	[13]	Effector T cell transmigration rate in Spleen
**J_Tmr**	0.0029	1/minute	[13]	Effector T cell transmigration rate in Tumor
**k__DoseAdmin_AntiCTLA4**	0.6666666	1/hour	[15]	Zero-order rate constant for the delivery of Anti-CTLA-4 into the central compartment for a designated dose
**k__DoseAdmin_AntiPDL1**	1	1/hour	[26, 27]	Zero-order rate constant for the delivery of Anti-PD-L1 into the central compartment for a designated dose
**k_DoseAdmin_AntiPD1**	1	1/hour	[25]	Zero-order rate constant for the delivery of Anti-PD-1 into the central compartment for a designated dose
**kf_APC_turnover**	0.462	1/day	[57]	Half-life of mAPC turnover in the lymph nodes
**kf_CanDecay**	0.001	1/day	[34, 35]	Rate constant defining the half-life decay of cancer cells by natural death
**kf_Liver**	7.6E-9	ml/cell/minute	[13]	Attachment rate for free Effector T cells in Liver vasculature
**kf_Lungs**	0.0000031	ml/cell/minute	[13]	Attachment rate for free Effector T cells in Lung vasculature
**kf_Monocytes_intoT**	0.0231	1/minute	[58]	Rate constant defining the half-life of the appearance of monocytes in the tumor following the appearance of tumor antigens
**kf_no_prolif**	99.72	1/day	Estimated in proportion to cells dividing 3 times a day for about 1 week as baseline	Rate constant defining the half-life for the fraction of Primed Naive T cells that will not successfully undergo the second phase of priming, and will therefore be considered anergic
**kf_Phase2P**	0.1155	1/hour	[59-61]	Rate constant defining the approximate half-life of the entire second phase of priming in the lymph nodes, considering that the Primed Naïve T cells are\in the deep T-cell areas in the lymph node. This, along with the second priming phase, accounts for T cells starting to proliferate 1.5-2 days following the encounter of antigens on mAPCs [61].
**kf_Prolif_end**	15000	1/day	[19, 60, 62]	Rate constant defining the half-life for conversion of proliferating Naive T cells into Effector T cells in the lymph nodes. The T cells were optimized to account for 3 divisions per day over 5 days.
**kf_RestingMacrophage**	0.01	1/day	[34, 41]	Rate constant defining the half-life for turnover of resting macrophages
**kf_Spleen**	0.0000081	ml/cell/minute	[13]	Attachment rate for free Effector T cells in Spleen vasculature
**kf_Tmr**	6.9E-9	ml/cell/minute	[13]	Attachment rate for free Effector T cells in Tumor vasculature
**kf_TRecover**	0.1386	1/minute	Estimated	Rate constant defining the half-life of Effector T cell recovery following their interaction with cancer cells in the tumor
**kg**	1	kilogram	Unit assignment	Assigns value of 1 kilogram to the term kg in the model
**Km**	0.344	µg/ml	[63]	EC50 Durvalumab saturation for ADA
**koff_CD28_CD80**	1.6	1/second	[64]	Dissociation rate constant for the interaction of CD28 with CD80
**koff_CD28_CD86**	28	1/second	[64]	Dissociation rate constant for the interaction of CD28 with CD86
**koff_CTLA4_CD80**	0.43	1/second	[64]	Dissociation rate constant for the interaction of CTLA-4 with CD80

Variable	Value	Units	Source	Description
**koff_CTLA4_CD86**	5.1	1/second	[64]	Dissociation rate constant for the interaction of CTLA-4 with CD86
**koff_CTLA4mAb_CTLA4**	0.3	1/second	[65]	Dissociation rate constant for the interaction of CTLA-4 with Anti-CTLA-4 (considering a Kd of 5.25+/-3.62 nM)
**koff_PD1_PDL1**	1.44	1/second	[66]	Dissociation rate constant for the interaction of PD-1 with PD-L1
**koff_PD1_PDL2**	0.55	1/second	[66]	Dissociation rate constant for the interaction of PD-1 with PD-L2
**koff_PD1-PD1mAb**	0.000768	1/second	[67]	Dissociation rate constant for the interaction of PD-1 with Anti-PD-1
**koff_PDL1_CD80**	5.94	1/second	[66]	Dissociation rate constant for the interaction of PD-L1 with CD80
**koff_PDL1-PDL1mAb**	0.000025	1/second	[26]	Dissociation rate constant for the interaction of PD-L1 with Anti-PD-L1 (considering a Kd of 100 pM)
**kon_CD28_CD80**	660000	1/(molarity*second)	[64]	Association rate constant for the interaction of CD28 with CD80
**kon_CD28_CD86**	1400000	1/(molarity*second)	[64]	Association rate constant for the interaction of CD28 with CD86
**kon_CTLA4_CD80**	2150000	1/(molarity*second)	[64]	Association rate constant for the interaction of CTLA-4 with CD80
**kon_CTLA4_CD86**	1960000	1/(molarity*second)	[64]	Association rate constant for the interaction of CTLA-4 with CD86
**kon_CTLA4mAb_CTLA4**	40900000	1/(molarity*second)	[65]	Association rate constant for the interaction of CTLA-4 with Anti-CTLA-4 (considering a Kd of 5.25+/-3.62 nM)
**kon_PD1_PDL1**	184000	1/(molarity*second)	[66]	Association rate constant for the interaction of PD-1 with PD-L1
**kon_PD1_PDL2**	250000	1/(molarity*second)	[66]	Association rate constant for the interaction of PD-1 with PD-L2
**kon_PD1-PD1mAb**	250000	1/(molarity*second)	[67]	Association rate constant for the interaction of PD-1 with Anti-PD-1
**kon_PDL1_CD80**	316000	1/(molarity*second)	[66]	Association rate constant for the interaction of PD-L1 with CD80
**kon_PDL1-PDL1mAb**	250000	1/(molarity*second)	[26]	Association rate constant for the interaction of PD-L1 with Anti-PD-L1 (considering a Kd of 100 pM)
**KP_CTLA4**	0.8	dimensionless	[56]	Available fraction of ISF for antibody distribution
**KP_PD1**	0.4	dimensionless	[56]	Available fraction of ISF for antibody distribution
**KP_PDL1**	0.4	dimensionless	[56]	Available fraction of ISF for antibody distribution
**Kpa_LNB**	9.87E-8	cm/second	[68]	Transport of antibody between Serum and Lymph Node
**Kpa_TB**	9.86E-8	cm/second	[68]	Transport of antibody between Serum and Tumor
**kr_Lungs**	2.3	1/second	[13]	Detachment rate of Effector T cells in the Lung vasculature
**kr_Periph**	3.3	1/second	[13]	Detachment rate of Effector T cells in the Peripheral vasculature
**kr_Tmr**	3.3	1/second	[13]	Detachment rate of Effector T cells in the Tumor vasculature
**L**	1	liter	Unit assignment	Assigns value of 1 liter to the term L in the model
**LC_GI**	0.3	ml/minute	[13]	Lymph flow rate of GI
**LC_Liver**	0.087	ml/minute	[13]	Lymph flow rate of Liver
**LC_LN**	0.77	ml/minute	[13]	Lymph flow rate of Lymph Node
**LC_Lungs**	0.043	ml/minute	[13]	Lymph flow rate of Lungs
**LC_Periph**	0.369	ml/minute	[13]	Lymph flow rate of Peripheral tissues
**LC_Spleen**	0.00087	ml/minute	[13]	Lymph flow rate of Spleen
**LC_Tmr**	0.03	ml/minute	[13]	Lymph flow rate of Tumor
**mAPC_activation_level**	1 (unless varied)	dimensionless	[13]	The fraction of CD80 and CD86 expression on the mAPCs from 0-1.
**mAPC_Debis_T_Inact**	0.05-0.95	dimensionless	Varied across range (5-95%) and estimated during fitting to anti-PD-1 data	The fraction of tumor debris that is considered to not be transported to the lymph nodes, and is removed from the system
**mAPC_Migrate**	0.2	1/day	[34, 41, 69]	Maximum migration rate of mAPCs
**mAPC_per_T_cell**	10	cell/cell	[19, 70-72]	Maximum number of mAPCs that can interact with each Naive and Primed Naive T cell. Note: all T cells were estimated to be the same size, and a surface area analysis was used for this estimation.

Variable	Value	Units	Source	Description
**mAPC_per_Tr_cell**	10	cell/cell	[19, 70-72]	Maximum number of mAPCs that can interact with each Effector T cell. Note: all T cells were estimated to be the same size, and a surface area analysis was used for this estimation.
**mAPC50_per_T_cell**	3	cell/cell	[40]	Number of mAPCs that should interact with each Naive and Primed Naive T cell to achieve a 50% priming rate
**MDSCs_per_Treg**	3	cell/cell	[73]	The ratio of MDSCs per T Regulatory cells in the tumor
**mg**	1	milligram	Unit assignment	Assigns value of 1 miligram to the term mg in the model
**mole**	1	mole	Unit assignment	Assigns value of 1 mole to the term mole in the model
**New_sched_CTLA4**	Based on regimen	day	[15] Based on regimen	New time between sequential doses of Anti-CTLA-4 in a regimen
**New_sched_PD1**	Based on regimen	day	[25] Based on regimen	New time between sequential doses of Anti-PD-1 in a regimen
**New_sched_PDL1**	Based on regimen	day	[26, 27] Based on regimen	New time between sequential doses of Anti-PD-L1 in a regimen
**Nivolumab_MW**	143600000	mg/mole	[36-38, 74]	Molecular weight of Anti-PD-1 antibody, Nivolumab
**Num_TDLN_Considered**	35	dimensionless	[75-78] Estimated	The number of total different lymph nodes (or lobules receiving a variety of antigens) in a region near the tumor (can be >9cm) that can produce an immune response against the tumor.
**PD1_change_schedule**	Based on regimen	day	[25] Based on regimen	Time in simulation to switch schedule in regimen for Anti-PD-1 therapy
**PD1_counter_off**	Based on regimen	day	[25] Based on regimen	Time in simulation to stop Anti-PD-1 dose
**PD1_counter_on**	Based on regimen	day	[25] Based on regimen	Time in simulation to start Anti-PD-1 dose
**PD1_DoseSet**	0-10	mg/kg	Based on regimen	Dose administered of anti-PD-1 therapy
**PD1_PNT_NEG-to-Max**	Calculated	dimensionless	Calculated with RO; see algebraic equations below	Fraction of total PD-1 receptors on Primed Naïve T cells that are involved in interacting with the PD-L1 and PD-L2 receptors on mAPCs during priming in the lymph nodes
**PD1_receptors_per_C_Cl**	9288	molecules/cell	Assumed same as on T cells	Number of PD-1 receptors expressed on each cancer cell
**PD1_receptors-per-mAPC**	9288	molecules/cell	Assumed same as on T cells	Maximum number of PD-1 receptors expressed on each mAPC (maximum taken as max from mAPC levels)
**PD1_receptors-per-Tcell**	3096	molecules/cell	[66]	Maximum number of PD-1 receptors expressed on each T cell
**PD1mAb**	Based on regimen	milligram/kilogram	[25] Based on regimen	Dose of Anti-PD-1 being delivered
**PD1mAb_Dose**	Based on regimen	milligram/kilogram	[25] Based on regimen	Dose of Anti-PD-1 to deliver based on the current schedule
**PD1mAb_New_Dose**	Based on regimen	milligram/kilogram	[25] Based on regimen	Dose of Anti-PD-1 to be delivered when schedule is changed
**PD1Sig_Tr-PNT**	Calculated	dimensionless	Calculated with RO; see algebraic equations below	Fraction of total PD-1 receptors on Primed Naïve T cells that are involved in interacting with the PD-L1 receptors on Regulatory T cells during priming in the lymph nodes
**PDL1_change_schedule**	Based on regimen	day	[26, 27] Based on regimen	Time in simulation to switch schedule in regimen for Anti-PD-L1 therapy
**PDL1_counter_off**	Based on regimen	day	[26, 27] Based on regimen	Time in simulation to stop Anti-PD-L1 dose
**PDL1_counter_on**	Based on regimen	day	[26, 27] Based on regimen	Time in simulation to start Anti-PD-L1 dose
**PDL1_DoseSet**	0-20	mg/kg	Based on regimen	Dose administered of anti-PD-L1 therapy
**PDL1_PNT_NEG-to-Max**	Calculated	dimensionless	Calculated with RO; see algebraic equations below	Fraction of total PD-L1 receptors on Primed Naïve T cells that are involved in interacting with the PD-1 and CD80 receptors on mAPCs during priming in the lymph nodes
**PDL1_receptors_per_C_Cl**	80372	molecules/cell	[66]	Number of PD-L1 receptors expressed on each cancer cell (maximum taken as max from mAPC levels)
**PDL1_receptors-per-mAPC**	80372	molecules/cell	[66]	Maximum number of PD-L1 receptors expressed on each mAPC

Variable	Value	Units	Source	Description
**PDL1_receptors-per-Tcell**	9282	molecules/cell	[66]	Maximum number of PD-L1 receptors expressed on each T cell
**PDL1mAb**	Based on regimen	milligram/kilogram	[26, 27] Based on regimen	Dose of Anti-PD-L1 being delivered
**PDL1mAb_Dose**	Based on regimen	milligram/kilogram	[26, 27] Based on regimen	Dose of Anti-PD-L1 to deliver based on the current schedule
**PDL1mAb_New_Dose**	Based on regimen	milligram/kilogram	[26, 27] Based on regimen	Dose of Anti-PD-L1 to be delivered when schedule is changed
**PDL1Sig_Tr-PNT**	Calculated	dimensionless	Calculated; see algebraic equations below	Fraction of total PD-L1 receptors on Primed Naïve T cells that are involved in interacting with the PD-1 receptors on Regulatory T cells during priming in the lymph nodes
**PDL2_receptors_per_C_Cl**	5243	molecules/cell	[66]	Number of PD-L2 receptors expressed on each cancer cell (maximum taken as max from mAPC levels)
**PDL2_receptors-per-mAPC**	5243	molecules/cell	[66]	Maximum number of PD-L2 receptors expressed on each mAPC
**Phago_Debris**	1E-7	1/(mole*day)	[34, 41]	Tumor antigen uptake rate by APCs
**Precursor_Frequen_NT**	Calculated	dimensionless	[72, 79]	Calculates the ratio of available Naive T cells for priming phase 1 to total T cells in the lymph nodes
**Precursor_Frequen_PNT**	Calculated	dimensionless	[72, 79]	Calculates the ratio of available Primed Naive T cells for priming phase 2 to total T cells in the lymph nodes
**PrimeNT_rate**	0.05775	1/hour	[59-61]	Rate constant defining the approximate half-life of the entire first phase of priming in the lymph nodes, including the priming of all of the superficial Naïve T cells and those in the deep T-cell areas in the lymph node. This, along with the second priming phase, accounts for T cells starting to proliferate 1.5-2 days following the encounter of antigens on mAPCs [61].
**PrimeNT1_rate**	2.772	1/hour	[59, 60, 80]	Rate constant defining the approximate half-life of each interaction between Naive T cells and mAPCs during the first phase of priming in the lymph nodes, including the formation time of mature synapses between the Naïve T cells and the mAPCs.
**PrimeTLN2_rate**	1.386	1/hour	[59, 60]	Rate constant defining the approximate half-life of each interaction between Primed Naive T cells and mAPCs during the second phase of priming in the lymph nodes, which was dominated by stable T-cell-mAPC interactions.
**Prob_NT-mAPC_Interact**	Calculated	dimensionless	[72]	Calculates the probability in a deterministic manner that at least one successful priming interaction will occur between Naive T cells and mAPCs during the first phase of priming
**Prob_PNT-mAPC_Interact**	Calculated	dimensionless	[72]	Calculates the probability in a deterministic manner that at least one successful priming interaction will occur between Primed Naive T cells and mAPCs during the second phase of priming
**Prolif_Fract**	718.30381081261	1/hour	[19, 60, 62]	Proliferation threshold for fully primed and activated T cells in the lymph nodes that are undergoing proliferation. The T cells were optimized to account for 3 divisions per day over 5 days.
**Prolif_Thresh**	93.32288149744991	1/hour	[19, 60, 62]	Proliferation level for fully primed and activated T cells in the lymph nodes that are undergoing proliferation. The T cells were optimized to account for 3 divisions per day over 5 days.
**Q_L**	8.000498419	L/day	[56]	Lymph flow rate for antibody transport
**QC_GI**	468	ml/minute	[13]	Blood flow rate in GI
**QC_Liver**	800	ml/minute	[13]	Blood flow rate in Liver
**QC_LN**	138	ml/minute	[13]	Blood flow rate in Lymph Node
**QC_Lungs**	3000	ml/minute	[13]	Blood flow rate in Lungs
**QC_Periph**	1521	ml/minute	[13]	Blood flow rate in Periphery
**QC_Spleen**	138	ml/minute	[13]	Blood flow rate in Spleen
**QC_Tmr**	0.564	ml/minute	[13]	Blood flow rate in Tumor

Variable	Value	Units	Source	Description
**Rate_Tumor_Growth**	64/144 day doubling: see description	1/day	[81]	Proliferation rate of cancer cells that defines the volumetric doubling time of the tumor. 64 day metastatic doubling: 0.010955675075414 144 day non-metastatic doubling: 0.004913635262592
**s_Assoc**	5.27	Dimensionless	[51]	Effector:Target cell association/killing EC50
**S_LNB**	166	cm^2/cm^3	[68]	Surface are of vasculature per volume Lymph Node for antibody transport
**S_TB**	108	cm^2/cm^3	[68]	Surface are of vasculature per volume Tumor for antibody transport
**Sig_MDSCTeff = Other**	0.15	dimensionless	Assumed	Fraction of inhibitory effect that other factors expressed by MDSCs have on the Effector T cells in the tumor
**Sig_MDSCTeff=CD80**	Calculated	dimensionless	Calculated with RO; see algebraic equations below	Fraction of CD80 receptors expressed on Effector T cells that are interacting with PD-L1 on the MDSCs in the lymph nodes
**Sig_MDSCTeff=PD1**	Calculated	dimensionless	Calculated with RO; see algebraic equations below	Fraction of PD-1 receptors expressed on Effector T cells that are interacting with PD-L1 on the MDSCs in the lymph nodes
**Sig_MDSCTeff=PDL1**	Calculated	dimensionless	Calculated with RO; see algebraic equations below	Fraction of PD-L1 receptors expressed on Effector T cells that are interacting with PD-1 on the MDSCs in the lymph nodes
**Sig_MDSCTeff=Total**	Calculated	dimensionless	Calculated with RO; see algebraic equations below	Total inhibitory fraction that MDSCs have on the Effector T cells in the tumor following their interaction
**Sig_NT=CD28**	Calculated	dimensionless	Calculated with RO; see algebraic equations below	Positive CD28 co-receptor fraction signaling on Naive T cells by CD80 and CD86 on mAPC in the lymph nodes following their interaction
**Sig_PNT=CTLA4/PD1/L1/CD80**	Calculated	dimensionless	Calculated with RO; see algebraic equations below	Total inhibitory fraction that mAPCs have on the Effector T cells during priming following their interaction
**Sig_T=Other**	0.15	dimensionless	Calculated with RO; see algebraic equations below	Fraction of inhibitory effect that other factors expressed by all cancer cells have on the Effector T cells in the tumor
**Sig_T1=Total**	Calculated	dimensionless	Calculated with RO; see algebraic equations below	Total fraction of inhibitory effect that cancer cells expressing only other factors have on the Effector T cells in the tumor
**Sig_T10=Total**	Calculated	dimensionless	Calculated with RO; see algebraic equations below	Total fraction of inhibitory effect that cancer cells expressing PD-1, PD-L1, PD-L2 and other factors have on the Effector T cells in the tumor
**Sig_T10a=PDL1**	Calculated	dimensionless	Calculated with RO; see algebraic equations below	Fraction of PD-L1 receptors on Effector T cells that are occupied by PD-1 expressed on the T10 subset of cancer cells in the tumor
**Sig_T10b=PD1**	Calculated	dimensionless	Calculated with RO; see algebraic equations below	Fraction of PD-1 receptors on Effector T cells that are occupied by PD-L1 and PD-L2 expressed on the T10 subset of cancer cells in the tumor
**Sig_T10c=CD80**	Calculated	dimensionless	Calculated with RO; see algebraic equations below	Fraction of CD80 receptors on Effector T cells that are occupied by PD-L1 expressed on the T10 subset of cancer cells in the tumor
**Sig_T11=Total**	Calculated	dimensionless	Calculated with RO; see algebraic equations below	Total fraction of inhibitory effect that cancer cells expressing PD-L1, CD80 and other factors have on the Effector T cells in the tumor
**Sig_T11a=PDL1**	Calculated	dimensionless	Calculated with RO; see algebraic equations below	Fraction of PD-L1 receptors on Effector T cells that are occupied by CD80 expressed on the T11 subset of cancer cells in the tumor
**Sig_T11b=PD1**	Calculated	dimensionless	Calculated with RO; see algebraic equations below	Fraction of PD-1 receptors on Effector T cells that are occupied by PD-L1 expressed on the T11 subset of cancer cells in the tumor
**Sig_T12=Total**	Calculated	dimensionless	Calculated with RO; see algebraic equations below	Total fraction of inhibitory effect that cancer cells expressing PD-L2, CD80 and other factors have on the Effector T cells in the tumor
**Sig_T12a=PDL1**	Calculated	dimensionless	Calculated with RO; see algebraic equations below	Fraction of PD-L1 receptors on Effector T cells that are occupied by CD80 expressed on the T12 subset of cancer cells in the tumor
**Sig_T12b=PD1**	Calculated	dimensionless	Calculated with RO; see algebraic equations below	Fraction of PD-1 receptors on Effector T cells that are occupied by PD-L2 expressed on the T12 subset of cancer cells in the tumor

Variable	Value	Units	Source	Description
**Sig_T13=Total**	Calculated	dimensionless	Calculated with RO; see algebraic equations below	Total fraction of inhibitory effect that cancer cells expressing PD-L1, PD-L2, CD80 and other factors have on the Effector T cells in the tumor
**Sig_T13a=PDL1**	Calculated	dimensionless	Calculated with RO; see algebraic equations below	Fraction of PD-L1 receptors on Effector T cells that are occupied by CD80 expressed on the T13 subset of cancer cells in the tumor
**Sig_T13b=PD1**	Calculated	dimensionless	Calculated with RO; see algebraic equations below	Fraction of PD-1 receptors on Effector T cells that are occupied by PD-L1 and PD-L2 expressed on the T13 subset of cancer cells in the tumor
**Sig_T14=Total**	Calculated	dimensionless	Calculated with RO; see algebraic equations below	Total fraction of inhibitory effect that cancer cells expressing PD-1, PD-L1, CD80 and other factors have on the Effector T cells in the tumor
**Sig_T14a=PDL1**	Calculated	dimensionless	Calculated with RO; see algebraic equations below	Fraction of PD-L1 receptors on Effector T cells that are occupied by CD80 and PD-1 expressed on the T14 subset of cancer cells in the tumor
**Sig_T14b=PD1**	Calculated	dimensionless	Calculated with RO; see algebraic equations below	Fraction of PD-1 receptors on Effector T cells that are occupied by PD-L1 expressed on the T14 subset of cancer cells in the tumor
**Sig_T14c=CD80**	Calculated	dimensionless	Calculated with RO; see algebraic equations below	Fraction of CD80 receptors on Effector T cells that are occupied by PD-L1 expressed on the T14 subset of cancer cells in the tumor
**Sig_T15=Total**	Calculated	dimensionless	Calculated with RO; see algebraic equations below	Total fraction of inhibitory effect that cancer cells expressing PD-1, PD-L2, CD80 and other factors have on the Effector T cells in the tumor
**Sig_T15a=PDL1**	Calculated	dimensionless	Calculated with RO; see algebraic equations below	Fraction of PD-L1 receptors on Effector T cells that are occupied by CD80 and PD-1 expressed on the T15 subset of cancer cells in the tumor
**Sig_T15b=PD1**	Calculated	dimensionless	Calculated with RO; see algebraic equations below	Fraction of PD-1 receptors on Effector T cells that are occupied by PD-L2 expressed on the T15 subset of cancer cells in the tumor
**Sig_T16=Total**	Calculated	dimensionless	Calculated with RO; see algebraic equations below	Total fraction of inhibitory effect that cancer cells expressing PD-1, PD-L1, PD-L2, CD80 and other factors have on the Effector T cells in the tumor
**Sig_T16a=PDL1**	Calculated	dimensionless	Calculated with RO; see algebraic equations below	Fraction of PD-L1 receptors on Effector T cells that are occupied by CD80 and PD-1 expressed on the T16 subset of cancer cells in the tumor
**Sig_T16b=PD1**	Calculated	dimensionless	Calculated with RO; see algebraic equations below	Fraction of PD-1 receptors on Effector T cells that are occupied by PD-L1 and PD-L2 expressed on the T16 subset of cancer cells in the tumor
**Sig_T16c=CD80**	Calculated	dimensionless	Calculated with RO; see algebraic equations below	Fraction of CD80 receptors on Effector T cells that are occupied by PD-L1 expressed on the T16 subset of cancer cells in the tumor
**Sig_T2=PDL1**	Calculated	dimensionless	Calculated with RO; see algebraic equations below	Fraction of PD-L1 receptors on Effector T cells that are occupied by PD-1 expressed on the T2 subset of cancer cells in the tumor
**Sig_T2=Total**	Calculated	dimensionless	Calculated with RO; see algebraic equations below	Total fraction of inhibitory effect that cancer cells expressing PD-1 and other factors have on the Effector T cells in the tumor
**Sig_T3=Total**	Calculated	dimensionless	Calculated with RO; see algebraic equations below	Total fraction of inhibitory effect that cancer cells expressing PD-L1 and other factors have on the Effector T cells in the tumor
**Sig_T3a=PD1**	Calculated	dimensionless	Calculated with RO; see algebraic equations below	Fraction of PD-1 receptors on Effector T cells that are occupied by PD-L1 expressed on the T3 subset of cancer cells in the tumor
**Sig_T3b=CD80**	Calculated	dimensionless	Calculated with RO; see algebraic equations below	Fraction of CD80 receptors on Effector T cells that are occupied by PD-L1 expressed on the T3 subset of cancer cells in the tumor
**Sig_T4=PD1**	Calculated	dimensionless	Calculated with RO; see algebraic equations below	Fraction of PD-1 receptors on Effector T cells that are occupied by PD-L2 expressed on the T4 subset of cancer cells in the tumor
**Sig_T4=Total**	Calculated	dimensionless	Calculated with RO; see algebraic equations below	Total fraction of inhibitory effect that cancer cells expressing PD-L2 and other factors have on the Effector T cells in the tumor
**Sig_T5=PDL1**	Calculated	dimensionless	Calculated with RO; see algebraic equations below	Fraction of PD-L1 receptors on Effector T cells that are occupied by CD80 expressed on the T5 subset of cancer cells in the tumor
**Sig_T5=Total**	Calculated	dimensionless	Calculated with RO; see algebraic equations below	Total fraction of inhibitory effect that cancer cells expressing CD80 and other factors have on the Effector T cells in the tumor

Variable	Value	Units	Source	Description
**Sig_T6=Total**	Calculated	dimensionless	Calculated with RO; see algebraic equations below	Total fraction of inhibitory effect that cancer cells expressing PD-1, PD-L1 and other factors have on the Effector T cells in the tumor
**Sig_T6a=PDL1**	Calculated	dimensionless	Calculated with RO; see algebraic equations below	Fraction of PD-L1 receptors on Effector T cells that are occupied by PD-1 expressed on the T6 subset of cancer cells in the tumor
**Sig_T6b=PD1**	Calculated	dimensionless	Calculated with RO; see algebraic equations below	Fraction of PD-1 receptors on Effector T cells that are occupied by PD-L1 expressed on the T6 subset of cancer cells in the tumor
**Sig_T6c=CD80**	Calculated	dimensionless	Calculated with RO; see algebraic equations below	Fraction of CD80 receptors on Effector T cells that are occupied by PD-L1 expressed on the T6 subset of cancer cells in the tumor
**Sig_T7=Total**	Calculated	dimensionless	Calculated with RO; see algebraic equations below	Total fraction of inhibitory effect that cancer cells expressing PD-1, PD-L2 and other factors have on the Effector T cells in the tumor
**Sig_T7a=PDL1**	Calculated	dimensionless	Calculated with RO; see algebraic equations below	Fraction of PD-L1 receptors on Effector T cells that are occupied by PD-1 expressed on the T7 subset of cancer cells in the tumor
**Sig_T7b=PD1**	Calculated	dimensionless	Calculated with RO; see algebraic equations below	Fraction of PD-1 receptors on Effector T cells that are occupied by PD-L2 expressed on the T7 subset of cancer cells in the tumor
**Sig_T8=Total**	Calculated	dimensionless	Calculated with RO; see algebraic equations below	Total fraction of inhibitory effect that cancer cells expressing PD-L1, PD-L2 and other factors have on the Effector T cells in the tumor
**Sig_T8a=PD1**	Calculated	dimensionless	Calculated with RO; see algebraic equations below	Fraction of PD-1 receptors on Effector T cells that are occupied by PD-L1 and PD-L2 expressed on the T8 subset of cancer cells in the tumor
**Sig_T8b=CD80**	Calculated	dimensionless	Calculated with RO; see algebraic equations below	Fraction of CD80 receptors on Effector T cells that are occupied by PD-L1 expressed on the T8 subset of cancer cells in the tumor
**Sig_T9=PDL1**	Calculated	dimensionless	Calculated with RO; see algebraic equations below	Fraction of PD-L1 receptors on Effector T cells that are occupied by PD-1 and CD80 expressed on the T9 subset of cancer cells in the tumor
**Sig_T9=Total**	Calculated	dimensionless	Calculated with RO; see algebraic equations below	Total fraction of inhibitory effect that cancer cells expressing PD-1, CD80 and other factors have on the Effector T cells in the tumor
**Sig_TrAPC = Other**	0	dimensionless	Calculated with RO; see algebraic equations below	Fraction of inhibitory effect that other factors expressed by T Regulatory cells have on the mAPCs in the tumor
**Sig_TrAPCT=CTLA4**	Calculated	dimensionless	Calculated with RO; see algebraic equations below	Fraction of CD80 and CD86 receptors on mAPCs that are occupied by CTLA-4 expressed on T Regulatory cells in the tumor
**Sig_TrAPCT=Total**	Calculated	dimensionless	Calculated with RO; see algebraic equations below	Total inhibitory fraction that T Regulatory cells have on the mAPCs in the tumor following their interaction
**Sig_TrPNT=PD1/L1/CD80**	Calculated	dimensionless	Calculated with RO; see algebraic equations below	Total inhibitory fraction that T Regulatory cells have on the Primed Naive T cells in the lymph nodes following their interaction
**Sig_TrTeff = Other**	0.15	dimensionless	Calculated with RO; see algebraic equations below	Fraction of inhibitory effect that other factors expressed by T Regulatory cells have on the Effector T cells in the tumor
**Sig_TrTeff=CD80**	Calculated	dimensionless	Calculated with RO; see algebraic equations below	Fraction of CD80 receptors expressed on Effector T cells that are interacting with PD-L1 on the T Regulatory cells in the lymph nodes
**Sig_TrTeff=PD1**	Calculated	dimensionless	Calculated with RO; see algebraic equations below	Fraction of PD-1 receptors expressed on Effector T cells that are interacting with PD-L1 on the T Regulatory cells in the lymph nodes
**Sig_TrTeff=PDL1**	Calculated	dimensionless	Calculated with RO; see algebraic equations below	Fraction of PD-L1 receptors expressed on Effector T cells that are interacting with PD-1 on the T Regulatory cells in the lymph nodes
**Sig_TrTeff=Total**	Calculated	dimensionless	Calculated with RO; see algebraic equations below	Total inhibitory fraction that T Regulatory cells have on the Effector T cells in the tumor following their interaction
**Sigma1_CTLA4**	0.996839823	dimensionless	[56]	Estimated leaky tissue reflection coefficient for anti-CTLA-4 therapy
**Sigma1_PD1**	0.912199893	dimensionless	[56]	Estimated leaky tissue reflection coefficient for anti-PD-1 therapy
**Sigma1_PDL1**	0.990145236	dimensionless	[56]	Estimated leaky tissue reflection coefficient for anti-PD-L1 therapy

Variable	Value	Units	Source	Description
**Sigma2_CTLA4**	0.97448852	dimensionless	[56]	Estimated tight tissue reflection coefficient for anti-CTLA-4 therapy
**Sigma2_PD1**	0.89230685	dimensionless	[56]	Estimated tight tissue reflection coefficient for anti-PD-1 therapy
**Sigma2_PDL1**	0.988702857	dimensionless	[56]	Estimated tight tissue reflection coefficient for anti-PD-L1 therapy
**SigmaL**	0.2	dimensionless	[56]	Lymphatic tissue reflection coefficient
**Sigmax_MDSCTeff=CD80**	0.033934378	dimensionless	Calculated with RO; see algebraic equations below	Maximum fraction of CD80 receptors expressed on Effector T cells that are interacting with PD-L1 on the MDSCs in the lymph nodes
**Sigmax_MDSCTeff=PD1**	0.073903164	dimensionless	Calculated with RO; see algebraic equations below	Maximum fraction of PD-1 receptors expressed on Effector T cells that are interacting with PD-L1 on the MDSCs in the lymph nodes
**Sigmax_MDSCTeff=PDL1**	0.028530339	dimensionless	Calculated with RO; see algebraic equations below	Maximum fraction of PD-L1 receptors expressed on Effector T cells that are interacting with PD-1 on the MDSCs in the lymph nodes
**Sigmax_NT=CD28**	0.839539945	dimensionless	Calculated with RO; see algebraic equations below	Maximum fraction of CD28 co-receptor interaction on Naive T cells by CD80 and CD86 on mAPC in the lymph nodes
**Sigmax_PNT=CD80**	0.013125	dimensionless	Calculated with RO; see algebraic equations below	Maximum fraction of total CD80 receptors on Primed Naïve T cells that can interact with the PD-L1 receptors on mAPCs during priming in the lymph nodes
**Sigmax_PNT=CTLA4**	0.564375	dimensionless	Calculated with RO; see algebraic equations below	Maximum fraction of total CTLA-4 receptors on Primed Naïve T cells that can interact with the CD80 and CD86 receptors on mAPCs during priming in the lymph nodes
**Sigmax_PNT=PD1**	0.02625	dimensionless	Calculated with RO; see algebraic equations below	Maximum fraction of total PD-1 receptors on Primed Naïve T cells that can interact with the PD-L1 and PD-L2 receptors on mAPCs during priming in the lymph nodes
**Sigmax_PNT=PDL1**	0.02625	dimensionless	Calculated with RO; see algebraic equations below	Maximum fraction of total PD-L1 receptors on Primed Naïve T cells that can interact with the PD-1 and CD80 receptors on mAPCs during priming in the lymph nodes
**Sigmax_T10a=PDL1**	0.033991727	dimensionless	Calculated with RO; see algebraic equations below	Maximum fraction of PD-L1 receptors on Effector T cells that are occupied by PD-1 expressed on the T10 subset of cancer cells in the tumor
**Sigmax_T10b=PD1**	0.24079052	dimensionless	Calculated with RO; see algebraic equations below	Maximum fraction of PD-1 receptors on Effector T cells that are occupied by PD-L1 and PD-L2 expressed on the T10 subset of cancer cells in the tumor
**Sigmax_T10c=CD80**	0.106540763	dimensionless	Calculated with RO; see algebraic equations below	Maximum fraction of CD80 receptors on Effector T cells that are occupied by PD-L1 expressed on the T10 subset of cancer cells in the tumor
**Sigmax_T11a=PDL1**	0.048200501	dimensionless	Calculated with RO; see algebraic equations below	Maximum fraction of PD-L1 receptors on Effector T cells that are occupied by CD80 expressed on the T11 subset of cancer cells in the tumor
**Sigmax_T11b=PD1**	0.229183607	dimensionless	Calculated with RO; see algebraic equations below	Maximum fraction of PD-1 receptors on Effector T cells that are occupied by PD-L1 expressed on the T11 subset of cancer cells in the tumor
**Sigmax_T12a=PDL1**	0.048200501	dimensionless	Calculated with RO; see algebraic equations below	Maximum fraction of PD-L1 receptors on Effector T cells that are occupied by CD80 expressed on the T12 subset of cancer cells in the tumor
**Sigmax_T12b=PD1**	0.064134991	dimensionless	Calculated with RO; see algebraic equations below	Maximum fraction of PD-1 receptors on Effector T cells that are occupied by PD-L2 expressed on the T12 subset of cancer cells in the tumor
**Sigmax_T13a=PDL1**	0.048200501	dimensionless	Calculated with RO; see algebraic equations below	Maximum fraction of PD-L1 receptors on Effector T cells that are occupied by CD80 expressed on the T13 subset of cancer cells in the tumor
**Sigmax_T13b=PD1**	0.26059594	dimensionless	Calculated with RO; see algebraic equations below	Maximum fraction of PD-1 receptors on Effector T cells that are occupied by PD-L1 and PD-L2 expressed on the T13 subset of cancer cells in the tumor
**Sigmax_T14a=PDL1**	0.078099729	dimensionless	Calculated with RO; see algebraic equations below	Maximum fraction of PD-L1 receptors on Effector T cells that are occupied by CD80 and PD-1 expressed on the T14 subset of cancer cells in the tumor

Variable	Value	Units	Source	Description
**Sigmax_T14b=PD1**	0.206595509	dimensionless	Calculated with RO; see algebraic equations below	Maximum fraction of PD-1 receptors on Effector T cells that are occupied by PD-L1 expressed on the T14 subset of cancer cells in the tumor
**Sigmax_T14c=CD80**	0.106540762	dimensionless	Calculated with RO; see algebraic equations below	Maximum fraction of CD80 receptors on Effector T cells that are occupied by PD-L1 expressed on the T14 subset of cancer cells in the tumor
**Sigmax_T15a=PDL1**	0.078099729	dimensionless	Calculated with RO; see algebraic equations below	Maximum fraction of PD-L1 receptors on Effector T cells that are occupied by CD80 and PD-1 expressed on the T15 subset of cancer cells in the tumor
**Sigmax_T15b=PD1**	0.064134991	dimensionless	Calculated with RO; see algebraic equations below	Maximum fraction of PD-1 receptors on Effector T cells that are occupied by PD-L2 expressed on the T15 subset of cancer cells in the tumor
**Sigmax_T16a=PDL1**	0.078099729	dimensionless	Calculated with RO; see algebraic equations below	Maximum fraction of PD-L1 receptors on Effector T cells that are occupied by CD80 and PD-1 expressed on the T16 subset of cancer cells in the tumor
**Sigmax_T16b=PD1**	0.24079052	dimensionless	Calculated with RO; see algebraic equations below	Maximum fraction of PD-1 receptors on Effector T cells that are occupied by PD-L1 and PD-L2 expressed on the T16 subset of cancer cells in the tumor
**Sigmax_T16c=CD80**	0.106540763	dimensionless	Calculated with RO; see algebraic equations below	Maximum fraction of CD80 receptors on Effector T cells that are occupied by PD-L1 expressed on the T16 subset of cancer cells in the tumor
**Sigmax_T2=PDL1**	0.033991727	dimensionless	Calculated with RO; see algebraic equations below	Maximum fraction of PD-L1 receptors on Effector T cells that are occupied by PD-1 expressed on the T2 subset of cancer cells in the tumor
**Sigmax_T3a=PD1**	0.206595509	dimensionless	Calculated with RO; see algebraic equations below	Maximum fraction of PD-1 receptors on Effector T cells that are occupied by PD-L1 expressed on the T3 subset of cancer cells in the tumor
**Sigmax_T3b=CD80**	0.106540753	dimensionless	Calculated with RO; see algebraic equations below	Maximum fraction of CD80 receptors on Effector T cells that are occupied by PD-L1 expressed on the T3 subset of cancer cells in the tumor
**Sigmax_T4=PD1**	0.064134991	dimensionless	Calculated with RO; see algebraic equations below	Maximum fraction of PD-1 receptors on Effector T cells that are occupied by PD-L2 expressed on the T4 subset of cancer cells in the tumor
**Sigmax_T5=PDL1**	0.048200501	dimensionless	Calculated with RO; see algebraic equations below	Maximum fraction of PD-L1 receptors on Effector T cells that are occupied by CD80 expressed on the T5 subset of cancer cells in the tumor
**Sigmax_T6a=PDL1**	0.033991727	dimensionless	Calculated with RO; see algebraic equations below	Maximum fraction of PD-L1 receptors on Effector T cells that are occupied by PD-1 expressed on the T6 subset of cancer cells in the tumor
**Sigmax_T6b=PD1**	0.206595509	dimensionless	Calculated with RO; see algebraic equations below	Maximum fraction of PD-1 receptors on Effector T cells that are occupied by PD-L1 expressed on the T6 subset of cancer cells in the tumor
**Sigmax_T6c=CD80**	0.106540761	dimensionless	Calculated with RO; see algebraic equations below	Maximum fraction of CD80 receptors on Effector T cells that are occupied by PD-L1 expressed on the T6 subset of cancer cells in the tumor
**Sigmax_T7a=PDL1**	0.033991727	dimensionless	Calculated with RO; see algebraic equations below	Maximum fraction of PD-L1 receptors on Effector T cells that are occupied by PD-1 expressed on the T7 subset of cancer cells in the tumor
**Sigmax_T7b=PD1**	0.064134991	dimensionless	Calculated with RO; see algebraic equations below	Maximum fraction of PD-1 receptors on Effector T cells that are occupied by PD-L2 expressed on the T7 subset of cancer cells in the tumor
**Sigmax_T8a=PD1**	0.24079052	dimensionless	Calculated with RO; see algebraic equations below	Maximum fraction of PD-1 receptors on Effector T cells that are occupied by PD-L1 and PD-L2 expressed on the T8 subset of cancer cells in the tumor
**Sigmax_T8b=CD80**	0.104091855	dimensionless	Calculated with RO; see algebraic equations below	Maximum fraction of CD80 receptors on Effector T cells that are occupied by PD-L1 expressed on the T8 subset of cancer cells in the tumor
**Sigmax_T9=PDL1**	0.078099729	dimensionless	Calculated with RO; see algebraic equations below	Maximum fraction of PD-L1 receptors on Effector T cells that are occupied by PD-1 and CD80 expressed on the T9 subset of cancer cells in the tumor
**Sigmax_TrAPCT=CTLA4**	0.0008355	dimensionless	Calculated with RO; see algebraic equations below	Maximum fraction of CD80 and CD86 receptors on mAPCs that are occupied by CTLA-4 expressed on T Regulatory cells in the tumor

Variable	Value	Units	Source	Description
**Sigmax_TrPNT=CD80**	0.020305139	dimensionless	Calculated with RO; see algebraic equations below	Maximum fraction of total CD80 receptors on Primed Naïve T cells that are involved in interacting with the PD-L1 receptors on Regulatory T cells during priming in the lymph nodes
**Sigmax_TrPNT=Other**	1	dimensionless	Calculated with RO; see algebraic equations below	Maximum level of inhibitory effects against Primed Naive T cells by other factors expressed by T Regulatory cells
**Sigmax_TrPNT=PD1**	0.034241129	dimensionless	Calculated with RO; see algebraic equations below	Maximum fraction of total PD-1 receptors on Primed Naïve T cells that are involved in interacting with the PD-L1 receptors on Regulatory T cells during priming in the lymph nodes
**Sigmax_TrPNT=PDL1**	0.010435276	dimensionless	Calculated with RO; see algebraic equations below	Maximum fraction of total PD-L1 receptors on Primed Naïve T cells that are involved in interacting with the PD-1 receptors on Regulatory T cells during priming in the lymph nodes
**Sigmax_TrTeff=CD80**	0.033934413	dimensionless	Calculated with RO; see algebraic equations below	Maximum fraction of CD80 receptors expressed on Effector T cells that are interacting with PD-L1 on the T Regulatory cells in the tumor
**Sigmax_TrTeff=PD1**	0.073903236	dimensionless	Calculated with RO; see algebraic equations below	Maximum fraction of PD-1 receptors expressed on Effector T cells that are interacting with PD-L1 on the T Regulatory cells in the tumor
**Sigmax_TrTeff=PDL1**	0.028530371	dimensionless	Calculated with RO; see algebraic equations below	Maximum fraction of PD-L1 receptors expressed on Effector T cells that are interacting with PD-1 on the T Regulatory cells in the tumor
**Sigweight_MDSCTeff=CD80**	0.248844361	dimensionless	Calculated as fraction of RO per total RO; see algebraic equations below	Weight of Effector T cell inhibition by signaling through CD80 receptors expressed on Effector T cells that are interacting with PD-L1 on the MDSCs in the tumor
**Sigweight_MDSCTeff=PD1**	0.541939663	dimensionless	Calculated as fraction of RO per total RO; see algebraic equations below	Weight of Effector T cell inhibition by signaling through PD-1 receptors expressed on Effector T cells that are interacting with PD-L1 on the MDSCs in the tumor
**Sigweight_MDSCTeff=PDL1**	0.209215976	dimensionless	Calculated as fraction of RO per total RO; see algebraic equations below	Weight of Effector T cell inhibition by signaling through PD-L1 receptors expressed on Effector T cells that are interacting with PD-1 on the MDSCs in the tumor
**SigWeight_PNT=CD80**	0.020833333	dimensionless	Calculated as fraction of RO per total RO; see algebraic equations below	Weight of Primed Naive T cell inhibition by signaling through CD80 receptors on Primed Naïve T cells that can interact with the PD-L1 receptors on mAPCs during priming in the lymph nodes
**SigWeight_PNT=CTLA4**	0.895833333	dimensionless	Calculated as fraction of RO per total RO; see algebraic equations below	Weight of Primed Naive T cell inhibition by signaling through CTLA-4 receptors on Primed Naïve T cells that can interact with the CD80 and CD86 receptors on mAPCs during priming in the lymph nodes
**SigWeight_PNT=PD1**	0.041666667	dimensionless	Calculated as fraction of RO per total RO; see algebraic equations below	Weight of Primed Naive T cell inhibition by signaling through PD-1 receptors on Primed Naïve T cells that can interact with the PD-L1 and PD-L2 receptors on mAPCs during priming in the lymph nodes
**SigWeight_PNT=PDL1**	0.041666667	dimensionless	Calculated as fraction of RO per total RO; see algebraic equations below	Weight of Primed Naive T cell inhibition by signaling through PD-L1 receptors on Primed Naïve T cells that can interact with the PD-1 and CD80 receptors on mAPCs during priming in the lymph nodes
**Sigweight_T10a=PDL1**	0.089141557	dimensionless	Calculated as fraction of RO per total RO; see algebraic equations below	Weight of Effector T cell inhibition by signaling through PD-L1 receptors on Effector T cells that are occupied by PD-1 expressed on the T10 subset of cancer cells in the tumor
**Sigweight_T10b=PD1**	0.631460767	dimensionless	Calculated as fraction of RO per total RO; see algebraic equations below	Weight of Effector T cell inhibition by signaling through PD-1 receptors on Effector T cells that are occupied by PD-L1 and PD-L2 expressed on the T10 subset of cancer cells in the tumor

Variable	Value	Units	Source	Description
**Sigweight_T10c=CD80**	0.279397677	dimensionless	Calculated as fraction of RO per total RO; see algebraic equations below	Weight of Effector T cell inhibition by signaling through CD80 receptors on Effector T cells that are occupied by PD-L1 expressed on the T10 subset of cancer cells in the tumor
**Sigweight_T11a=PDL1**	0.173768069	dimensionless	Calculated as fraction of RO per total RO; see algebraic equations below	Weight of Effector T cell inhibition by signaling through PD-L1 receptors on Effector T cells that are occupied by CD80 expressed on the T11 subset of cancer cells in the tumor
**Sigweight_T11b=PD1**	0.826231931	dimensionless	Calculated as fraction of RO per total RO; see algebraic equations below	Weight of Effector T cell inhibition by signaling through PD-1 receptors on Effector T cells that are occupied by PD-L1 expressed on the T11 subset of cancer cells in the tumor
**Sigweight_T12a=PDL1**	0.429076332	dimensionless	Calculated as fraction of RO per total RO; see algebraic equations below	Weight of Effector T cell inhibition by signaling through PD-L1 receptors on Effector T cells that are occupied by CD80 expressed on the T12 subset of cancer cells in the tumor
**Sigweight_T12b=PD1**	0.570923668	dimensionless	Calculated as fraction of RO per total RO; see algebraic equations below	Weight of Effector T cell inhibition by signaling through PD-1 receptors on Effector T cells that are occupied by PD-L2 expressed on the T12 subset of cancer cells in the tumor
**Sigweight_T13a=PDL1**	0.156091504	dimensionless	Calculated as fraction of RO per total RO; see algebraic equations below	Weight of Effector T cell inhibition by signaling through PD-L1 receptors on Effector T cells that are occupied by CD80 expressed on the T13 subset of cancer cells in the tumor
**Sigweight_T13b=PD1**	0.843908496	dimensionless	Calculated as fraction of RO per total RO; see algebraic equations below	Weight of Effector T cell inhibition by signaling through PD-1 receptors on Effector T cells that are occupied by PD-L1 and PD-L2 expressed on the T13 subset of cancer cells in the tumor
**Sigweight_T14a=PDL1**	0.199623063	dimensionless	Calculated as fraction of RO per total RO; see algebraic equations below	Weight of Effector T cell inhibition by signaling through PD-L1 receptors on Effector T cells that are occupied by CD80 and PD-1 expressed on the T14 subset of cancer cells in the tumor
**Sigweight_T14b=PD1**	0.528058536	dimensionless	Calculated as fraction of RO per total RO; see algebraic equations below	Weight of Effector T cell inhibition by signaling through PD-1 receptors on Effector T cells that are occupied by PD-L1 expressed on the T14 subset of cancer cells in the tumor
**Sigweight_T14c=CD80**	0.272318401	dimensionless	Calculated as fraction of RO per total RO; see algebraic equations below	Weight of Effector T cell inhibition by signaling through CD80 receptors on Effector T cells that are occupied by PD-L1 expressed on the T14 subset of cancer cells in the tumor
**Sigweight_T15a=PDL1**	0.549090467	dimensionless	Calculated as fraction of RO per total RO; see algebraic equations below	Weight of Effector T cell inhibition by signaling through PD-L1 receptors on Effector T cells that are occupied by CD80 and PD-1 expressed on the T15 subset of cancer cells in the tumor
**Sigweight_T15b=PD1**	0.450909533	dimensionless	Calculated as fraction of RO per total RO; see algebraic equations below	Weight of Effector T cell inhibition by signaling through PD-1 receptors on Effector T cells that are occupied by PD-L2 expressed on the T15 subset of cancer cells in the tumor
**Sigweight_T16a=PDL1**	0.183577893	dimensionless	Calculated as fraction of RO per total RO; see algebraic equations below	Weight of Effector T cell inhibition by signaling through PD-L1 receptors on Effector T cells that are occupied by CD80 and PD-1 expressed on the T16 subset of cancer cells in the tumor
**Sigweight_T16b=PD1**	0.565991931	dimensionless	Calculated as fraction of RO per total RO; see	Weight of Effector T cell inhibition by signaling through PD-1 receptors on Effector T cells that are occupied by PD-L1 and PD-L2 expressed on the T16 subset of cancer cells in the tumor

Variable	Value	Units	Source	Description
			algebraic equations below	
**Sigweight_T16c=CD80**	0.250430176	dimensionless	Calculated as fraction of RO per total RO; see algebraic equations below	Weight of Effector T cell inhibition by signaling through CD80 receptors on Effector T cells that are occupied by PD-L1 expressed on the T16 subset of cancer cells in the tumor
**Sigweight_T2=PDL1**	1	dimensionless	Calculated as fraction of RO per total RO; see algebraic equations below	Weight of Effector T cell inhibition by signaling through PD-L1 receptors on Effector T cells that are occupied by PD-1 expressed on the T2 subset of cancer cells in the tumor
**Sigweight_T3a=PD1**	0.659762327	dimensionless	Calculated as fraction of RO per total RO; see algebraic equations below	Weight of Effector T cell inhibition by signaling through PD-1 receptors on Effector T cells that are occupied by PD-L1 expressed on the T3 subset of cancer cells in the tumor
**Sigweight_T3b=CD80**	0.340237673	dimensionless	Calculated as fraction of RO per total RO; see algebraic equations below	Weight of Effector T cell inhibition by signaling through CD80 receptors on Effector T cells that are occupied by PD-L1 expressed on the T3 subset of cancer cells in the tumor
**Sigweight_T4=PD1**	1	dimensionless	Calculated as fraction of RO per total RO; see algebraic equations below	Weight of Effector T cell inhibition by signaling through PD-1 receptors on Effector T cells that are occupied by PD-L2 expressed on the T4 subset of cancer cells in the tumor
**Sigweight_T5=PDL1**	1	dimensionless	Calculated as fraction of RO per total RO; see algebraic equations below	Weight of Effector T cell inhibition by signaling through PD-L1 receptors on Effector T cells that are occupied by CD80 expressed on the T5 subset of cancer cells in the tumor
**Sigweight_T6a=PDL1**	0.097922746	dimensionless	Calculated as fraction of RO per total RO; see algebraic equations below	Weight of Effector T cell inhibition by signaling through PD-L1 receptors on Effector T cells that are occupied by PD-1 expressed on the T6 subset of cancer cells in the tumor
**Sigweight_T6b=PD1**	0.595156574	dimensionless	Calculated as fraction of RO per total RO; see algebraic equations below	Weight of Effector T cell inhibition by signaling through PD-1 receptors on Effector T cells that are occupied by PD-L1 expressed on the T6 subset of cancer cells in the tumor
**Sigweight_T6c=CD80**	0.30692068	dimensionless	Calculated as fraction of RO per total RO; see algebraic equations below	Weight of Effector T cell inhibition by signaling through CD80 receptors on Effector T cells that are occupied by PD-L1 expressed on the T6 subset of cancer cells in the tumor
**Sigweight_T7a=PDL1**	0.346406438	dimensionless	Calculated as fraction of RO per total RO; see algebraic equations below	Weight of Effector T cell inhibition by signaling through PD-L1 receptors on Effector T cells that are occupied by PD-1 expressed on the T7 subset of cancer cells in the tumor
**Sigweight_T7b=PD1**	0.653593562	dimensionless	Calculated as fraction of RO of total ROs in signaling equation	Weight of Effector T cell inhibition by signaling through PD-1 receptors on Effector T cells that are occupied by PD-L2 expressed on the T7 subset of cancer cells in the tumor
**Sigweight_T8a=PD1**	0.698181576	dimensionless	Calculated as fraction of RO per total RO; see algebraic equations below	Weight of Effector T cell inhibition by signaling through PD-1 receptors on Effector T cells that are occupied by PD-L1 and PD-L2 expressed on the T8 subset of cancer cells in the tumor
**Sigweight_T8b=CD80**	0.301818424	dimensionless	Calculated as fraction of RO per total RO; see algebraic equations below	Weight of Effector T cell inhibition by signaling through CD80 receptors on Effector T cells that are occupied by PD-L1 expressed on the T8 subset of cancer cells in the tumor
**Sigweight_T9=PDL1**	1	dimensionless	Calculated as fraction of RO per	Weight of Effector T cell inhibition by signaling through PD-L1 receptors on Effector T cells that

Variable	Value	Units	Source	Description
			total RO; see algebraic equations below	are occupied by PD-1 and CD80 expressed on the T9 subset of cancer cells in the tumor
**SigWeight_TrPNT=CD80**	0.266666667	dimensionless	Calculated as fraction of RO per total RO; see algebraic equations below	Weight of Primed Naive T cell inhibition by signaling through CD80 receptors on Primed Naïve T cells that are involved in interacting with the PD-L1 receptors on Regulatory T cells during priming in the lymph nodes
**SigWeight_TrPNT=Other**	0.2	dimensionless	Calculated as fraction of RO per total RO; see algebraic equations below	Weight of of inhibitory effects against Primed Naive T cells by other factors expressed by T Regulatory cells
**SigWeight_TrPNT=PD1**	0.266666667	dimensionless	Calculated as fraction of RO per total RO; see algebraic equations below	Weight of Primed Naive T cell inhibition by signaling through PD-1 receptors on Primed Naïve T cells that are involved in interacting with the PD-L1 receptors on Regulatory T cells during priming in the lymph nodes
**SigWeight_TrPNT=PDL1**	0.266666667	dimensionless	Calculated as fraction of RO per total RO; see algebraic equations below	Weight of Primed Naive T cell inhibition by signaling through PD-L1 receptors on Primed Naïve T cells that are involved in interacting with the PD-1 receptors on Regulatory T cells during priming in the lymph nodes
**Sigweight_TrTeff=CD80**	0.248844363	dimensionless	Calculated as fraction of RO per total RO; see algebraic equations below	Weight of Effector T cell inhibition by signaling through CD80 receptors expressed on Effector T cells that are interacting with PD-L1 on the T Regulatory cells in the tumor
**Sigweight_TrTeff=PD1**	0.54193964	dimensionless	Calculated as fraction of RO per total RO; see algebraic equations below	Weight of Effector T cell inhibition by signaling through PD-1 receptors expressed on Effector T cells that are interacting with PD-L1 on the T Regulatory cells in the tumor
**Sigweight_TrTeff=PDL1**	0.209215997	dimensionless	Calculated as fraction of RO per total RO; see algebraic equations below	Weight of Effector T cell inhibition by signaling through PD-L1 receptors expressed on Effector T cells that are interacting with PD-1 on the T Regulatory cells in the tumor
**Starting_Cancer_Diam_mm**	15-80	mm	Set value; varied	The diameter of the tumor at which to start its growth in the simulation
**Success_NT-mAPC**	500	dimensionless	[60, 62, 72, 79, 82, 83]	The number of Naive T:mAPC interactions that would occur prior to a successful priming interaction
**Success_PNT-mAPC**	50	dimensionless	[60, 62, 72, 79, 82, 84]	The number of Primed Naive T:mAPC interactions that would occur prior to a successful priming interaction. The difference between the number of successful interactions prior to priming between the first and second stage is estimated by enhanced immunity through chemokines that attract the CD8+ T cells where the APCs and T cells have gathered in a more localized area during priming [84].
**T_Cell_Clonality**	10-130	cell	[28, 30, 85]	Number of Naïve T cells populations specific for different tumor antigens; in other words; the number of anti-tumor T cell clones in each lymph node.
**T_Cell_Diffusion**	10	$\mu m^2/min$	[62, 72, 79, 86]	The diffusion coefficient of Naive and Primed Naive T cells in the lymph nodes during priming
**T_cells_per_mAPC**	75	cell/cell	[19, 40, 71, 72, 87, 88]	The maximum number of Naïve and Primed Naïve T cells that can interact with an mAPC at once (surface area of APCs is ~900μm^2 as a sphere – from ~2500 μm^3 [88] per APC – and APC:T contact area is ~50μm^2[40], but have been shown to range from 1um^2 to >70um^2 and even as much as 50% of a cell's surface area [19]). Note: all T cells were estimated to be the same size, and a surface area analysis was used for this estimation. Furthermore, the available surface area on the

Variable	Value	Units	Source	Description
				mAPCs was multiplied by a factor of 3-4-fold [19] to consider dendritic projections, which would increase the surface area relative to the volume.
**T_Density**	Calculated	cell/μm^3	[72]	The density of total T cells in the lymph nodes
**T_IVinject**	1	hour	Can be varied	Injection time of antibody
**T_per_Cancer_Cell_Int**	1	cell/cell	Can be varied	The number of Effector T cells that interact with each cancer cell at once
**T_per_Cancer_Cell_max**	10	cell/cell	[71, 72]	The maximum number of Effector T cells that can interact with a cancer at once. Note: all T cells were estimated to be the same size, and a surface area analysis was used for this estimation.
**T_per_Tr_cell**	10	cell/cell	[71, 72, 89]	The maximum number of T (Naive, Primed Naive and Effector) cells that can interact with a T regulatory cell at once. Note: all T cells were estimated to be the same size, and a surface area analysis was used for this estimation.
**TCytokineHoming**	100	dimensionless	[90, 91]; estimated with fitting to PD1 monotherapy tumor response	The factor specifying homing of Effector T cells to extravasate into the tumor more so than into the peripheral tissues, or to remain in the blood
**TDLN_Radius**	6203.50490 9	μm	[72]	The radius of the lymph node region where T cell priming and interactions occur
**TheEnd**	1	dimensionless	Assigned	Factor that is 0 or 1, which stops the immune response when the tumor has died, former, or allows the immune response to be active, latter
**Time_Start_Therapy**	Calculated	day	Function of tumor diameter	Recorded time when therapy began
**Time_Tumor_Death**	Calculated	day	Function of tumor diameter	Recorded time when tumor was sufficiently killed by the immune response
**T-mAPC_Cell_Size**	12	μm	[72]	The diameter of a T cell undergoing priming
**T-mAPC_Inter_tmax_min**	1440	min	[72]	The maximum time (24 hours) over which interaction during each priming stage are considered.
**Total_T_per_TDLN**	5000000	cell	[3]	The total number of T cells in each lymph node
**Total_TC_Sig**	Calculated	dimensionless	Sum of all cancer checkpoint signaling modules	The total immune checkpoint inhibiting effect on the Effector T cells by the tumor
**Tr_cells_per_mAPC**	75	cell/cell	[19, 71, 72, 87, 88]	The maximum number of T Regulatory cells that can interact with a mAPC cell at once. Note: all T cells were estimated to be the same size, and an approximate surface area analysis was used for this estimation. Furthermore, the available surface area on the mAPCs was multiplied by a factor of 3-4-fold [19] to consider dendritic projections, which would increase the surface area relative to the volume.
**Tr_per_T_cell**	10	cell/cell	[71, 72, 89]	The maximum number of T Regulatory cells that can interact with a T (Naive, Primed Naive and Effector) cell at once. Note: all T cells were estimated to be the same size, and a surface area analysis was used for this estimation.
**Treg:mAPC_IntTime**	0.0924	1/minute	[89]	Rate constant defining the half-life of interaction between T Regulatory cells or MDSCs and mAPCs
**Treg:T_IntTime**	0.1386	1/minute	[89]	Rate constant defining the half-life of interaction between T Regulatory cells or MDSCs and T (Naive, Primed Naive and Effector) cells
**TregLN_Engage**	0.05775	1/(mole*hour)	[59-61]; assumed the same	Rate constant defining the half-life of engagement between T Regulatory cells and mAPCs or T (Naive, Primed Naive and Effector) cells
**TregTMDSCEng**	0.2079	1/hour	Assumed 0.1 of Effect:Target engagement time	Rate constant defining the half-life of engagement between mAPCs or Effector T cells, and T Regulatory cells or MDSCs in the tumor
**TrLN-mAPC_Cell_Size**	12	μm	[72, 89]	The diameter of a T Regulatory cell interacting with mAPCs
**TrLN-PNT_Cell_Size**	12	μm	[72, 89]	The diameter of a T Regulatory cell interacting with Primed Naive T cells
**Total_Tumor_Void_Frac**	0.57	dimensionless	[13, 16]	The fraction of tumor volume that is not occupied by cancer cells, consider vasculature

Variable	Value	Units	Source	Description
**Tumor_Void_Fraction**	0.5	dimensionless	[16]	The fraction of tumor volume that is not occupied by cancer cells, not consider vasculature
**Tumor_Vol_Ellipse_c_ratio**	0.5	dimensionless	[92]	The approximate shortest radius of the ellipsoid tumor
**V_T:C_10**	Calculated	liter	Calculated (cell SA times synapse diameter); see equations below	Volume of receptor interactions between Effector T cells and cancer cells in the T10 subgroup at the immunological synapse
**V_T:C_11**	Calculated	liter	Calculated (cell SA times synapse diameter); see equations below	Volume of receptor interactions between Effector T cells and cancer cells in the T11 subgroup at the immunological synapse
**V_T:C_12**	Calculated	liter	Calculated (cell SA times synapse diameter); see equations below	Volume of receptor interactions between Effector T cells and cancer cells in the T12 subgroup at the immunological synapse
**V_T:C_13**	Calculated	liter	Calculated (cell SA times synapse diameter); see equations below	Volume of receptor interactions between Effector T cells and cancer cells in the T13 subgroup at the immunological synapse
**V_T:C_14**	Calculated	liter	Calculated (cell SA times synapse diameter); see equations below	Volume of receptor interactions between Effector T cells and cancer cells in the T14 subgroup at the immunological synapse
**V_T:C_15**	Calculated	liter	Calculated (cell SA times synapse diameter); see equations below	Volume of receptor interactions between Effector T cells and cancer cells in the T15 subgroup at the immunological synapse
**V_T:C_16**	Calculated	liter	Calculated (cell SA times synapse diameter); see equations below	Volume of receptor interactions between Effector T cells and cancer cells in the T16 subgroup at the immunological synapse
**V_T:C_2**	Calculated	liter	Calculated (cell SA times synapse diameter); see equations below	Volume of receptor interactions between Effector T cells and cancer cells in the T2 subgroup at the immunological synapse
**V_T:C_3**	Calculated	liter	Calculated (cell SA times synapse diameter); see equations below	Volume of receptor interactions between Effector T cells and cancer cells in the T3 subgroup at the immunological synapse
**V_T:C_4**	Calculated	liter	Calculated (cell SA times synapse diameter); see equations below	Volume of receptor interactions between Effector T cells and cancer cells in the T4 subgroup at the immunological synapse
**V_T:C_5**	Calculated	liter	Calculated (cell SA times synapse diameter); see equations below	Volume of receptor interactions between Effector T cells and cancer cells in the T5 subgroup at the immunological synapse
**V_T:C_6**	Calculated	liter	Calculated (cell SA times synapse diameter); see equations below	Volume of receptor interactions between Effector T cells and cancer cells in the T6 subgroup at the immunological synapse
**V_T:C_7**	Calculated	liter	Calculated (cell SA times synapse diameter); see equations below	Volume of receptor interactions between Effector T cells and cancer cells in the T7 subgroup at the immunological synapse
**V_T:C_8**	Calculated	liter	Calculated (cell SA times synapse diameter); see equations below	Volume of receptor interactions between Effector T cells and cancer cells in the T8 subgroup at the immunological synapse
**V_T:C_9**	Calculated	liter	Calculated (cell SA times synapse diameter); see equations below	Volume of receptor interactions between Effector T cells and cancer cells in the T9 subgroup at the immunological synapse
**Vc_CTLA4**	2.727814034	liter	[15]	Volume of the central compartment for Anti-CTLA-4
**Vc_PD1**	2.716739643	liter	[25]	Volume of the central compartment for Anti-PD-1

Variable	Value	Units	Source	Description
**Vc_PDL1**	4.051599814	liter	[26, 27]	Volume of the central compartment for Anti-PD-L1
**Vc_Teff**	2.7	liter	[13, 93]	Volume of blood through which Effector T cells circulate throughout the body
**Vex_Tmr**	~50% (void space) of total tumor volume	ml	[13] Calculated dynamically	Volume of Tumor interstitium
**Vext_Liver**	361.8	ml	[13]	Volume of Liver interstitium
**Vext_LN**	34.7	ml	[13]	Volume of Lymph Node interstitium
**Vext_Lungs**	299.7	ml	[13]	Volume of Lungs interstitium
**Vext_Spleen**	34.7	ml	[13]	Volume of Spleen interstitium
**VL**	5.2	liter	[56]	Volume of Lymph
**Vm**	0.824	mg/day	[63]	Vmax for ADA saturation for Durvalumab
**Vol_Cell-Rec_MDSC-TeffT**	Calculated	liter	Calculated (cell SA times synapse diameter); see equations below	Volume of receptor interactions between Effector T cells and MDSCs at the immunological synapse in the tumor
**Vol_Cell-Rec_Tr-APCT**	Calculated	liter	Calculated (cell SA times synapse diameter); see equations below	Volume of receptor interactions between mAPCs and T Regulatory cells at the immunological synapse in the tumor
**Vol_Cell-Rec_Tr-mAPC**	Calculated	liter	Calculated (cell SA times synapse diameter); see equations below	Volume of receptor interactions between mAPCs and T Regulatory cells at the immunological synapse in the lymph nodes
**Vol_Cell-Rec_Tr-PNT**	Calculated	liter	Calculated (cell SA times synapse diameter); see equations below	Volume of receptor interactions between Primed Naive T cells and T Regulatory cells at the immunological synapse in the lymph nodes
**Vol_Cell-Rec_Tr-TeffT**	Calculated	liter	Calculated (cell SA times synapse diameter); see equations below	Volume of receptor interactions between Effector T cells and T Regulatory cells at the immunological synapse in the tumor
**Vol_per_TDLN**	1	ml	[94]	Volume of a single human lymph node (represented as a sphere) where the T cells reside and undergo priming for probability calculations
**Volume_NT-Receptor_Int**	Calculated	liter	Calculated (cell SA times synapse diameter); see equations below	Volume of receptor interactions between Naive T cells and mAPCs at the immunological synapse in the lymph nodes during priming
**Volume_PNT-Receptor_Int**	Calculated	liter	Calculated (cell SA times synapse diameter); see equations below	Volume of receptor interactions between Primed Naive T cells and mAPCs at the immunological synapse in the lymph nodes during priming
**Vt**	0.01	liter	Estimated	Volume of the tumor compartment for PK calculations
**Vt_avg_const**	33.5	cm^3	[68]	Average tumor volume considered diffusive transport
**Vtdln**	5.2	liter	[56, 94]	Volume of total lymph
**Vt_Teff**	Based on Cancer_Vol_cm3	liter	Calculated dynamically	Total tumor volume based on cancer cell number, void space and tumor vasculature
**Vv_GI**	43	ml	[13]	Vasculature volume of GI
**Vv_Liver**	180.9	ml	[13]	Vasculature volume of Liver
**Vv_LN**	17	ml	[13]	Vasculature volume of Lymph Node
**Vv_Lungs**	99.9	ml	[13]	Vasculature volume of Lungs
**Vv_Periph**	1355.4	ml	[13]	Vasculature volume of Peripheral for Effector T cell trafficking
**Vv_Spleen**	17	ml	[13]	Vasculature volume of Spleen
**Vv_Tmr**	~7% of total tumor volume	ml	[13] Calculated dynamically	Vasculature volume of Tumor based on fraction of total tumor volume

Table S5 – Model Parameters (End)

Table S6 – Model Algebraic Equations (Start)

Parameter and Species Assignment Equation	Assignment Type
[%{CD80}{PD1}C] = Frac_PD1_Exp_Cancer*Frac_CD80_Exp_Cancer*(1-Frac_PDL2_Exp_Cancer)*(1-Frac_PDL1_Exp_Cancer)	Repeated
[%{CD80}C] = Frac_CD80_Exp_Cancer*(1-Frac_PDL1_Exp_Cancer)*(1-Frac_PDL2_Exp_Cancer)*(1-Frac_PD1_Exp_Cancer)	Repeated
[%{Other}C] = (1-Frac_PDL1_Exp_Cancer)*(1-Frac_CD80_Exp_Cancer)*(1-Frac_PDL2_Exp_Cancer)*(1-Frac_PD1_Exp_Cancer)	Repeated
[%{PD1}{80}{L1}{L2}C] = Frac_PDL1_Exp_Cancer*Frac_CD80_Exp_Cancer*Frac_PDL2_Exp_Cancer*Frac_PD1_Exp_Cancer	Repeated
[%{PD1}{80}{L1}C] = Frac_PD1_Exp_Cancer*Frac_CD80_Exp_Cancer*Frac_PDL1_Exp_Cancer*(1-Frac_PDL2_Exp_Cancer)	Repeated
[%{PD1}{80}{PDL2}C] = Frac_PD1_Exp_Cancer*Frac_CD80_Exp_Cancer*Frac_PDL2_Exp_Cancer*(1-Frac_PDL1_Exp_Cancer)	Repeated
[%{PD1}{L1}{L2}C] = Frac_PD1_Exp_Cancer*Frac_PDL1_Exp_Cancer*Frac_PDL2_Exp_Cancer*(1-Frac_CD80_Exp_Cancer)	Repeated
[%{PD1}{L1}C] = Frac_PD1_Exp_Cancer*Frac_PDL1_Exp_Cancer*(1-Frac_PDL2_Exp_Cancer)*(1-Frac_CD80_Exp_Cancer)	Repeated
[%{PD1}{L2}C] = Frac_PD1_Exp_Cancer*Frac_PDL2_Exp_Cancer*(1-Frac_PDL1_Exp_Cancer)*(1-Frac_CD80_Exp_Cancer)	Repeated
[%{PD1}C] = Frac_PD1_Exp_Cancer*(1-Frac_PDL1_Exp_Cancer)*(1-Frac_PDL2_Exp_Cancer)*(1-Frac_CD80_Exp_Cancer)	Repeated
[%{PDL1}{80}{L2}C] = Frac_PDL1_Exp_Cancer*Frac_CD80_Exp_Cancer*Frac_PDL2_Exp_Cancer*(1-Frac_PD1_Exp_Cancer)	Repeated
[%{PDL1}{80}C] = Frac_CD80_Exp_Cancer*Frac_PDL1_Exp_Cancer*(1-Frac_PDL2_Exp_Cancer)*(1-Frac_PD1_Exp_Cancer)	Repeated
[%{PDL1}{PDL2}C] = Frac_PDL1_Exp_Cancer*Frac_PDL2_Exp_Cancer*(1-Frac_PD1_Exp_Cancer)*(1-Frac_CD80_Exp_Cancer)	Repeated
[%{PDL1}C] = Frac_PDL1_Exp_Cancer*(1-Frac_PD1_Exp_Cancer)*(1-Frac_PDL2_Exp_Cancer)*(1-Frac_CD80_Exp_Cancer)	Repeated
[%{PDL2}{80}C] = Frac_CD80_Exp_Cancer*Frac_PDL2_Exp_Cancer*(1-Frac_PDL1_Exp_Cancer)*(1-Frac_PD1_Exp_Cancer)	Repeated
[%{PDL2}C] = Frac_PDL2_Exp_Cancer*(1-Frac_PDL1_Exp_Cancer)*(1-Frac_PD1_Exp_Cancer)*(1-Frac_CD80_Exp_Cancer)	Repeated
[Blood-Lymph].CTLA4_mabB_ugml = ([Blood-Lymph].CTLA4_mabB*Ipilimumab_MW)/(Vc_CTLA4)	Repeated
[Blood-Lymph].Effector_T_Blood_Con = Effector_TB/(Vc_Teff * 5/2.7)	Repeated
[Blood-Lymph].Effector_T_TB = Effector_T * [Num_TDLN_Considered]	Repeated
[Blood-Lymph].Effector_T_TOTAL = EffT_P_f_Vasc+Effector_TT_Count+Effector_TB	Repeated
[Blood-Lymph].PD1_mabB_ugml = ([Blood-Lymph].PD1_mabb*Nivolumab_MW)/Vc_PD1	Repeated
[Blood-Lymph].PDL1_mabB_ugml = ([Blood-Lymph].PDL1_mabb*Durvalumab_MW)/Vc_PDL1	Repeated
[CD28_POS-Sig_NT] = (POS_Sig_NT_CD80+POS_Sig_NT_CD86)/(POS_Sig_NT_CD80+POS_Sig_NT_CD86+NT_Int_CD28+1E-100*mole)	Repeated
[CD28_POS-to-Total] = (POS_Sig_PNT_CD80+POS_Sig_PNT_CD86)/(POS_Sig_PNT_CD80+POS_Sig_PNT_CD86+PNT_Int_CD28+1E-100*mole)	Repeated
[CD80_PNT_NEG-to-Max] = [PNT_CD80-PDL1]/([PNT_Int_CD80] + [PNT_CD80-PDL1]+1E-100*mole)	Repeated
[CD80_receptors-per-mAPC] = 130000*mAPC_activation_level	Repeated
[CD80Sig_Tr-PNT] = [TrPNT_PDL1-CD80]/([TrPNT_PDL1-CD80] + [PNT-Tr_CD80] +1E-100*mole)	Repeated
[CD86_receptors-per-mAPC] = 208000*mAPC_activation_level	Repeated
[CTLA4_NEG-to-Total] = (NEG_Sig_PNT_CD80+NEG_Sig_PNT_CD86)/(NEG_Sig_PNT_CD80+NEG_Sig_PNT_CD86+PNT_CTLA4+CTLA4_mAb_CTLA4+1E-100*mole)	Repeated
[CTLA4_receptors-Int-PNT] = [CD28_receptors-per-Tcell]*[CTLA4-to-CD28_Ratio_Int]	Repeated
[CTLA4_receptors-nInt-PNT] = [CD28_receptors-per-Tcell]*[CTLA4-to-CD28_Ratio_nInt]	Repeated
[CTLA4_receptors-Tr] = [CD28_receptors-per-Tcell]*[CTLA4-to-CD28_Ratio_Tr]	Repeated
[CTLA4Sig_TrLN-mAPC] = ([TrALN_CT_aCT])/([TrALN_CT_CD80] + [TrALN_CT_CD86] + [Tr-mAPC_CTLA4] + [TrALN_CT_aCT]+1E-100*mole)	Repeated
[CTLA4-to-CD28_Ratio_Int] = 0.035*(1+Antigen_Intensity*2)	Repeated
[CTLA4-to-CD28_Ratio_nInt] = 0.035	Repeated
[Max_#Cells_per_mm^3] = 1/[Cancer_Cell_Vol_mm3]	Initial
[PD1_PNT_NEG-to-Max] = ([PNT_PD1-PDL2]+[PNT_PD1-PDL1])/([PNT_Int_PD1] + [PNT_PD1-PDL2]+[PNT_PD1-PDL1] + [PD1mAb_PNT_PD1]+1E-100*mole)	Repeated
[PD1Sig_Tr-PNT] = ([TrPNT_PDL1-PD1])/([TrPNT_PDL1-PD1] + [PNT-Tr_PD1] + [PNTTr_PD1_aPD1] +1E-100*mole)	Repeated
[PDL1_PNT_NEG-to-Max] = ([PNT_PDL1-CD80]+[PNT_PDL1-PD1])/([PNT_Int_PDL1] +[PNT_PDL1-CD80]+[PNT_PDL1-PD1] + [PDL1mAb-PNT_PDL1]+1E-100*mole)	Repeated
[PDL1Sig_Tr-PNT] = ([TrPNT_PD1-L1])/([TrPNT_PD1-L1] +[PNT-Tr_PDL1]+[PNTTr_PDL1_aPDL1] + 1E-100*mole)	Repeated

Parameter and Species Assignment Equation	Assignment Type
[Prob_NT-mAPC_Interact] = (1-exp((-4*pi*[T-mAPC_Cell_Size]*T_Cell_Diffusion*mAPC_Total_Calc_Pr*Precursor_Frequen_NT*T_Density*[T-mAPC_Inter_tmax_min]*Antigen_Intensity)/(T_Cell_Clonality*[Success_NT-mAPC])))	Repeated
[Prob_PNT-mAPC_Interact] = (1-exp((-4*pi*[T-mAPC_Cell_Size]*T_Cell_Diffusion*mAPC_Total_Calc_Pr*Precursor_Frequen_PNT*T_Density*[T-mAPC_Inter_tmax_min]*Antigen_Intensity)/(T_Cell_Clonality*[Success_PNT-mAPC])))	Repeated
[Sig_MDSCTeff=CD80] = ([PDL1:CD80_MDSCT])/([PDL1:CD80_MDSCT] +[CD80_TeffT1] + 1E-100*mole)	Repeated
[Sig_MDSCTeff=PD1] = ([PDL1:PD1_MDSCT])/([PDL1:PD1_MDSCT] + [PD1_TeffT1] + [PD1:aPD1_Teff1] + 1E-100*mole)	Repeated
[Sig_MDSCTeff=PDL1] = ([PD1:PDL1_MDSCT])/([PD1:PDL1_MDSCT] + [PDL1_TeffT1] + [PDL1:aPDL1_Teff1] + 1E-100*mole)	Repeated
[Sig_MDSCTeff=Total] = (([Sigweight_MDSCTeff=CD80]*([Sig_MDSCTeff=CD80]/[Sigmax_MDSCTeff=CD80])+[Sigweight_MDSCTeff=PD1]*([Sig_MDSCTeff=PD1]/[Sigmax_MDSCTeff=PD1])+[Sigweight_MDSCTeff=PDL1]*([Sig_MDSCTeff=PDL1]/[Sigmax_MDSCTeff=PDL1]))^gamma_Sig1 / (1 + ([Sigweight_MDSCTeff=CD80]*([Sig_MDSCTeff=CD80]/[Sigmax_MDSCTeff=CD80])+[Sigweight_MDSCTeff=PD1]*([Sig_MDSCTeff=PD1]/[Sigmax_MDSCTeff=PD1])+[Sigweight_MDSCTeff=PDL1]*([Sig_MDSCTeff=PDL1]/[Sigmax_MDSCTeff=PDL1]))^gamma_Sig1))*(1-[Sig_MDSCTeff = Other])+[Sig_MDSCTeff = Other]	Repeated
[Sig_NT=CD28] = ([CD28_POS-Sig_NT]/[Sigmax_NT=CD28])	Repeated
[Sig_PNT=CTLA4/PD1/L1/CD80] = ([SigWeight_PNT=CTLA4]*[CTLA4_NEG-to-Total]/[Sigmax_PNT=CTLA4]+[SigWeight_PNT=PD1]*[PD1_PNT_NEG-to-Max]/[Sigmax_PNT=PD1]+[SigWeight_PNT=PDL1]*[PDL1_PNT_NEG-to-Max]/[Sigmax_PNT=PDL1]+[SigWeight_PNT=CD80]*[CD80_PNT_NEG-to-Max]/[Sigmax_PNT=CD80])^gamma_Sig/(1+([SigWeight_PNT=CTLA4]*[CTLA4_NEG-to-Total]/[Sigmax_PNT=CTLA4]+[SigWeight_PNT=PD1]*[PD1_PNT_NEG-to-Max]/[Sigmax_PNT=PD1]+[SigWeight_PNT=PDL1]*[PDL1_PNT_NEG-to-Max]/[Sigmax_PNT=PDL1]+[SigWeight_PNT=CD80]*[CD80_PNT_NEG-to-Max]/[Sigmax_PNT=CD80])^gamma_Sig)	Repeated
[Sig_T1=Total] = 1	Repeated
[Sig_T10=Total] = (([Sigweight_T10a=PDL1]*[Sig_T10a=PDL1]/[Sigmax_T10a=PDL1] + [Sigweight_T10b=PD1]*[Sig_T10b=PD1]/[Sigmax_T10b=PD1] + [Sigweight_T10c=CD80]*[Sig_T10c=CD80]/[Sigmax_T10c=CD80]) ^gamma_Sig1/(1 + ([Sigweight_T10a=PDL1]*[Sig_T10a=PDL1]/[Sigmax_T10a=PDL1] + [Sigweight_T10b=PD1]*[Sig_T10b=PD1]/[Sigmax_T10b=PD1] + [Sigweight_T10c=CD80]*[Sig_T10c=CD80]/[Sigmax_T10c=CD80])^gamma_Sig1))*(1-[Sig_T=Other])+[Sig_T=Other]	Repeated
[Sig_T10a=PDL1] = ([T10a=PDL1:PD1=C10a])/([T10a=PDL1:PD1=C10a] +[T10a=PDL1]+[T10a=PDL1:aPDL1] +1E-100*mole)	Repeated
[Sig_T10b=PD1] = ([T10b=PD1:PDL1=C10b] + [T10b=PD1:PDL2=C10c])/([T10b=PD1:PDL1=C10b] + [T10b=PD1:PDL2=C10c] +[T10b=PD1]+[T10b=PD1:aPD1] +1E-100*mole)	Repeated
[Sig_T10c=CD80] = ([T10c=CD80:PDL1=C10b])/([T10c=CD80:PDL1=C10b] +[T10c=CD80] +1E-100*mole)	Repeated
[Sig_T11=Total] = (([Sigweight_T11a=PDL1]*[Sig_T11a=PDL1]/[Sigmax_T11a=PDL1] + [Sigweight_T11b=PD1]*[Sig_T11b=PD1]/[Sigmax_T11b=PD1]) ^gamma_Sig1/(1 + ([Sigweight_T11a=PDL1]*[Sig_T11a=PDL1]/[Sigmax_T11a=PDL1] + [Sigweight_T11b=PD1]*[Sig_T11b=PD1]/[Sigmax_T11b=PD1])^gamma_Sig1))*(1-[Sig_T=Other])+[Sig_T=Other]	Repeated
[Sig_T11a=PDL1] = ([T11a=PDL1:CD80=C11b])/([T11a=PDL1:CD80=C11b] +[T11a=PDL1]+[T11a=PDL1:aPDL1] +1E-100*mole)	Repeated
[Sig_T11b=PD1] = ([T11b=PD1:PDL1=C11a])/([T11b=PD1:PDL1=C11a] +[T11b=PD1]+[T11b=PD1:aPD1] +1E-100*mole)	Repeated
[Sig_T12=Total] = (([Sigweight_T12a=PDL1]*[Sig_T12a=PDL1]/[Sigmax_T12a=PDL1] + [Sigweight_T12b=PD1]*[Sig_T12b=PD1]/[Sigmax_T12b=PD1]) ^gamma_Sig1/(1 + ([Sigweight_T12a=PDL1]*[Sig_T12a=PDL1]/[Sigmax_T12a=PDL1] + [Sigweight_T12b=PD1]*[Sig_T12b=PD1]/[Sigmax_T12b=PD1])^gamma_Sig1))*(1-[Sig_T=Other])+[Sig_T=Other]	Repeated
[Sig_T12a=PDL1] = ([T12a=PDL1:CD80=C12b])/([T12a=PDL1:CD80=C12b] +[T12a=PDL1]+[T12a=PDL1:aPDL1] +1E-100*mole)	Repeated
[Sig_T12b=PD1] = ([T12b=PD1:PDL2=C12a])/([T12b=PD1:PDL2=C12a] +[T12b=PD1]+[T12b=PD1:aPD1] +1E-100*mole)	Repeated
[Sig_T13=Total] = (([Sigweight_T13a=PDL1]* [Sig_T13a=PDL1]/[Sigmax_T13a=PDL1] + [Sigweight_T13b=PD1]*[Sig_T13b=PD1]/[Sigmax_T13b=PD1]) ^gamma_Sig1/(1 + ([Sigweight_T13a=PDL1]* [Sig_T13a=PDL1]/[Sigmax_T13a=PDL1] + [Sigweight_T13b=PD1]*[Sig_T13b=PD1]/[Sigmax_T13b=PD1])^gamma_Sig1))*(1-[Sig_T=Other])+[Sig_T=Other]	Repeated
[Sig_T13a=PDL1] = ([T13a=PDL1:CD80=C13c])/([T13a=PDL1:CD80=C13c] +[T13a=PDL1]+[T13a=PDL1:aPDL1] +1E-100*mole)	Repeated
[Sig_T13b=PD1] = ([T13b=PD1:PDL2=C13b] + [T13b=PD1:PDL1=C13a])/([T13b=PD1:PDL2=C13b] + [T13b=PD1:PDL1=C13a] +[T13b=PD1]+[T13b=PD1:aPD1] +1E-100*mole)	Repeated
[Sig_T14=Total] = (([Sigweight_T14a=PDL1]*[Sig_T14a=PDL1]/[Sigmax_T14a=PDL1] + [Sigweight_T14b=PD1]*[Sig_T14b=PD1]/[Sigmax_T14b=PD1] + [Sigweight_T14c=CD80]*[Sig_T14c=CD80]/[Sigmax_T14c=CD80]) ^gamma_Sig1/(1 +	Repeated

Parameter and Species Assignment Equation	Assignment Type
([Sigweight_T14a=PDL1]*[Sig_T14a=PDL1]/[Sigmax_T14a=PDL1] + [Sigweight_T14b=PD1]*[Sig_T14b=PD1]/[Sigmax_T14b=PD1] + [Sigweight_T14c=CD80]*[Sig_T14c=CD80]/[Sigmax_T14c=CD80])^gamma_Sig1))*(1-[Sig_T=Other])+[Sig_T=Other]	
[Sig_T14a=PDL1] = ([T14a=PDL1:PD1=C14a] + [T14a=PDL1:CD80=C14c])/([T14a=PDL1:PD1=C14a] + [T14a=PDL1:CD80=C14c] +[T14a=PDL1]+[T14a=PDL1:aPDL1] +1E-100*mole)	Repeated
[Sig_T14b=PD1] = ([T14b=PD1:PDL1=C14b])/([T14b=PD1:PDL1=C14b] +[T14b=PD1]+[T14b=PD1:aPD1] +1E-100*mole)	Repeated
[Sig_T14c=CD80] = ([T14c=CD80:PDL1=C14b])/([T14c=CD80:PDL1=C14b] +[T14c=CD80] +1E-100*mole)	Repeated
[Sig_T15=Total] = (([Sigweight_T15a=PDL1]*[Sig_T15a=PDL1]/[Sigmax_T15a=PDL1] + [Sigweight_T15b=PD1]*[Sig_T15b=PD1]/[Sigmax_T15b=PD1]) ^gamma_Sig1/(1 + ([Sigweight_T15a=PDL1]*[Sig_T15a=PDL1]/[Sigmax_T15a=PDL1] + [Sigweight_T15b=PD1]*[Sig_T15b=PD1]/[Sigmax_T15b=PD1])^gamma_Sig1))*(1-[Sig_T=Other])+[Sig_T=Other]	Repeated
[Sig_T15a=PDL1] = ([T15a=PDL1:CD80=C15c] + [T15a=PDL1:PD1=C15a])/([T15a=PDL1:CD80=C15c]+[T15a=PDL1:PD1=C15a] +[T15a=PDL1]+[T15a=PDL1:aPDL1] +1E-100*mole)	Repeated
[Sig_T15b=PD1] = ([T15b=PD1:PDL2=C15b])/([T15b=PD1:PDL2=C15b] +[T15b=PD1]+[T15b=PD1:aPD1] +1E-100*mole)	Repeated
[Sig_T16=Total] = (([Sigweight_T16a=PDL1]*[Sig_T16a=PDL1]/[Sigmax_T16a=PDL1] + [Sigweight_T16b=PD1]*[Sig_T16b=PD1]/[Sigmax_T16b=PD1] + [Sigweight_T16c=CD80]*[Sig_T16c=CD80]/[Sigmax_T16c=CD80]) ^gamma_Sig1/(1 + ([Sigweight_T16a=PDL1]*[Sig_T16a=PDL1]/[Sigmax_T16a=PDL1] + [Sigweight_T16b=PD1]*[Sig_T16b=PD1]/[Sigmax_T16b=PD1] + [Sigweight_T16c=CD80]*[Sig_T16c=CD80]/[Sigmax_T16c=CD80])^gamma_Sig1))*(1-[Sig_T=Other])+[Sig_T=Other]	Repeated
[Sig_T16a=PDL1] = ([T16a=PDL1:PD1=C16a] + [T16a=PDL1:CD80=C16d])/([T16a=PDL1:PD1=C16a]+[T16a=PDL1:CD80=C16d] +[T16a=PDL1]+[T16a=PDL1:aPDL1] +1E-100*mole)	Repeated
[Sig_T16b=PD1] = ([T16b=PD1:PDL2=C16c]+[T16b=PD1:PDL1=C16b])/([T16b=PD1:PDL2=C16c]+[T16b=PD1:PDL1=C16b] +[T16b=PD1]+[T16b=PD1:aPD1] +1E-100*mole)	Repeated
[Sig_T16c=CD80] = ([T16c=CD80:PDL1=C16b])/([T16c=CD80:PDL1=C16b] +[T16c=CD80]+1E-100*mole)	Repeated
[Sig_T2=PDL1] = ([T2=PDL1:PD1=C2])/([T2=PDL1:PD1=C2] +[T2=PDL1]+[T2=PDL1:aPDL1] +1E-100*mole)	Repeated
[Sig_T2=Total] = (([Sigweight_T2=PDL1]*[Sig_T2=PDL1]/[Sigmax_T2=PDL1]) ^gamma_Sig1/(1 + ([Sigweight_T2=PDL1]*[Sig_T2=PDL1]/[Sigmax_T2=PDL1])^gamma_Sig1))*(1-[Sig_T=Other])+[Sig_T=Other]	Repeated
[Sig_T3=Total] = (([Sigweight_T3a=PD1]*[Sig_T3a=PD1]/[Sigmax_T3a=PD1] + [Sigweight_T3b=CD80]*[Sig_T3b=CD80]/[Sigmax_T3b=CD80]) ^gamma_Sig1/(1 + ([Sigweight_T3a=PD1]*[Sig_T3a=PD1]/[Sigmax_T3a=PD1] + [Sigweight_T3b=CD80]*[Sig_T3b=CD80]/[Sigmax_T3b=CD80])^gamma_Sig1))*(1-[Sig_T=Other])+[Sig_T=Other]	Repeated
[Sig_T3a=PD1] = ([T3a=PD1:PDL1=C3])/([T3a=PD1:PDL1=C3] +[T3a=PD1]+[T3a=PD1:aPD1] +1E-100*mole)	Repeated
[Sig_T3b=CD80] = ([T3b=CD80:PDL1=C3])/([T3b=CD80:PDL1=C3] +[T3b=CD80] +1E-100*mole)	Repeated
[Sig_T4=PD1] = ([T4=PD1:PDL2=C4])/([T4=PD1:PDL2=C4] +[T4=PD1]+[T4=PD1:aPD1] +1E-100*mole)	Repeated
[Sig_T4=Total] = (([Sigweight_T4=PD1]*[Sig_T4=PD1]/[Sigmax_T4=PD1])^ gamma_Sig1/(1 + ([Sigweight_T4=PD1]*[Sig_T4=PD1]/[Sigmax_T4=PD1])^gamma_Sig1))*(1-[Sig_T=Other])+[Sig_T=Other]	Repeated
[Sig_T5=PDL1] = ([T5=PDL1:CD80=C5])/([T5=PDL1:CD80=C5] +[T5=PDL1]+[T5=PDL1:aPDL1] +1E-100*mole)	Repeated
[Sig_T5=Total] = (([Sigweight_T5=PDL1]*[Sig_T5=PDL1]/[Sigmax_T5=PDL1])^ gamma_Sig1/(1 + ([Sigweight_T5=PDL1]*[Sig_T5=PDL1]/[Sigmax_T5=PDL1])^gamma_Sig1))*(1-[Sig_T=Other])+[Sig_T=Other]	Repeated
[Sig_T6=Total] = (([Sigweight_T6a=PDL1]*[Sig_T6a=PDL1]/[Sigmax_T6a=PDL1] + [Sigweight_T6b=PD1]*[Sig_T6b=PD1]/[Sigmax_T6b=PD1] + [Sigweight_T6c=CD80]*[Sig_T6c=CD80]/[Sigmax_T6c=CD80])^ gamma_Sig1/(1 + ([Sigweight_T6a=PDL1]*[Sig_T6a=PDL1]/[Sigmax_T6a=PDL1] + [Sigweight_T6b=PD1]*[Sig_T6b=PD1]/[Sigmax_T6b=PD1] + [Sigweight_T6c=CD80]*[Sig_T6c=CD80]/[Sigmax_T6c=CD80])^gamma_Sig1))*(1-[Sig_T=Other])+[Sig_T=Other]	Repeated
[Sig_T6a=PDL1] = ([T6a=PDL1:PD1=C6a])/([T6a=PDL1:PD1=C6a] +[T6a=PDL1]+[T6a=PDL1:aPDL1] +1E-100*mole)	Repeated
[Sig_T6b=PD1] = ([T6b=PD1:PDL1=C6b])/([T6b=PD1:PDL1=C6b] +[T6b=PD1]+[T6b=PD1:aPD1] +1E-100*mole)	Repeated
[Sig_T6c=CD80] = ([T6c=CD80:PDL1=C6b])/([T6c=CD80:PDL1=C6b] +[T6c=CD80] +1E-100*mole)	Repeated
[Sig_T7=Total] = (([Sigweight_T7a=PDL1]*[Sig_T7a=PDL1]/[Sigmax_T7a=PDL1] + [Sigweight_T7b=PD1]*[Sig_T7b=PD1]/[Sigmax_T7b=PD1]) ^gamma_Sig1/(1 + ([Sigweight_T7a=PDL1]*[Sig_T7a=PDL1]/[Sigmax_T7a=PDL1] + [Sigweight_T7b=PD1]*[Sig_T7b=PD1]/[Sigmax_T7b=PD1])^gamma_Sig1))*(1-[Sig_T=Other])+[Sig_T=Other]	Repeated
[Sig_T7a=PDL1] = ([T7a=PDL1:PD1=C7a])/([T7a=PDL1:PD1=C7a] +[T7a=PDL1]+[T7a=PDL1:aPDL1] +1E-100*mole)	Repeated
[Sig_T7b=PD1] = ([T7b=PD1:PDL2=C7b])/([T7b=PD1:PDL2=C7b] +[T7b=PD1]+[T7b=PD1:aPD1] +1E-100*mole)	Repeated
[Sig_T8=Total] = (([Sigweight_T8a=PD1]*[Sig_T8a=PD1]/[Sigmax_T8a=PD1] + [Sigweight_T8b=CD80]*[Sig_T8b=CD80]/[Sigmax_T8b=CD80]) ^gamma_Sig1/(1 + ([Sigweight_T8a=PD1]*[Sig_T8a=PD1]/[Sigmax_T8a=PD1] + [Sigweight_T8b=CD80]*[Sig_T8b=CD80]/[Sigmax_T8b=CD80])^gamma_Sig1))*(1-[Sig_T=Other])+[Sig_T=Other]	Repeated
[Sig_T8a=PD1] = ([T8a=PD1:PDL1=C8a] + [T8a=PD1:PDL2=C8b])/([T8a=PD1:PDL1=C8a] +[T8a=PD1:PDL2=C8b] + [T8a=PD1]+[T8a=PD1:aPD1] +1E-100*mole)	Repeated

Parameter and Species Assignment Equation	Assignment Type
[Sig_T8b=CD80] = ([T8b=CD80:PDL1=C8a])/([T8b=CD80:PDL1=C8a] +[T8b=CD80] +1E-100*mole)	Repeated
[Sig_T9=PDL1] = ([T9=PDL1-PD1=C9a] + [T9=PDL1-CD80=C9b])/([T9=PDL1-PD1=C9a] + [T9=PDL1-CD80=C9b] +[T9=PDL1]+[T9=PDL1:aPDL1] +1E-100*mole)	Repeated
[Sig_T9=Total] = (([Sigweight_T9=PDL1]*[Sig_T9=PDL1]/[Sigmax_T9=PDL1]) ^gamma_Sig1/(1 + ([Sigweight_T9=PDL1]*[Sig_T9=PDL1]/[Sigmax_T9=PDL1])^gamma_Sig1))*(1-[Sig_T=Other])+[Sig_T=Other]	Repeated
[Sig_TrAPCT=CTLA4] = ([CTLA4:CD80_TrAT] + [CTLA4:CD86_TrAT])/ ([CTLA4:CD80_TrAT] + [CTLA4:CD86_TrAT] + [CD80_mAPCT] + [CD86_mAPCT] + [CTLA4_CTLA4-Trt] + 1E-100*mole)	Repeated
[Sig_TrAPCT=Total] = (([Sig_TrAPCT=CTLA4]/[Sigmax_TrAPCT=CTLA4])^gamma_Sig / (1 + ([Sig_TrAPCT=CTLA4]/[Sigmax_TrAPCT=CTLA4])^gamma_Sig))*(1-[Sig_TrAPC = Other])+[Sig_TrAPC = Other]	Repeated
[Sig_TrPNT=PD1/L1/CD80] = (([SigWeight_TrPNT=CD80]*[CD80Sig_Tr-PNT]/[Sigmax_TrPNT=CD80] + [SigWeight_TrPNT=PD1]*[PD1Sig_Tr-PNT]/[Sigmax_TrPNT=PD1] + [SigWeight_TrPNT=PDL1]*[PDL1Sig_Tr-PNT]/[Sigmax_TrPNT=PDL1])^gamma_SigTr / (1 + ([SigWeight_TrPNT=CD80]*[CD80Sig_Tr-PNT]/[Sigmax_TrPNT=CD80] + [SigWeight_TrPNT=PD1]*[PD1Sig_Tr-PNT]/[Sigmax_TrPNT=PD1] + [SigWeight_TrPNT=PDL1]*[PDL1Sig_Tr-PNT]/[Sigmax_TrPNT=PDL1]))^gamma_SigTr)*(1-[SigWeight_TrPNT=Other]*([Sigmax_TrPNT=Other]))+ [SigWeight_TrPNT=Other]*([Sigmax_TrPNT=Other])	Repeated
[Sig_TrTeff=CD80] = ([PDL1:CD80_TrTeff])/([PDL1:CD80_TrTeff] + [CD80_TeffT] + 1E-100*mole)	Repeated
[Sig_TrTeff=PD1] = ([PDL1:PD1_TrTeff])/([PDL1:PD1_TrTeff] + [PD1_TeffT] + [PD1:aPD1_Teff] + 1E-100*mole)	Repeated
[Sig_TrTeff=PDL1] = ([PD1:PDL1_TrTeff])/([PD1:PDL1_TrTeff] + [PDL1_TeffT] + [PDL1:aPDL1_Teff] + 1E-100*mole)	Repeated
[Sig_TrTeff=Total] = (([Sigweight_TrTeff=PD1]*[Sig_TrTeff=PD1]/[Sigmax_TrTeff=PD1] +[Sigweight_TrTeff=PDL1]*[Sig_TrTeff=PDL1]/[Sigmax_TrTeff=PDL1] +[Sigweight_TrTeff=CD80]*[Sig_TrTeff=CD80]/[Sigmax_TrTeff=CD80])^gamma_Sig1/(1+([Sigweight_TrTeff=PD1]*[Sig_TrTeff=PD1]/[Sigmax_TrTeff=PD1] +[Sigweight_TrTeff=PDL1]*[Sig_TrTeff=PDL1]/[Sigmax_TrTeff=PDL1] +[Sigweight_TrTeff=CD80]*[Sig_TrTeff=CD80]/[Sigmax_TrTeff=CD80])^gamma_Sig1)) *(1-[Sig_TrTeff = Other])+[Sig_TrTeff = Other]	Repeated
[Success_NT-mAPC] = 500	Repeated
[Success_PNT-mAPC] = 50	Repeated
[V_T:C_10] = ((4*pi*(([T-mAPC_Cell_Size]/2)^2)*([T{PD1}{L1}{80}-{PD1}{L1}{L2}C])*(Cancer_per_T_Cell_Int/Cancer_per_T_Cell_max)*15E-3)*(L/mole))*(1E-15)*(IS_Scaling)	Repeated
[V_T:C_11] = ((4*pi*(([T-mAPC_Cell_Size]/2)^2)*([T{PD1}{L1}-{PDL1}{80}C])*(Cancer_per_T_Cell_Int/Cancer_per_T_Cell_max)*15E-3)*(L/mole))*(1E-15)*(IS_Scaling)	Repeated
[V_T:C_12] = ((4*pi*(([T-mAPC_Cell_Size]/2)^2)*([T{PD1}{L1}-{PDL2}{80}C])*(Cancer_per_T_Cell_Int/Cancer_per_T_Cell_max)*15E-3)*(L/mole))*(1E-15)*(IS_Scaling)	Repeated
[V_T:C_13] = ((4*pi*(([T-mAPC_Cell_Size]/2)^2)*([T{PD1}{L1}-{PDL1}{80}{L2}C])*(Cancer_per_T_Cell_Int/Cancer_per_T_Cell_max)*15E-3)*(L/mole))*(1E-15)*(IS_Scaling)	Repeated
[V_T:C_14] = ((4*pi*(([T-mAPC_Cell_Size]/2)^2)*([T{PD1}{L1}{80}-{PD1}{80}{L1}C])*(Cancer_per_T_Cell_Int/Cancer_per_T_Cell_max)*15E-3)*(L/mole))*(1E-15)*(IS_Scaling)	Repeated
[V_T:C_15] = ((4*pi*(([T-mAPC_Cell_Size]/2)^2)*([T{PD1}{L1}-{PD1}{80}{PDL2}C])*(Cancer_per_T_Cell_Int/Cancer_per_T_Cell_max)*15E-3)*(L/mole))*(1E-15)*(IS_Scaling)	Repeated
[V_T:C_16] = ((4*pi*(([T-mAPC_Cell_Size]/2)^2)*([T{PD1}{L1}{80}-{PD1}{80}{L1}{L2}C])*(Cancer_per_T_Cell_Int/Cancer_per_T_Cell_max)*15E-3)*(L/mole))*(1E-15)*(IS_Scaling)	Repeated
[V_T:C_2] = ((4*pi*(([T-mAPC_Cell_Size]/2)^2)*([T{PDL1}-{PD1}C])*(Cancer_per_T_Cell_Int/Cancer_per_T_Cell_max)*15E-3)*(L/mole))*(1E-15)*(IS_Scaling)	Repeated
[V_T:C_3] = ((4*pi*(([T-mAPC_Cell_Size]/2)^2)*([T{PD1}{CD80}-{PDL1}C])*(Cancer_per_T_Cell_Int/Cancer_per_T_Cell_max)*15E-3)*(L/mole))*(1E-15)*(IS_Scaling)	Repeated
[V_T:C_4] = ((4*pi*(([T-mAPC_Cell_Size]/2)^2)*([T{PD1}-{PDL2}C])*(Cancer_per_T_Cell_Int/Cancer_per_T_Cell_max)*15E-3)*(L/mole))*(1E-15)*(IS_Scaling)	Repeated
[V_T:C_5] = ((4*pi*(([T-mAPC_Cell_Size]/2)^2)*([T{PDL1}-{CD80}C])*(Cancer_per_T_Cell_Int/Cancer_per_T_Cell_max)*15E-3)*(L/mole))*(1E-15)*(IS_Scaling)	Repeated
[V_T:C_6] = ((4*pi*(([T-mAPC_Cell_Size]/2)^2)*([T{PD1}{L1}{80}-{PD1}{L1}C])*(Cancer_per_T_Cell_Int/Cancer_per_T_Cell_max)*15E-3)*(L/mole))*(1E-15)*(IS_Scaling)	Repeated
[V_T:C_7] = ((4*pi*(([T-mAPC_Cell_Size]/2)^2)*([T{PD1}{L1}-{PD1}{L2}C])*(Cancer_per_T_Cell_Int/Cancer_per_T_Cell_max)*15E-3)*(L/mole))*(1E-15)*(IS_Scaling)	Repeated
[V_T:C_8] = ((4*pi*(([T-mAPC_Cell_Size]/2)^2)*([T{PD1}{80}-{PDL1}{PDL2}C])*(Cancer_per_T_Cell_Int/Cancer_per_T_Cell_max)*15E-3)*(L/mole))*(1E-15)*(IS_Scaling)	Repeated
[V_T:C_9] = ((4*pi*(([T-mAPC_Cell_Size]/2)^2)*([T{PDL1}-{CD80}{PD1}C])*(Cancer_per_T_Cell_Int/Cancer_per_T_Cell_max)*15E-3)*(L/mole))*(1E-15)*(IS_Scaling)	Repeated
[Vol_Cell-Rec_MDSC-TeffT] = ((4*pi*(([TrLN-PNT_Cell_Size]/2)^2)*(MDSCsT_EngTeff/T_per_Tr_cell)*15E-3))*(1E-15)*(IS_Scaling)*(L/mole)	Repeated
[Vol_Cell-Rec_Tr-APCT] = ((4*pi*(([TrLN-mAPC_Cell_Size]/2)^2)*(TregT_EngAPC/mAPC_per_Tr_cell)*15E-3))*(1E-15)*(IS_Scaling)*(L/mole)	Repeated
[Vol_Cell-Rec_Tr-mAPC] = ((4*pi*(([TrLN-mAPC_Cell_Size]/2)^2)*(TregLN_mAPC1/mAPC_per_Tr_cell)*15E-3))*(1E-15)*(IS_Scaling)*(L/mole)	Repeated
[Vol_Cell-Rec_Tr-PNT] = ((4*pi*(([TrLN-PNT_Cell_Size]/2)^2)*([TregLN-PNT1]/T_per_Tr_cell)*15E-3))*(1E-15)*(IS_Scaling)*(L/mole)	Repeated
[Vol_Cell-Rec_Tr-TeffT] = ((4*pi*(([TrLN-PNT_Cell_Size]/2)^2)*(TregT_EngTeff/T_per_Tr_cell)*15E-3))*(1E-15)*(IS_Scaling)*(L/mole)	Repeated

Parameter and Species Assignment Equation	Assignment Type
[Volume_NT-Receptor_Int] = ((4*pi*(([T-mAPC_Cell_Size]/2)^2)*((mAPC_Int_P1)/mAPC_per_T_cell)*15E-3)*(L/mole))*(1E-15)*(IS_Scaling)	Repeated
[Volume_PNT-Receptor_Int] = ((4*pi*(([T-mAPC_Cell_Size]/2)^2)*((mAPC_Int_P2)/mAPC_per_T_cell)*15E-3)*(L/mole))*(1E-15)*(IS_Scaling)	Repeated
Cancer_Cell_Vol_mm3 = (4/3)*pi*(([Cancer_Cell_Diam_um]/2000)^3)	Initial
Cancer_Diam_mm = ((6*Cancer)/(pi*Tumor_Vol_Ellipse_c_ratio*(1/((4/3)*pi*((Cancer_Cell_Diam_um/2000)^3)))*(1-Total_Tumor_Void_Frac)*mole))^(1/3)	Repeated
Cancer_Vol_cm3 = (4/3)*pi*(([Cancer_Diam_mm]/2)^2)*(([Cancer_Diam_mm]*[Tumor_Vol_Ellipse_c_ratio])/2000)	Repeated
CTLA4Sig_TrLN = [TrLN_CT_aCT]/([TrLN_CT_aCT] + [TrLN_CTLA4] +1E-100*mole)	Repeated
Frac_CD80_Exp_Cancer = [%CD80_Exp_Cancer]/100	Repeated
Frac_PD1_Exp_Cancer = [%PD1_Exp_Cancer]/100	Repeated
Frac_PDL1_Exp_Cancer = [%PDL1_Exp_Cancer]/100	Repeated
Frac_PDL2_Exp_Cancer = [%PDL2_Exp_Cancer]/100	Repeated
Liv_Spln_GI.Liver_Free_Sites = Adhesion_D_Lung_Liv_spln*Vv_Liver - EffT_Liver_b_Vasc-EffT_Liver_AR_Vasc	Repeated
Liv_Spln_GI.Spleen_Free_Sites = Adhesion_D_Lung_Liv_spln*Vv_Spleen-EffT_Spleen_b_Vasc-EffT_Spleen_AR_Vasc	Repeated
Lungs.Lung_Free_Sites = Adhesion_D_Lung_Liv_spln*Vv_Lungs - EffT_r_LungsVasc - EffT_AR_LungsVasc	Repeated
Lymph_Node.[Total_T_CD8-CD4] = Total_T_per_TDLN	Repeated
Lymph_Node.[TregLN-PNT1] = Lymph_Node.[TregLN-PNT]+0.001*mole	Repeated
Lymph_Node.CTLA4_mab_ugml = (Lymph_Node.CTLA4_mab*Ipilimumab_MW)/VL	Repeated
Lymph_Node.mAPC_cells_per_ml = mAPC/Vext_LN	Repeated
Lymph_Node.mAPC_Int_P1 = 0.001*mole+gt(Naive_T1,0)*gt(Primed_Naive_T1+Naive_T1,0)*(Naive_T1*[mAPC_per_T_cell] * gt(((Naive_T1 * mAPC)/(Primed_Naive_T1 + Naive_T1+1E-100*mole)) / (Naive_T1 *[mAPC_per_T_cell]+ 1E-100*mole),1) + (Naive_T1/(Primed_Naive_T1 + Naive_T1+1E-100*mole)) *mAPC* le(((Naive_T1 * mAPC)/(Primed_Naive_T1 + Naive_T1+1E-100*mole)) / (Naive_T1 *[mAPC_per_T_cell]+ 1E-100*mole),1))	Repeated
Lymph_Node.mAPC_Int_P2 = 0.001*mole+gt(Primed_Naive_T1,0)*gt(Primed_Naive_T1+Naive_T1,0)*(Primed_Naive_T1*[mAPC_per_T_cell] * gt(((Primed_Naive_T1 * mAPC)/(Primed_Naive_T1 + Naive_T1+1E-100*mole)) / (Primed_Naive_T1 *[mAPC_per_T_cell]+ 1E-100*mole),1) + (Primed_Naive_T1/(Primed_Naive_T1 + Naive_T1+1E-100*mole)) *mAPC* le(((Primed_Naive_T1 * mAPC)/(Primed_Naive_T1 + Naive_T1+1E-100*mole)) / (Primed_Naive_T1 *[mAPC_per_T_cell]+ 1E-100*mole),1))	Repeated
Lymph_Node.mAPC_nInt = (mAPC - mAPC_Int_P1 - mAPC_Int_P2) * ge(mAPC - mAPC_Int_P1 - mAPC_Int_P2,0)	Repeated
Lymph_Node.mAPC_Total_Calc_Pr = (mAPC-mAPC_Int_P1-mAPC_Int_P2)*ge(mAPC-mAPC_Int_P1-mAPC_Int_P2,0)	Repeated
Lymph_Node.mAPC1 = mAPC - [TregLN_mAPC]	Repeated
Lymph_Node.Naive_T = Lymph_Node.Naive_T0	Initial
Lymph_Node.Naive_T0 = T_Cell_Clonality * [Copies-per-T_Cell_Clone]	Repeated
Lymph_Node.Naive_T2 = Naive_T - [TregLN-NT]	Repeated
Lymph_Node.NT1_Int1 = Naive_T+0.001*mole	Repeated
Lymph_Node.PD1_mabLN_ugml = (Lymph_Node.PD1_mab*Nivolumab_MW)/VL	Repeated
Lymph_Node.PDL1_mabLN_ugml = (Lymph_Node.PDL1_mab*Durvalumab_MW)/VL	Repeated
Lymph_Node.PNT1_Int = Lymph_Node.Primed_Naive_T1+0.001*mole	Repeated
Lymph_Node.Primed_Naive_T2 = Primed_Naive_T - [TregLN-PNT]	Repeated
Lymph_Node.TregLN = ((Naive_T0)*([%_Tr_LN]/[%_NT_LN]) - TregLN_mAPC - [TregLN-NT] - [TregLN-PNT])*gt(mAPC,0)*(1-[CTLA4Sig_TrLN])	Repeated
Lymph_Node.TregLN_mAPC1 = Lymph_Node.TregLN_mAPC + 0.001*mole	Repeated
Peripheral.CTLA4_leaky_ugml = (Peripheral.CTLA4_mabP_leaky*Ipilimumab_MW)/(0.35*ISF)	Repeated
Peripheral.CTLA4_tight_ugml = (Peripheral.CTLA4_mabP_tight*Ipilimumab_MW)/(0.65*ISF)	Repeated
Peripheral.PD1_leaky_ugml = (Peripheral.PD1_mabP_leaky*Nivolumab_MW)/(0.35*ISF)	Repeated
Peripheral.PD1_tight_ugml = (Peripheral.PD1_mabP_tight*Nivolumab_MW)/(0.65*ISF)	Repeated
Peripheral.PDL1_leaky_ugml = (Peripheral.PDL1_mabP_leaky*Durvalumab_MW)/(0.35*ISF)	Repeated
Peripheral.PDL1_tight_ugml = (Peripheral.PDL1_mabP_tight*Durvalumab_MW)/(0.65*ISF)	Repeated
Precursor_Frequen_NT = Naive_T/[Total_T_CD8-CD4]	Repeated
Precursor_Frequen_PNT = Primed_Naive_T/[Total_T_CD8-CD4]	Repeated
Starting_Cancer_Diam_mm = Cancer_mm_Start_Therapy*0.95	Repeated
T_Density = [Total_T_CD8-CD4]/((4/3)*pi*TDLN_Radius^3)	Repeated
TDLN_Radius = 10000*([Vol_per_TDLN]*(3/(4*pi)))^(1/3)	Repeated
Total_TC_Sig = [Sig_T1=Total]*[%{Other}C] + [Sig_T2=Total]*[%{PD1}C] + [Sig_T3=Total]*[%{PDL1}C] + [Sig_T4=Total]*[%{PDL2}C] + [Sig_T5=Total]*[%{CD80}C] + [Sig_T6=Total]*[%{PD1}{L1}C] + [Sig_T7=Total]*[%{PD1}{L2}C] + [Sig_T8=Total]*[%{PDL1}{PDL2}C] + [Sig_T9=Total]*[%{CD80}{PD1}C] + [Sig_T10=Total]*[%{PD1}{L1}{L2}C] + [Sig_T11=Total]*[%{PDL1}{80}C] + [Sig_T12=Total]*[%{PDL2}{80}C] + [Sig_T13=Total]*[%{PDL1}{80}{L2}C] + [Sig_T14=Total]*[%{PD1}{80}{L1}C] + [Sig_T15=Total]*[%{PD1}{80}{PDL2}C] + [Sig_T16=Total]*[%{PD1}{80}{L1}{L2}C]	Repeated
Total_Tumor_Void_Frac = Tumor_Void_Fraction + Frac_Vv_Tmr	Repeated
Tumor.[C{CD80}] = 0.001*mole+TC1*[%{CD80}C]	Repeated
Tumor.[C{PD1}] = 0.001*mole+TC1*[%{PD1}C]	Repeated
Tumor.[C{PD1}{CD80}] = 0.001*mole+TC1*[%{CD80}{PD1}C]	Repeated

Parameter and Species Assignment Equation	Assignment Type
Tumor.[C{PD1}{PDL1}] = 0.001*mole+TC1*[%{PD1}{L1}C]	Repeated
Tumor.[C{PD1}{PDL1}{CD80}] = 0.001*mole+TC1*[%{PD1}{80}{L1}C]	Repeated
Tumor.[C{PD1}{PDL1}{PDL2}] = 0.001*mole+TC1*[%{PD1}{L1}{L2}C]	Repeated
Tumor.[C{PD1}{PDL1}{PDL2}{CD80}] = 0.001*mole+TC1*[%{PD1}{80}{L1}{L2}C]	Repeated
Tumor.[C{PD1}{PDL2}] = 0.001*mole+TC1*[%{PD1}{L2}C]	Repeated
Tumor.[C{PD1}{PDL2}{CD80}] = 0.001*mole+TC1*[%{PD1}{80}{PDL2}C]	Repeated
Tumor.[C{PDL1}] = 0.001*mole+TC1*[%{PDL1}C]	Repeated
Tumor.[C{PDL1}{CD80}] = 0.001*mole+TC1*[%{PDL1}{80}C]	Repeated
Tumor.[C{PDL1}{PDL2}] = 0.001*mole+TC1*[%{PDL1}{PDL2}C]	Repeated
Tumor.[C{PDL1}{PDL2}{CD80}] = 0.001*mole+TC1*[%{PDL1}{80}{L2}C]	Repeated
Tumor.[C{PDL2}] = 0.001*mole+TC1*[%{PDL2}C]	Repeated
Tumor.[C{PDL2}{CD80}] = 0.001*mole+TC1*[%{PDL2}{80}C]	Repeated
Tumor.[T{PD1}{80}-{PDL1}{PDL2}C] = 0.001*mole+TC2*[%{PDL1}{PDL2}C]	Repeated
Tumor.[T{PD1}{CD80}-{PDL1}C] = 0.001*mole+TC2*[%{PDL1}C]	Repeated
Tumor.[T{PD1}{L1}{80}-{PD1}{80}{L1}{L2}C] = 0.001*mole+TC2*[%{PD1}{80}{L1}{L2}C]	Repeated
Tumor.[T{PD1}{L1}{80}-{PD1}{80}{L1}C] = 0.001*mole+TC2*[%{PD1}{80}{L1}C]	Repeated
Tumor.[T{PD1}{L1}{80}-{PD1}{L1}{L2}C] = 0.001*mole+TC2*[%{PD1}{L1}{L2}C]	Repeated
Tumor.[T{PD1}{L1}{80}-{PD1}{L1}C] = 0.001*mole+TC2*[%{PD1}{L1}C]	Repeated
Tumor.[T{PD1}{L1}-{PD1}{80}{PDL2}C] = 0.001*mole+TC2*[%{PD1}{80}{PDL2}C]	Repeated
Tumor.[T{PD1}{L1}-{PD1}{L2}C] = 0.001*mole+TC2*[%{PD1}{L2}C]	Repeated
Tumor.[T{PD1}{L1}-{PDL1}{80}{L2}C] = 0.001*mole+TC2*[%{PDL1}{80}{L2}C]	Repeated
Tumor.[T{PD1}{L1}-{PDL1}{80}C] = 0.001*mole+TC2*[%{PDL1}{80}C]	Repeated
Tumor.[T{PD1}{L1}-{PDL2}{80}C] = 0.001*mole+TC2*[%{PDL2}{80}C]	Repeated
Tumor.[T{PD1}-{PDL2}C] = 0.001*mole+TC2*[%{PDL2}C]	Repeated
Tumor.[T{PDL1}-{CD80}{PD1}C] = 0.001*mole+TC2*[%{CD80}{PD1}C]	Repeated
Tumor.[T{PDL1}-{CD80}C] = 0.001*mole+TC2*[%{CD80}C]	Repeated
Tumor.[T{PDL1}-{PD1}C] = 0.001*mole+TC2*[%{PD1}C]	Repeated
Tumor.Cancer = (4/3)*pi*((Starting_Cancer_Diam_mm/2)^2)*((Starting_Cancer_Diam_mm*Tumor_Vol_Ellipse_c_ratio)/2)*(1/((4/3)*pi*((Cancer_Cell_Diam_um/2000)^3)))*(1-Total_Tumor_Void_Frac)*mole	Initial
Tumor.Cancer1 = (Cancer-TC1)*ge(Cancer-TC1,0)	Repeated
Tumor.CTLA4_mabt_ugml = (Tumor.CTLA4_mabt*Ipilimumab_MW)/Vex_Tmr	Repeated
Tumor.Effector_TT_C_Eng = (Effector_TT-TregT_Teff - MDSCsT_Teff - TC2-T_Recover_Can_Dead)*ge(Effector_TT-TC2-T_Recover_Can_Dead,0)	Repeated
Tumor.Effector_TT_Count = Effector_TT + Effector_TT_Res	Repeated
Tumor.Effector_TT_MDSCs = Effector_TT-TregT_Teff - MDSCsT_Teff - TC1	Repeated
Tumor.Effector_TT_per_mL = Effector_TT_Count/Vex_Tmr	Repeated
Tumor.Effector_TT_per_Treg = (Effector_TT_Count/(Cancer*[%T_Tregs_per_Cancer]/100))*ge(Cancer,10*mole)	Repeated
Tumor.Effector_TT_TregT = Effector_TT-TregT_Teff - MDSCsT_Teff - TC1	Repeated
Tumor.mAPCT_EngTregT = mAPC_T+0.001*mole	Repeated
Tumor.MDSC_T = (Cancer*[%T_Tregs_per_Cancer]/100)*[MDSCs_per_Treg] -MDSCsT_Teff	Repeated
Tumor.MDSCsT_EngTeff = MDSCsT_Teff+0.001*mole	Repeated
Tumor.PD1_mabT_ugml = (Tumor.PD1_mabt*Nivolumab_MW)/Vex_Tmr	Repeated
Tumor.PDL1_mabT_ugml = (Tumor.PDL1_mabt*Durvalumab_MW)/Vex_Tmr	Repeated
Tumor.TC2 = TC1*T_per_Cancer_Cell_Int	Repeated
Tumor.Teff_EngMDSC = MDSCsT_Teff+0.001*mole	Repeated
Tumor.Teff_EngTregT = TregT_Teff+0.001*mole	Repeated
Tumor.Tmr_Free_Sites = Vv_Tmr*Adhesion_Density_Tmr-EffT_b_Vasc-EffT_AR_Vasc	Repeated
Tumor.TregT = (((Cancer*[%T_Tregs_per_Cancer]/100)) - TregT_Teff)*(1-[Sig_TrAPCT=Total])	Repeated
Tumor.TregT_EngAPC = mAPC_T+0.001*mole	Repeated
Tumor.TregT_EngTeff = TregT_Teff+0.001*mole	Repeated
Vex_Tmr = (Total_Tumor_Void_Frac - Frac_Vv_Tmr)*Vt_Teff	Repeated
Vt = Vex_Tmr + ml_correct	Repeated
Vt_Teff = ([Cancer_Vol_cm3]*0.001+1E-20)*L	Repeated
Vv_Tmr = Vt_Teff*Frac_Vv_Tmr	Repeated

Table S6 – Model Algebraic Equations (End)

Table S7 – Model Discontinuous Equation Sets (Start)

Trigger	Event Functions	Description
**CTLA4_counter_on<=time**	CTLA4_counter_off = time+0.0625*day;CTLA4mAb = CTLA4mAb_Dose*EndTherapy	CTLA4 counter for dosing

Trigger	Event Functions	Description
**CTLA4_counter_off<=time**	CTLA4_counter_on = time+Dose_sched_CTLA4 - 0.0625*day;CTLA4mAb = 0*(mg/kg)	CTLA4 counter for dosing
**time>=CTLA4_change_schedule**	CTLA4mAb = 0*(mg/kg);CTLA4mAb_Dose = CTLA4mAb_New_Dose;New_sched_CTLA4 = 365*day + time;Dose_sched_CTLA4 = New_sched_CTLA4	CTLA4 change schedule for dosing
**PD1_counter_on<=time**	PD1_counter_off = time+0.0416666*day;PD1mAb = PD1mAb_Dose*EndTherapy	PD1 counter for dosing
**PD1_counter_off<=time**	PD1_counter_on = time+Dose_sched_PD1 - 0.0416666*day;PD1mAb = 0*(mg/kg)	PD1 counter for dosing
**time>=PD1_change_schedule**	PD1mAb = 0*(mg/kg);PD1mAb_Dose = PD1mAb_New_Dose;Dose_sched_PD1 = New_sched_PD1;PD1_change_schedule = 1500*day	PD1 change schedule for dosing
**PDL1_counter_off<=time**	PDL1_counter_on = time+Dose_sched_PDL1 - 0.0416666*day;PDL1mAb = 0*(mg/kg)	PDL1 counter for dosing
**time>=PDL1_change_schedule**	PDL1mAb = 0*(mg/kg);PDL1mAb_Dose = PDL1mAb_New_Dose;Dose_sched_PDL1 = New_sched_PDL1;PDL1_change_schedule = 1500*day	PDL1 change schedule for dosing
**PDL1_counter_on<=time**	PDL1_counter_off = time+0.0416666*day;PDL1mAb = PDL1mAb_Dose*EndTherapy	PDL1 counter for dosing
**Cancer<=1*mole**	TheEnd = 0	End extravasation at tumor death
**Cancer<=1.446759259259259e+05*mole**	EndTherapy = 0;Time_Tumor_Death = time	Stop therapy at tumor death
**Cancer_Diam_mm>=Cancer_mm_Start_Therapy**	CTLA4mAb_Dose = CTLA4_DoseSet*(mg/kg);CTLA4_counter_on = time+0.0001*day;CTLA4_counter_off = CTLA4_counter_on + 0.0625*day;CTLA4_change_schedule = time + 64*day;Cancer_mm_Start_Therapy = Cancer_mm_Start_Therapy*100;Time_Start_Therapy = time;TCytokineHoming = 100	CTLA4 dosing
**Cancer_Diam_mm>=Cancer_mm_Start_Therapy**	CTLA4mAb_Dose = CTLA4_DoseSet*(mg/kg);CTLA4_counter_on = time+0.0001*day;CTLA4_counter_off = CTLA4_counter_on + 0.0625*day;CTLA4_change_schedule = time + 64*day;PD1_counter_on = time+0.0001*day;PD1_counter_off = PD1_counter_on + 0.0416666*day;PD1mAb_Dose = PD1_DoseSet*(mg/kg);PD1_change_schedule = time + 64*day;Cancer_mm_Start_Therapy = Cancer_mm_Start_Therapy*100;Time_Start_Therapy = time;TCytokineHoming = 100	CTLA4 and PD1 dosing
**Cancer_Diam_mm>=Cancer_mm_Start_Therapy**	CTLA4mAb_Dose = CTLA4_DoseSet*(mg/kg);CTLA4_counter_on = time+0.0001*day;CTLA4_counter_off = CTLA4_counter_on + 0.0625*day;CTLA4_change_schedule = time + 64*day;PD1_counter_on = time+85.0001*day;PD1_counter_off = PD1_counter_on + 0.0416666*day;PD1mAb_Dose = PD1_DoseSet*(mg/kg);Cancer_mm_Start_Therapy = Cancer_mm_Start_Therapy*100;Time_Start_Therapy = time;TCytokineHoming = 100	CTLA4 then PD1 dosing
Cancer_Diam_mm>=Cancer_mm_Start_Therapy && time<=150*day	PD1mAb_Dose = PD1_DoseSet*(mg/kg);PD1_counter_on = time+0.0001*day;PD1_counter_off = PD1_counter_on + 0.0416666*day;PD1_change_schedule = time + 71.0001*day;CTLA4_counter_on = time+85.0001*day;CTLA4_counter_off = CTLA4_counter_on + 0.0625*day;CTLA4_change_schedule = time + 64*day + 85.0001*day;CTLA4mAb_Dose = CTLA4_DoseSet*(mg/kg);Cancer_mm_Start_Therapy = Cancer_mm_Start_Therapy*100;Time_Start_Therapy = time;TCytokineHoming = 100	PD1 then CTLA4 dosing: set CTLA4 and PD1 dosing

Trigger	Event Functions	Description
**Cancer_Diam_mm>=Cancer_mm_Start_Therapy**	PD1_counter_on = time+0.0001*day;PD1_counter_off = PD1_counter_on + 0.0416666*day;PD1mAb_Dose = PD1_DoseSet*(mg/kg);Cancer_mm_Start_Therapy = Cancer_mm_Start_Therapy*100;Time_Start_Therapy = time;TCytokineHoming = 100	PD1 dosing
**Cancer_Diam_mm>=Cancer_mm_Start_Therapy**	PDL1mAb_Dose = PDL1_DoseSet*(mg/kg);PD1mAb_Dose = PD1_DoseSet*(mg/kg);PDL1_counter_on = time+0.0001*day;PD1_counter_on = time+0.0001*day;PDL1_counter_off = PDL1_counter_on + 0.0416666*day;PD1_counter_off = PD1_counter_on + 0.0416666*day;Cancer_mm_Start_Therapy = Cancer_mm_Start_Therapy*100;Time_Start_Therapy = time;TCytokineHoming = 100	PD1 and PDL1 dosing
**Cancer_Diam_mm>=Cancer_mm_Start_Therapy**	PDL1_counter_on = time+0.0001*day;PDL1_counter_off = PDL1_counter_on + 0.0416666*day;PDL1mAb_Dose = PDL1_DoseSet*(mg/kg);Cancer_mm_Start_Therapy = Cancer_mm_Start_Therapy*100;Time_Start_Therapy = time;TCytokineHoming = 100	PDL1 dosing
**Cancer_Diam_mm>=Cancer_mm_Start_Therapy**	CTLA4mAb_Dose = CTLA4_DoseSet*(mg/kg);CTLA4_counter_on = time+0.0001*day;CTLA4_counter_off = CTLA4_counter_on + 0.0625*day;CTLA4_change_schedule = time + 64*day;PDL1_counter_on = time+0.0001*day;PDL1_counter_off = PDL1_counter_on + 0.0416666*day;PDL1mAb_Dose = PDL1_DoseSet*(mg/kg);PDL1_change_schedule = time + 64*day;Cancer_mm_Start_Therapy = Cancer_mm_Start_Therapy*100;Time_Start_Therapy = time;TCytokineHoming = 100	CTLA4 and PDL1 dosing
**Cancer_Diam_mm>=Cancer_mm_Start_Therapy**	CTLA4mAb_Dose = CTLA4_DoseSet*(mg/kg);CTLA4_counter_on = time+0.0001*day;CTLA4_counter_off = CTLA4_counter_on + 0.0625*day;CTLA4_change_schedule = time + 64*day;PDL1_counter_on = time+0.0001*day;PDL1_counter_off = PDL1_counter_on + 0.0416666*day;PDL1mAb_Dose = PDL1_DoseSet*(mg/kg);PDL1_change_schedule = time + 64*day;PD1_counter_on = time+0.0001*day;PD1_counter_off = PD1_counter_on + 0.0416666*day;PD1mAb_Dose = PD1_DoseSet*(mg/kg);PD1_change_schedule = time + 64*day;Cancer_mm_Start_Therapy = Cancer_mm_Start_Therapy*100;Time_Start_Therapy = time;TCytokineHoming = 100	CTLA4, PD1 and PDL1 dosing
**time>=Time_Start_Therapy + 163*day**	PD1mAb_New_Dose = PD1_DoseSet*(mg/kg);PD1_change_schedule = 2000*day;PD1mAb_Dose = PD1_DoseSet*(mg/kg);TCytokineHoming = 100	PD1 then CTLA4 therapy: set delayed PD1 dosing
**Cancer_Diam_mm>=Cancer_mm_Start_Therapy**	Time_Start_Therapy = time	Time to start therapy once tumor diameter reaches a certain threshold

Table S7 – Model Discontinuous Equation Sets (End)

Table S8 – Model Compartment Volumes (Start)

Compartment	Variable	Volume	Units	Reference	Description
**Blood-Lymph**	Vc_CTLA4	2.727814034	liter	[15]	Volume of the central compartment for Anti-CTLA-4
**Blood-Lymph**	Vc_PD1	2.716739643	liter	[25]	Volume of the central compartment for Anti-PD-1
**Blood-Lymph**	Vc_PDL1	4.051599814	liter	[26, 27]	Volume of the central compartment for Anti-PD-L1

Compartment	Variable	Volume	Units	Reference	Description
**Blood-Lymph**	Vc_Teff	2.7	liter	[13, 93]	Volume of blood through which Effector T cells circulate throughout the body
**Liv_Spln_GI**	Vv_GI	43	ml	[13]	Vasculature volume of GI for Effector T cell trafficking
**Liv_Spln_GI**	Vext_Liver	361.8	ml	[13]	Volume of Liver interstitium for Effector T cell trafficking
**Liv_Spln_GI**	Vv_Liver	180.9	ml	[13]	Vasculature volume of Liver for Effector T cell trafficking
**Liv_Spln_GI**	Vext_Spleen	34.7	ml	[13]	Volume of Spleen interstitium for Effector T cell trafficking
**Liv_Spln_GI**	Vv_Spleen	17	ml	[13]	Vasculature volume of Spleen for Effector T cell trafficking
**Lungs**	Vext_Lungs	299.7	ml	[13]	Volume of Lungs interstitium for Effector T cell trafficking
**Lungs**	Vv_Lungs	99.9	ml	[13]	Vasculature volume of Lungs for Effector T cell trafficking
**Lymph_Node**	Vext_LN	34.7	ml	[13]	Volume of Lymph Node interstitium for Effector T cell trafficking
**Lymph_Node**	VL	5.2	liter	[56, 94]	Volume of total Lymph for antibody PK
**Lymph_Node**	Vtdln	5.2	liter	[56, 94]	Volume of total Lymph for antibody PK
**Lymph_Node**	Vv_LN	17	ml	[13]	Vasculature volume of Lymph Node for Effector T cell trafficking
**Peripheral**	ISF	15.9	liter	[56]	Volume of interstitial fluid (ISF) is tissues for antibody PK
**Peripheral**	Vv_Periph	1355.4	ml	[13]	Vasculature volume of Peripheral (other tissue not accounted for) for Effector T cell trafficking
**Tumor**	Vex_Tmr	~50% (void space) of total tumor volume	ml	[13] Calculated dynamically	Volume of Tumor interstitium
**Tumor**	Vt_Teff	Based on Cancer_Vol_cm3	liter	Calculated dynamically	Total tumor volume calculated from cancer cell number, void space and tumor vasculature
**Tumor**	Vv_Tmr	~7% of total tumor volume	ml	[13] Calculated dynamically	Vasculature volume of Tumor based on fraction of total tumor volume

Table S8 – Model Compartment Volumes (End)

Table S9 – Parameters Varied in Virtual Clinical Trials (Start)

Parameter Name in Model	Value Range	Units	Source	Description
**%PDL1_Exp_Cancer**	40-90; Median: 75	dimensionless	[11]	Percent of cancer cells of total cancer cells that are PD-L1 positive that interact with the Effector T cells in the tumor compartment.
**%T_Tregs_per_Cancer**	0-0.25; Median: 0.13	dimensionless	Assumed as recruited by tumor	Defines the percentage of Regulatory T cells in the tumor compartment as a percent of total tumor cells.
**%_Tr_LN**	5-35; Median: 20	dimensionless	[1, 3-5]	Percent Regulatory T cells of total T cells in each lymph node.
**Antigen_Intensity**	Assigned from 0.8-1.0 for melanoma (on a scale of 0.1-1.0); Median: 0.9	dimensionless	Upper range of cancer types [14]	Defines the strength of the TAA/TSA that are involved in priming of the T cells in the lymph nodes. Also known as antigen strength.
**AntSpread**	x = 2 (Where it is used as 10^x)	dimensionless	Estimated during fitting to anti-PD-1 data	Sets the number of TAA/TSA released per cancer cell upon natural death and decay or following cytotoxic killing. The value is used to set a multiple of cancer cells in the formation of debris for APC maturation.
**Cancer_mm_Start_Therapy**	15-80; Median: 40	mm	Approximated based on reported literature range	Diameter of the tumor when to start the therapeutic regimen.
**Chemokine**	100	dimensionless	Estimated during fitting to anti-PD-1 data	The chemokine factor to promote extravasation of effector T cells into the tumor.

Parameter Name in Model	Value Range	Units	Source	Description
**T_Cell_Clonality**	10-130; Median: 70	cell	[28, 30, 85]	Number of Naïve T cells populations specific for different tumor antigens; in other words; the number of anti-tumor T cell clones in each lymph node.

Table S9 – Parameters Varied in Virtual Clinical Trials (End)

Table S10 – Parameters Varied in Sensitivity Analysis (Start)

Parameter Name in Model	Value Range	Units	Source	Description
**%CD80_Exp_Cancer**	1-100	dimensionless	[6-8]	Percent of cancer cells of total cancer cells that are CD80 positive that interact with the Effector T cells in the tumor compartment.
**%PD1_Exp_Cancer**	1-100	dimensionless	[9]	Percent of cancer cells of total cancer cells that are PD-1 positive that interact with the Effector T cells in the tumor compartment.
**%PDL1_Exp_Cancer**	1-100	dimensionless	[11]	Percent of cancer cells of total cancer cells that are PD-L1 positive that interact with the Effector T cells in the tumor compartment.
**%PDL2_Exp_Cancer**	1-100	dimensionless	[12]	Percent of cancer cells of total cancer cells that are PD-L2 positive that interact with the Effector T cells in the tumor compartment.
**%T_Tregs_per_Cancer**	0-0.25	dimensionless	Assumed as recruited by tumor	Defines the percentage of Regulatory T cells in the tumor compartment as a percent of total tumor cells.
**%_Tr_LN**	5-35	dimensionless	[1, 3-5]	Percent Regulatory T cells of total T cells in each lymph node.
**Antigen_Intensity**	Assigned from 0.8-1.0 for melanoma (on a scale of 0.1-1.0)	dimensionless	Upper range of cancer types [14]	Defines the strength of the TAA/TSA that are involved in priming of the T cells in the lymph nodes. Also known as antigen strength.
**AntSpread**	x = 1-5 (Where it is used as 10^x)	dimensionless	Estimated during fitting to anti-PD-1 data and varied across range	Sets the number of TAA/TSA released per cancer cell upon natural death and decay or following cytotoxic killing. The value is used to set a multiple of cancer cells in the formation of debris for APC maturation.
**Cancer_mm_Start_Therapy**	5-80	mm	Assigned based on reported literature range	Longest diameter of the tumor when to start the therapeutic regimen.
**Chemokine**	50-500	dimensionless	Estimated during anti-PD-1 therapy fitting; varied in sensitivity analysis	The chemokine factor to promote extravasation of effector T cells into the tumor.
**CTLA4_DoseSet**	0.3-10	mg/kg	Based on regimen	Dose of anti-CTLA-4 therapy
**mAPC_activation_level**	0.1-1.0	dimensionless	[13]	The fraction of CD80 and CD86 expression on the mAPCs from 0.1-1.
**mAPC_Debis_T_Inact**	0.05-0.95	dimensionless	Varied across range (5-95%) and estimated during fitting to anti-PD-1 data	The fraction of tumor debris that is considered to not be transported to the lymph nodes and is removed from the system.
**PD1_DoseSet**	0.03-2	mg/kg	Based on regimen	Dose administered of anti-PD-1 therapy.
**PDL1_DoseSet**	0.003-0.1	mg/kg	Based on regimen	Dose administered of anti-PD-L1 therapy.
**Rate_Tumor_Growth**	64-144 day doubling time	1/day	[81]	Proliferation rate of cancer cells that defines the volumetric doubling time of the tumor.<br>64 day metastatic doubling: 0.010955675075414<br>144 day non-metastatic doubling: 0.004913635262592.

Parameter Name in Model	Value Range	Units	Source	Description
**T_Cell_Clonality**	5-130	cell	[28, 30, 85]	Number of Naïve T cells populations specific for different tumor antigens; in other words; the number of anti-tumor T cell clones in each lymph node.

Table S10 Parameters Varied in Sensitivity Analysis (End)

Table S11 – Conditions Specific to Each Clinical Scenario Simulated (Start)

Fig.	Tiral	Dose/Regimen	Patients Simulated	Parameter ranges and medians
**2**	Anti-PD-1	3 mg/kg, Q2W	200	Same for all simulations: Table S9 for references and ranges. Latin Hypercube Sampling (LHS) with equal distribution was utilized for simulations.
**3**	Anti-PD-1 with Anti-CTLA-4	3 mg/kg anti-CTLA-4 and 1 mg/kg anti-PD-1<br>Q3W, 4 doses combo; while anti-PD-1, 8 doses; then, combo Q12W	47	
**4**	Anti-PD-L1	20 mg/kg, Q2W	200	
**5**	Anti-PD-1 and Anti-CTLA-4 Sequential Therapy	3 mg/kg anti-CTLA-4 and 3 mg/kg anti-PD-1<br>a) Q3W anti-CTLA-4, 4 doses; Q2W anti-PD-1<br>b) Q2W anti-PD-1, 6 doses; Q3W anti-CTLA-4, 4 doses; Q2W anti-PD-1	200	
**S1**	Anti-CTLA-4	3 and 10 mg/kg, Q3W, 4 doses; then, Q12W	200	
**S2**	Anti-PD-L1 with Anti-CTLA-4	1 mg/kg anti-CTLA-4 and 20 mg/kg anti-PD-L1<br>Q3W, 4 doses combo; anti-PD-L1 Q2W	200	
**S3**	Anti-PD-1 with Anti-PD-L1	3 mg/kg anti-PD-1 and 20 mg/kg anti-PD-L1, Q2W	200	

Table S11 – Conditions Specific to Each Clinical Scenario Simulated (End)

References:

1. Battaglia, A., et al., *Lymphocyte populations in human lymph nodes. Alterations in CD4+ CD25+ T regulatory cell phenotype and T-cell receptor Vβ repertoire.* Immunology, 2003. **110**(3): p. 304-12.
2. Backteman, K., et al., *Lymphocyte subpopulations in lymph nodes and peripheral blood: a comparison between patients with stable angina and acute coronary syndrome.* PLoS One, 2012. **7**(3): p. e32691.
3. Höfer, T., O. Krichevsky, and G. Altan-Bonnet, *Competition for IL-2 between Regulatory and Effector T Cells to Chisel Immune Responses.* Front Immunol., 2012. **3**: p. 1-9.
4. Viguier, M., et al., *Foxp3 Expressing CD4+CD25high Regulatory T Cells Are Overrepresented in Human Metastatic Melanoma Lymph Nodes and Inhibit the Function of Infiltrating T Cells.* J Immunol., 2004. **173**(2): p. 1444-53.
5. Jandus, C., et al., *Selective accumulation of differentiated FOXP3+ CD4+ T cells in metastatic tumor lesions from melanoma patients compared to peripheral blood.* Cancer Immunol Immunother., 2008. **57**(12): p. 1795-805.
6. Tirapu, I., et al., *Low surface expression of B7-1 (CD80) is an immunoescape mechanism of colon carcinoma.* Cancer Res., 2006. **66**(4): p. 2442-50.
7. *CD80 in Melanoma*. 2015; Available from: http://www.proteinatlas.org/ENSG00000121594-CD80/cancer/tissue/melanoma.
8. Uhlén, M., et al., *Proteomics. Tissue-based map of the human proteome.* Science, 2015. **347**(6220): p. 1260419.

9. Kleffel, S., et al., *Melanoma cell-intrinsic PD-1 receptor functions promote tumor growth.* Cell, 2015. **162**(6): p. 1242-56.
10. Chemnitz, J.M., et al., *SHP-1 and SHP-2 associate with immunoreceptor tyrosine-based switch motif of programmed death 1 upon primary human T cell stimulation, but only receptor ligation prevents T cell activation.* J Immunol., 2004. **173**(2): p. 945-54.
11. Madore, J., et al., *PD-L1 expression in melanoma shows marked heterogeneity within and between patients: implications for anti-PD-1/PD-L1 clinical trials.* Pigment Cell Melanoma Res., 2015. **28**(3): p. 245-53.
12. Obeid, J.M., et al., *PD-L1, PD-L2 and PD-1 expression in metastatic melanoma: Correlation with tumor-infiltrating immune cells and clinical outcome.* Oncoimmunology, 2016. **5**(11): p. e1235107.
13. Zhu, H., et al., *Physiologically based kinetic model of effector cell biodistribution in mammals: implications for adoptive immunotherapy.* Cancer Res., 1996. **56**(16): p. 3771-81.
14. Yarchoan, M., A. Hopkins, and E.M. Jaffee, *Tumor Mutational Burden and Response Rate to PD-1 Inhibition.* N Engl J Med., 2017. **377**(25): p. 2500-2501.
15. Feng, Y., et al., *Model-based clinical pharmacology profiling of ipilimumab in patients with advanced melanoma.* Br J Clin Pharmacol., 2014. **78**(1): p. 106-17.
16. Lyng, H., O. Haraldseth, and E.K. Rofstad, *Measurement of Cell Density and Necrotic Fraction in Human Melanoma Xenografts by Diffusion Weighted Magnetic Resonance Imaging.* Magn Reson Med., 2000. **43**(6): p. 828-36.
17. Thurber, G.M., M.M. Schmidt, and K.D. Wittrup, *Antibody tumor penetration: transport opposed by systemic and antigen-mediated clearance.* Adv Drug Deliv Rev., 2008. **60**(12): p. 1421-34.
18. Ritter, A.T., et al., *Actin Depletion Initiates Events Leading to Granule Secretion at the Immunological Synapse.* Immunity, 2015. **42**(5): p. 864-76.
19. Miller, M.J., et al., *T cell repertoire scanning is promoted by dynamic dendritic cell behavior and random T cell motility in the lymph node.* Proc Natl Acad Sci U S A., 2004. **101**(4): p. 998–1003.
20. Mrass, P., et al., *Random migration precedes stable target cell interactions of tumor-infiltrating T cells.* J Exp Med., 2006. **203**(12): p. 2749-61.
21. Ewa Bryl, et al., *Modulation of CD28 expression with anti-tumor necrosis factor alpha therapy in rheumatoid arthritis.* Arthritis Rheum., 2005. **52**(10): p. 2996-3003.
22. Unternaehrer, J.J., et al., *The tetraspanin CD9 mediates lateral association of MHC class II molecules on the dendritic cell surface.* Proc Natl Acad Sci U S A., 2007. **104**(1): p. 234-9.
23. Haile, S.T., et al., *Tumor Cell Programmed Death Ligand 1-Mediated T Cell Suppression Is Overcome by Coexpression of CD80.* J Immunol., 2011. **186**(12): p. 6822-9.
24. Tatari-Calderone, Z., et al., *Acquisition of CD80 by human T cells at early stages of activation: functional involvement of CD80 acquisition in T cell to T cell interaction.* J Immunol., 2002. **169**(11): p. 6162-9.
25. BMS, *Clinical Pharmacology BLA Review - Opdivo (nivolumab)*. 2014, CENTER FOR DRUG EVALUATION AND RESEARCH (CDER): CLINICAL PHARMACOLOGY AND BIOPHARMACEUTICS REVIEW(S). p. 1-28.
26. Cogswell, J.P., et al., *Cancer immunotherapy by disrupting pd-1/pd-l1 signaling*. 2013, Google Patents.
27. Deng, R., et al., *Preclinical pharmacokinetics, pharmacodynamics, tissue distribution, and tumor penetration of anti-PD-L1 monoclonal antibody, an immune checkpoint inhibitor.* MAbs, 2016. **8**(3): p. 593-603.
28. Qia, Q., et al., *Diversity and clonal selection in the human T-cell repertoire.* Proc Natl Acad Sci U S A., 2014. **111**(36): p. 13139–44.

29. Jenkins, M.K. and J.J. Moon, *The Role of Naive T Cell Precursor Frequency and Recruitment in Dictating Immune Response Magnitude.* J Immunol., 2012. **188**(9): p. 4135-40.
30. Zarnitsyna, V.I., et al., *Estimating the diversity, completeness, and cross-reactivity of the T cell repertoire.* Front Immunol., 2013. **4**.
31. Egen, J.G. and J.P. Allison, *Cytotoxic T Lymphocyte Antigen-4 Accumulation in the Immunological Synapse Is Regulated by TCR Signal Strength.* Immunity, 2002. **16**(1): p. 23-35.
32. Linsley, P.S., et al., *Coexpression and functional cooperation of CTLA-4 and CD28 on activated T lymphocytes.* J Exp Med., 1992. **176**(6): p. 1595-604.
33. Read S, G.R., Izcue A, Robinson N, Mandelbrot D, Francisco L, Sharpe AH, Powrie F, *Blockade of CTLA-4 on CD4+CD25+ regulatory T cells abrogates their function in vivo.* J Immunol., 2006. **177**(7): p. 4376-83.
34. Palsson, S., et al., *The development of a fully-integrated immune response model (FIRM) simulator of the immune response through integration of multiple subset models.* BMC Syst Biol., 2013. **7**.
35. De Boer RJ, H.P., Dullens HF, De Weger RA, Den Otter W., *Macrophage T lymphocyte interactions in the anti-tumor immune response: a mathematical model.* J Immunol., 1985. **134**(4): p. 2748-58.
36. Kanehisa, M., et al., *KEGG: new perspectives on genomes, pathways, diseases and drugs.* Nucleic Acids Res., 2017. **45**(D1): p. D353-D361.
37. Kanehisa, M., et al., *KEGG as a reference resource for gene and protein annotation.* Nucleic Acids Res., 2016. **44**(D1): p. D457-62.
38. Kanehisa, M. and S. Goto, *KEGG: kyoto encyclopedia of genes and genomes.* Nucleic Acids Res., 2000. **28**(1): p. 27-30.
39. Wikipedia contributors. *Durvalumab*. 2016 8 December 2016 18:37 UTC; Available from: https://en.wikipedia.org/wiki/Durvalumab.
40. Henrickson, S.E., et al., *T cell sensing of antigen dose governs interactive behavior with dendritic cells and sets a threshold for T cell activation.* Nat Immunol., 2008. **9**(3): p. 282-291.
41. Marino, S. and D.E. Kirschner, *The human immune response to Mycobacterium tuberculosis in lung and lymph node.* J Theor Biol., 2004. **227**(4): p. 463-86.
42. Klebanoff, C.A., L. Gattinoni, and N.P. Restif, *CD8+ T-cell memory in tumor immunology and immunotherapy.* Immunol Rev., 2006. **211**: p. 214-24.
43. Northrop, J.K. and H. Shen, *CD8+ T-cell memory: only the good ones last.* Curr Opin Immunol., 2004. **16**(4): p. 451-5.
44. Macallan, D.C., et al., *Rapid Turnover of Effector–Memory CD4+ T Cells in Healthy Humans.* J Exp Med., 2004. **200**(2): p. 255-60.
45. De Boer RJ, et al., *Turnover rates of B cells, T cells, and NK cells in simian immunodeficiency virus-infected and uninfected rhesus macaques.* J Immunol., 2003. **170**(5): p. 2479-87.
46. Qureshi, O.S., et al., *Constitutive Clathrin-mediated Endocytosis of CTLA-4 Persists during T Cell Activation.* J Biol Chem., 2012. **287**(12): p. 9429-40.
47. Kaur, S., O.S. Qureshi, and D.M. Sansom, *Comparison of the Intracellular Trafficking Itinerary of CTLA-4 Orthologues.* PLoS One, 2013. **8**(4): p. e60903.
48. Pentcheva-Hoang, T., et al., *Programmed death-1 concentration at the immunological synapse is determined by ligand affinity and availability.* Proc Natl Acad Sci U S A., 2007. **104**(45): p. 17765-70.
49. Linsley, P.S., et al., *Intracellular Trafficking of CTLA-4 and Focal Localization Towards Sites of TCR Engagement.* Immunity, 1996. **4**(6): p. 535-43.
50. Jafarnejad, M., et al., *Modeling Lymph Flow and Fluid Exchange with Blood Vessels in Lymph Nodes.* Lymphat Res Biol., 2015. **13**(4): p. 234-247.

51. Pillis, L.G.d., A.E. Radunskaya, and C.L. Wiseman, *A validated mathematical model of cell-mediated immune response to tumor growth.* Cancer Res., 2005. **65**(17): p. 7950-8.
52. Patel, S.P. and S.E. Woodman, *Profile of ipilimumab and its role in the treatment of metastatic melanoma.* Drug Des Devel Ther., 2011. **5**: p. 489-95.
53. Wikipedia contributors. *Ipilimumab*. 2017 26 February 2017 03:23 UTC; Available from: https://en.wikipedia.org/w/index.php?title=Ipilimumab&oldid=767469815.
54. Wild, M.K., et al., *Dependence of T Cell Antigen Recognition on the Dimensions of an Accessory Receptor–Ligand Complex.* J Exp Med., 1999. **190**(1): p. 31-41.
55. Burroughs, N.J. and C. Wulfing, *Differential Segregation in a Cell-Cell Contact Interface: The Dynamics of the Immunological Synapse.* Biophys J., 2002. **83**(4): p. 1784-96.
56. Cao, Y., J.P. Balthasar, and W.J. Jusko, *Second-generation minimal physiologically-based pharmacokinetic model for monoclonal antibodies.* J Pharmacokinet Pharmacodyn., 2013. **40**(5): p. 597-607.
57. Ruedl C, K.P., Bachmann M, Hess M, Karjalainen K., *Anatomical origin of dendritic cells determines their life span in peripheral lymph nodes.* 165, 2000. **9**(4910-6).
58. Domínguez PM, A.C., *Differentiation and function of mouse monocyte-derived dendritic cells in steady state and inflammation.* Immunol Rev., 2010. **234**(1): p. 90-104.
59. Henrickson, S.E., et al., *In vivo imaging of T cell priming.* Sci Signal, 2008. **1**(12): p. pt2.
60. Mempel, T.R., S.E. Henrickson, and U.H.v. Andrian, *T-cell priming by dendritic cells in lymph nodes occurs in three distinct phases.* Nature, 2004. **427**(6970): p. 154-9.
61. Stoll, S., et al., *Dynamic imaging of T cell-dendritic cell interactions in lymph nodes.* Science, 2002. **296**(5574): p. 1873-6.
62. Miller, M.J., et al., *Imaging the Single Cell Dynamics of CD4+ T Cell Activation by Dendritic Cells in Lymph Nodes.* J Exp Med., 2004. **200**(7): p. 847-56.
63. Baverel, P.G., et al., *Population Pharmacokinetics of Durvalumab in Cancer Patients and Association With Longitudinal Biomarkers of Disease Status.* Clin Pharmacol Ther., 2018. **103**(4): p. 631-642.
64. Collins, A.V., et al., *The Interaction Properties of Costimulatory Molecules Revisited.* Immunity, 2002. **17**(2): p. 201-10.
65. EMA, *Assessment Report For Yervoy (ipilimumab)* 2011, European Medicines Agency (EMA)/Committee for Medicinal Products for Human Use (CHMP).
66. Cheng, X., et al., *Structure and Interactions of the Human Programmed Cell Death 1 Receptor.* J Biol Chem., 2013. **288**(17): p. 11771-85.
67. EMA, *Nivolumab BMS, INN-nivolumab*. 2015, European Medicines Agency (EMA)/Committee for Medicinal Products for Human Use (CHMP).
68. Finley, S.D., et al., *Pharmacokinetics and pharmacodynamics of VEGF-neutralizing antibodies.* BMC Syst Biol. , 2011. **5**.
69. Norbury, C.C., et al., *Visualizing priming of virus-specific CD8+ T cells by infected dendritic cells in vivo.* Nat Immunol., 2002. **3**(3): p. 265-71.
70. Haghighi, H.R., et al., *Identification of a Dual-Specific T Cell Epitope of the Hemagglutinin Antigen of an H5 Avian Influenza Virus in Chickens.* PLoS ONE, 2009. **4**(11): p. e7772.
71. Sims, T.N., et al., *Opposing effects of PKCtheta and WASp on symmetry breaking and relocation of the immunological synapse.* Cell, 2007. **129**(4): p. 773-85.
72. Celli, S., et al., *How many dendritic cells are required to initiate a T-cell response?* Blood, 2012. **120**(19): p. 3945-8.
73. Currana, M.A., et al., *PD-1 and CTLA-4 combination blockade expands infiltrating T cells and reduces regulatory T and myeloid cells within B16 melanoma tumors.* Proc Natl Acad Sci U S A., 2010. **107**(9): p. 4275-80.

74. Wikipedia contributors. *Nivolumab*. 2017 27 February 2017 13:59 UTC; Available from: https://en.wikipedia.org/wiki/Nivolumab.
75. Farzad, Z., et al., *Lymphocyte subset alterations in nodes regional to human melanoma.* Cancer Res., 1990. **50**(12): p. 3585-8.
76. Willard-Mack, C.L., *Normal Structure, Function, and Histology of Lymph Nodes.* Toxicol Pathol., 2006. **34**(5): p. 409-24.
77. Native American Cancer Researc (NACR). *Introduction to Lymphedema Branch*. 2016; Available from: http://natamcancer.org/lymphedema-intro.html.
78. Wikipedia contributors. *List of lymph nodes of the human body*. 2016 11 December 2016 00:14 UTC; Available from: https://en.wikipedia.org/wiki/List_of_lymph_nodes_of_the_human_body.
79. Bousso, P. and E. Robey, *Dynamics of CD8+ T cell priming by dendritic cells in intact lymph nodes.* Nat Immunol., 2003. **4**(6): p. 579-85.
80. Lee, K.-H., et al., *T cell receptor signaling precedes immunological synapse formation.* Science, 2002. **295**(5559): p. 1539-42.
81. Carlson, J.A., *Tumor doubling time of cutaneous melanoma and its metastasis.* Am J Dermatopathol., 2003. **25**(4): p. 291-9.
82. Bousso, P., *T-cell activation by dendritic cells in the lymph node: lessons from the movies.* Nat Rev Immunol., 2008. **8**(9): p. 675-84.
83. Obst, R., *The timing of T cell priming and cycling.* Front Immunol., 2015. **6**.
84. Castellino, F., et al., *Chemokines enhance immunity by guiding naive CD81 T cells to sites of CD41 T cell–dendritic cell interaction.* Nature, 2006. **440**(7086): p. 890-5.
85. Tumeh, P.C., et al., *PD-1 blockade induces responses by inhibiting adaptive immune resistance.* Nature, 2014. **515**(7528): p. 568-71.
86. Miller, M.J., et al., *Two-Photon Imaging of Lymphocyte Motility and Antigen Response in Intact Lymph Node.* Science, 2002. **296**(5574): p. 1869-73.
87. Baey, A.d. and A. Lanzavecchia, *The role of aquaporins in dendritic cell macropinocytosis.* J Exp Med., 2000. **191**(4): p. 743-8.
88. Sallusto, F., et al., *Dendritic cells use macropinocytosis and the mannose receptor to concentrate macromolecules in the major histocompatibility complex class II compartment: downregulation by cytokines and bacterial products.* J Exp Med., 1995. **182**(2): p. 389-400.
89. Matheu, M.P., et al., *Imaging regulatory T cell dynamics and suppression of T cell priming mediated by CTLA4.* Nat Commun., 2015. **6**: p. 6219.
90. Papatriantafyllou, M., *Trafficking: Effector T cells cross the line.* Nat Rev Immunol., 2011. **12**(2): p. 74.
91. Shulman, Z., et al., *Transendothelial migration of lymphocytes mediated by intraendothelial vesicle stores rather than by extracellular chemokine depots.* Nat Immunol., 2011. **13**(1): p. 67-76.
92. Voss, B., et al., *Tumor Volume as a Prognostic Factor in Resectable Malignant Melanoma.* Dermatology, 2014. **228**(1): p. 66-70.
93. Dean, L., *Chapter 1, Blood and the cells it contains*, in *Blood Groups and Red Cell Antigens*. 2005, Bethesda (MD): National Center for Biotechnology Information (US): Internet.
94. Baldazzi, V., et al., *Modeling lymphocyte homing and encounters in lymph nodes.* BMC Bioinformatics, 2009. **10**: p. 1-11.

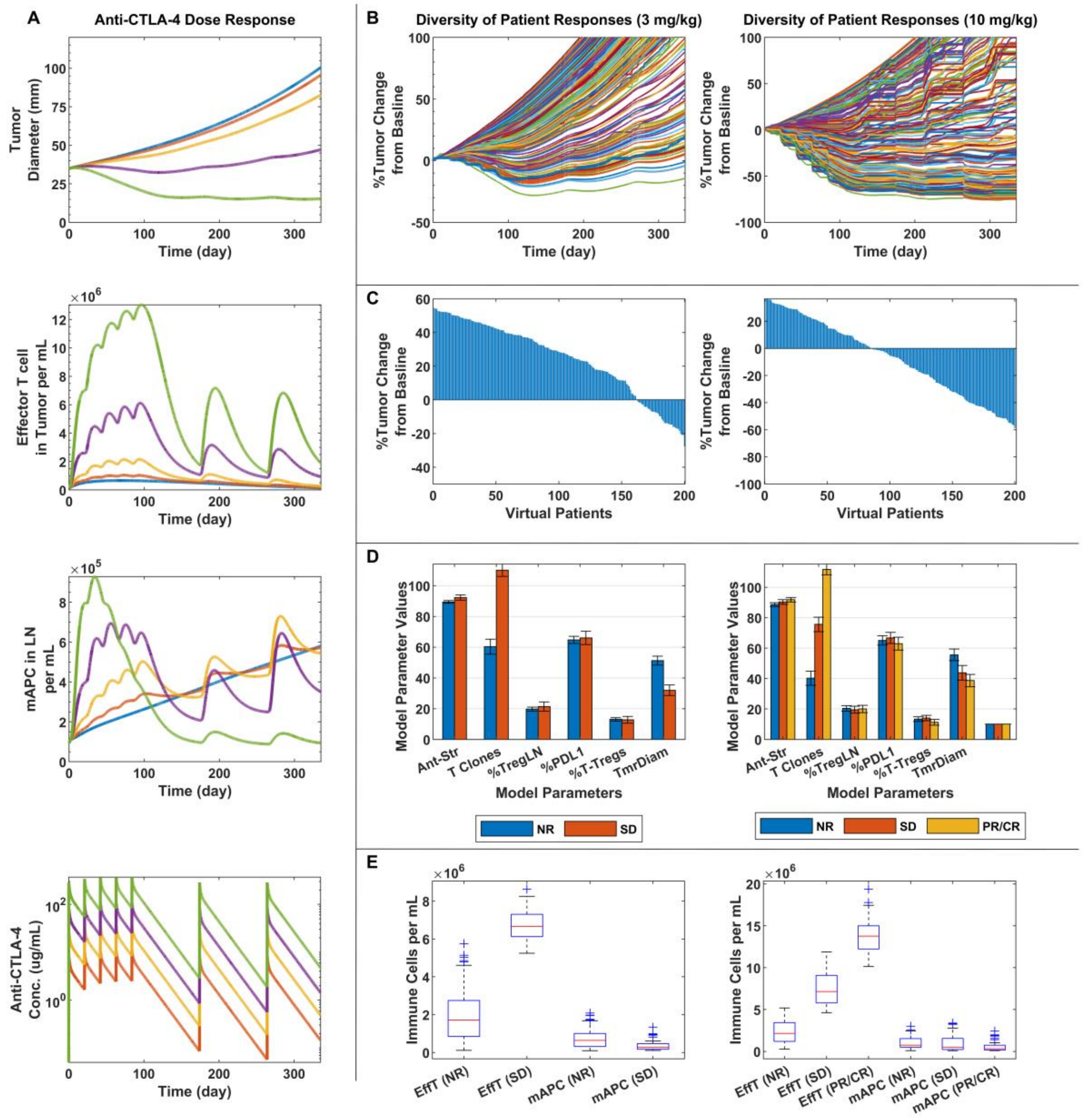

Figure S1. Dose response anti-CTLA-4 mono-therapy. A) From top to bottom: Tumor response to anti-CTLA-4 therapy at doses of 0.3, 1, 3 and 10 mg/kg as represented by the colors in the bottom figure in ascending order; the blue line indicates no therapy in the top figure. Then, Effector T cell density in the tumor (second from the top), mAPC density in the lymph nodes (third from the top) and finally, the PK of anti-CTLA-4 at the given doses. **B)** Diversity of tumor response for 3 mg/kg (left) and 10 mg/kg (right). **C)** Waterfall plot of VPs at 3 mg/kg (left) and 10 mg/kg (right). **D)** Bar graph comparison parameters varied in model for each type of responder at 3 mg/kg (left) and 10 mg/kg (right). **E)** Max Effector T cell density in the tumor and average mAPC density in the lymph nodes for each responder category at 3 mg/kg (left) and 10 mg/kg (right).

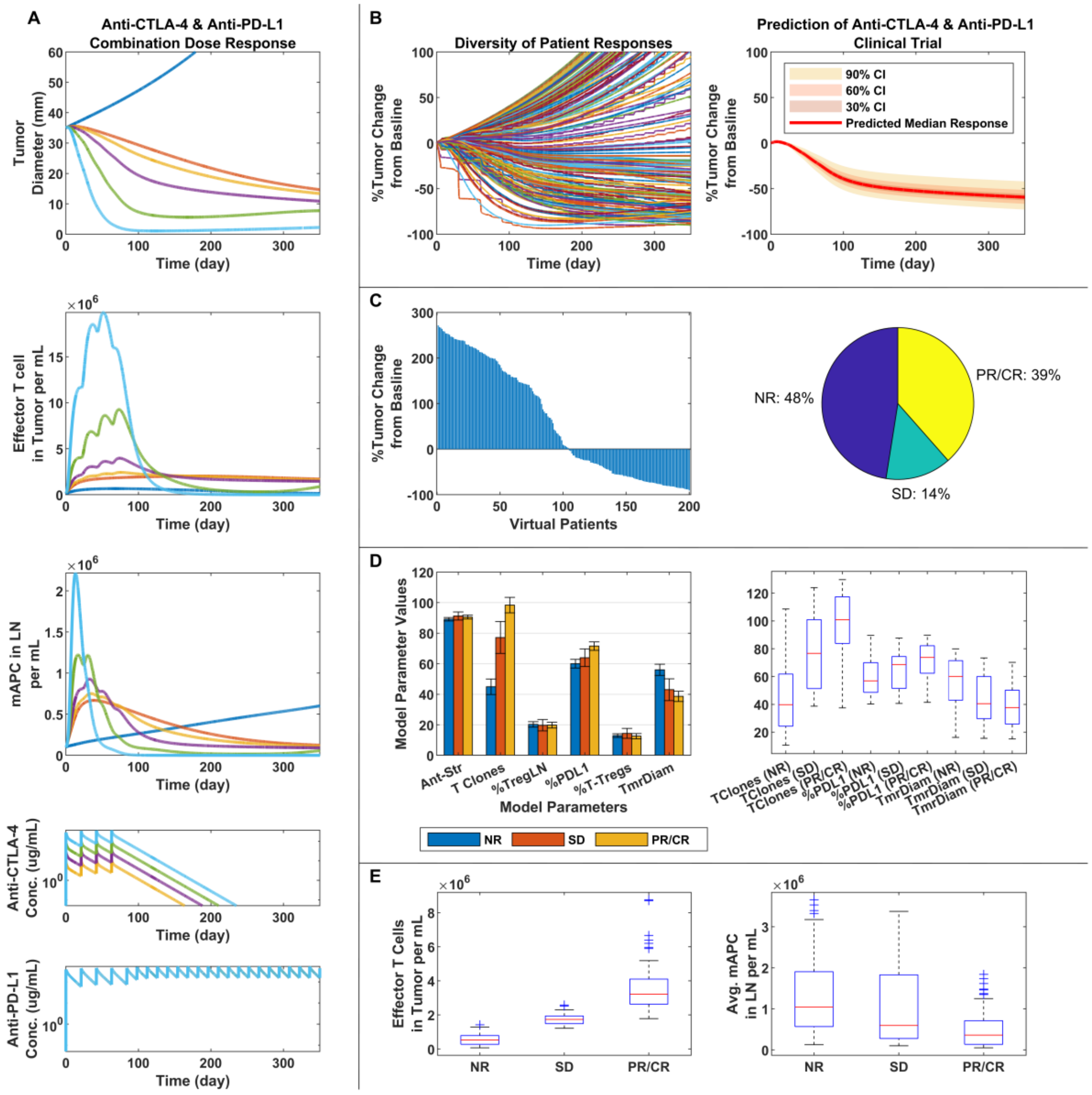

Figure S2. Dose response and clinical validation of anti-CTLA-4/anti-PD-L1 combo-therapy. A) From top to bottom: Tumor response to combination therapy at doses of 0.3, 1, 3 and 10 mg/kg of anti-CTLA-4, as represented by the colors in the second from the bottom figure in ascending order and 20 mg/kg for anti-PD-L1 was used for all simulations; the blue line indicates no therapy (top figure), and orange indicates only anti-PD-L1. Then, Effector T cell density in the tumor (second from the top), mAPC density in the lymph nodes (third from the top) and finally, the PK of anti-CTLA-4 and lastly, anti-PD-L1 at the given doses. For all following figures, 20 mg/kg anti-PD-L1 and 1 mg/kg anti-CTLA-4 were used, following the same regimen. **B)** Diversity of tumor response (left), prediction of median clinical response data (right). **C)** Waterfall plot of VPs (left) and pie chart (right) with percent of virtual non-responders (NR), stable disease (SD) and partial or complete responders (PR/CR). **D)** Bar graph comparison parameters varied in model for each responder type (left) and box plots of significant differentiators (right). **E)** Max

Effector T cell density in the tumor (left) and average mAPC density in the lymph nodes (right) for each responder category.

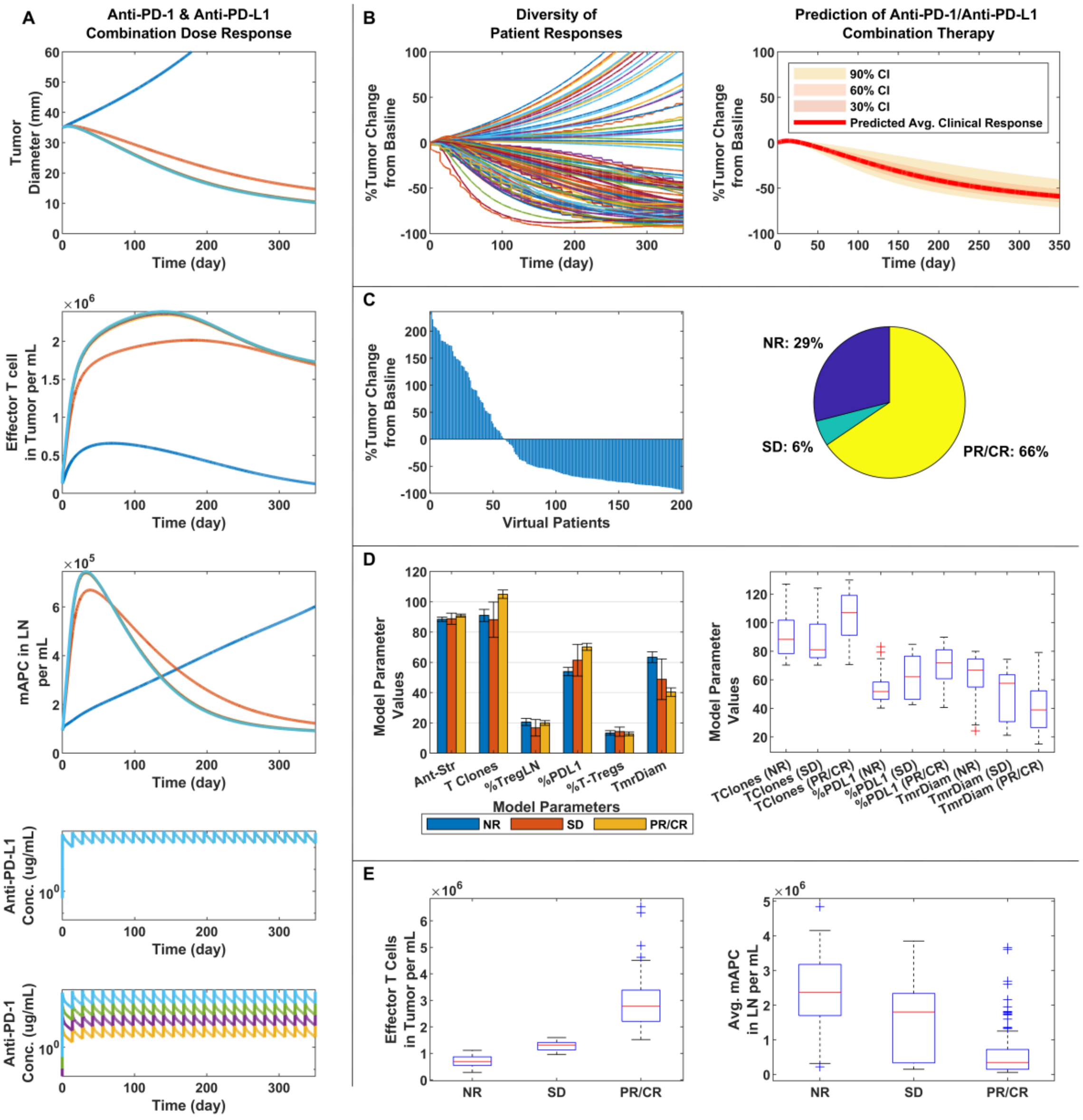

Figure S3. Dose response and clinical validation of anti-PD-1/anti-PD-L1 combo-therapy. A) From top to bottom: Tumor response to combination therapy at doses of 0.3, 1, 3 and 10 mg/kg of anti-PD-1, as represented by the colors in the bottom figure in ascending order and 20 mg/kg for anti-PD-L1 was used for all simulations; the blue line indicates no therapy (top figure), and orange indicates only anti-PD-L1. Then, Effector T cell density in the tumor (second from the top), mAPC density in the lymph nodes (third from the top) and finally, the PK of anti-PD-L1 and lastly, anti-PD-1 at the given doses. For all following

figures, 20 mg/kg anti-PD-L1 and 3 mg/kg anti-PD-1 was used, following the same regimen. **B)** Diversity of tumor response (left), prediction of median clinical response data (right). **C)** Waterfall plot of VPs (left) and pie chart (right) with percent of virtual non-responders (NR), stable disease (SD) and partial or complete responders (PR/CR). **D)** Bar graph comparison parameters varied in model for each responder type (left) and box plots of significant differentiators (right). **E)** Max Effector T cell density in the tumor (left) and average mAPC density in the lymph nodes (right) for each responder category.

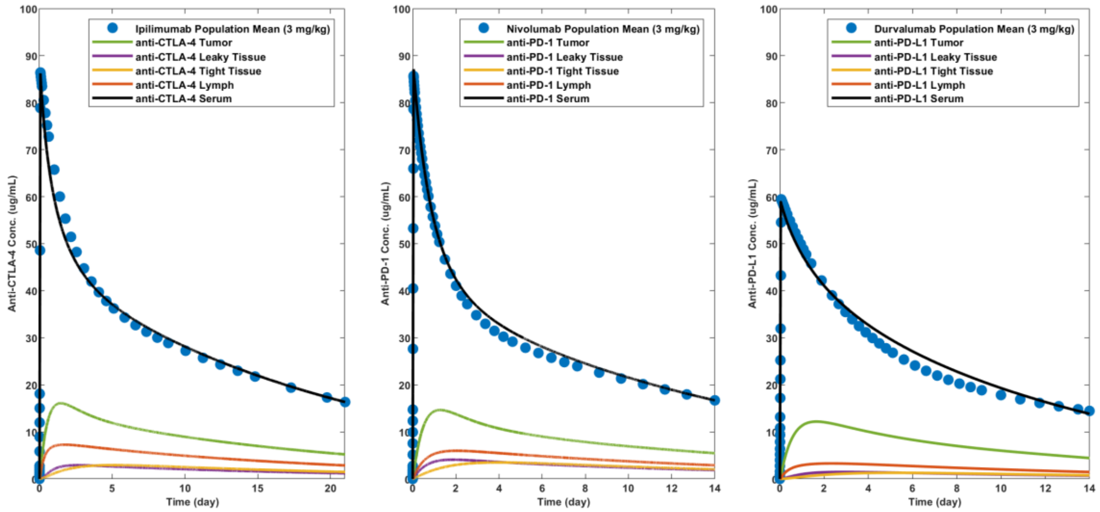

Figure S4. Pharmacokinetics of A) anti-CTLA-4, B) anti-PD-1 and C) anti-PD-L1 at 3 mg/kg each in the serum, tumor, leaky and tight tissues, lymph and serum. Results are based on a minimal-PBPK model, combined with tumor transport equations with diffusion across the vascular surface area.

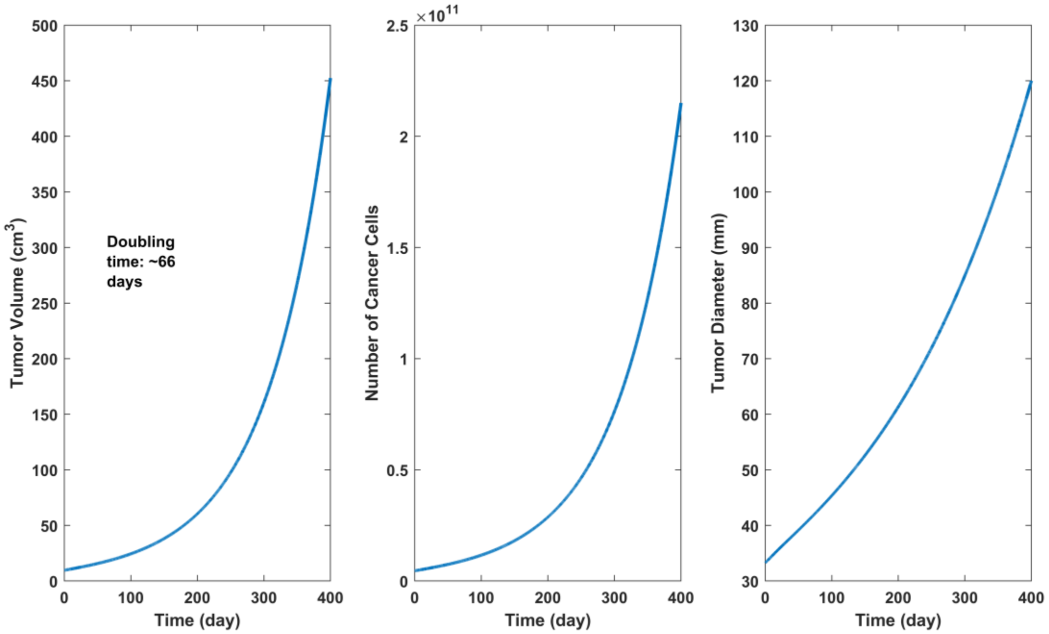

Figure S5. Simulation of tumor volume doubling time (left) and associated number of cancer cells (middle) and tumor diameter (right). A tumor doubling time of ~66 days was used for all model simulations. Variability in tumor growth was an emergent outcome for each simulation in the model.

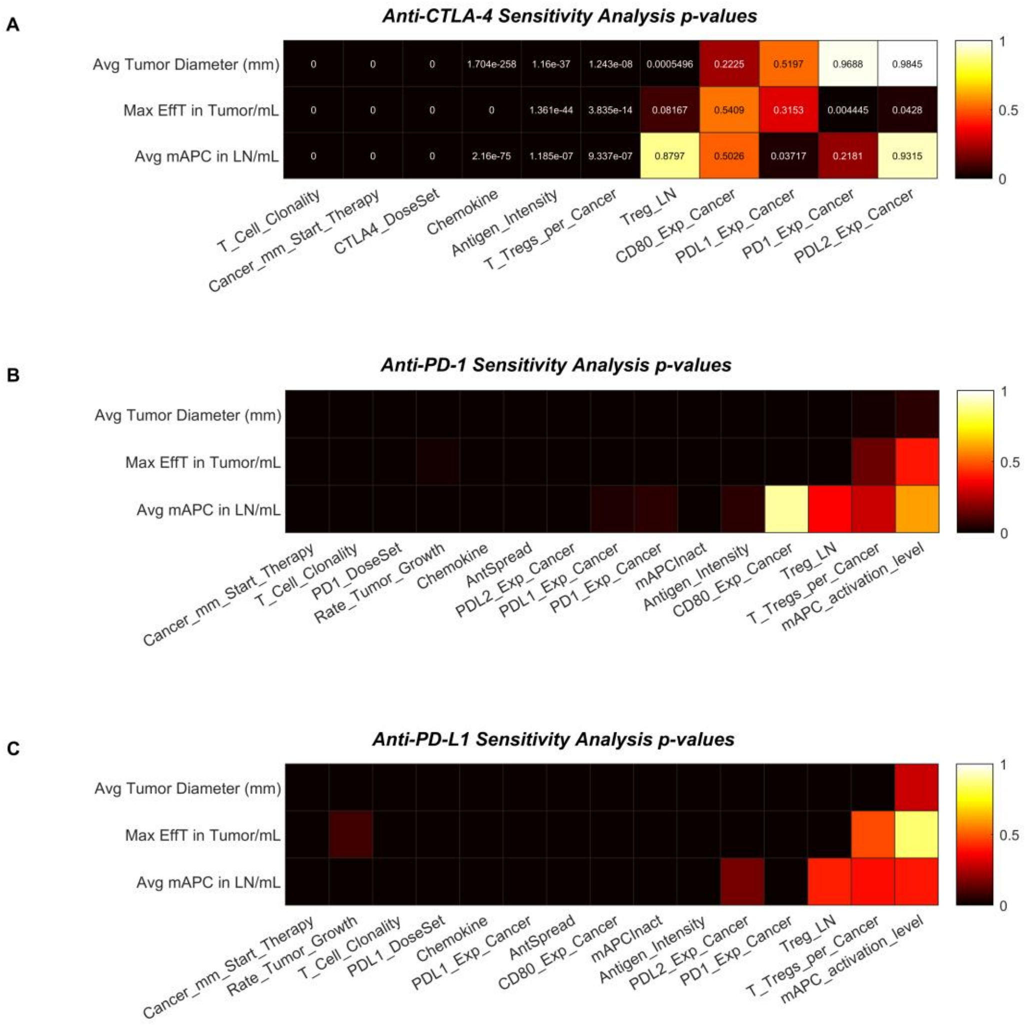

Figure S6. Sensitivity analysis p-values for A) anti-CTLA-4, B) anti-PD-1 and C) anti-PD-L1.

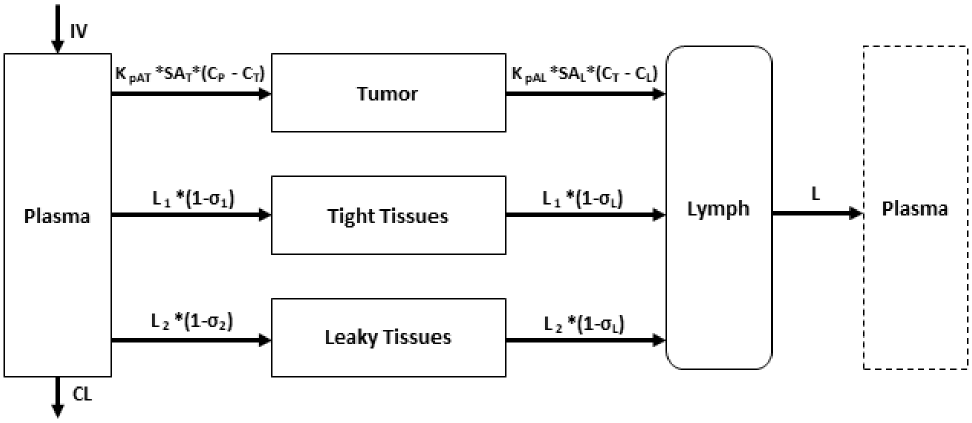

Figure S7. Minimal PBPK model of antibody distribution used in the QSP model.

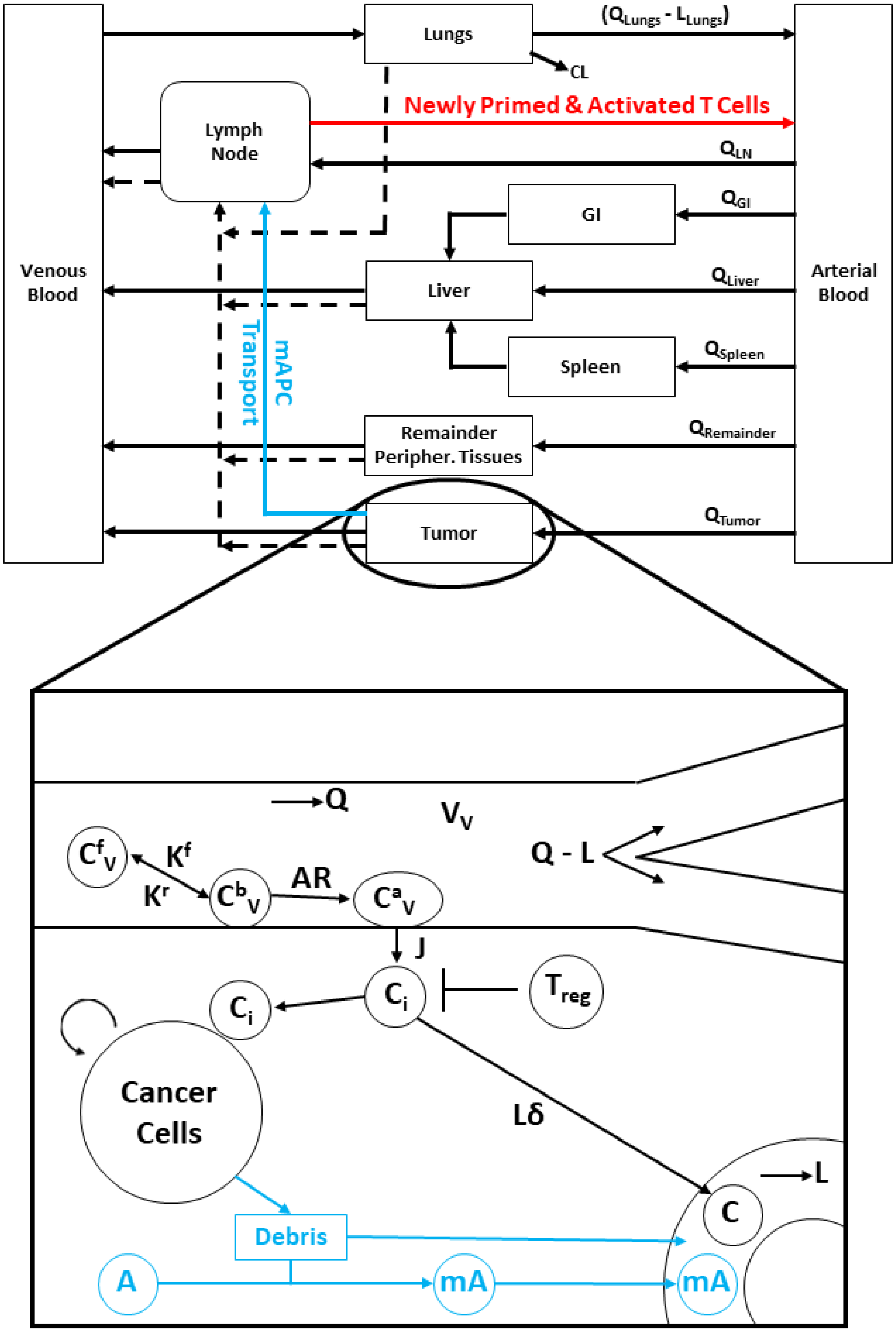

Figure S8. PBPK model cell trafficking used in the QSP model. Model PBPK of Effector T cell (C) trafficking through the tissues in the model via the blood (solid black lines) and lymph (dotted black

lines). Also represented is the trafficking of newly primed and activated Effector T cells from the lymph node to the blood for redistribution (solid red line) the trafficking of mature APCs (solid blue line) to the lymph nodes for priming and activation of naïve T cells to Effector T cells. Cancer Debris also enter the lymph fluid and is brought to the lymph node from the tumor. The bottom panel shows how free Effector cells (C^f_V) in the tumor vasculature are bound the vascular wall (C^b_V), arrested (C^a_V), extravasated (via rate J) into the interstitial space (C_i) to interact with the cancer cells. Regulatory cells (T_{reg}) inhibit the activity of the Effector cells in the tumor and APCs (A) pick up debris to become mAPC (mA). Effector cells can exit the interstitial space (via rate Lδ) and recirculate through the lymphatic system.

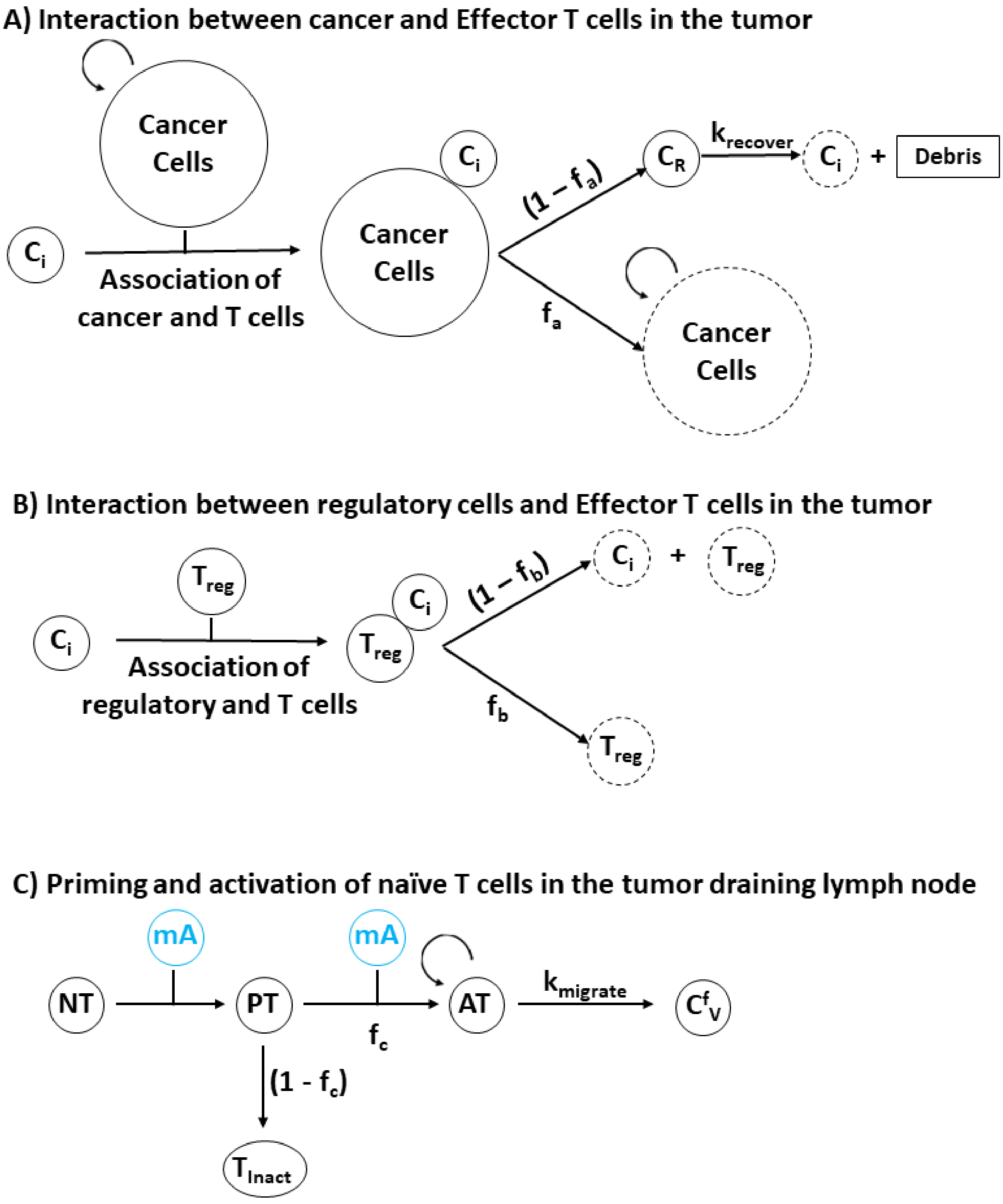

Figure S9. Abstract representation of cell-cell interactions in the model. A) Effector T cells in the interstitial space of the tumor (C_i) interact with cancer cells to form a bound complex (at a rate defined

by the De Pillis-Radunskaya equation described in the methods, equation 1.3). The complex then dissociates with the regeneration of cancer cells and Effector cells relative to the fraction of active checkpoint signaling (f_a). If checkpoint signaling is fully active at inhibiting the Effector cells, for example, then only the cancer cells are regenerated ($f_a = 1$). Alternatively, is antibodies block the signaling to full effect ($f_a = 0$), then only the Effector cells emerge from the bound interaction; along with cancer cell debris that can promote further priming and activation of Effector cells in the lymph nodes. The value of signaling comes out to be between 0 and 1 and is dependent on the checkpoint signaling and interactions with the antibodies that block it. The emerging Effector T cells undergo a recovery delay (C_R) before engaging in interactions with other cancers cells. B) Similarly, as described above, Effector T cells in the tumor interstitial space can interact with regulatory cells (T_{reg}) to form a state variable representing a bound complex. The dissociation of the bound complex determines to what extent the Effector cells are regenerated, which is directly a function of the checkpoint signaling (f_b). C) Mature APCs (mA) interact with naïve and primed T cells (NT and PT, respectively) during two priming stages in the lymph node. Depending on the extent of checkpoint signaling (f_c), primed T cells either become anergic (T_{Inact}) and/or activated T cells (AT); the latter proliferate and become Effector T cells (C^f_V) that migrate out into peripheral circulation.
